# New series of 4,6-diaryl pyrimidines: facile synthesis and antiproliferative activity as dual EGFR/VEGFR-2 inhibitors

**DOI:** 10.3389/fchem.2024.1498104

**Published:** 2024-11-06

**Authors:** Yaser A. Mostafa, Jalil Abdeljalil Assoud, Ahmed Y. Desoky, Samy Mohamady, Nesma M. Mohamed, Ola I. A. Salem, Zainab M. Almarhoon, Stefan Bräse, Bahaa G. M. Youssif

**Affiliations:** ^1^ Pharmaceutical Organic Chemistry Department, Faculty of Pharmacy, Assiut University, Assiut, Egypt; ^2^ Pharmaceutical Chemistry Department, Faculty of Pharmacy, Badr University in Assiut, Assiut, Egypt; ^3^ Department of Chemistry, University of Waterloo, Waterloo, ON, Canada; ^4^ Faculty of Pharmacy, The British University in Egypt, Al-Sherouk, Egypt; ^5^ Department of Pharmacognosy, Faculty of Pharmacy, Assiut University, Assiut, Egypt; ^6^ Pharmacognosy Department, Faculty of Pharmacy, Badr University in Assiut, Assiut, Egypt; ^7^ Department of Chemistry, College of Science, King Saud University, Riyadh, Saudi Arabia; ^8^ Institute of Biological and Chemical Systems, Institute of Biological and Chemical Systems-Functional Molecular Systems (IBCS-FMS), Karlsruhe Institute of Technology, Karlsruhe, Germany

**Keywords:** pyrimidine, synthesis, antiproliferative, protein kinase, docking, lipophilicity

## Abstract

**Introduction:**

We developed and produced a new series of 4,6-diaryl-pyrimidines **9–29** as antiproliferative agents targeting EGFR/VEGFR-2.

**Methods:**

The antiproliferative efficacy of the novel targets was assessed against a panel of 60 NCI cancer cell lines and four cancer cell lines *in vitro*.

**Results and Discussion:**

Compounds **14**, **17**, **19**, **22**, **25**, and **29** demonstrated the greatest potency among the derivatives, with GI_50_ values between 22 and 33 nM; compounds **22** and **29** exhibited the highest potency, with GI_50_ values of 22 and 24 nM, respectively. We subsequently examined the most efficient derivatives as dual EGFR/VEGFR-2 inhibitors, finding that compounds **22** and **29** functioned as dual inhibitors. Moreover, **22** and **29** can act as apoptotic inducers by increasing Bax levels and decreasing levels of the anti-apoptotic protein Bcl2. At both 24- and 48-h intervals, the cell migration rates of compounds **22** and **29** were lower than those of untreated cells, according to the migration rate and wound closure percentage assessment. The wound closure rate reached 100% after 72 h of therapy with compound **22** but only 80% with compound **29**. The docking study showed that compounds **22** and **29** had docking scores similar to those of Erlotinib and Sorafenib, co-crystallized ligands, for the EGFR and VEGFR-2 proteins. The experiments on lipophilicity showed that the new pyrimidines had a consistent result. This group of compounds has better biological activity in all the biological systems studied with low lipophilicity.

## 1 Introduction

Protein tyrosine kinases are important in transmitting signals that control numerous cellular functions, such as growth, specialization, mobility, and the development of new blood vessels (angiogenesis) (Lemmon and Schlessinger, 2010; Liao, 2007).

The epidermal growth factor receptor (EGFR) is a type of membrane receptor tyrosine kinase that is excessively expressed in various tumors. The signal transduction of EGFR tyrosine kinase is strongly linked to tumor progression. Therefore, inhibiting the activity of these receptors can effectively suppress tumor growth (Al-Wahaibi et al., 2024a; Mohassab et al., 2024; Mohamed et al., 2021; Antonello et al., 2006; Abourehab et al., 2021). Vascular endothelial growth factor receptor (VEGFR-2), another tyrosine kinase, is crucial in promoting angiogenesis (Holmes and Zachary, 2005; Modi and Kulkarni, 2019). VEGFR-2, a constituent of VEGFRs, has been demonstrated to be the primary mediator in tumor angiogenesis, a process essential for the growth of solid tumors. Inhibiting VEGFR-2 has been regarded as a successful approach to prevent angiogenesis (Peng et al., 2017; Ceci et al., 2020).

Pharmaceutical developers have invested decades in developing selective therapeutics for specific targets (Zhou et al., 2019). Despite the success of numerous single-target selective medicines, the advancement of multifactorial disorders such as cancer and neurodegenerative diseases involves multiple signaling pathways (Raghavendra et al., 2018). As a result, there is growing interest in developing medicines that address multiple objectives at once. There are presently two approaches for developing multi-targeted medications. The initial strategy entails establishing an additive or synergistic effect by utilizing multiple drugs that act on distinct targets via combination drug therapy. The FDA endorsed the use of a combination of dabrafenib (a BRAF inhibitor) and trametinib (a MEK inhibitor) for treating metastatic melanoma with BRAF mutations (Wahid et al., 2018).

The second strategy involves the design and generation of multi-targeted therapeutics that collaboratively inhibit many carcinogenic pathways (Al-Wahaibi et al., 2024b). The approach of multi-targeting therapeutics involves identifying a single agent capable of acting on two or more targets concurrently. The FDA approved cabozantinib, also known as cabometyx, as a small-molecule dual-targeting inhibitor of the tyrosine kinases c-Met (mesenchymal-epithelial transition factor) and VEGFR-2 (Vascular Endothelial Growth Factor Receptor), demonstrating its ability to inhibit tumor growth, metastasis, and angiogenesis (Abou-Alfa et al., 2018). The EGFR and VEGFR-2 pathways are closely interconnected, sharing common downstream signaling pathways. In addition to impacting the growth of cancer cells, the activation of EGFR also promotes the formation of new blood vessels (angiogenesis). In certain instances, inhibiting EGFR can cause an increase in the expression of VEGFR-2, which in turn speeds up the signaling for tumor growth independently of EGFR. This can result in the development of resistance to EGFR inhibitors (Liu et al., 2023; Li et al., 2022). The emergence of secondary drug resistance after initiating treatment with EGFR inhibitors poses a significant obstacle in cancer therapy and warrants the exploration of novel therapeutic options (Ward et al., 2020). Hence, the simultaneous suppression of both EGFR and VEGFR-2 holds great potential as a cancer treatment strategy due to its synergistic impact (Liu et al., 2023).

**FIGURE 1:**
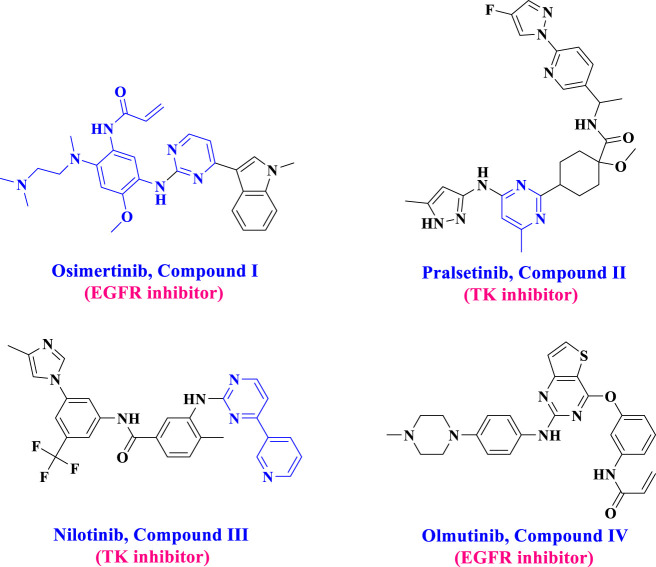
Structure of FDA-approved drugs **I-IV**.

Pyrimidine derivatives have garnered significant attention from researchers recently due to their diverse biological activities, including anticancer properties (Al-Wahaibi et al., 2024a; Ward et al., 2020; Liu et al., 2023) and their effects on the cardiovascular system and bronchodilation (Heber et al., 1993). The core component of some FDA-approved drugs, such as Olmutinib **(I)**, Pralsetinib **(II)**, Nilotinib **(III)**, and Osimertinib **(IV)** (Figure 1), has a pyrimidine ring (Guo et al., 2023; Al-Huseini et al., 2023). Moreover, due to their synthetic flexibility, several substituents can substitute carbon atoms 2, 4, 5, or 6, producing many derivatives. Furthermore, pyrimidines can establish hydrogen bonds with various targets, giving them distinct physicochemical properties that improve the pharmacokinetics and pharmacodynamics of drugs (Ali et al., 2019; Mallinson and Collins, 2012).

**FIGURE 2:**
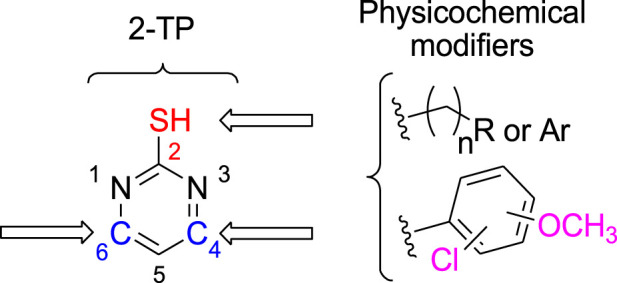
Design of antiproliferative pyrimidine-based derivatives **9–29**.

2-thiopyrimidines (2-TPs) are widely recognized pyrimidine derivatives with antiproliferative activity against leukemia, breast, and colon cell lines (Abdel-Mohsen et al., 2019; Abdel-Mohsen et al., 2019; Ali et al., 2022; Mahapatra et al., 2021). This study proposes that adding an aryl ring to the 2-TP ring at positions 4 and 6, which can form hydrogen bonds with proteins and nucleic acids, will modify the ring and result in antiproliferative properties. When substituents at positions 2, 4, or 6 are changed, the physicochemical features of 2-TPs change, such as lipophilicity, which affects their capacity to enter cell membranes and, hence, their antiproliferative effect. As far as we know, no studies have experimentally tested the lipophilicity of various reported derivatives of 2-TP. However, in this study, we presented the lipophilicity of our novel 2-TPs, which was assessed by both theoretical and experimental methods. The aim was to establish a correlation between lipophilicity and anticancer effectiveness. To enhance the antiproliferative effect of 2-TPs by inhibiting tumor angiogenesis regulators, the 2-thiol moiety was changed with either small, (un)branched alkyl groups or bulky benzylic groups. Two phenyl rings containing either methoxy or chloro groups were inserted at the 4- and 6-positions of the pyrimidine ring, as shown in Figure 2.

Consequently, the current study focuses on synthesizing novel 4,6-diaryl pyrimidine derivatives for developing antiproliferative drugs (Figure 2). The novel chemicals were tested for cytotoxic activity against four human cancer cell lines. In addition, we investigated compounds with potential anticancer activities to see if they may act as dual inhibitors of EGFR and VEGFR-2 in order to get insight into their biological process. The cytotoxicity of the most potent compounds was further confirmed by determining their ability to cause apoptosis, as shown by the Bax/Bcl2 ratio. Concurrently, the physicochemical characteristics and molecular docking of the produced compounds with the EGFR and VEGFR-2 binding sites were investigated. This study sought to determine the mechanism of inhibitor binding.

## 2 Results and discussion

### 2.1 Chemistry

**Scheme 1:**
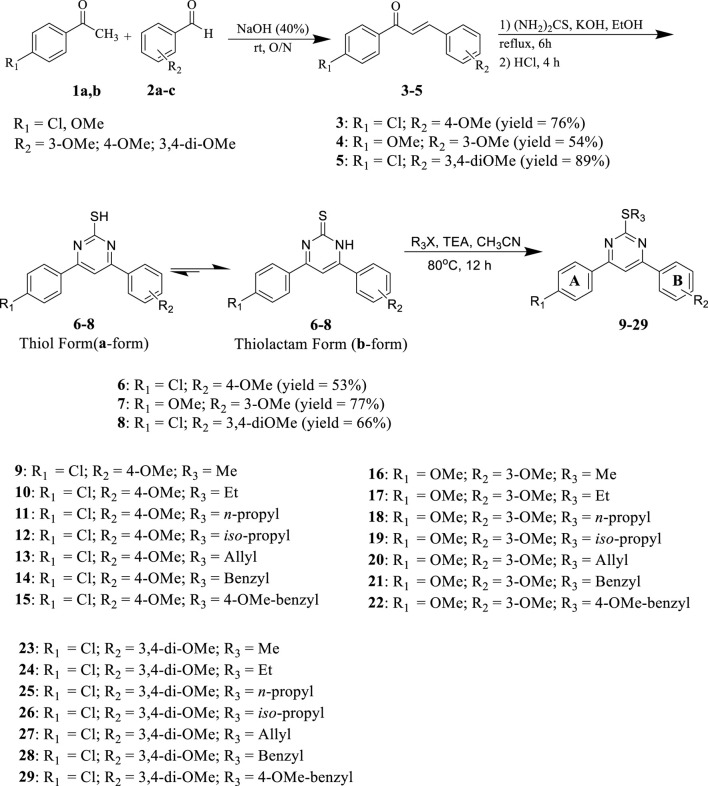
Synthesis of target Pyrimidines **9–29**

Scheme 1 details the synthesis of key intermediates **6-8** and target compounds **9–29**. Target compounds **9–29** were synthesized using mixed aldol condensation of either 4-chloro or 4-methoxy acetophenone **1** with different benzaldehydes **2a-c** to yield chalcones **3–5** (Richard et al., 2023; Jebapriya et al., 2021). These chalcones were then cyclocondensed via Michael addition with thiourea as a nitrogen source under basic conditions (Sánchez-Sancho et al., 2022), yielding pyrimidine-2(1*H*)-thione/2-thiol intermediates **6–8**. These intermediates were subsequently alkylated with alkyl, allyl, and/or aralkyl halides to yield the target pyrimidines **9–29**.

**FIGURE 3:**
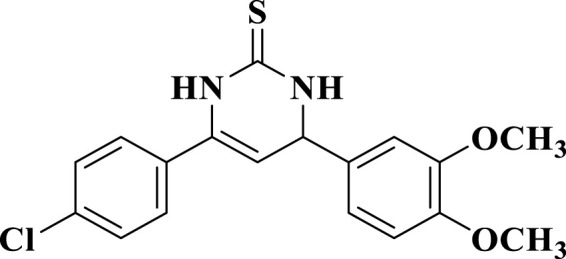
Thiouridine (**c-form**) of compound **8**.

According to the literature, it is important to note that the key intermediates **6-8** are mainly present in the pyrimidine-2(1*H*)-thione (thiolactam; **b-form**) tautomeric form rather than the pyrimidine-2-thiols tautomeric form (**a-form**) (Wahid et al., 2018; Ward et al., 2020; Liu et al., 2023). This was observed in two key intermediates, compounds **6** and **7**. The ^1^H NMR spectra of intermediates **6** and **7** in CDCl_3_ revealed that they were mostly found in the pyrimidine-2(1*H*)-thione (**b-form**) form. Two signals at δ = 5.19 and 5.27 ppm recognized the intermediates, corresponding to N_1_-H and olefinic C_5_-H, respectively. Due to its low solubility, we could not get a ^1^H NMR for the third intermediate **8** in CDCl_3_. However, the ^1^H NMR spectra of intermediate **8** in DMSO-*d*_*6*_ exhibited an unexpected pattern, showing that **8** is in the ureide-like 3,4-dihydropyrimidine-2(1*H*)-thione tautomeric form (**c-form**), as seen in Figure 3.

**FIGURE 4:**
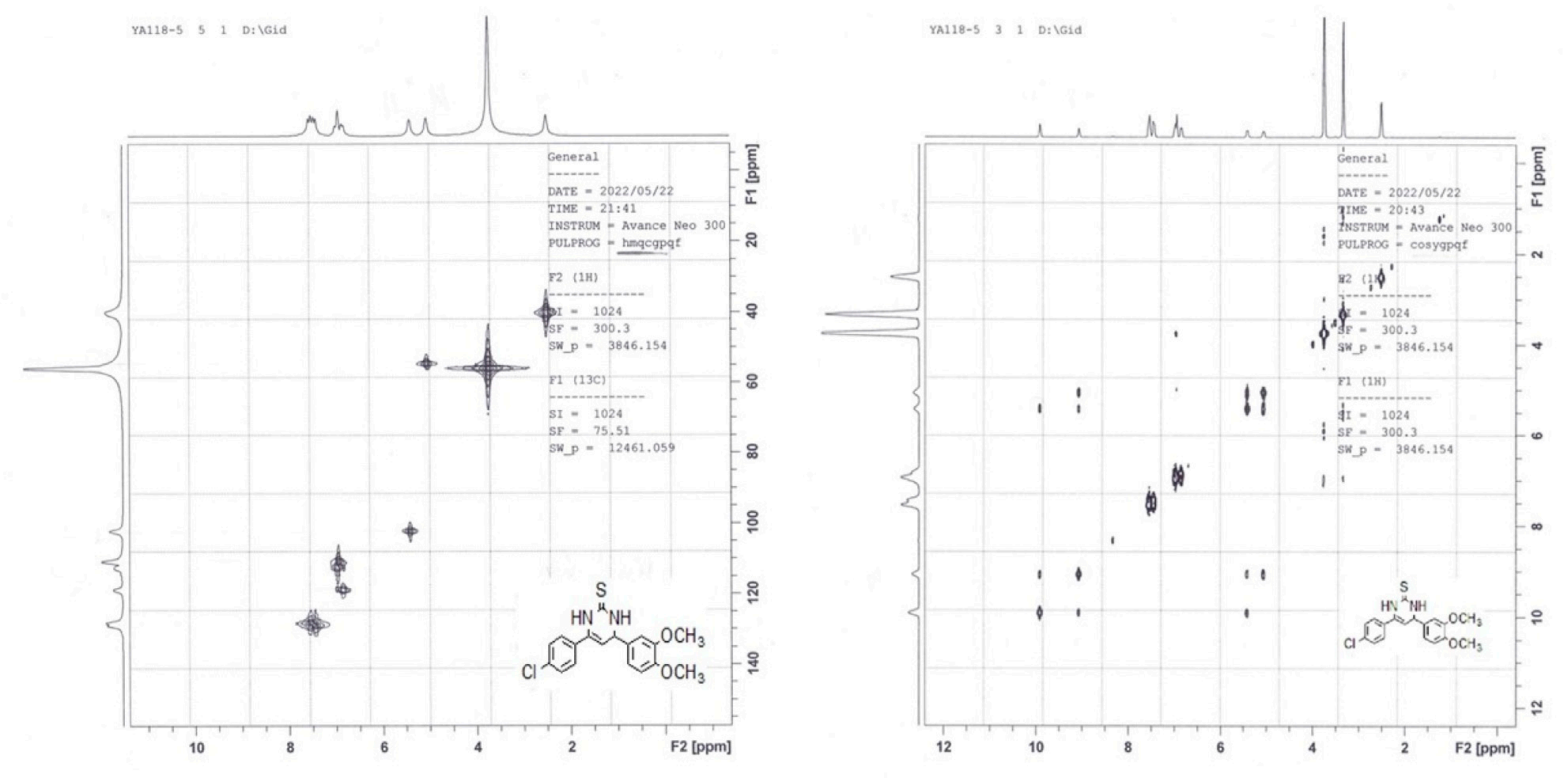
HMQC and H-H cosy of compound **8** in DMSO-*d*_*6*_.

This form differs from the other thiolactam form (**b-form**) by having new signals at δ 9 and 10 ppm corresponding to N_1_-H (c.f. 5.27 ppm of b-form) and N_3_-H atoms of the pyrimidine ring, in addition to a signal at δ = 5 ppm corresponding to the C_4_-H. In 1978, Heber et al. (1993) reported that these pyrimidines are primarily present in the thiol form in non-polar solvents and in the thione form in polar solvents such ethanol (EtOH), chloroform (CHCl_3_), and dimethyl sulfoxide (DMSO). Therefore, we repeated the ^1^H NMR experiment for one of these intermediates, which had previously been observed in the **b-form**. However, instead of CDCl_3_, we utilized DMSO-*d*_*6*_ as the solvent (Baddar et al.). We observed the same three signals for N_1_-H, N_3_-H, and C_4_-H, which indicated the presence of the c-tautomeric form based on their chemical shifts. Based on these findings, we decided to explore the structural features of such tautomeric forms of derivative **8**. We ran a jmod ^13^C NMR (which places the CH_2_ and quaternary C’s in the positive phase and both CH and CH_3_ in the negative phase) experiment on the me intermediate **8**. We found 7 signals of quaternary carbons (5 of aromatic quaternary C’s, one of olefinic C_6_, and one of thione carbon at δ = 175.4 ppm), and 5 signals of aromatic CH’s, one signal for olefinic C_5_-H at = 102.3 ppm, one signal for allylic C_4_-H at δ = 54.7 ppm, and two overlapped signals of OCH_3_ groups at δ = 55.9 ppm. Additionally, running two different 2D NMR experiments on the same intermediate (**8**) revealed the following characteristics: The HMQC experiment showed a cross peak of C_4_-H and C_5_-H, in addition to the disappearance of signals corresponding to pyrimidine ring N_1_-H and N_3_-H (as shown in Figure 4).

On the other hand, the H-H Cosy experiment showed spin-spin coupling between pyrimidine H_4_ at δ = 5.06 ppm and both H_5_ at δ = 5.41 ppm and H_3_ at δ = 9.06 ppm, while pyrimidine H_5_ showed in addition to its coupling to H_4_, two long-range couplings with both H_1_ and H_3_, as shown in Figure 4. Finally, positive mode ESI mass spectrometric analysis showed two peaks for molecular ion peak (M + H) at m/z = 361 and its isomeric peak (M + H+2) at m/z = 363, which confirms all the previous findings about the presence of this intermediate in the 3,4-dihydropyrimidine-2(1*H*)-thione form (**c-form**) rather than either the thiol (**a-form**) or the thione (**b-form**) tautomeric forms.

### 2.2 Single crystal X-ray diffraction of *S*-Benzyl derivative (compound 14)

**FIGURE 5:**
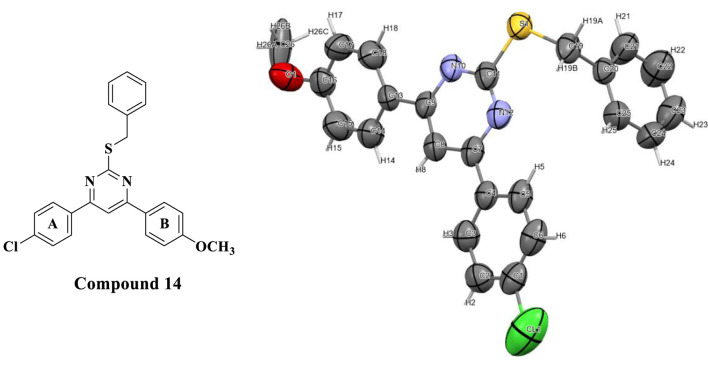
The asymmetric unit of compound **14** represented as an ORTEP plot with Ellipsoids of thermal displacement at 50% probability.

To increase the significance of this study by gaining extra relevant data on the structure of this specific class of compounds, we have decided to perform an X-ray crystallography analysis. Figure 5 shows a successful single crystal X-ray image obtained using derivative **14**. The graphic showed an ORTEP plot with an ellipsoid portrayal of all the atoms in its structure.

**FIGURE 6:**
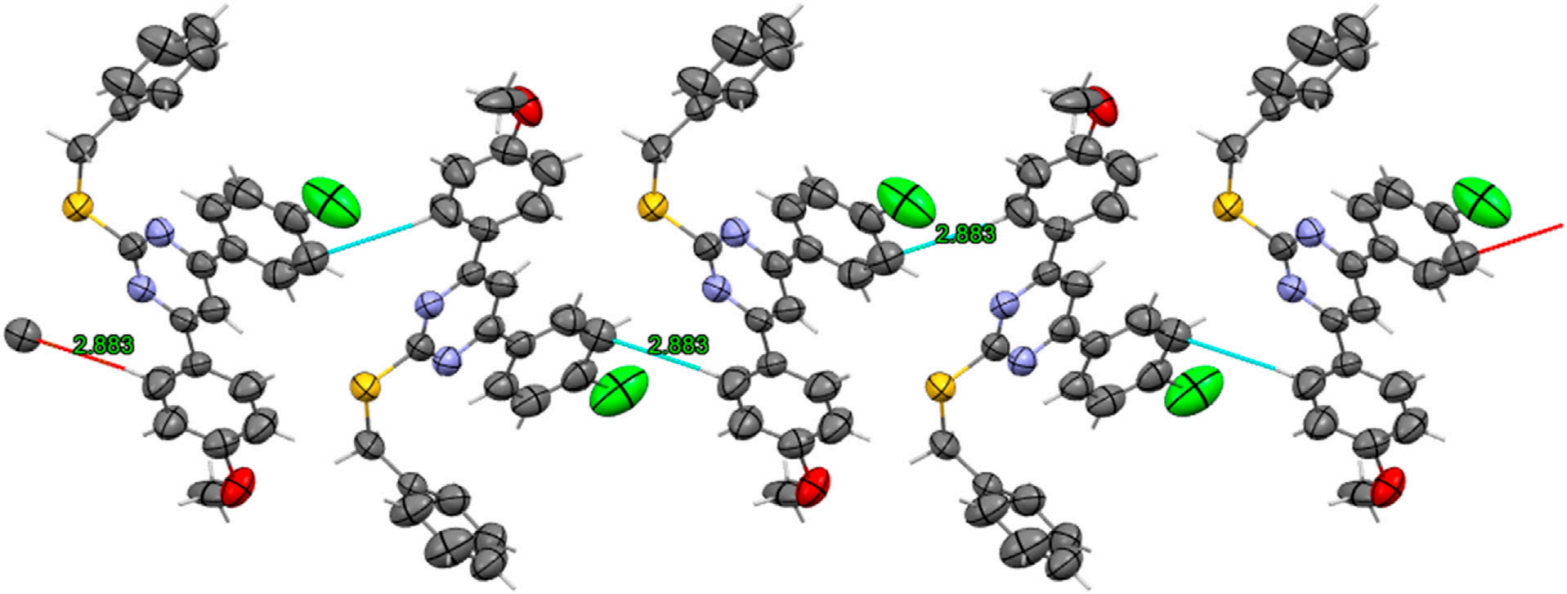
Wireframe representation of Orthorhombic Unit showing hybridization of heteroatoms, in addition to short contact networks between molecules of compound **14**.

The ORTEP plot shows that the pyrimidine ring, its 2 aryl substituents (rings A and B), and the S-benzyl group were found on pyrimidine C-2. Additionally, the asymmetric unit was found to be composed of three molecules of compound **14** in close contact with each other with a 2.883 Å van der Waals attractive force, as shown in the wireframe figure below (Figure 6).

### 2.3 Biology

#### 2.3.1 *In vitro* NCI antiproliferative screening

The National Cancer Institute’s Developmental Therapeutic Program (www.dtp.nci.nih.gov) has tested compounds **9–29** (except compounds **21** and **22**) against 60 cancer cell lines from nine types (leukemia, lung, colon, CNS, melanoma, ovarian, renal, prostate, and breast cancer) at a single dose (10 μM) (El-Sherief et al., 2018). Supplementary Tables S1, S2 provide further information (see the Supplementary Material file). As indicated in Table 1, various compounds selectively suppressed the growth of various cancer cells, with percentage inhibition values more than or equal to 40. The most sensitive cells included leukemia (SR, K-562, and MOLT-4), lung (NCI-H522, RPMI-8226, and EKVX), breast (MCF-7 and T-47D), colon (HCT-15 and HT-29), and ovarian (NCI/ADR-RES).

**TABLE 1:** Compounds with the highest % inhibition in sensitive cell lines.

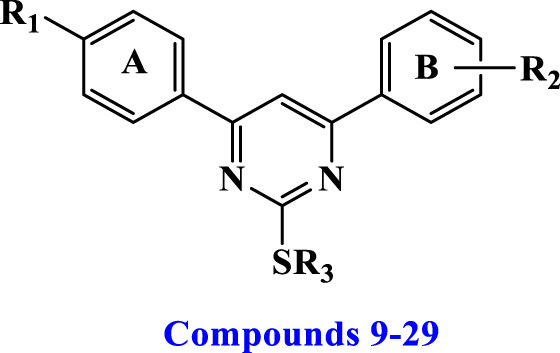			
Compound No.	Cell line	Cancer type	% inhibition at 10 µM
12	HL-60 (TB)	Leukemia	32.59
	MOLT-4		29.88
	K-562		30.86
	SR		**47.1**
	HCT-15	Colon cancer	38.68
	HT-29		29.24
	MCF7	Breast cancer	28.79
	T-47D		**40.44**
	MDA-MB-468		35.14
19	K-562	Leukemia	29.5
	HT 29	Colon cancer	32.48
20	HL-60 (TB)	Leukemia	31.34
23	K-562	Leukemia	**39.05**
	SR		34.01
	HCT-116	Colon cancer	33.72
24	K-562	Leukemia	**41.5**
	MOLT-4		36.97
	SR		**39.6**
	COLO 205	Colon cancer	35.43
	HCT-15		30.71
	HT29		28.29
	KM12		29.81
	T-47D	Breast cancer	37.62
	K-562	Leukemia	27.05
25	COLO 205	Colon cancer	31.68
	T-47D	Breast cancer	34.51
	HL-60 (TB)	Leukemia	29.25
27	K-562	Leukemia Melanoma	**41.97**
	SR		30.31
	UACC-62		25.96
	K-562	Leukemia	30.95
28	MOLT-4	Leukemia Non-Small Cell Lung cancer	32.81
	SR		**39.44**
	NCI-H522		31.82
	HCT-15	Colon cancer	27.64
	SK-MEL-5	Melanoma	30.09
	T-47D	Breast cancer	32.97
	HL-60 (TB)	Leukemia	38.84
29	K-562	Leukemia Non-Small Cell Lung cancer	**54.54**
	MOLT-4		**46.5**
	RPMI-8226		36.9
	SR		**45.8**
	A549/ATCC		30.07
	EKVX	Non-Small Cell Lung cancer Colon cancer	29.67
	HCT-116		30.23
	HCT-15	Colon cancer CNS cancer	**42.15**
	HT29		38.02
	SF-268		32.98
	UACC-62	Melanoma	38.69
	NCI/ADR-RES	Ovarian cancer	32.11
	SN12C	Renal cancer	35.57
	UO-31	Renal cancer Prostate cancer	33.09
	PC-3		36.44
	DU-145	Prostate cancer Breast cancer	28.55
	MCF7		27.37
	MDA-MB231/ATCC	Breast cancer	30.34
	HS 578 T		37.47
	T-47D		34.57
	MDA-MB-468		**40.21**

Pyrimidine derivatives **12** (R_1_ = 4-Cl, R_2_ = 4-OMe, R_3_ = *iso*-propyl), **23** (R_1_ = 4-Cl, R_2_ = 3,4-di-OMe, R_3_ = Me), **24** (R_1_ = 4-Cl, R_2_ = 3,4-di-OMe, R_3_ = Et), **25** (R_1_ = 4-Cl, R_2_ = 3,4-di-OMe, R_3_ = *n*-propyl), **28** (R_1_ = 4-Cl, R_2_ = 3,4-di-OMe, R_3_ = benzyl), and **29** (R_1_ = 4-Cl, R_2_ = 3,4-di-OMe, R_3_ = 4-OMe-benzyl) exhibited the highest activity, indicating that derivatives with a *p*-chloro substituent on the A-ring and a 3,4-dimethoxy substituent on the B-ring consistently showed the greatest percentage of inhibition compared to other similar compounds. Unfortunately, none of the tested compounds were selected for the five-dose experiment. Therefore, we decided to test compounds **9–29** against a panel of four cancer cell lines.

#### 2.3.2 Cell viability assay

This experiment examines the impact of the newly developed compounds **9–29** on normal cell lines to assess their safety level. The vitality of the investigated compounds was assessed using the MCF-10 A cell line, a normal human mammary gland epithelial cell line. Following 4 days of incubation on MCF-10 A cells with each examined compound at a concentration of 50 μM, the vitality of the cells was assessed using the MTT test (Mahmoud et al., 2022; Mekheimer et al., 2022). The results from Table 2 indicate that none of the compounds tested exhibited cytotoxicity, and all compounds displayed cell viability of over 87% at a concentration of 50 µM.

**TABLE 2:** IC_50_ values of compounds **9–29** and Erlotinib against four cancer cell lines.

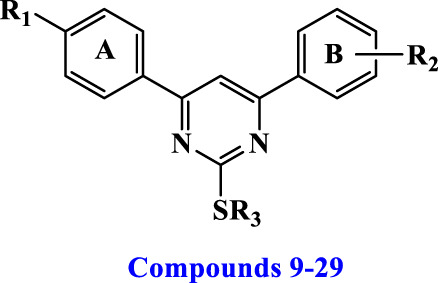						
Comp	Cell viability % (MCF-10 A)	Antiproliferative activity IC50 ± SEM (nM)				
		A-549	MCF-7	Panc-1	HT-29	Average IC_50_ (GI_50_)
9	91	60 ± 5	65 ± 6	62 ± 6	62 ± 6	62
10	90	75 ± 7	79 ± 7	76 ± 7	74 ± 7	76
11	89	52 ± 5	55 ± 5	54 ± 5	53 ± 5	54
12	89	35 ± 3	37 ± 3	36 ± 3	36 ± 3	36
13	90	64 ± 6	68 ± 6	66 ± 6	64 ± 6	66
14	90	28 ± 2	30 ± 2	29 ± 2	28 ± 2	29
15	91	79 ± 7	82 ± 7	80 ± 7	80 ± 7	80
16	92	86 ± 7	89 ± 8	85 ± 8	85 ± 8	86
17	90	28 ± 2	30 ± 3	28 ± 2	29 ± 2	29
18	87	45 ± 4	46 ± 4	44 ± 4	46 ± 4	45
19	90	29 ± 2	32 ± 3	30 ± 2	30 ± 2	30
20	90	70 ± 6	73 ± 6	70 ± 6	72 ± 4	71
21	89	83 ± 7	86 ± 8	82 ± 8	82 ± 8	83
22	89	21 ± 2	23 ± 2	22 ± 2	23 ± 2	22
23	90	48 ± 4	50 ± 4	47 ± 4	48 ± 4	48
24	91	30 ± 3	33 ± 3	30 ± 3	31 ± 3	31
25	91	32 ± 3	34 ± 3	32 ± 3	32 ± 3	33
26	90	40 ± 3	42 ± 3	40 ± 3	42 ± 3	41
27	89	56 ± 5	60 ± 6	58 ± 5	60 ± 6	59
28	92	39 ± 3	43 ± 3	40 ± 3	41 ± 3	41
29	91	23 ± 2	25 ± 2	23 ± 2	24 ± 2	24
Erlotinib	ND	30 ± 3	40 ± 3	30 ± 3	30 ± 3	33

#### 2.3.3 Antiproliferative assay

The antiproliferative activity of new compounds **9–29** was examined against four human cancer cell lines (colon - HT-29, pancreatic - Panc-1, lung - A-549, and breast - MCF-7) using Erlotinib as a reference. The MTT test was employed for this investigation (Hisham et al., 2022; El-Sherief et al., 2019; Ramadan et al., 2020). Table 2 presents the four cancer cell lines’ median inhibitory concentration (IC_50_) and GI_50_ (average IC_50_) values.

In general, the studied compounds **9–29** demonstrated strong antiproliferative activity against the four cancer cell lines tested, with GI_50_ values ranging from 22 nM to 86 nM, in comparison to the standard Erlotinib, which had a GI_50_ value of 33 nM. Compounds **14**, **17**, **19**, **22**, **25**, and **29** exhibited the highest potency among the derivatives, with GI_50_ values ranging from 22 to 33 nM.

Among the newly synthesized derivatives **9–29**, Compounds **22** (R_1_ = 4-OMe, R_2_ = 3-OMe, R_3_ = 4-OMe-benzyl) and **29** (R_1_ = 4-Cl, R_2_ = 3,4-di-OMe, R_3_ = 4-OMe-benzyl) have the highest potency with GI_50_ values of 22 and 24 nM, respectively, which is 1.5 times greater than the reference Erlotinib (GI_50_ = 33 nM). Compounds **22** and **29** exhibited greater potency than the reference drug Erlotinib against all four tested cancer cell lines.

According to the findings, the type of substitution found on the sulfur atom at position two of the di-aryl pyrimidine moiety appears critical for antiproliferative action. Compounds **16** (R_1_ = 4-OMe, R_2_ = 3-OMe, R_3_ = Me), **17** (R_1_ = 4-OMe, R_2_ = 3-OMe, R_3_ = Et), **18** (R_1_ = 4-OMe, R_2_ = 3-OMe, R_3_ = *n*-propyl), **19** (R_1_ = 4-OMe, R_2_ = 3-OMe, R_3_ = *iso*-propyl), **20** (R_1_ = 4-OMe, R_2_ = 3-OMe, R_3_ = ally), and **21** (R_1_ = 4-OMe, R_2_ = 3-OMe, R_3_ = 4-benzyl), which share the same molecular structure as compound **22** but have different substituent groups attached to the sulphur atom, exhibited GI_50_ values of 86, 29, 45, 30, 71, and 83 nM, respectively. In all cases, these compounds have lower efficacy than compound **22**, suggesting that the 4-OMe-benzyl group at position two is critical for the antiproliferative action.

This hypothesis is supported by the results of compound **29** (R_1_ = 4-Cl, R_2_ = 3,4-di-OMe, R_3_ = 4-OMe-benzyl), which ranks second in terms of biological efficacy and, despite containing different groups (R_1_ and R_2_) on the two aryl rings (position 4 and 6), still has a 4-OMe-benzyl group on the sulfur atom at position 2. In addition, when comparing the GI_50_ values of compound **29** with compounds **25–28**, which have the same structure but differ only in the substitution at position 2 of the sulfur atom, compound **29** exhibits greater potency than compounds **25–28**, Table 1. This provides further evidence supporting the significance of the 4-MeO-benzyl group for antiproliferative activity.

The substitution of the di-aryl pyrimidine moiety at the 4 and/or 6-positions is crucial for activity and is another topic of focus. The GI_50_ values for compounds **22** (R_1_ = 4-OMe, R_2_ = 3-OMe, R_3_ = 4-OMe-benzyl), **29** (R_1_ = 4-Cl, R_2_ = 3,4-di-OMe, R_3_ = 4-OMe-benzyl), and **15** (R_1_ = 4-Cl, R_2_ = 4-OMe, R_3_ = 4-OMe-benzyl) were 22, 24, and 80 nM, respectively. These data show that the 4-position substitution has a significant effect on the antiproliferative activity of these compounds, with the highest activity shown in compounds with a 4-Cl phenyl or 4-OMe phenyl groups, whereas in the 6-position, both 3-OMe phenyl or 3,4-di-OMe phenyl are tolerated for antiproliferative action, but 4-OMe phenyl is not favored.

ND: Not Determined.

#### 2.3.4 EGFR inhibitory assay

The EGFR-TK assay (Al-Wahaibi et al., 2022; Alshammari et al., 2022) was used to assess the inhibitory activity of the most potent antiproliferative compounds **14**, **17**, **19**, **22**, **25**, and **29**, against EGFR. The results are shown in Table 3. This assay’s results are congruent with those from the antiproliferative assay. Compounds **22** (R_1_ = 4-OMe, R_2_ = 3-OMe, R_3_ = 4-OMe-benzyl) and **29** (R_1_ = 4-Cl, R_2_ = 3,4-di-OMe, R_3_ = 4-OMe-benzyl) proved to be the most efficient antiproliferative hybrids and EGFR inhibitor derivatives. Their IC_50_ values were 74 ± 5 nM and 72 ± 5 nM, respectively, surpassing the reference medication Erlotinib (IC_50_ = 80 ± 5).

**TABLE 3:** IC_50_ values of compounds **14**, **17**, **19**, **22**, **25**, and **29** against EGFR and VEGFR-2.

Compound	EGFR inhibition IC_50_ ± SEM (nM)	VEGFR-2 inhibition IC_50_ ± SEM (nM)
14	90 ± 7	2.20 ± 0.02
17	87 ± 6	1.85 ± 0.01
19	83 ± 6	2.70 ± 0.02
22	74 ± 5	1.15 ± 0.01
25	78 ± 5	2.95 ± 0.02
29	72 ± 5	1.60 ± 0.01
Erlotinib	80 ± 5	ND
Sorafenib	ND	0.17 ± 0.01

Compounds **14**, **17**, **19**, and **25** exhibited noteworthy inhibitory action against EGFR, with IC_50_ values of 90, 87, 83, and 78 nM, respectively, relatively similar to the reference compound Erlotinib. These results indicate that compounds **22** and **29** exhibit substantial inhibitory activity against EGFR and can potentially be utilized as medicines that prevent cell proliferation.

#### 2.3.5 VEGFR-2 inhibitory assay

An *in vitro* study examined the anti-VEGFR-2 activity of compounds **14**, **17**, **19**, **22**, **25**, and **29** (Marzouk et al., 2020; Mahmoud et al., 2024). The enzyme assay revealed that the six hybrids studied significantly inhibited VEGFR-2, with IC_50_ values ranging from 1.15 to 2.95 nM (Table 3). In all instances, the IC_50_ values of the tested compounds are higher (less potent) than that of the reference Sorafenib (IC_50_ = 0.17 nM). Compounds **22** and **29** had the highest inhibitory action against VEGFR-2, with IC_50_ values of 1.15 and 1.60 nM, respectively. Additionally, these compounds were potent inhibitors of cancer cell growth, with GI_50_ values of 22 and 24 nM, respectively. These data demonstrated that compounds **22** and **29** exhibit efficacy as antiproliferative agents by acting as inhibitors for both EGFR and VEGFR-2.

#### 2.3.6 Targeting apoptosis modulators

The balance of two proteins, Bax and Bcl2, controls apoptosis, a programmed cell death program (PCD) (Ouyang et al., 2012). The Bax protein, a member of the BCL-2 gene family that contains other apoptosis regulators, is known for triggering programmed cell death. The Bcl-2 protein, another family member, was recognized for suppressing apoptosis (Youssif et al., 2019).

Compounds **22** and **29**, the most potent derivatives, were further investigated against the Bax/Bcl2 ratio using Staurosporine as the reference drug (Youssif et al., 2019). Compounds **22** and **29** increased Bax levels by up to 294 and 278 pg/mL compared to staurosporine (280 pg/mL), a 37-fold and 35-fold improvement compared to untreated A-549 cancer cells, Table 4.

**TABLE 4:** Inhibition studies on tumor angiogenesis regulators and wound closure %.

Compound number	Bax		Bcl-2		Wound closure %		
	Conc (pg/mL)	Fold change	Conc (ng/mL)	Fold reduction	24 h	48 h	72 h
22	294 ± 6	37	0.90	6	25	50	100
29	278 ± 5	35	1.15	5	11	34	80
Staurosporine	280 ± 5	35	1.10	5	--	--	--
Control	8	1	5	1	37	75	100

Also, compound **22** caused a big drop in the amount of Bcl-2 protein (0.90 ng/mL), followed by compound **29** (1.10 ng/mL) in the A-549 cell line compared to staurosporine (1.10 ng/mL). The apoptosis experiment showed that compounds **22** and **29** have dual inhibitory effects on EGFR and VEGFR-2 and a strong effect on stopping cell growth through apoptosis.

#### 2.3.7 Estimation of migration rate and wound closure percentage

Cancer invasion and the ability of malignant tumor cells for direct migration and metastasis are two main patterns exhibited by tumor cells to overcome barriers of the extracellular matrix and spread into surrounding tissues (Wu et al., 2021). Wound healing assay is a simple, reproducible, and non-expensive method to study cancer cell migration *in vitro* (Rodriguez et al., 2005). We investigated the wound-healing activities of compounds **22** and **29** in A-549 cancer cells. Table 4 illustrates that the cell migration rates of compounds **22** and **29** were inferior to those of untreated cells at both 24- and 48 h intervals. Furthermore, the wound closure percentage achieved 100% after 72 h of treatment with a compound **22**, whereas it reached only 80% with a compound **29**. These results demonstrate the potential of this family of compounds to inhibit the invasive propensity of cancers, particularly malignant ones.

### 2.4 Docking study

Docking simulations of best 6 pyrimidine derivatives **14**, **17**, **19**, **22**, **25**, and **29** within the active site of both EGFR and VEGFR-2 proteins.

To validate the docking study, Erlotinib (for 1M17 as crystal structure of EGFR) and Sorafenib (for 3WZE as crystal structure of VEGFR-2 ( were docked into the binding site using a set of parameters of minimization via MMF94FX forcefield with gradient RMS of 0.0001 kcal/mol. The RMSDs of the best docked poses were 1.28 and 0.37 Å (for 1M17 and 3WZE; respectively) and the binding scores were -7.30 and −10.71 kcal/mol (for 1M17 and 3WZE; respectively). The ligands were then docked in the binding‏ ‏site using the alpha triangle placement method (Anighoro and Bajorath, 2016). The refinement was carried out using Forcefield and was‏ ‏scored using the Affinity ΔG scoring system.

Results of the docking revealed several interesting findings: most of the test derivatives have moderate to strong docking scores (except compound **14** gave weak docking score of 4.25 kcal/mol within VEGRF-2 protein). Notably, the methoxy-substituted derivative (compound **22**) showed higher docking score over its 4-chloro-congener (compound **29**), and by changing the S-substitution from being a benzyl methoxy group to an alkyl group (Compounds **17** and **19**), an observable decrease in docking score matching their measured *in vitro* activity against EGFR and VEGFR-2 enzymes, as shown Table 5.

**TABLE 5:** Docking simulations of best compounds within EGFR and VEGFR-2 active sites.

Compound	EGFR (PDB ID: 1M17)				VEGFR-2 (PDB ID: 3WZE)			
	S (Kcal/mol)	Binding interactions			S (Kcal/mol)	Binding interactions		
		Type	a.a. Residue	Length (Å)		Type	a.a. Residue	Length (Å)
14	-6.33	pi-H	Gly772	3.65	−4.25	pi-H	Cys1045	4.14
17	-5.92	H-acceptor	Met769	3.91	−6.82	H-acceptor	Lys868	3.30
		pi-cation	Lys721	4.23		pi-H	Asp1046	4.36
19	-6.84	pi-cation	Lys721	4.71	−6.28	H-pi	His1026	4.03
		pi-H	Val702	4.43		pi-H	Leu889	4.27
22	-6.88	H-donor	Arg817	3.42	−7.94	H-donor	Glu885	2.7
		pi-H	Lys721	4.63		H-acceptor	Lys868	3.15
29	-6.58	H-donor	Arg817	3.73	−6.28	H-donor	Glu885	2.7
						H-acceptor	Lys868	3.15
25	-6.19	pi-H	Val702	4.25	−6.15	pi-H	Cys1045	4.15
		pi-H	Gly772	4.19				
Erlotinib	−7.3	H-donor	Gln767	3.15				
		H-acceptor	Met769	2.7				
Sorafenib					−10.71	H-donor	Cys919	2.76
						H-donor	Glu885	2.7
						H-acceptor	Asp1046	2.83
						H-pi	Phe1047	3.76

**FIGURE 7:**
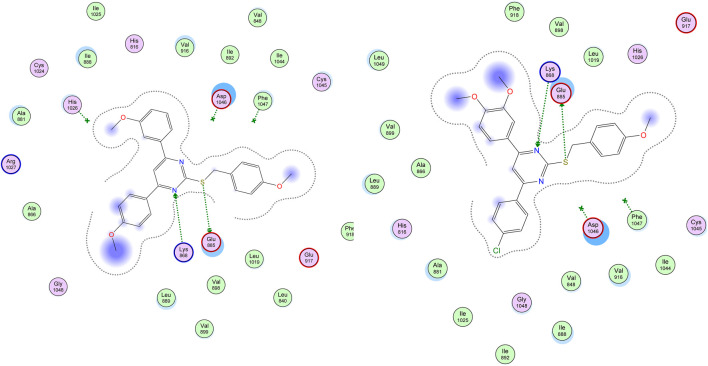
Docking of compounds **22** and **29** within the active site of VEGFR-2 (PDB ID: 3WZE) showing H-bonds (donor and acceptor) with crucial amino acid residues (Glu 885 and Lys 868).

Visual inspection of all docking poses obtained for these S-benzyloxy derivatives within VEGFR-2 proteins revealed a strong H-bonding interaction between the S-benzyloxy moiety and a crucial amino acid residue, GLU 885. This interaction brings the molecule into close contact with the Lys 868 amino acid residue, which stabilizes the molecule better within the VEGFR-2 active site, as shown in Figure 7.

**FIGURE 8:**
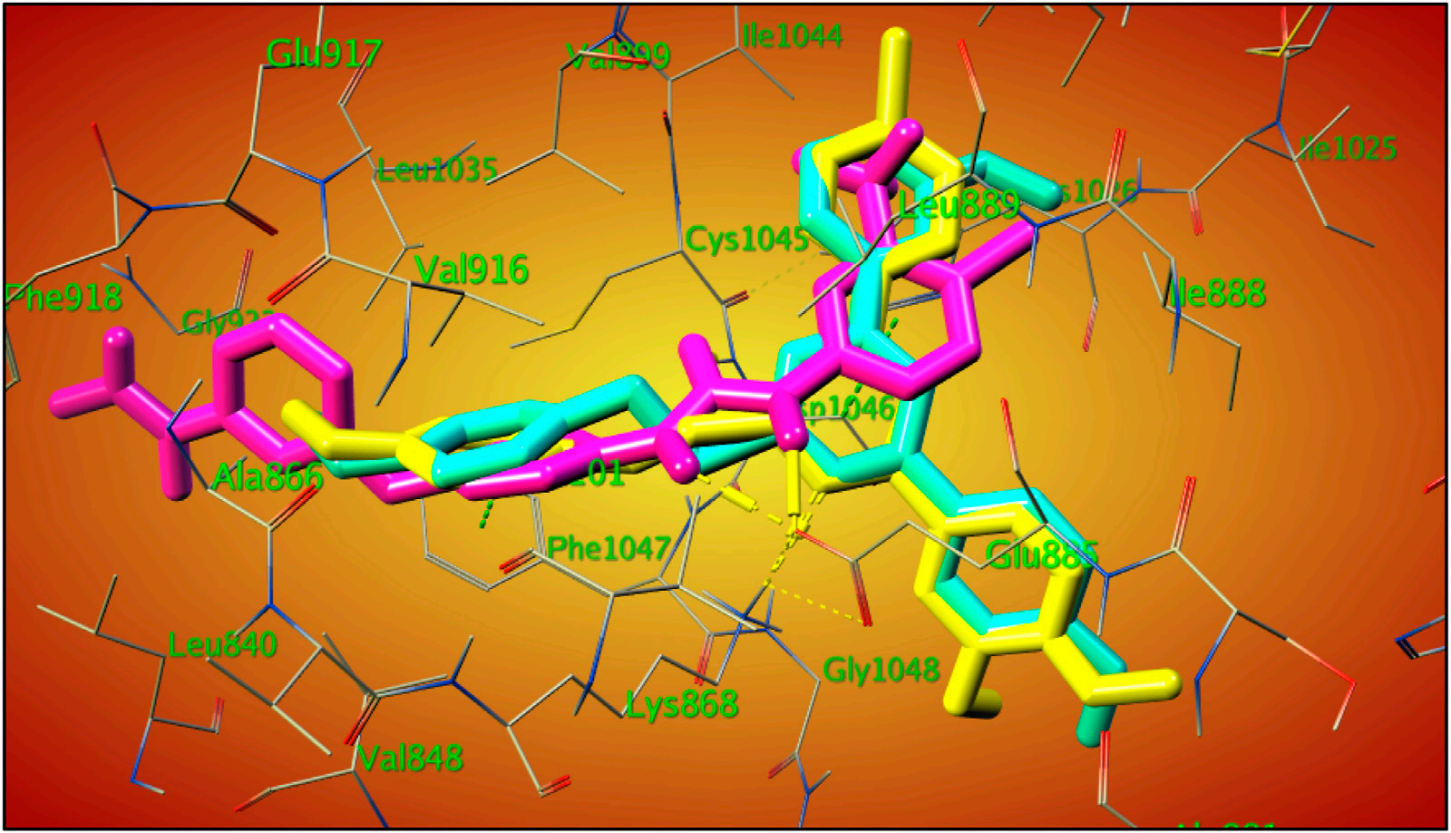
3D-overlay of compound **22** (cyan), **29** (yellow), **sorafenib** (purple) within VEGFR-2 active (PDB ID: 3WZE).

The overlay of compounds **22** and **29** with sorafenib showed the excellent overlay of the S-Benzyloxy moiety with the ureido-group of sorafenib at the same region of the VEGRF-2 active site, which is responsible for its interaction with the Glu 885 amino acid residue, as shown in Figure 8.

**FIGURE 9:**
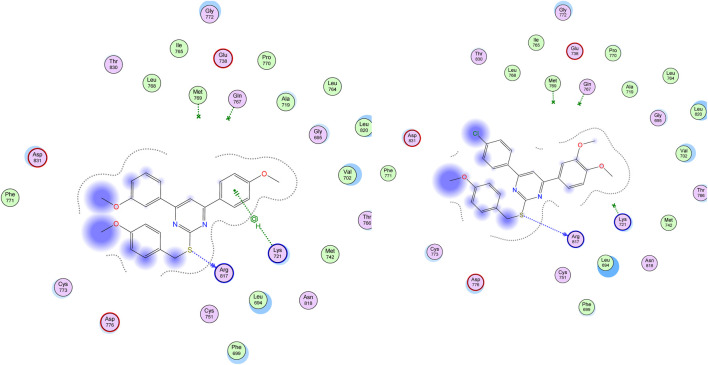
Binding Interactions of compounds 22 (left panel) and 95 (right panel) within EGFR protein (PDN ID: 1M17) showing H-bonding (as a blue-dotted arrow) and H-Pi interactions (as a green-dotted line).

On the other hand, visual inspection of docking poses of compound **22** within the EGFR active site revealed a common binding mode mediated through the S-benzyloxy moiety with Arg 817, which caused the whole molecule to move closer to Lys 721 and stabilized the whole molecule more efficiently than compound **29**, as shown in Figure 9.

Collectively, most of the test derivatives have comparable docking scores to co-crystallized ligands, Erlotinib and Sorafenib, against EGFR and VEGFR-2 proteins, and these scores were higher with S-benzyl methoxy derivatives than those of other congeners. Additionally, derivatives with a methoxy group at *meta*-position showed better interaction profiles than other derivatives.

### 2.5 ADMET study

Prediction of these ADMET properties helps in predicting the transport properties of the molecules through the membranes such as blood-brain barrier (BBB) and gastrointestinal tract (GI tract). If the compounds failed to obey two or more of these drug-like parameters, there would be a high possibility of their poor bioavailability (Martin, 2005). All test compounds were characterized by having 3 to 5 H-bond acceptor atoms, these centers helped in H-bond formation and therefore enhanced water solubility. Another important physicochemical parameter is lipophilicity or partition coefficient (iLogP); compounds having values less than −0.5 will have poor dissolution in lipids and will not be able to penetrate cell membranes. The prepared compounds were found to have iLogP values between 4.21–4.74 which indicated the probability of a good penetration through cell membranes and hence better bioavailability. Likewise, TPSA and MW affect the transportation of the molecules through the biological membranes. Generally, compounds with MW below 500 g/mol and TPSA less than 140 Ȧ2 could transfix through membranes easily, rapidly and subsequently will have less undesirable side effects. Moreover, assessment of the bioactivity score (F) of the new compounds using Swiss ADMET showed their substantial bioavailability (score above zero) and hence a higher probability of medicinal impact and biological activities in clinical trials.

#### 2.5.1 Theoretical lipophilicity calculations

Three different software programs (MOE, ChemDraw, and MolSoft) were used to estimate the theoretical lipophilicity (log *P*) of 6 derivatives (**14**, **19**, **22**, **26–28**) (Hassan et al., 2023). The calculated log *p* values of test compounds were in the range of 5.23–7.48. As shown in Table 6, the *S*-alkyl derivatives (**19** and **26**) showed the lowest *p* values. Moreover, changing substitution from isopropyl to allyl (derivative **27**) caused a marked decrease in log *p* values of two software estimates (MOE and MolSoft). In contrast, the estimate increased in the ChemDraw software. Additionally, the *S*-benzyl derivatives (**14**, **22**, and **28**) gave very high *p* values with the MolSoft and ChemDraw software compared to those obtained with the MOE program. To conclude, the lipophilicity prediction software gave contradicting results despite the structural similarity among the tested compounds and thus it is not reliable to have a clear idea about the differences found in the antiproliferative activities among test compounds. So, from such a perspective, we thought the experimental determination of lipophilicity would give information and a clear idea of the relationship between lipophilicity and antiproliferative activity.

**TABLE 6:** Calculated log *P* using the different computational software programs.

Cpd. No.	Calcd. Log *P*			Experimental lipophilicity		
	MOE	ChemDraw	MolSoft	R_Mo_	*b*	C_o_
14	7.03	7.41	7.48			
19	5.33	5.65	6.40	7.02	−0.074	91.32
22	6.40	6.6	6.80	6.24	−0.0717	87.7
26	5.98	6.21	6.56	6.45	−0.0713	90.64
27	5.76	6.24	6.47	5.79	−0.0669	86.52
28	7.04	7.29	7.01	5.32	−0.0637	87.18

#### 2.5.2 Experimental lipophilicity (log *P*_*O/W*_) determination using RP-TLC

The RP-TLC method was used to measure the lipophilicity parameters (R_Mo_, b, and C_o_) described in the experimental section. The mobile phase consisted of different proportions of water and organic modifier (MeOH). R_F_ and R_M_ values were obtained for each ratio of organic modifier/water system (Supplementary Table S3), and a linear regression analysis was achieved to obtain the lipophilicity chromatographic descriptors (R_Mo_, b, and C_o_, see Table 6). Relative lipophilicity (R_Mo_) describes the partitioning of the solute between pure water and nonpolar stationary phase. In contrast, the second lipophilicity parameter (C_o_) represents the concentration of organic modifier in the mobile phase in which the solute is equally distributed between two phases (i.e., R_M_ = 0, and R_f_ = 0.5). Also, C_o_ is known to be widely used in QSAR analysis as it embraces both the specific hydrophobic surface area of the solute and R_Mo_. Also, correlating values of R_Mo_ with that of C_o_ were significantly high in all proportions of the methanol/water mobile phase (r ≈ −0.9915). Finally, the slope of the regression line (*b*) was used as a descriptor of the specific hydrophobic surface area of the compound. In a series of structurally related compounds, *b* is linearly correlated with R_Mo,_ resulting in a linear relationship that is the basic feature of the chromatographic determination of lipophilicity. As seen from Table 6, slope (*b*) correlated efficiently with R_Mo_ when changing substituents from simple alkyl to allyl and benzyl. A significant correlation exists between R_Mo_ and slope (*b*) with r ≈ −0.9985 in MeOH/H_2_O, which suggests a similar chromatographic retention mechanism for this congeneric series of compounds. Also, correlating values of R_Mo_ with that of C_o_ were significantly high in all proportions of the MeOH/H_2_O mobile phase (r ≈ −0.9915). Moreover, substituents affected tested compounds’ measured lipophilicity parameter (RMo) values. Higher R_Mo_ values were found with lipophilic derivatives containing the benzyl group (**14**, **22**, and **28**), while lower R_Mo_ values were found with derivatives containing alkyl or allyl groups (**19**, **26**, and **27**). An interesting contradiction was found with experimental lipophilicity value of **28** and its congener **14**, where compound **28** was found more lipophilic than its congener **14** even though it contains two methoxy groups and according to ChemDraw^®^ and MolSoft^®^ programs, derivative **14** should be more lipophilic while the MOE^®^ program expects values matching our findings. A comparison of results from NCI-60 antiproliferative activity, *in vitro* assays, apoptosis markers and experimentally measured lipophilicity revealed that *S*-benzylated derivatives (**14**, **19**, and **28**), which have the highest lipophilicity showed lower antiproliferative activity compared to *S*-alkyl (**26**) and *S*-allyl congeners (**27**). Also, *S*-allyl derivatives were nearly equipotent to *S*-alkyl congeners as antiproliferative agents but quite different in terms of lipophilicity. Taking all these findings about these new pyrimidines revealed a common observation, that such a class of compounds performs better biological activities within all tested biological systems when having low lipophilicity characters.

#### 2.5.3 ADME-calculations using Swiss-ADME website

Pharmacokinetics’ prediction is commonly used as of late in drug discovery of new leads or new modifications leading to better therapeutics (Wu et al., 2020). Calculated physicochemical parameters of test molecules (**14**, **19**, **22**, and **26–28**) were listed in Supplementary Table S5 (Supplementary Data Sheet 1). The Lipinski rule of five (RO5) instates that compounds with MW below 500 will penetrate easily and rapidly through biological membranes. Additionally, the presence of acceptable values for HBA, HBD, and rotatable bonds within molecules indicate a high possibility of their efficient interaction with biological targets and water solubility (Ugbe et al., 2023). Additionally, the calculated TPSA was found below 140 Å^2^ indicating the feasibility of these compounds to pass the blood-brain barrier (BBB). Finally, the Abbott bioavailability score (F) was found above zero for all test compounds. Collectively, the fulfillment of all or most of Lipinski’s parameters indicates the high probability of a compound in being a drug-candidate.

### 2.6 Structure activity relationship (SAR) analysis


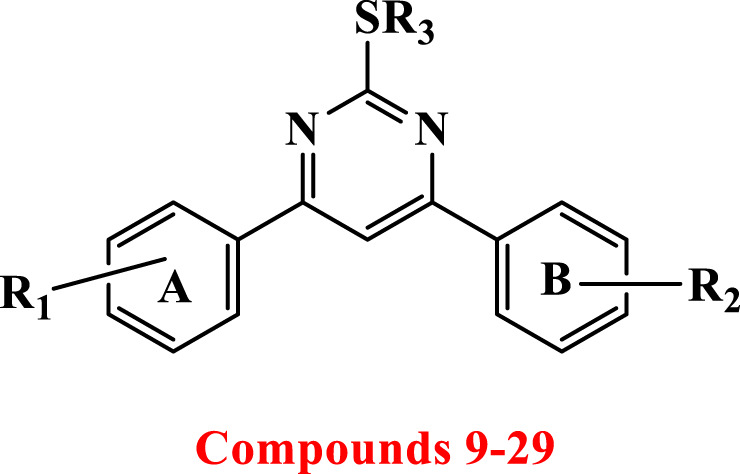

1. For antiproliferative efficacy, the substitution type on the sulfur atom (R_3_) at the second position of the di-aryl pyrimidine moiety is crucial, with activity ascending in the following sequence: 4-OMe-benzyl > Ethyl > n-Propyl > Allyl > Benzyl > Methyl.

The 4-OMe-benzyl group enhances action against EGFR by forming hydrogen bonds with critical amino acids Arg 817 and Lys 721. In VEGFR-2, hydrogen bonds between Glu 885 and Lys 868 enhance binding to receptor sites, hence increasing activity.

2. The replacement of the di-aryl pyrimidine group at the 4 and/or 6-positions is essential for activity and is another area of interest.

The data indicate that the 4-position substitution markedly influences the antiproliferative activity of these compounds, with the most pronounced activity observed in compounds containing 4-Cl phenyl or 4-OMe phenyl groups. Conversely, at the 6-position, both 3-OMe phenyl and 3,4-di-OMe phenyl are acceptable for antiproliferative efficacy, while 4-OMe phenyl is not preferred.

## 3 Conclusion

This study explores the synthesis of pyrimidine heterocycles **9–29**. The exact structure was determined and confirmed by NMR, HRMS, and X-ray diffraction investigation. Compounds **9–29** were evaluated as dual inhibitors of EGFR and VEGFR-2 in order to develop a scaffold capable of stopping cell growth. The findings indicated that compounds **22** and **29** are potential apoptotic antiproliferative agents that inhibit EGFR and VEGFR-2. Molecular docking studies have clearly shown how compounds **22** and **29** bind to the active sites of EGFR and VEGFR-2. This comprehensive examination is essential for comprehending their mechanism of action as antiproliferative agents. Moreover, the in-depth study of these hybrids’ absorption, distribution, metabolism, and excretion (ADME) features shows how useful they could be as therapeutic agents.

Conversely, additional structural modifications may be necessary to effectively obtain more potent lead molecules for the development of future cancer therapeutics.

## 4 Experimental

### 4.1 Chemistry

**General details:** See Supplementary Material A1.

#### 4.1.1 General procedures for the synthesis of compounds 9–29

Generally, stirring pyrimidine-2(1*H*)-thione/2-thiol intermediates **6–8** (1.5 mmol) with different alkyl, aralkyl, or allyl halides (1.5 mmol) in dry acetonitrile (10–15 mL) and triethyl amine (TEA) (1.7 mmol) at 70–80°C for 10–12 h yielded target 2-substitutedthio-4,6-diaryl pyrimidines **9–29**. Using n-hexane: EtOAc as the primary elution solvent (in varying amounts from 2% to 10% EtOAc), we purified the new compounds **9–29** using Biotage^®^ Select ELSD, resulting in pure compounds with yields between 53% and 91%.

##### 4.1.1.1 4-(4-Chlorophenyl)-6-(4-methoxyphenyl)-2-(methylthio)pyrimidine (9)

Yield 82%; m. p. 220–221°C; ^1^H NMR (300 MHz, CDCl_3_) δ (ppm) = 8.13 (overlap d.d., 4H, Ar-H), 7.69 (s, 1H, pyrimidine–C_5_-H), 7.5 (d., 2H, Ar-H, *J* = 9 Hz), 7.04 (d., 2H, Ar-H, *J* = 9 Hz), 3.92 (s, 3H, OCH_3_), 2.73 (s, 3H, SCH_3_); ^13^C NMR (75 MHz, CDCl_3_) δ (ppm) = 172.7, 164.3, 163.14, 162.17, 136.9, 135.5, 129.1, 128.8, 128.5, 114.3, 106.7, 55.5, 14.4; HRMS (ESI+) calcd. For C_18_H_16_ClN_2_OS (M + H)^+^ 343.06664; found 343.06726.

##### 4.1.1.2 4-(4-Chlorophenyl)-2-(ethylthio)-6-(4-methoxyphenyl)pyrimidine (10)

Yield 77%; m. p. 230–231°C; ^1^H NMR (300 MHz, CDCl_3_) δ (ppm) = 8.1 (overlap d.d., 4H, Ar-H), 7.63 (s, 1H, pyrimidine–C_5_-H), 7.48 (d., 2H, Ar-H, *J* = 9 Hz), 7.01 (d., 2H, Ar-H, *J* = 9 Hz), 3.89 (s, 3H, OCH_3_), 3.3 (q, 2H, SCH_2_CH_3_, *J* = 7.2 Hz), 1.51 (t, 3H, SCH_2_CH_3_, *J* = 7.2 Hz); ^13^C NMR (75 MHz, CDCl_3_) δ (ppm) = 172.4, 164.3, 163.1, 162.1, 136.9, 135.5, 129, 128.8, 128.5, 114.2, 106.6, 55.4, 25.5, 14.7; LRMS (ESI+) m/z (%) 359 (M + H+2, 39), 357 (M + H, 100).

##### 4.1.1.3 4-(4-Chlorophenyl)-6-(4-methoxyphenyl)-2-(propylthio)pyrimidine (11)

Yield 85%; m. p. 228–230°C; ^1^H NMR (300 MHz, CDCl_3_) δ (ppm) = 8.11 (overlap d.d., 4H, Ar-H), 7.67 (s, 1H, pyrimidine–C_5_-H), 7.49 (d., 2H, Ar-H, *J* = 9 Hz), 7.03 (d., 2H, Ar-H, *J* = 9 Hz), 3.91 (s, 3H, OCH_3_), 3.29 (t, 2H, SCH_2_CH_2_CH_3_, *J* = 7.2 Hz), 1.9 (m., 2H, SCH_2_CH_2_CH_3_), 1.14 (t, 3H, SCH_2_CH_2_CH_3_, *J* = 7.2 Hz); ^13^C NMR (75 MHz, CDCl_3_) δ (ppm) = 172.5, 164.3, 163.2, 162.1, 136.9, 135.5, 129.1, 128.8, 128.5, 114.3, 106.7, 55.5, 33.1, 22.8, 13.6; LRMS (ESI+) m/z (%) 373 (M + H+2, 38), 371 (M + H, 100).

##### 4.1.1.4 4-(4-Chlorophenyl)-2-(isopropylthio)-6-(4-methoxyphenyl)pyrimidine (12)

Yield 63%; m. p. 198–201°C; ^1^H NMR (300 MHz, CDCl_3_) δ (ppm) = 8.1 (overlap d.d., 4H, Ar-H), 7.65 (s, 1H, pyrimidine–C_5_-H), 7.48 (d., 2H, Ar-H, *J* = 9 Hz), 7.02 (d., 2H, Ar-H, *J* = 9 Hz), 4.14 (m, 1H, SCH(CH_3_)_2_), 3.9 (s, 3H, OCH_3_), 1.55 (d, 6H, SCH(CH_3_)_2_, *J* = 6.6 Hz); ^13^C NMR (75 MHz, CDCl_3_) δ (ppm) = 172.6, 164.3, 163.1, 162.1, 136.9, 135.5, 129.1, 128.8, 128.4, 114.2, 106.5, 55.4, 35.9, 22.9; LRMS (ESI+) m/z (%) 373 (M + H+2, 38), 371 (M + H, 100).

##### 4.1.1.5 2-(Allylthio)-4-(4-chlorophenyl)-6-(4-methoxyphenyl)pyrimidine (13)

Yield 52%; m. p. 245–247°C; ^1^H NMR (300 MHz, CDCl_3_) δ (ppm) = 8.09 (overlap d.d., 4H, Ar-H), 7.66 (s, 1H, pyrimidine–C_5_-H), 7.47 (d., 2H, Ar-H, *J* = 9 Hz), 7.03 (d., 2H, Ar-H, *J* = 9 Hz), 6.11 (m, 1H, SCH_2_CH = CH_2_), 5.41 (d.d., 1H, *J* = 1.2 and 1.5 Hz, SCH_2_CH = CH_2_), 5.18 (d.d., 1H, *J* = 1.5 and 1.5 Hz, SCH_2_CH = CH_2_), 3.99 (overlap d.d., 2H, SCH_2_CH = CH_2_), 3.89 (s, 3H, OCH_3_), 1.55 (d, 6H, SCH(CH_3_)_2_, *J* = 6.6 Hz); ^13^C NMR (75 MHz, CDCl_3_) δ (ppm) = 171.7, 164.4, 163.2, 162.2, 137, 135.4, 134, 129.1, 128.8, 128.5, 117.5, 114.3, 106.8, 55.4, 34; LRMS (ESI+) m/z (%) 371 (M + H+2, 38), 369 (M + H, 100).

##### 4.1.1.6 2-(Benzylthio)-4-(4-chlorophenyl)-6-(4-methoxyphenyl)pyrimidine (14)

Yield 87%; m. p. 288–290°C; ^1^H NMR (300 MHz, CDCl_3_) δ (ppm) = 8.09 (overlap d.d., 4H, Ar-H), 7.69 (s, 1H, pyrimidine–C_5_-H), 7.49 (m., 4H, Ar-H), 7.31 (m., 3H, Ar-H), 7.03 (d, 2H, Ar-H, *J* = 9 Hz), 4.6 (s, 2H, SCH_2_-), 3.91 (s, 3H, OCH_3_); ^13^C NMR (75 MHz, CDCl_3_) δ (ppm) = 171.9, 164.4, 163.3, 162.2, 138, 137, 135.4, 129.1, 128.9, 128.5, 127.1, 114.3, 107.1, 55.5, 35.5; HRMS (ESI+) calcd. For C_24_H_20_ClN_2_OS (M + H)^+^ 419.09794; found 419.09800.

##### 4.1.1.7 2-(4-Methoxybenzylthio)-4-(4-chlorophenyl)-6-(4-methoxyphenyl)pyrimidine (15)

Yield 73%; m. p. 278–280°C; ^1^H NMR (300 MHz, CDCl_3_) δ (ppm) = 8.12 (overlap d.d., 4H, Ar-H), 7.7 (s, 1H, pyrimidine–C_5_-H), 7.5 (d., 2H, Ar-H, *J* = 8.7 Hz), 7.42 (d, 2H, Ar-H, *J* = 8.7 Hz), 7.04 (d, 2H, Ar-H, *J* = 9 Hz), 6.86 (d., 2H, Ar-H, *J* = 9 Hz), 4.55 (s, 2H, SCH_2_-), 3.92 (s, 3H, OCH_3_), 3.81 (s, 3H, OCH_3_); ^13^C NMR (75 MHz, CDCl_3_) δ (ppm) = 172.1, 164.4, 163.3, 162.2, 158.8, 137, 135.5, 130.1, 129.9, 129.1, 128.9, 128.5, 114.3, 113.9, 107, 55.5, 55.3, 35; HRMS (ESI+) calcd. For C_25_H_21_ClN_2_O_2_S (M + H)^+^ 449.10850; found 449.10841.

##### 4.1.1.8 6-(3-Methoxyphenyl)-4-(4-methoxyphenyl)-2-(methylthio)pyrimidine (16)

Yield 72%; m. p. 213–215°C; ^1^H NMR (300 MHz, CDCl_3_) δ (ppm) = 8.14 (d, 2H, Ar-H, *J* = 9 Hz), 7.72 (m, 3H, Ar-H and pyrimidine–C_5_-H), 7.43 (d.d., 2H, Ar-H, *J* = 7.8 and 8.1 Hz), 7.06 (m, 3H, Ar-H), 3.91 (overlap singlets, 6H, 2 OCH_3_) 2.73 (s, 3H, SCH_3_); ^13^C NMR (75 MHz, CDCl_3_) δ (ppm) = 172.5, 164.2, 164.1, 162.1, 160.1, 138.6, 129.8, 129.3, 128.8, 119.6, 116.5, 114.2, 112.6, 107.2, 55.4, 14.4; HRMS (ESI+) calcd. For C_19_H_19_N_2_O_2_S (M + H)^+^ 339.11618; found 339.11696.

##### 4.1.1.9 2-(Ethylthio)-6-(3-methoxyphenyl)-4-(4-methoxyphenyl)pyrimidine (17)

Yield 81%; m. p. 221–222°C; ^1^H NMR (300 MHz, CDCl_3_) δ (ppm) = 8.13 (d.d., 2H, Ar-H, *J* = 2.1 and 6.9 Hz), 7.72 (m, 3H, Ar-H and pyrimidine–C_5_-H), 7.43 (d.d., 2H, Ar-H, *J* = 7.8 and 8.1 Hz), 7.05 (m, 3H, Ar-H), 3.91 (overlap singlets, 6H, 2 OCH_3_) 3.32 (q, 2H, SCH_2_CH_3_, *J* = 7.2 Hz), 1.53 (t, 3H, SCH_2_CH_3_, *J* = 7.2 Hz); ^13^C NMR (75 MHz, CDCl_3_) δ (ppm) = 172.2, 164.2, 164.1, 162, 160.1, 138.6, 129.8, 129.3, 128.8, 119.5, 116.5, 114.2, 112.5, 107.1, 55.4, 25.5, 14.7; HRMS (ESI+) calcd. For C_20_H_21_N_2_O_2_S (M + H)^+^ 353.13183; found 353.13198.

##### 4.1.1.10 6-(3-Methoxyphenyl)-4-(4-methoxyphenyl)-2-(propylthio)pyrimidine (18)

Yield 67%; m. p. 218–220°C; ^1^H NMR (300 MHz, CDCl_3_) δ (ppm) = 8.13 (d., 2H, Ar-H, *J* = 9 Hz), 7.73 (m, 3H, Ar-H and pyrimidine–C_5_-H), 7.42 (d.d., 2H, Ar-H, *J* = 7.8 and 8.1 Hz), 7.06 (m, 3H, Ar-H), 3.91 (overlap singlets, 6H, 2 OCH_3_) 3.3 (t, 2H, SCH_2_CH_2_CH_3_, *J* = 7.2 Hz), 1.91 (m., 2H, SCH_2_CH_2_CH_3_), 1.53 (t, 3H, SCH_2_CH_2_CH_3_, *J* = 7.5 Hz); ^13^C NMR (75 MHz, CDCl_3_) δ (ppm) = 172.3, 164.2, 164.1, 162, 160.1, 138.6, 129.8, 129.3, 128.8, 119.5, 116.6, 114.2, 112.4, 107.1, 55.4, 33.1, 22.9, 13.7; HRMS (ESI+) calcd. For C_21_H_23_N_2_O_2_S (M + H)^+^ 367.14748; found 367.14773.

##### 4.1.1.11 2-(Isopropylthio)-6-(3-methoxyphenyl)-4-(4-methoxyphenyl)pyrimidine (19)

Yield 65%; m. p. 235–237°C; ^1^H NMR (300 MHz, CDCl_3_) δ (ppm) = 8.14 (d., 2H, Ar-H, J = 9 Hz), 7.73 (m, 3H, Ar-H and pyrimidine–C5-H), 7.43 (d.d., 2H, Ar-H, J = 7.8 and 8.1 Hz), 7.02 (m, 3H, Ar-H), 4.18 (m., 1H, SCH(CH_3_)_2_), 3.91 (overlap singlets, 6H, 2 OCH_3_), 1.55 (d, 6H, SCH(CH_3_)_2_, *J* = 6.9 Hz); ^13^C NMR (75 MHz, CDCl3) δ (ppm) = 172.4, 164.2, 162, 160.1, 138.6, 129.8, 129.3, 128.8, 119.5, 116.6, 114.2, 112.5, 107, 55.4, 35.9, 23; HRMS (ESI+) calcd. For C_21_H_23_N_2_O_2_S (M + H)^+^ 367.14748; found 367.14763.

##### 4.1.1.12 2-(Allylthio)-6-(3-methoxyphenyl)-4-(4-methoxyphenyl)pyrimidine (20)

Yield 54%; m. p. 258–260°C; ^1^H NMR (300 MHz, CDCl_3_) δ (ppm) = 8.14 (d., 2H, Ar-H, J = 9 Hz), 7.71 (m, 3H, Ar-H and pyrimidine–C_5_-H), 7.44 (d.d., 2H, Ar-H, J = 7.8 and 8.1 Hz), 7.07 (m, 3H, Ar-H), 6.12 (m, 1H, SCH_2_CH = CH_2_), 5.41 (d.d., 1H, SCH_2_CH = CH_2_, *J* = 1.2 and 16.8 Hz), 5.18 (d.d., 1H, SCH_2_CH = CH_2_, *J* = 1.5 and 17.1 Hz), 4.01 (d., 2H, SCH_2_CH = CH_2_, *J* = 6.9 Hz) 3.92 (overlap singlets, 6H, 2 OCH_3_), 1.55 (d, 6H, SCH(CH_3_)_2_, *J* = 6.9 Hz); ^13^C NMR (75 MHz, CDCl_3_) δ (ppm) = 171.5, 164.3, 164.3, 162.1, 160.1, 138.5, 134.2, 129.8, 129.2, 128.8, 119.6, 117.4, 116.6, 114.2, 112.5, 107.4, 55.5, 34; HRMS (ESI+) calcd. For C_21_H_21_N_2_O_2_S (M + H)^+^ 365.13183; found 365.13191.

##### 4.1.1.13 2-(Benzylthio)-6-(3-methoxyphenyl)-4-(4-methoxyphenyl)pyrimidine (21)

Yield 87%; m. p. 245–247°C; ^1^H NMR (300 MHz, CDCl_3_) δ (ppm) = 8.13 (d., 2H, Ar-H, J = 9 Hz), 7.72 (m, 2H, Ar-H and pyrimidine–C_5_-H), 7.53 (d, 2H, Ar-H, *J* = 6.9 Hz), 7.44 (d., 1H, Ar-H, *J* = 8.1 Hz), 7.32 (m, 4H, Ar-H), 7.04 (m, 3H, Ar-H), 4.62 (s., 2H, SCH_2_C_6_H_5_), 3.90 (overlap singlets, 6H, 2 OCH_3_); ^13^C NMR (75 MHz, CDCl_3_) δ (ppm) = 171.7, 164.4, 164.3, 162.1, 160.1, 138.5, 129.8, 128.9, 128.8, 128.5, 127.1, 119.6, 116.7, 114.2, 112.5, 107.5, 55.5, 35.5; LRMS (ESI+) m/z (%) 416 (M + H+2, 30), 415 (M + H, 100).

##### 4.1.1.14 2-(4-Methoxybenzylthio)-6-(3-methoxyphenyl)-4-(4-methoxyphenyl)pyrimidine (22)

Yield 77%; m. p. 265–267°C; ^1^H NMR (300 MHz, CDCl_3_) δ (ppm) = 8.14 (d., 2H, Ar-H, *J* = 9 Hz), 7.72 (m, 3H, Ar-H and pyrimidine–C_5_-H), 7.44 (m., 3H, Ar-H), 7.04 (m, 3H, Ar-H), 6.86 (d., 2H, Ar-H, *J* = 8.7 Hz), 4.57 (s., 2H, SCH_2_C_6_H_5_), 3.91(overlap singlets, 6H, 2 OCH_3_), 3.81 (s, 3H, SCH_2_C_6_H_4_OCH_3_); ^13^C NMR (75 MHz, CDCl_3_) δ (ppm) = 171.9, 164.3, 164.2, 162.1, 160.1, 158.7, 138.6, 129.8, 128.9, 119.6, 116.7, 114.3, 113.9, 112.5, 107.5, 55.5, 34.9; HRMS (ESI+) calcd. For C_26_H_25_N_2_O_3_S (M + H)^+^ 445.15804; found 445.15721.

##### 4.1.1.15 4-(4-Chlorophenyl)-6-(3,4-dimethoxyphenyl)-2-(methylthio)pyrimidine (23)

Yield 91%; m. p. 228–229°C; ^1^H NMR (300 MHz, CDCl_3_) δ (ppm) = 8.13 (d.d., 2H, Ar-H, *J* = 1.8 and 8.7 Hz), 7.8 (d., 1H, Ar-H, *J* = 1.8 Hz), 7.75 (d.d., 1H, Ar-H, *J* = 2.1 and 8.4 Hz), 7.69 (s, 1H, pyrimidine–C_5_-H), 7.52 (d.d., 2H, Ar-H, J = 1.8 and 8.7 Hz), 7.01 (d., 1H, Ar-H, *J* = 8.4 Hz), 4.01(2 overlap singlets, 6H, 2 OCH_3_), 2.73 (s, 3H, SCH_3_); ^13^C NMR (75 MHz, CDCl_3_) δ (ppm) = 171.9, 164.3, 163.1, 151.8, 149.3, 137, 135.5, 129.4, 129.1, 128.5, 120.4, 110.9, 110.1, 106.9, 56.1, 14.4; HRMS (ESI+) calcd. For C_19_H_18_N_2_O_2_SCl (M + H)^+^ 373.07720; found 373.07750.

##### 4.1.1.16 4-(4-Chlorophenyl)-2-(ethylthio)-6-(3,4-dimethoxyphenyl)pyrimidine (24)

Yield 87%; m. p. 229–231°C; ^1^H NMR (300 MHz, CDCl_3_) δ (ppm) = 8.1 (d.d., 2H, Ar-H, *J* = 2.1 and 8.7 Hz), 7.81 (d., 1H, Ar-H, *J* = 2.1 Hz), 7.72 (d.d., 1H, Ar-H, *J* = 2.1 and 8.7 Hz), 7.68 (s, 1H, pyrimidine–C_5_-H), 7.5 (d.d., 2H, Ar-H, J = 2.1 and 8.7 Hz), 6.99 (d., 1H, Ar-H, *J* = 8.4 Hz), 4.02(2 overlap singlets, 6H, 2 OCH_3_), 3.32 (apparent d., 2H, SCH_2_CH_3_, *J* = 7.2 Hz), 1.54 (apparent q., 3H, SCH_2_CH_3_, *J* = 7.2 Hz); ^13^C NMR (75 MHz, CDCl_3_) δ (ppm) = 172.4, 164.4, 163.2, 151.7, 149.3, 137, 135.5, 129.5, 129.1, 128.5, 120.4, 110.9, 110, 106.9, 56, 25.5, 14.7; HRMS (ESI+) calcd. For C_20_H_20_N_2_O_2_SCl (M + H)^+^ 387.09285; found 387.09215.

##### 4.1.1.17 4-(4-Chlorophenyl)-6-(3,4-dimethoxyphenyl)-2-(propylthio)pyrimidine (25)

Yield 79%; m. p. 217–219°C; ^1^H NMR (300 MHz, CDCl_3_) δ (ppm) = 8.09 (d., 2H, Ar-H, *J* = 9 Hz), 7.8 (d., 1H, Ar-H, *J* = 2.1 Hz), 7.7 (d.d., 1H, Ar-H, *J* = 2.1 and 8.4 Hz), 7.68 (s, 1H, pyrimidine–C_5_-H), 7.49 (d.d., 2H, Ar-H, J = 2.1 and 8.9 Hz), 6.98 (d., 1H, Ar-H, *J* = 8.4 Hz), 3.99(2 overlap singlets, 6H, 2 OCH_3_), 3.28 (apparent q., 2H, SCH_2_CH_2_CH_3_, *J* = 7.2 Hz), 1.91 (m., 2H, SCH_2_CH_2_CH_3_), 1.35 (t., 3H, SCH_2_CH_2_CH_3_, *J* = 7.2 Hz); ^13^C NMR (75 MHz, CDCl_3_) δ (ppm) = 172.5, 164.3, 163.2, 151.7, 149.3, 137, 135.5, 129.4, 129.1, 128.5, 120.4, 110.9, 110, 106.9, 56, 33.1, 22.9, 13.7; HRMS (ESI+) calcd. For C_21_H_22_N_2_O_2_SCl (M + H)^+^ 401.10850; found 401.10746.

##### 4.1.1.18 4-(4-Chlorophenyl)-2-(isopropylthio)-6-(3,4-dimethoxyphenyl)pyrimidine (26)

Yield 81%; m. p. 233–253°C; ^1^H NMR (300 MHz, CDCl_3_) δ (ppm) = 8.09 (d., 2H, Ar-H, *J* = 9 Hz), 7.8 (d., 1H, Ar-H, *J* = 2.1 Hz), 7.7 (d.d., 1H, Ar-H, *J* = 2.1 and 8.4 Hz), 7.68 (s, 1H, pyrimidine–C_5_-H), 7.49 (d.d., 2H, Ar-H, J = 2.1 and 8.9 Hz), 6.98 (d., 1H, Ar-H, *J* = 8.4 Hz), 4.13 (m., 1H, SCH(CH_3_)_2_), 4.01 (2 overlap singlets, 6H, 2 OCH_3_), 1.55 (d., 6H, SCH(CH_3_)_2_, *J* = 6.9 Hz); ^13^C NMR (75 MHz, CDCl_3_) δ (ppm) = 172.6, 164.3, 163.2, 151.7, 149.3, 136.9, 135.5, 129.5, 129.1, 128.5, 120.3, 110.9, 110, 106.7, 56, 36, 22.9; HRMS (ESI+) calcd. For C_21_H_22_N_2_O_2_SCl (M + H)^+^ 401.10850; found 401.10739.

##### 4.1.1.19 2-(Allylthio)-4-(4-chlorophenyl)-6-(3,4-dimethoxyphenyl)pyrimidine (27)

Yield 57%; m. p. 244–246°C; ^1^H NMR (300 MHz, CDCl_3_) δ (ppm) = 8.08 (d., 2H, Ar-H, *J* = 8.7 Hz), 7.87 (d., 1H, Ar-H, *J* = 1.8 Hz), 7.7 (d.d., 1H, Ar-H, *J* = 1.8 and 8.7 Hz), 7.68 (s, 1H, pyrimidine–C_5_-H), 7.49 (d., 2H, Ar-H, *J* = 8.9 Hz), 6.98 (d., 1H, Ar-H, *J* = 8.7 Hz), 6.13 (m., 1H, SCH_2_CH = CH_2_), 5.4 (d.d., 1H, SCH_2_CH = CH_2_, *J* = 1.5 and 16.8 Hz), 5.17 (d.d., 1H, SCH_2_CH = CH_2_, *J* = 1.5 and 9.9 Hz), 4.01 (2 overlap singlets, 6H, 2 OCH_3_), 3.82 (m, 2H, SCH_2_CH = CH_2_); ^13^C NMR (75 MHz, CDCl_3_) δ (ppm) = 171.7, 164.4, 163.3, 151.8, 149.3, 137.1, 135.4, 134, 129.5, 129.4, 129.3, 129.1, 128.9, 128.5, 120.4, 118.8, 117.5, 110.9, 110, 107.1, 56.1, 33.9; HRMS (ESI+) calcd. For C_21_H_20_N_2_O_2_SCl (M + H)^+^ 399.09285; found 399.09199.

##### 4.1.1.20 2-(Benzylthio)-4-(4-chlorophenyl)-6-(3,4-dimethoxyphenyl)pyrimidine (28)

Yield 87%; m. p. 239–241°C; ^1^H NMR (300 MHz, CDCl_3_) δ (ppm) = 8.09 (d., 2H, Ar-H, *J* = 8.7 Hz), 7.7 (m, 3H, Ar-H and pyrimidine–C_5_-H), 7.51 (m., 4H, Ar-H), 7.31 (m., 3H, Ar-H), 6.99 (d., 1H, Ar-H, J = 8.4 Hz), 4.61 (s., 2H, SCH_2_C_6_H_5_), 3.99 (2 overlap singlets, 6H, 2 OCH_3_); ^13^C NMR (75 MHz, CDCl_3_) δ (ppm) = 171.9, 164.5, 163.3, 151.8, 149.3, 138, 137.1, 135.4, 129.4, 129.1, 128.8, 128.5, 127.1, 120.4, 110.9, 110, 107.2, 56.1, 35.5; HRMS (ESI+) calcd. For C_25_H_22_N_2_O_2_SCl (M + H)^+^ 449.10850; found 449.10846.

##### 4.1.1.21 2-(4-Methoxybenzylthio)-4-(4-chlorophenyl)-6-(3,4-dimethoxyphenyl)pyrimidine (29)

Yield 88%; m. p. 279–281°C; ^1^H NMR (300 MHz, CDCl_3_) δ (ppm) = 8.1 (d., 2H, Ar-H, *J* = 8.7 Hz), 7.78 (d., 1H, Ar-H, J = 2.1 Hz), 7.73 (d., 1H, Ar-H, J = 2.1 Hz), 7.7 (s, 1H, pyrimidine–C_5_-H), 7.5 (d.d., 2H, Ar-H, J = 2.1 and 6.9 Hz), 7.43 (d.d., 2H, Ar-H, J = 2.1 and 6.9 Hz), 7 (d., 1H, Ar-H, J = 8.4 Hz), 6.86 (d., 2H, Ar-H, J = 9.0 Hz), 4.56 (s., 2H, SCH_2_C_6_H_4-_), 3.99 (2 overlap singlets, 6H, 2 OCH_3_), 3.81 (s., 3H, SCH_2_C_6_H_4_OCH_3_); ^13^C NMR (75 MHz, CDCl_3_) δ (ppm) = 172.1, 164.5, 163.3, 158.8, 151.8, 149.4, 137.1, 135.4, 129.9, 129.8, 129.4, 129.1, 128.5, 120.4, 113.9, 110.9, 110.1, 107.2, 56.1, 55.3, 35; HRMS (ESI+) calcd. For C_26_H_24_N_2_O_3_SCl (M + H)^+^ 479.11907; found 479.12000.

#### 4.1.2 X-ray single crystal diffraction of compound 14

The experiment was performed on colorless needles of compound (**14**) at 200 K on a Bruker Kappa APEX II diffractometer and the data was corrected for absorption using intensity measurements (SADABS). Crystal data: C_24_ H_19_ Cl N_2_ O S, MW = 478.99, Orthorhombic; *a* = 6.193 (2) Å, *b* = 14.250 (5) Å, *c* = 23.313 (6) Å; a = 90°, β = 90°, y = 90o; *V* = 2057.38 A^3^; space group *P*2_1_2_1_2_1_, *Z:* 4 and *Z':* 0; Dc = 1.352 g cm^-3^. The structure was solved by direct methods and refined using the Flack parameter measurement (Watkin and Cooper, 2016). Geometric data of compound **14** were listed in Supplementary Tables S7–S12.

### 4.2 Biology

#### 4.2.1 in vitro NCI antiproliferative screening

The National Cancer Institute’s Developmental Therapeutic Program (www.dtp.nci.nih.gov) evaluated compounds **9–29** (excluding **21** and **22**) against 60 cancer cell lines from nine categories (leukemia, lung, colon, CNS, melanoma, ovarian, renal, prostate, and breast cancer) at a single dose (10 μM) (El-Sherief et al., 2018). See Supplementary Material A1 for more details.

#### 4.2.2 Cell viability assay

This experiment examines the impact of the newly developed compounds **9–29** on normal cell lines to assess their safety level. The viability of **9–29** was assessed using the MCF-10 A cell line, a normal human mammary gland epithelial cell line. Following 4 days of incubation on MCF-10 A cells with each examined compound at a concentration of 50 μM, the vitality of the cells was assessed using the MTT test (Mahmoud et al., 2022; Mekheimer et al., 2022). Refer to Supplementary Material A1 for more details.

#### 4.2.3 Antiproliferative assay

The antiproliferative activity of **9–29** was examined against four human cancer cell lines (colon - HT-29, pancreatic - Panc-1, lung - A-549, and breast - MCF-7) using Erlotinib as a reference. The MTT test was employed for this investigation (Hisham et al., 2022; El-Sherief et al., 2019; Ramadan et al., 2020). The IC_50_ values were derived from dose-response tests. The reported data are derived from at least two independent studies, each comprising three replicates per concentration. See Supplementary Material A1 for more details.

#### 4.2.4 EGFR inhibitory assay

The EGFR-TK assay (Al-Wahaibi et al., 2022; Alshammari et al., 2022) was used to assess the inhibitory activity of the most potent antiproliferative compounds **14**, **17**, **19**, **22**, **25**, and **29**, against EGFR. Erlotinib was used as the reference compound. Refer to Supplementary Material A1 for more details.

#### 4.2.5 VEGFR-2 inhibitory assay

An *in vitro* study examined the anti-VEGFR-2 activity of compounds **14**, **17**, **19**, **22**, **25**, and **29** (Marzouk et al., 2020; Mahmoud et al., 2024), using Sorafenib as the reference drug. See Supplementary Material A1 for more details.

#### 4.2.6 BAX and Bcl2 assays

Compounds **22** and **29**, the most potent derivatives, were further investigated against the Bax/Bcl2 ratio using Staurosporine as the reference drug (Youssif et al., 2019). See Supplementary Material A1 for more details.

### 4.3 Docking study

Molecular docking simulations of 15 derivatives (9a-o) were performed via Molecular Operating Environment (MOE^®^) software according to reported protocols (Abou-Zied et al., 2023) within the active site of EGFR tyrosine kinase domain (PDB ID: 1M17), and VEGFR-2 (PDB ID: 3WZE) crystals structures downloaded from RSCB protein data bank (https://www.rcsb.org/). For more details, see Supplementary Material A1.

### 4.4 Calculations of SwissADME

Pharmacokinetics and drug-likeness prediction for all the newly synthesized compounds was performed using the online tool SwissADME predictor software (http://www.swissadme.ch/) made by the Swiss Institute of Bioinformatics.

## Data Availability

The original contributions presented in the study are included in the article/Supplementary Material, further inquiries can be directed to the corresponding authors.
