# Supplementary material for: New series of 4,6-diaryl pyrimidines: facile synthesis and antiproliferative activity as dual EGFR/VEGFR-2 inhibitors
Source: Front Chem. 2024 Nov 6;12:1498104. doi: 10.3389/fchem.2024.1498104 (PMC11576293; doi:10.3389/fchem.2024.1498104)
Supplement: Supplementary file 1 [file DataSheet1.PDF]

Supporting Information

New series of 4,6-diaryl pyrimidines: Facile synthesis and antiproliferative activity as dual EGFR/VEGFR-2 inhibitors

Yaser A. Mostafa[1,2]* Jalil Abdeljalil Assoud[3], Ahmed Y Desoky[3], Samy Mohamady[4], Nesma M. Mohamed[5,6], Ola I. A. Salem[1], Zainab M. Almarhoon[7], Stefan Bräse[8]*, Bahaa G. M. Youssif[1]*.

[1]Pharmaceutical Organic Chemistry Department, Faculty of Pharmacy, Assiut University, Assiut 71526, Egypt; [2]Pharmaceutical Chemistry Department, Faculty of Pharmacy, Badr University in Assiut, Assiut 2014101, Egypt; [3]Department of Chemistry, University of Waterloo, Waterloo, Ontario, Canada N2L 3G1; [4]Faculty of Pharmacy, The British University in Egypt, Cairo-Suez, Desert Road, Al-Sherouk 11837, Egypt; [5]Department of Pharmacognosy, Faculty of Pharmacy, Assiut University, Assiut 71526, Egypt; [6]Pharmacognosy Department, Faculty of Pharmacy, Badr University in Assiut, Assiut 71111, Egypt. [7]Department of Chemistry, College of Science, King Saud University, Riyadh 11451, Saudi Arabia; [8]Institute of Biological and Chemical Systems, IBCS-FMS, Karlsruhe Institute of Technology, 76131 Karlsruhe, Germany

*To whom correspondence should be addressed:

Yaser A. Mostafa, Ph.D. Pharmaceutical Chemistry Department, Faculty of Pharmacy, Badr University in Assiut 71111, Egypt.

E-mail: yaabdelkarem@Pharm.aun.edu.eg, y3abdelh@UWaterloo.ca

Bahaa G. M. Youssif, Ph.D. Pharmaceutical Organic Chemistry Department, Faculty of Pharmacy, Assiut University, Assiut 71526, Egypt.

Tel.: (002)-01098294419

E-mail address: bgyoussif2@gmail.com, bahaa.youssif@pharm.aun.edu.eg

Stefan Bräse

Institute of Biological and Chemical Systems, IBCS-FMS, Karlsruhe Institute of Technology, 76131 Karlsruhe, Germany. E-mail: braese@kit.edu

Supporting Information Index

S. No.	Content	Page no.
**Section 1.** NMR Data		
**S1.1**	^{1}H NMR for compound **3**	**4**
**S1.2**	^{1}H NMR for compound **4**	**5**
**S1.3**	^{1}H NMR for compound **5**	**6**
**S1.4**	^{1}H NMR for compound **6**	**7**
**S1.5 & S1.6**	^{1}H NMRs for compound **7** in $CDCl_3$ and DMSO-*d6*	**8-9**
**S1.7 & S1.8**	^{1}H NMR and ^{13}C NMR for compound **8**	**10-11**
**S1.9**	HMQC for compound **8**	**12**
**S1.10**	H-H Cosy for compound **8**	**13**
**S1.11 & S1.12**	^{1}H NMR and ^{13}C NMR for compound **9**	**14-15**
**S1.13 & S1.14**	^{1}H NMR and ^{13}C NMR for compound **10**	**16-17**
**S1.15 & S1.16**	^{1}H NMR and ^{13}C NMR for compound **11**	**18-19**
**S1.17 & S1.18**	^{1}H NMR and ^{13}C NMR for compound **12**	**20-21**
**S1.19 & S1.20**	^{1}H NMR and ^{13}C NMR for compound **13**	**22-23**
**S1.21 & S1.22**	^{1}H NMR and ^{13}C NMR for compound **14**	**24-25**
**S1.23 & S1.24**	^{1}H NMR and ^{13}C NMR for compound **15**	**26-27**
**S1.25 & S1.26**	^{1}H NMR and ^{13}C NMR for compound **16**	**28-29**
**S1.27 & S1.28**	^{1}H NMR and ^{13}C NMR for compound **17**	**30-31**
**S1.29 & S1.30**	^{1}H NMR and ^{13}C NMR for compound **18**	**32-33**
**S1.31 & S1.32**	^{1}H NMR and ^{13}C NMR for compound **19**	**34-35**
**S1.33 & S1.34**	^{1}H NMR and ^{13}C NMR for compound **20**	**36-37**
**S1.35 & S1.36**	^{1}H NMR and ^{13}C NMR for compound **21**	**38-39**
**S1.37 & S1.38**	^{1}H NMR and ^{13}C NMR for compound **22**	**40-41**
**S1.39 & S1.40**	^{1}H NMR and ^{13}C NMR for compound **23**	**42-43**
**S1.41 & S1.42**	^{1}H NMR and ^{13}C NMR for compound **24**	**44-45**
**S1.43 & S1.44**	^{1}H NMR and ^{13}C NMR for compound **25**	**46-47**
**S1.45 & S1.46**	^{1}H NMR and ^{13}C NMR for compound **26**	**48-49**
**S1.47 & S1.48**	^{1}H NMR and ^{13}C NMR for compound **27**	**50-51**

**S1.49 & S1.50**	^{1}H NMR and ^{13}C NMR for compound **28**	**52-53**
**S1.51 & S1.52**	^{1}H NMR and ^{13}C NMR for compound **29**	**54-55**
**Section 2. NCI Cancer Cell Panel Assays**		
**a.**	**Tables S1** and **S2**	**56-59**
**b.**	Monographs	**60-78**
**Section 3. Wound Closure Assays Images** (compounds 26 & 27 and control)		
**a.**	**Table S3**. Wound Healing Activity of Compounds 26 & 27	**79**
**b.**	Wound closure Images	**79-82**
**Section 4**. **Experimental Lipophilicity Calculations**		
**Table S4**	Determination of Partition Coefficient (lipophilicity measurements) Experiment	**83**
**Section 5. ADME Calculations**		
**Table S5**	Physicochemical Descriptors of Test Compounds	**84**
**Section 6. X-Ray Coordinates of Compound 14**		
**Tables S6- S11**		**85-94**

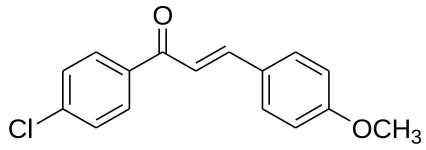

(3)

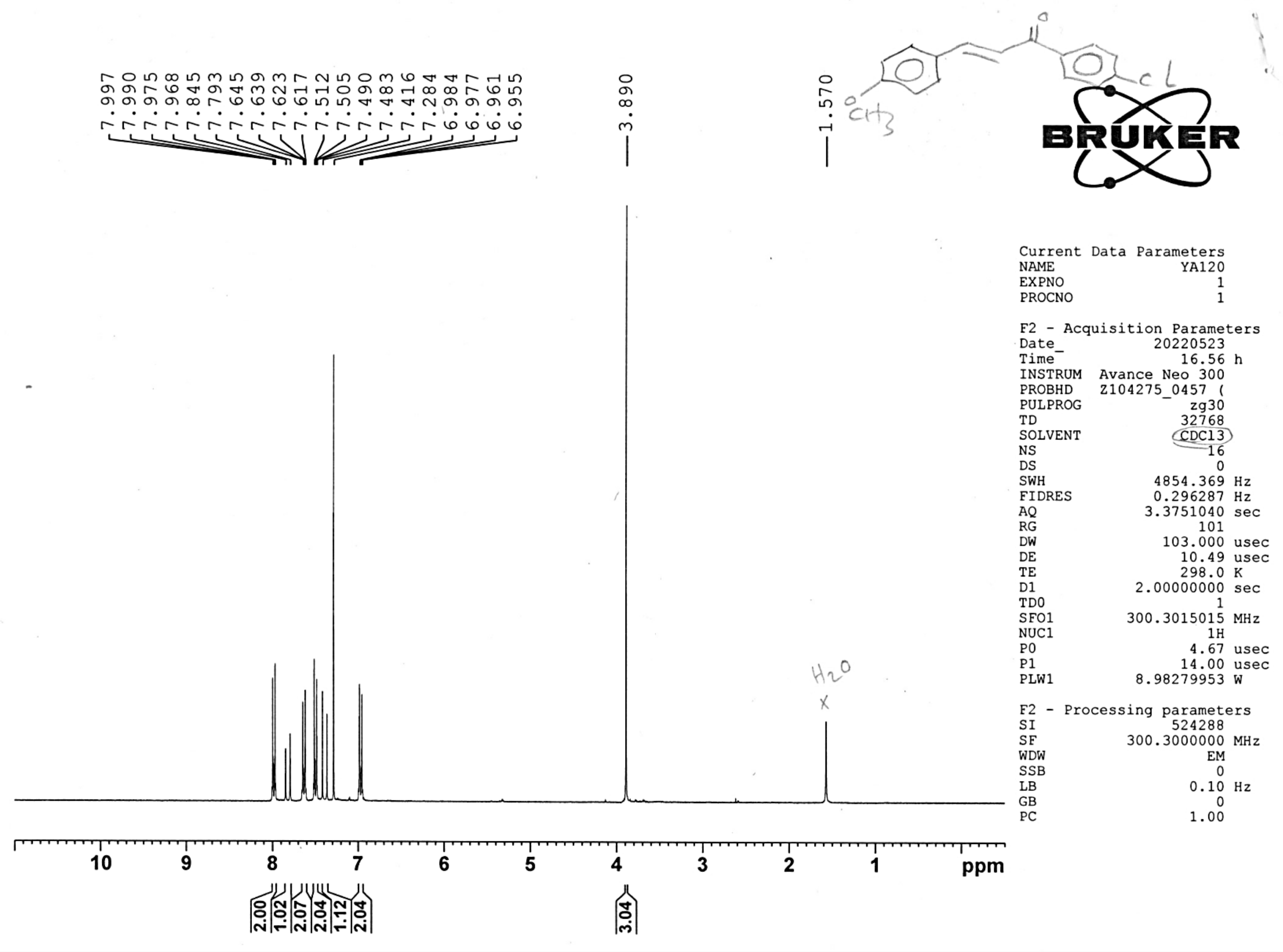

Figure S1.1. ^{1}H NMR of compound **3** ($CDCl_3$)

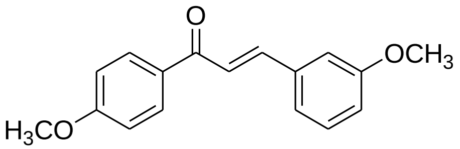

(4)

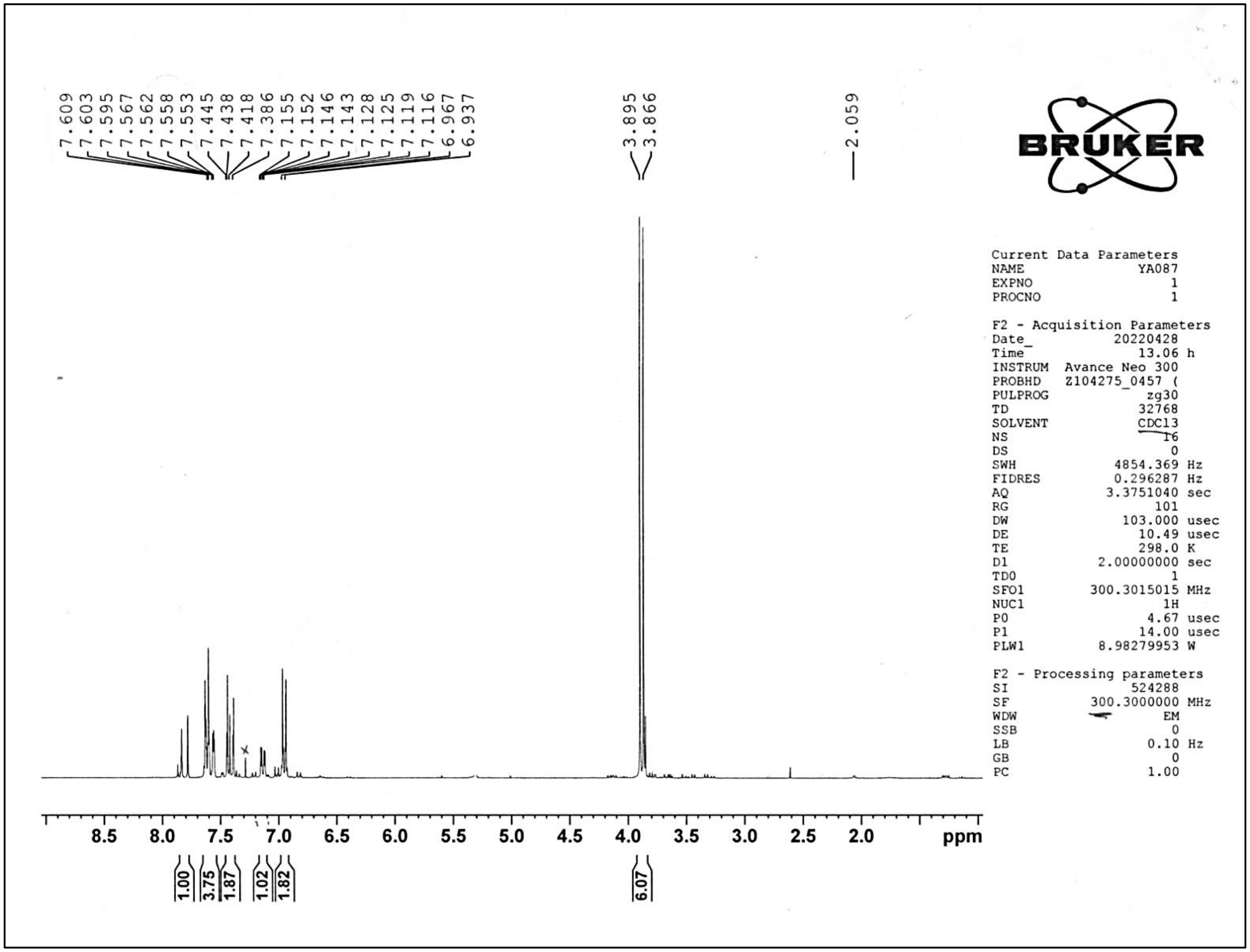

Figure S1.2. ^{1}H NMR of compound **4** ($CDCl_3$)

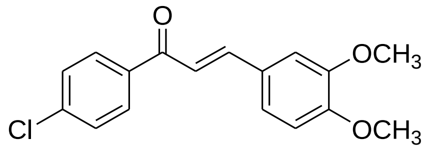

(5)

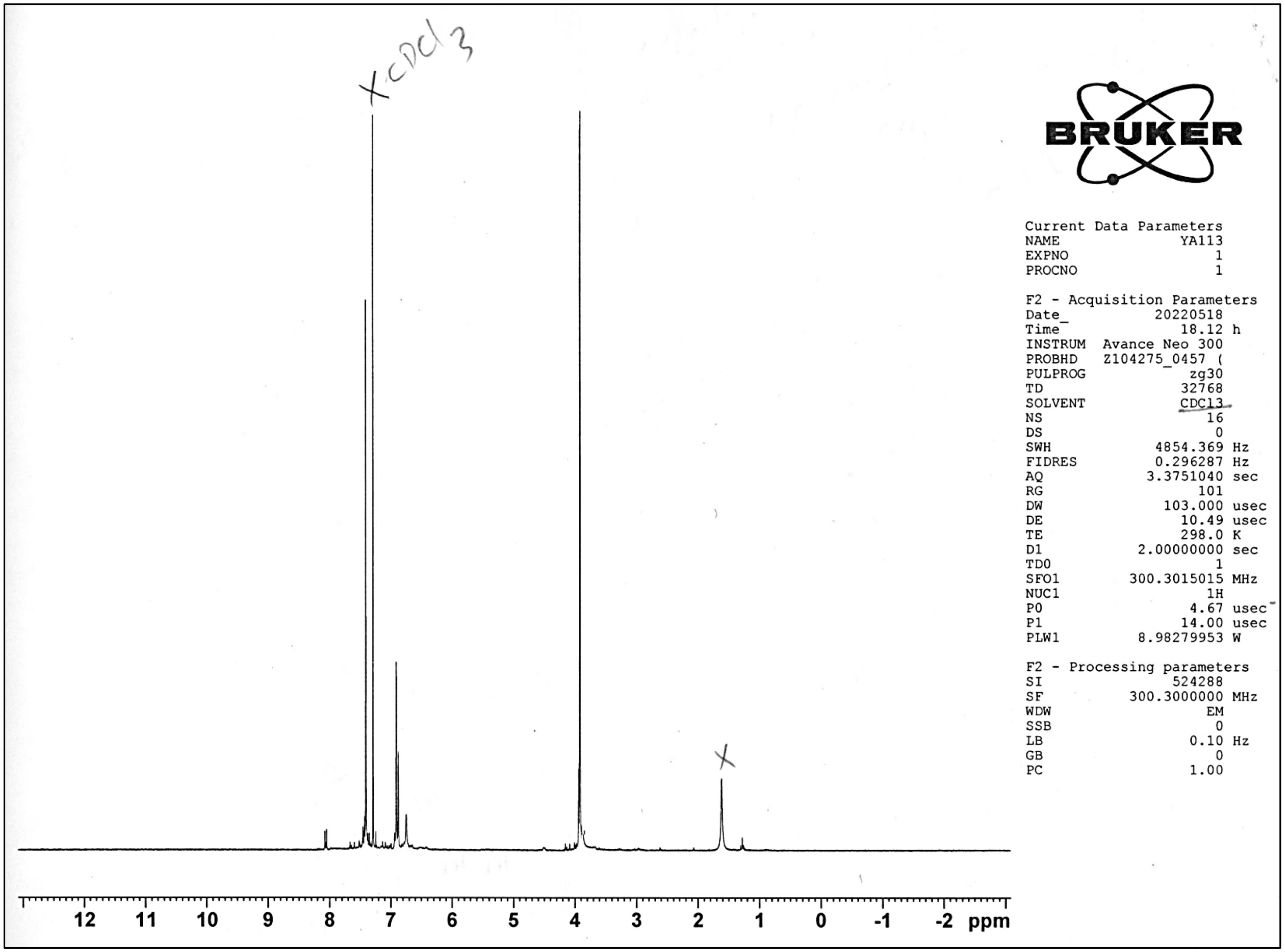

Figure S1.3. ^{1}H NMR of compound **5** ($CDCl_3$)

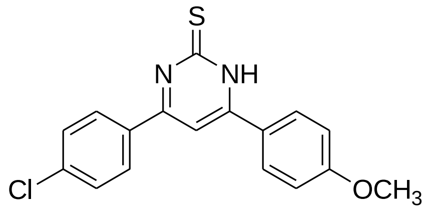

(6)

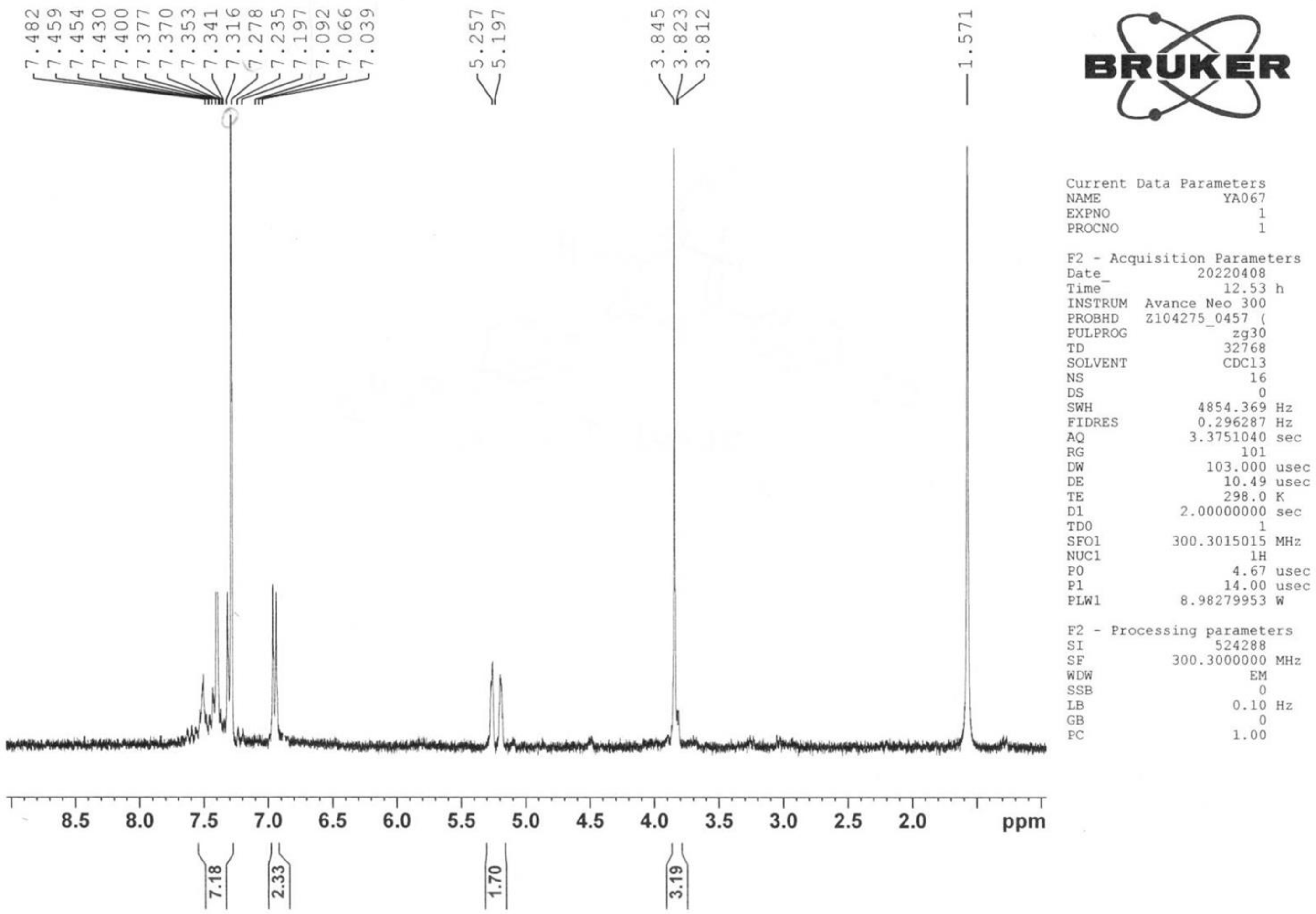

Figure S1.4. ^{1}H NMR of compound **6** ($CDCl_3$)

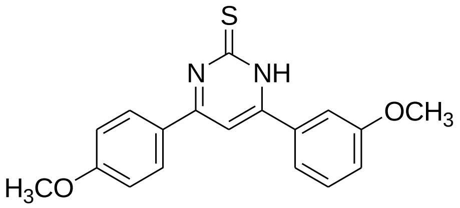

(7) (b-form)

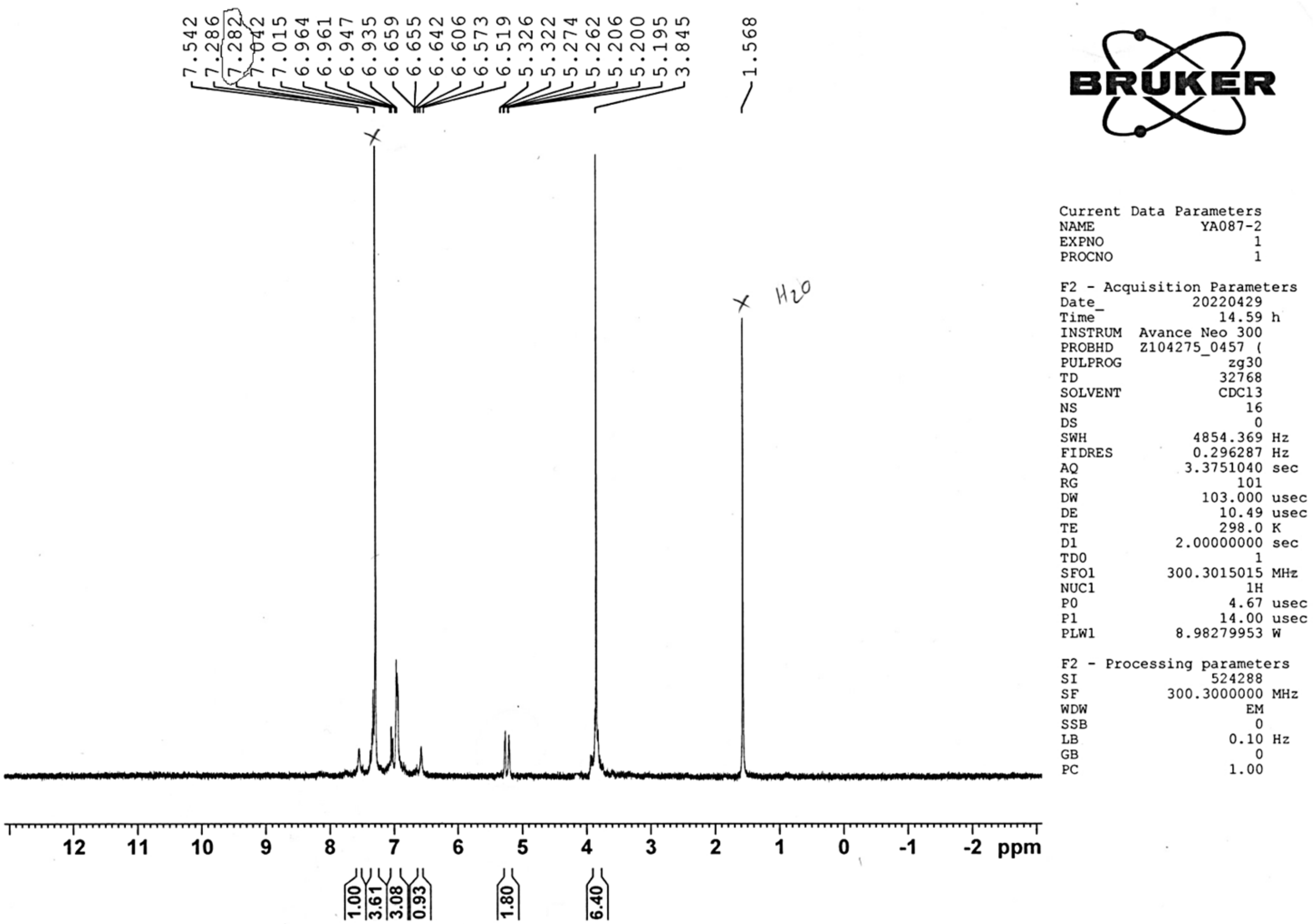

Figure S1.5. ^{1}H NMR of compound **7** ($CDCl_3$)

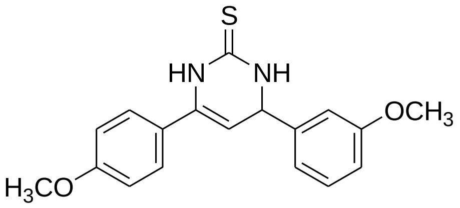

(7) (c-form)

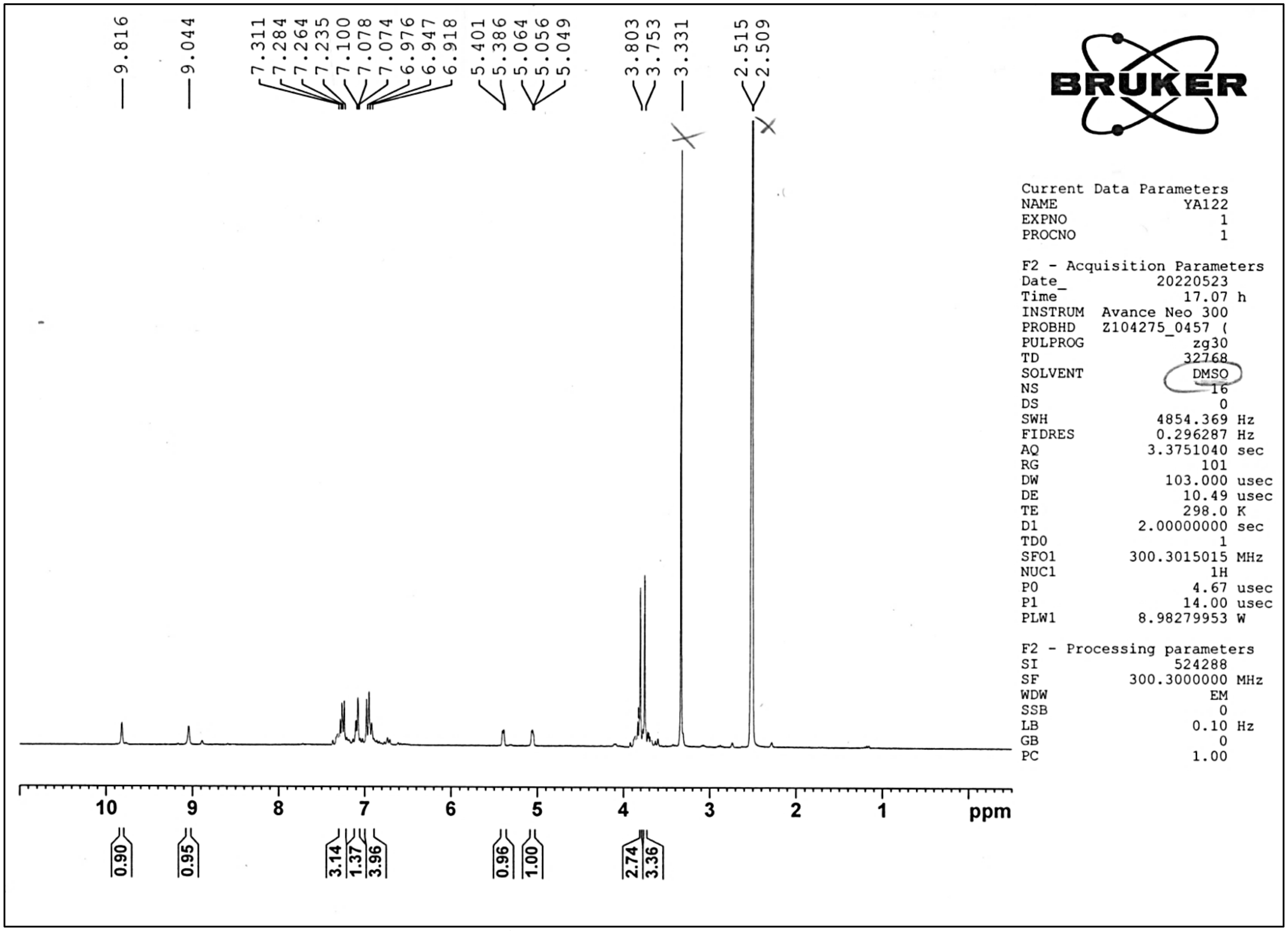

Figure S1.6. ^{1}H NMR of compound **7** (DMSO-d_6)

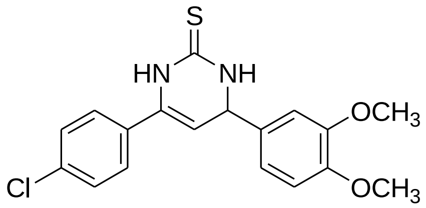

(8)

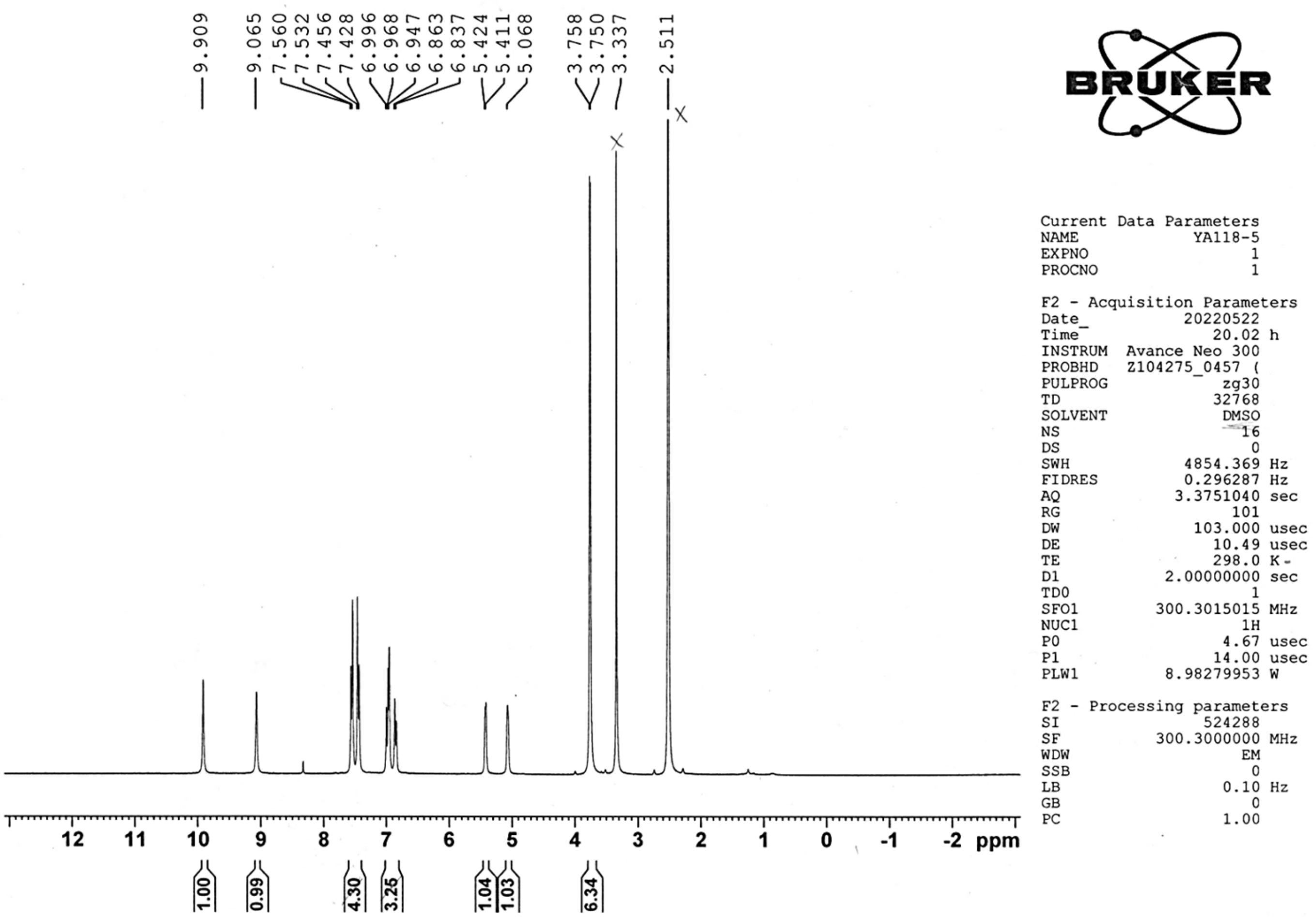

Figure S1.7. ^{1}H NMR of compound **8** (DMSO-d_6)

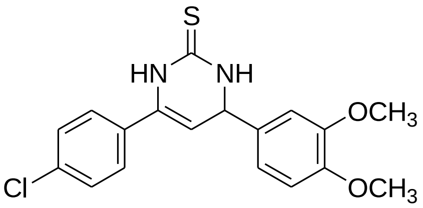

(8)

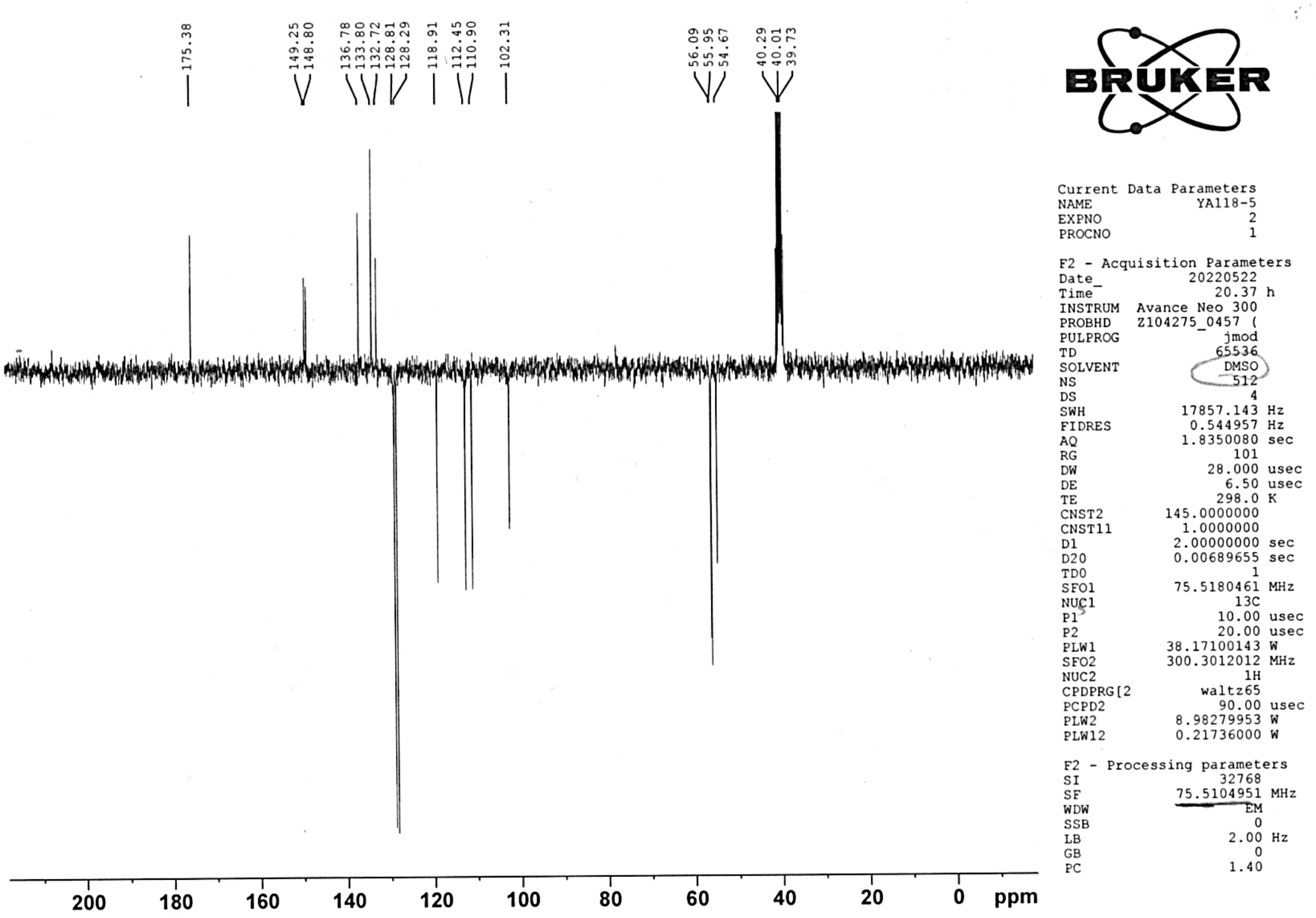

Figure S1.8. ^{13}C NMR of compound **8** (DMSO-d_6)

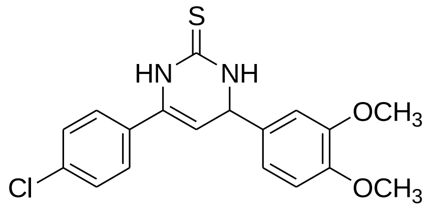

(8)

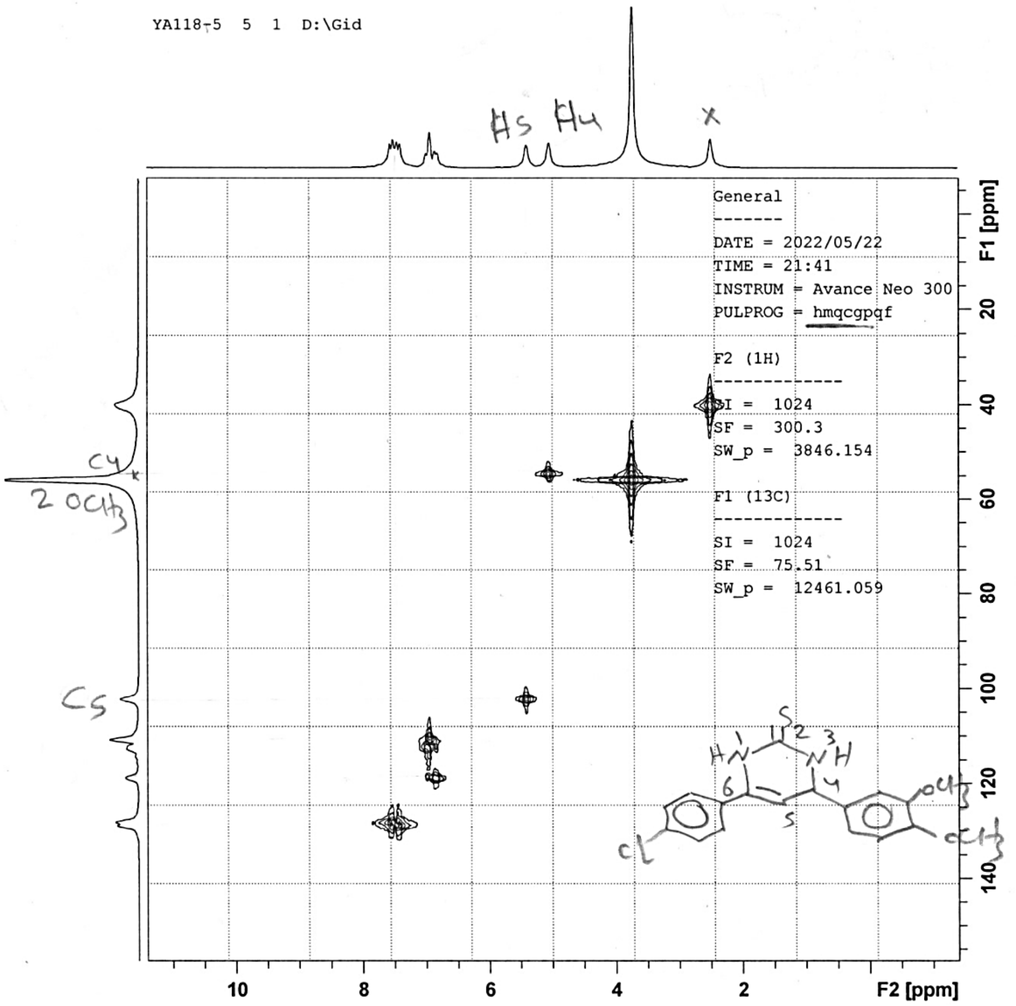

Figure S1.9. HMQC of compound **8** (DMSO-d_6)

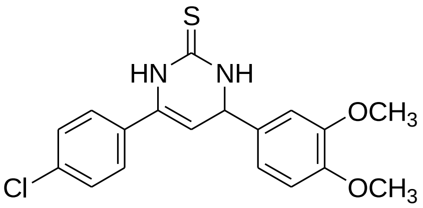

(8)

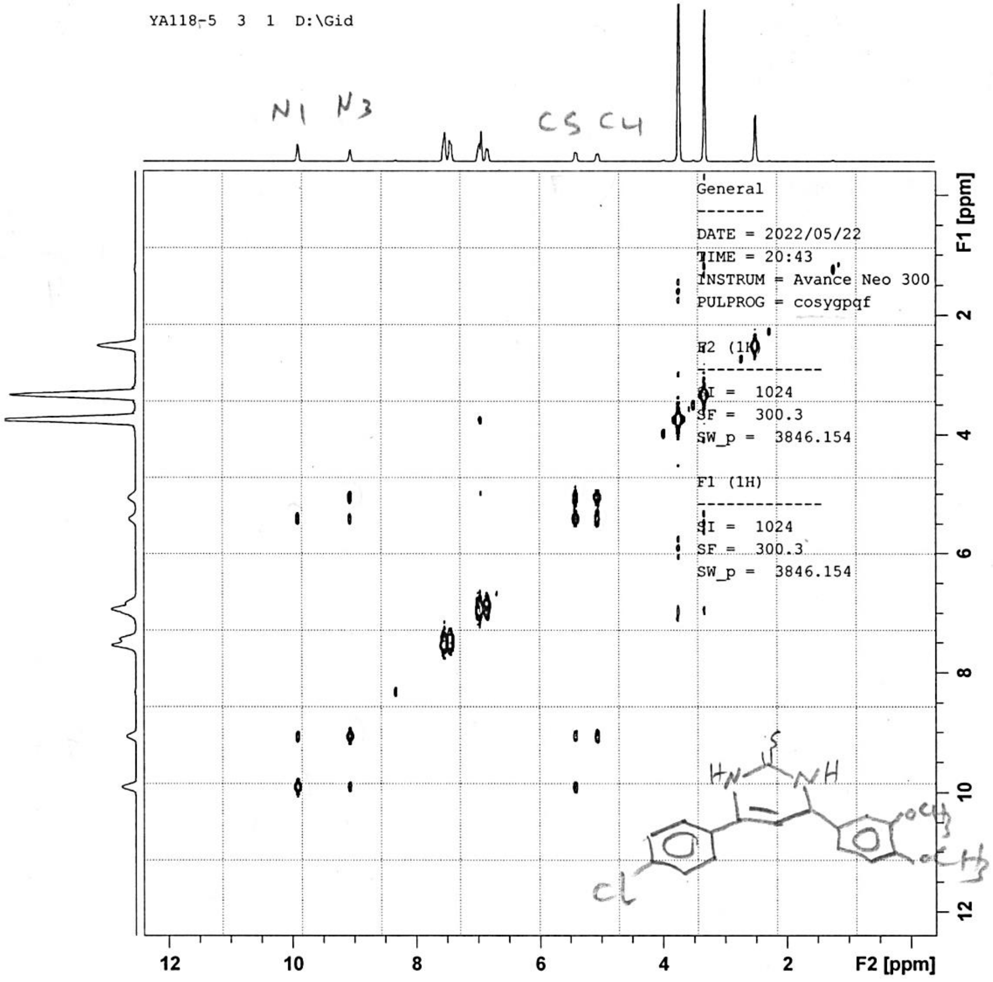

Figure S1.10. H-H Cosy of compound **8** (DMSO-d_6)

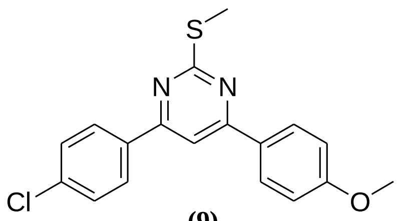

(9)

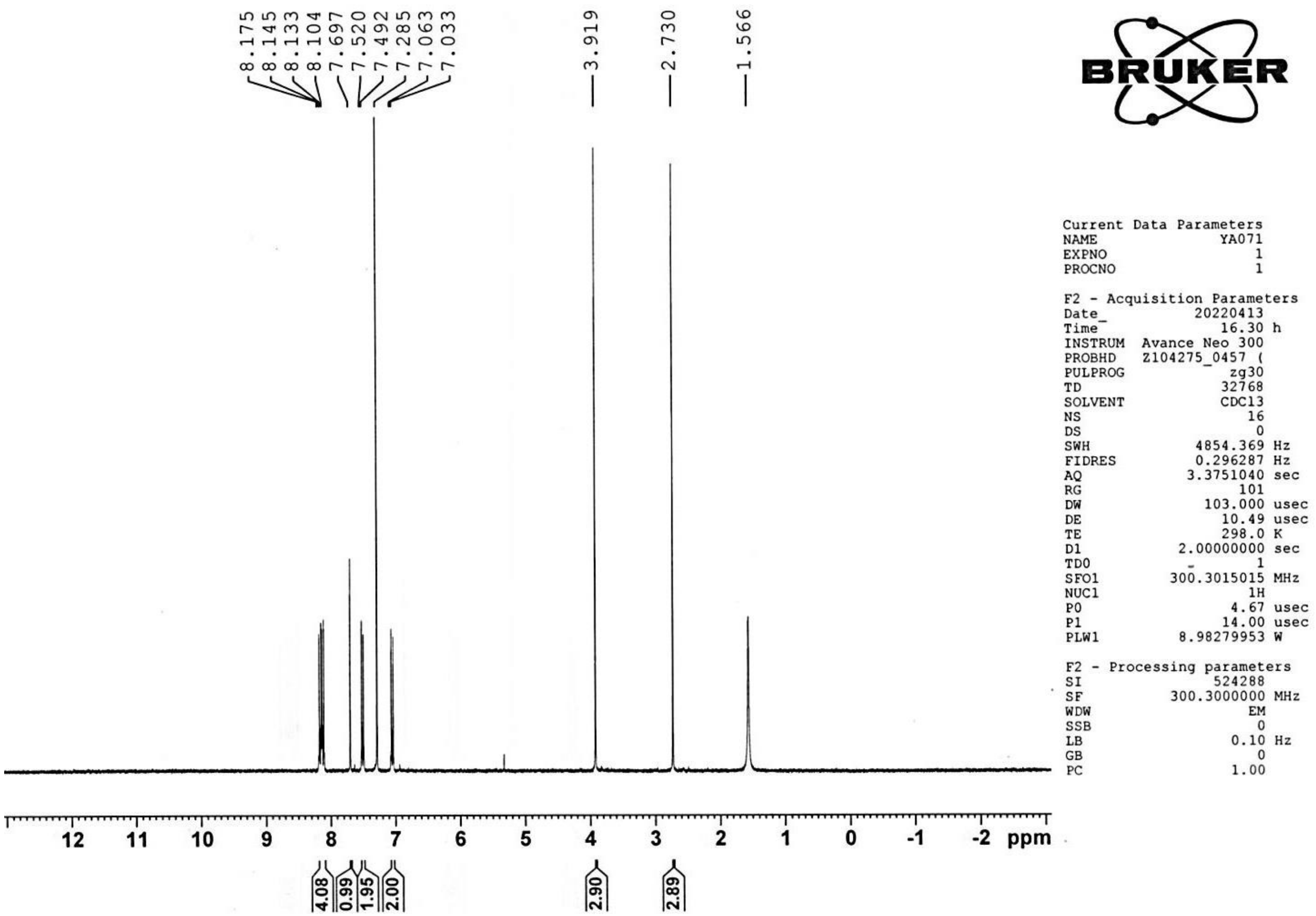

Figure S1.11. ^{1}H NMR of compound **9** ($CDCl_3$)

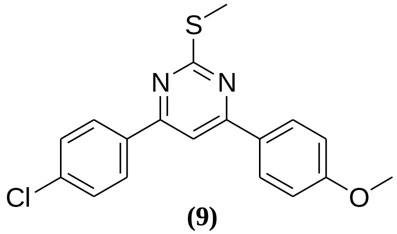

(9)

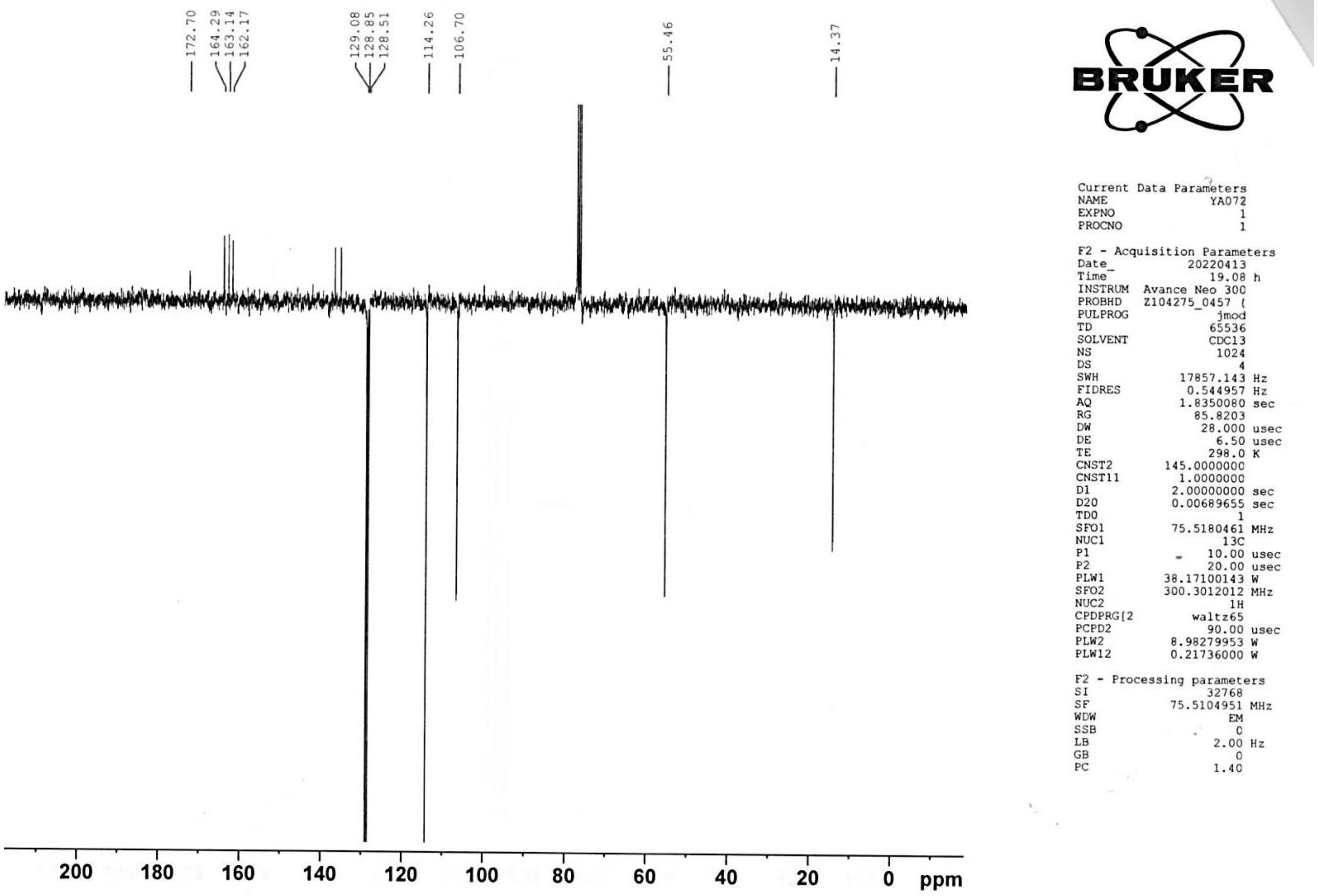

Figure S1.12. ^{13}C NMR of compound **9** ($CDCl_3$)

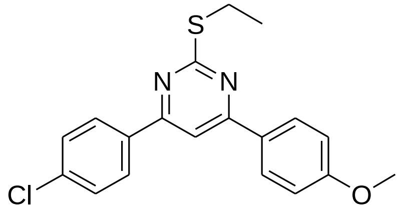

(10)

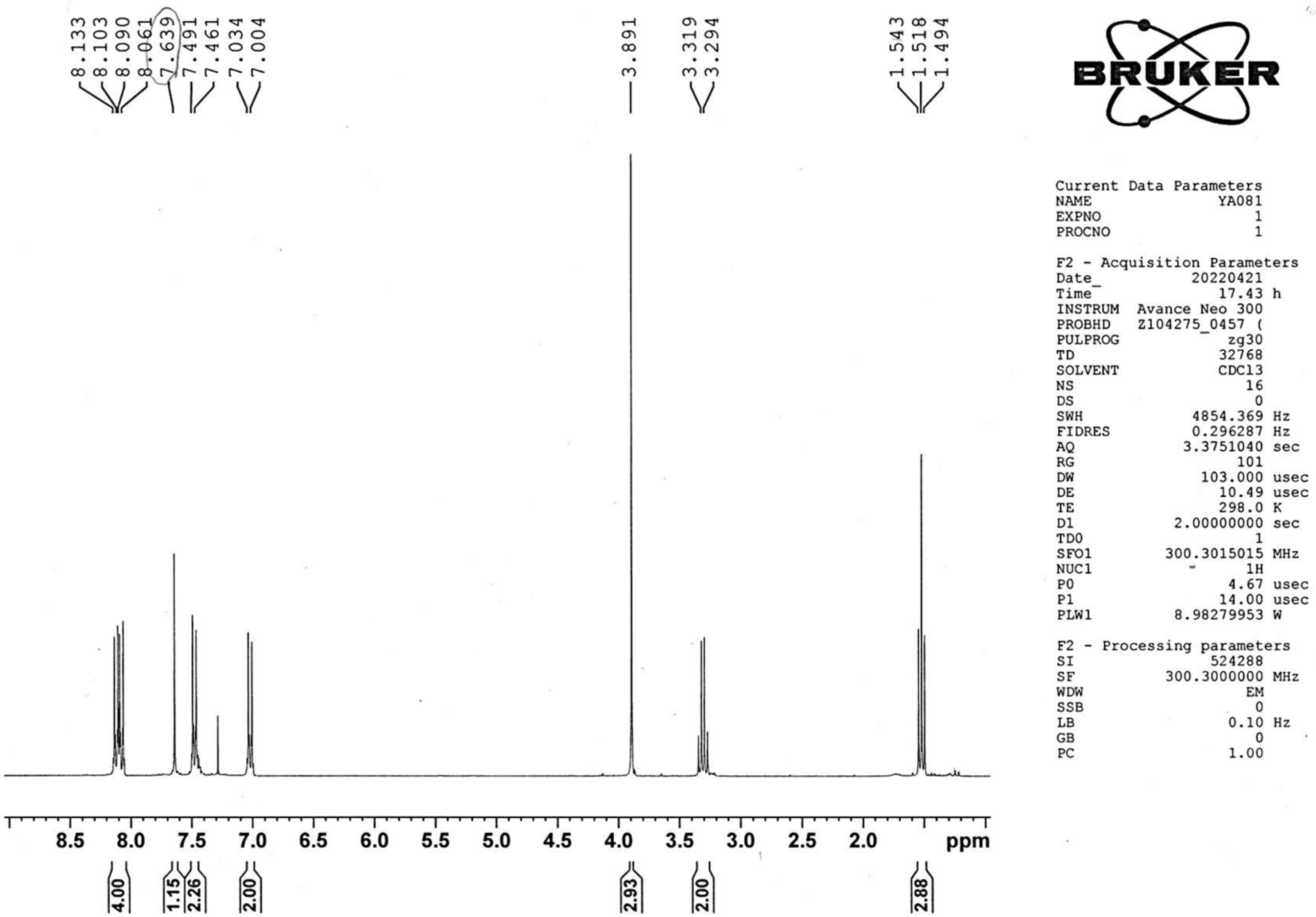

Figure S1.13. ^{1}H NMR of compound **10** ($CDCl_3$)

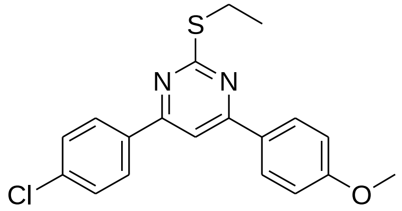

(10)

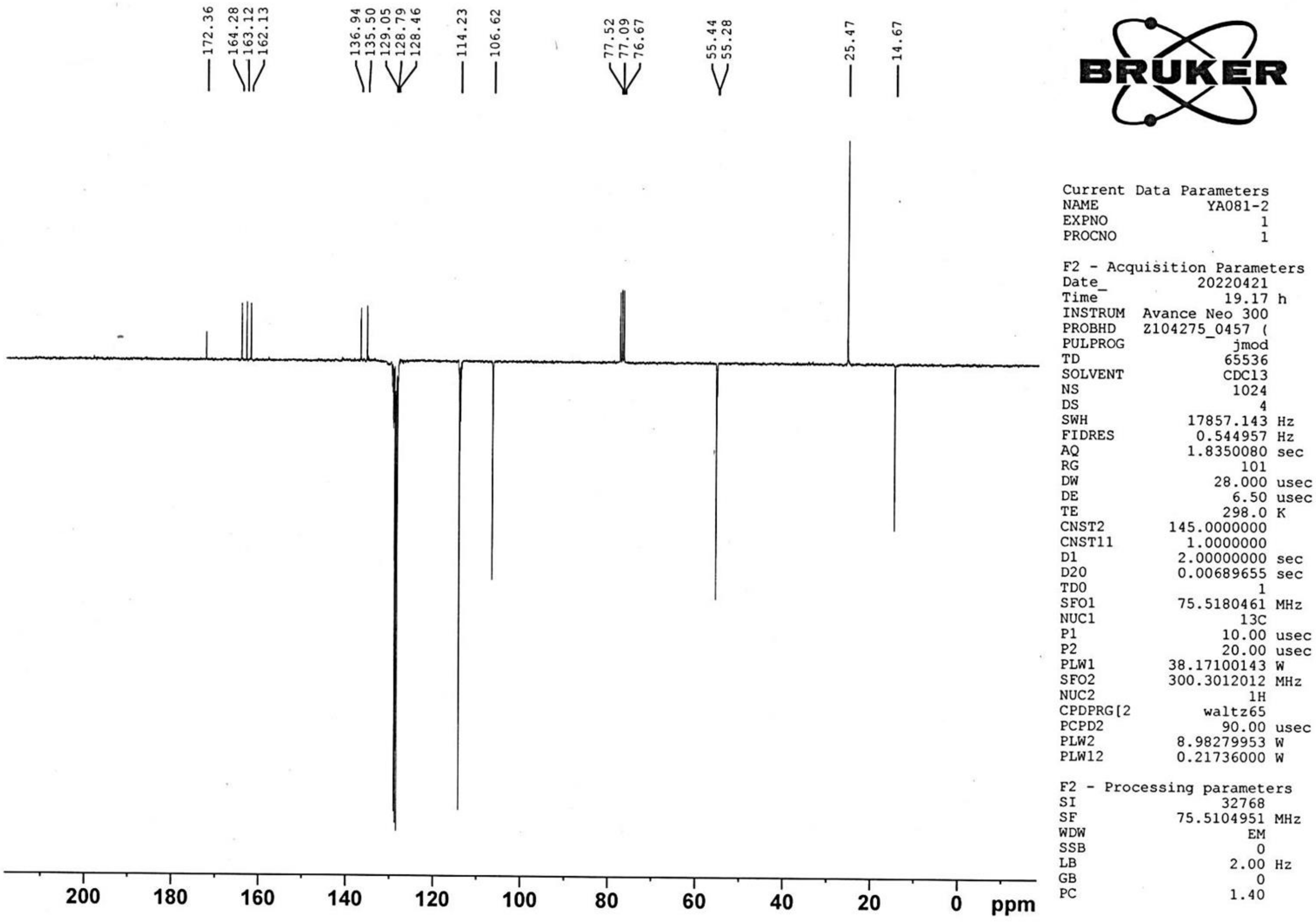

Figure S1.14. ^{13}C NMR of compound **10** ($CDCl_3$)

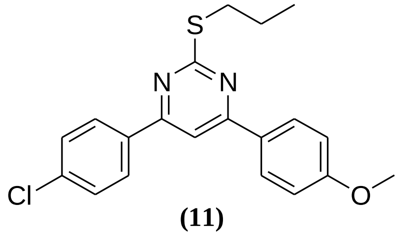

(11)

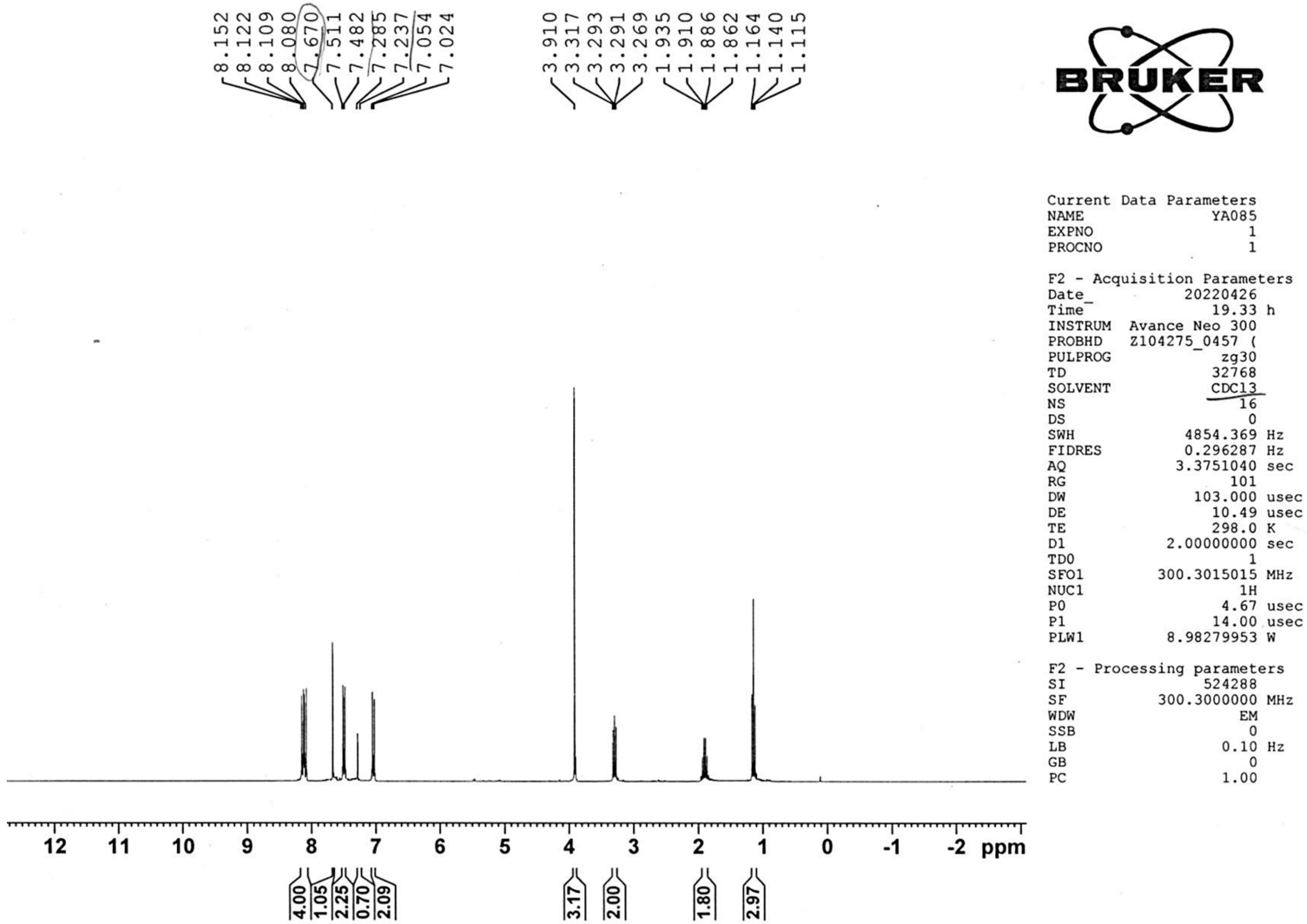

Figure S1.15. ^{1}H NMR of compound **11** ($CDCl_3$)

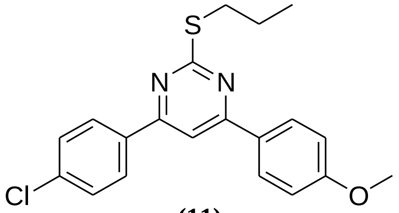

(11)

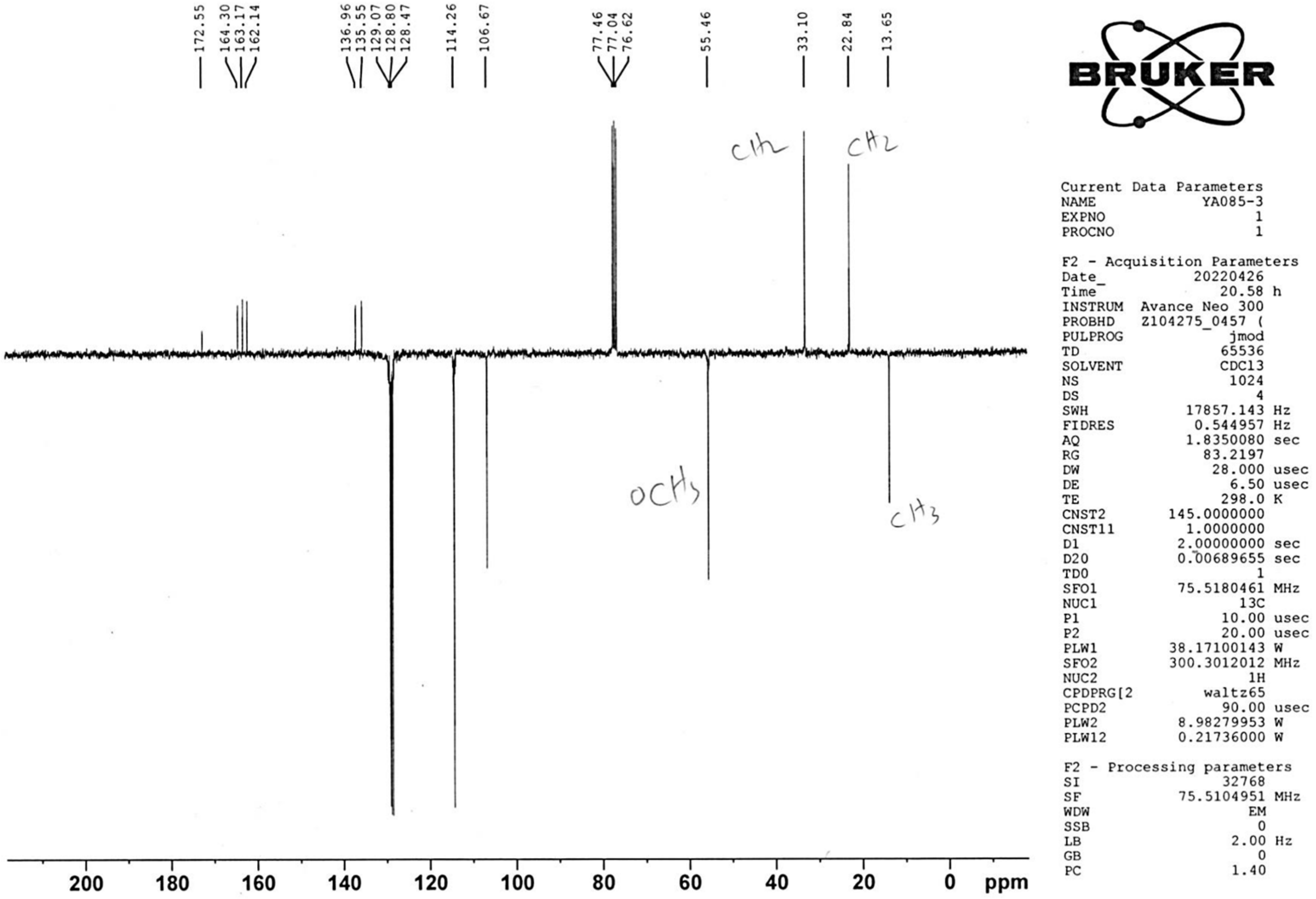

Figure S1.16. ^{13}C NMR of compound **11** ($CDCl_3$)

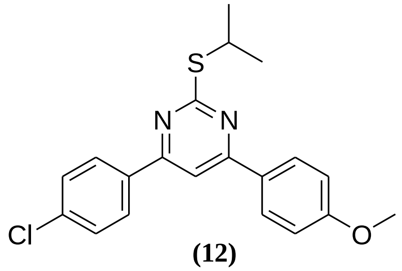

(12)

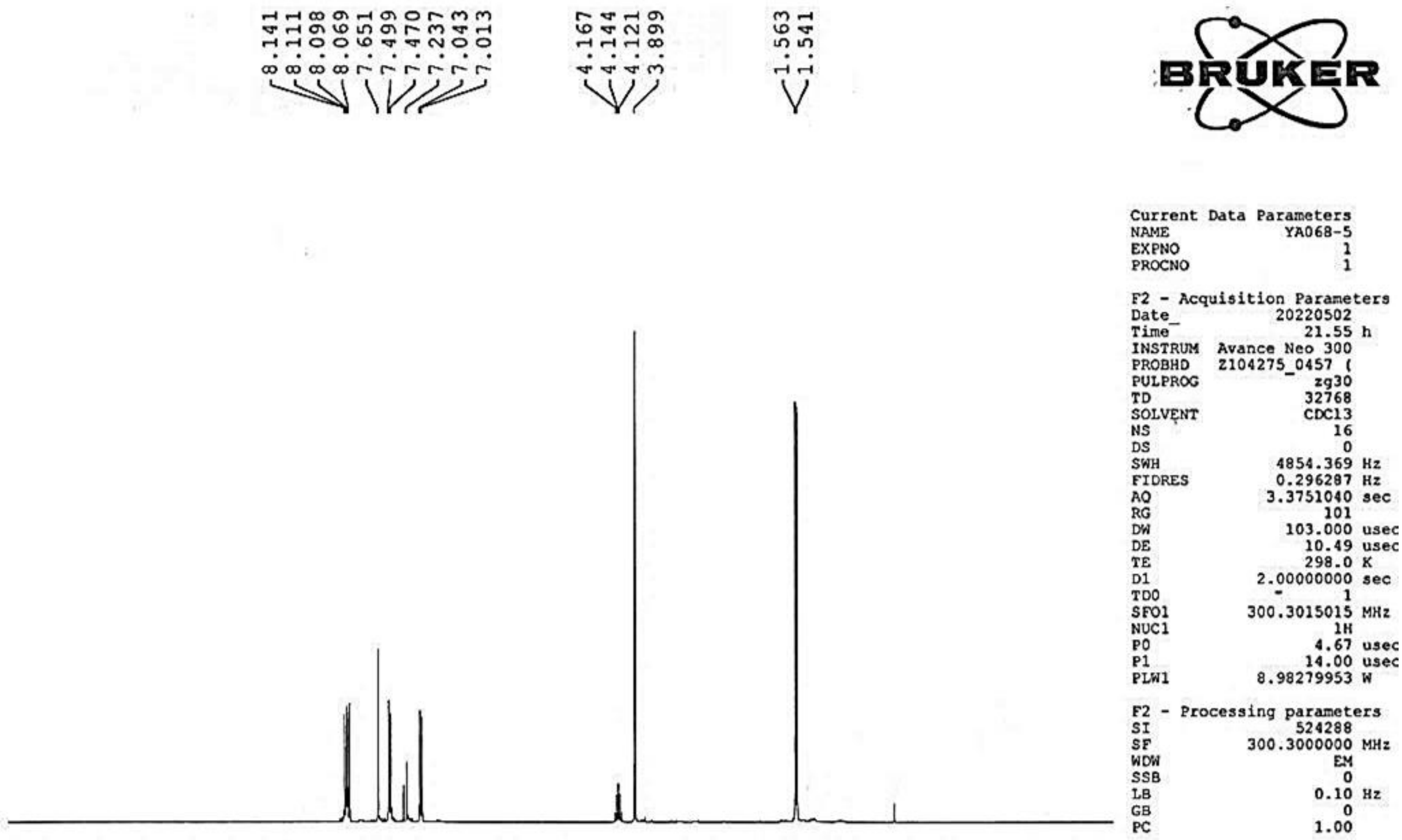

Figure S1.17. ^{1}H NMR of compound **12** ($CDCl_3$)

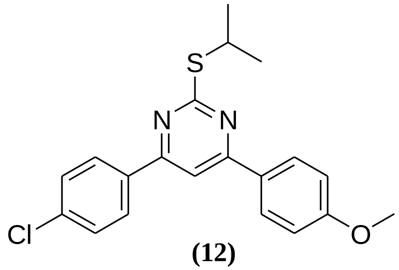

(12)

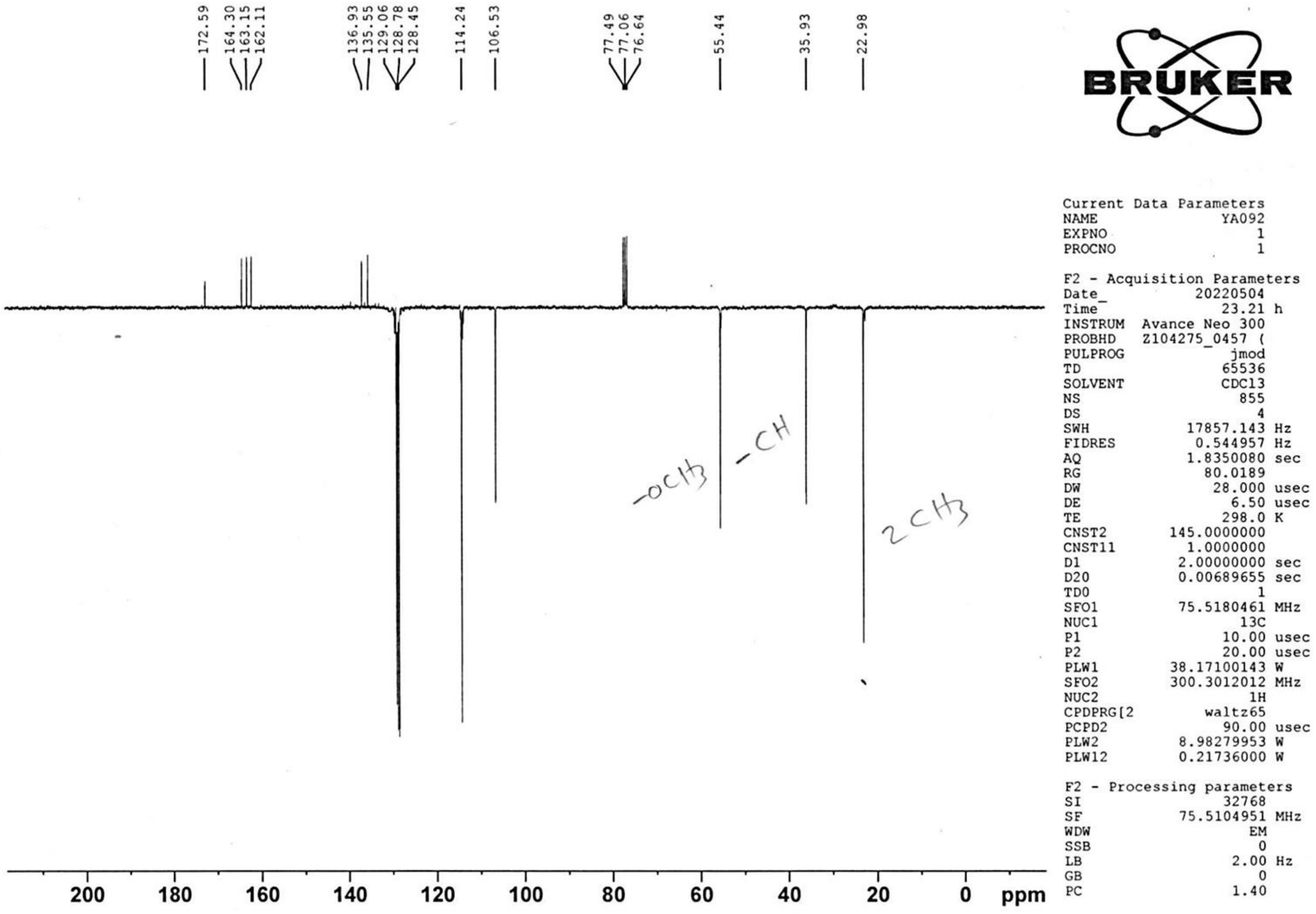

Figure S1.18. ^{13}C NMR of compound **12** ($CDCl_3$)

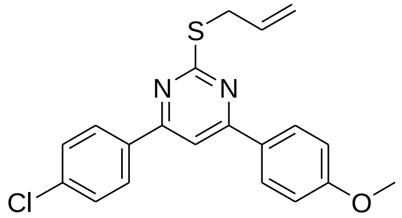

(13)

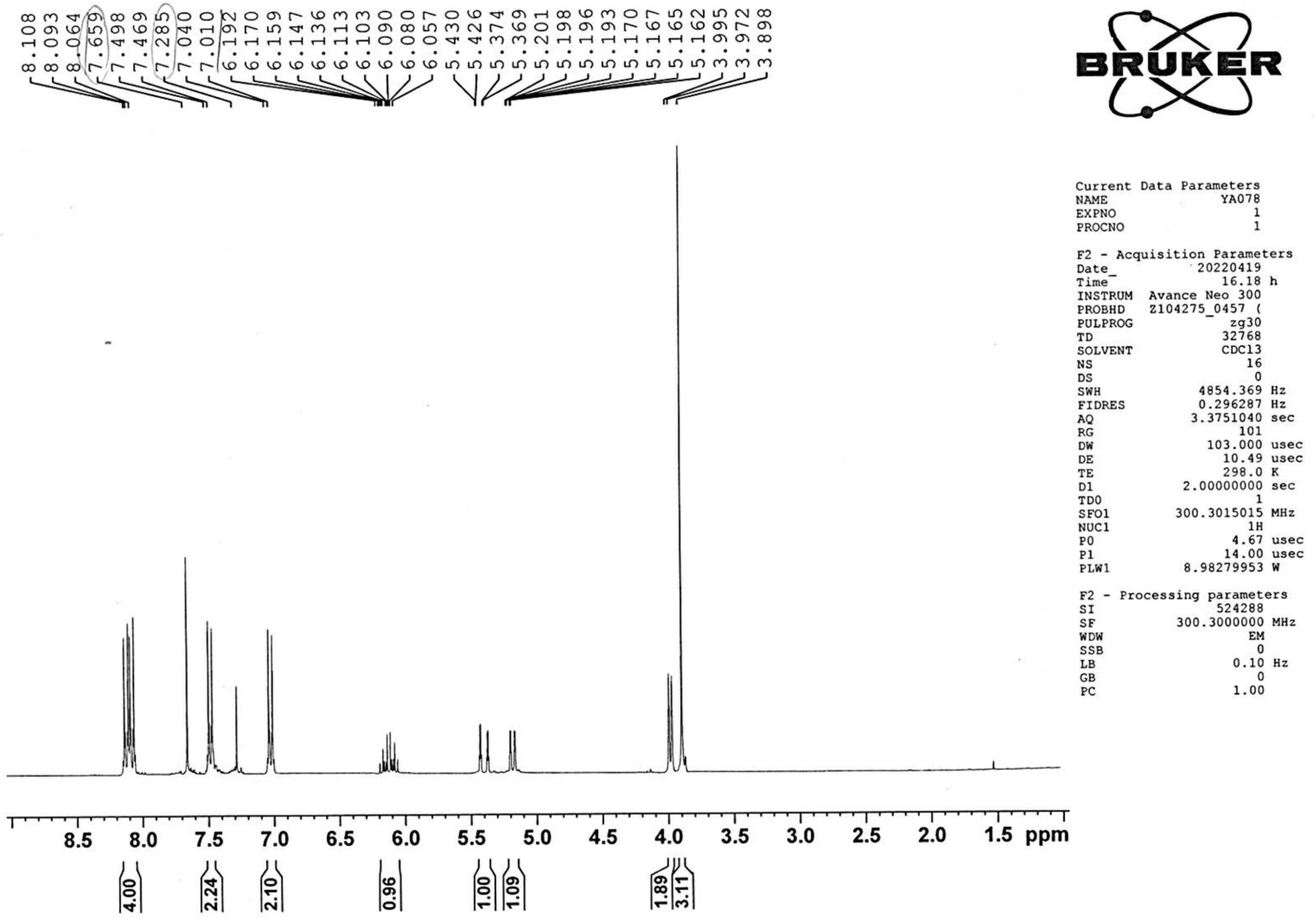

Figure S1.19. ^{1}H NMR of compound **13** ($CDCl_3$)

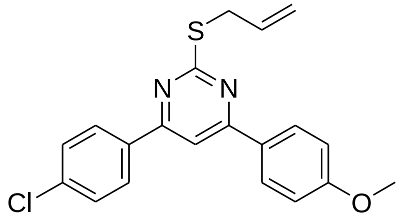

(13)

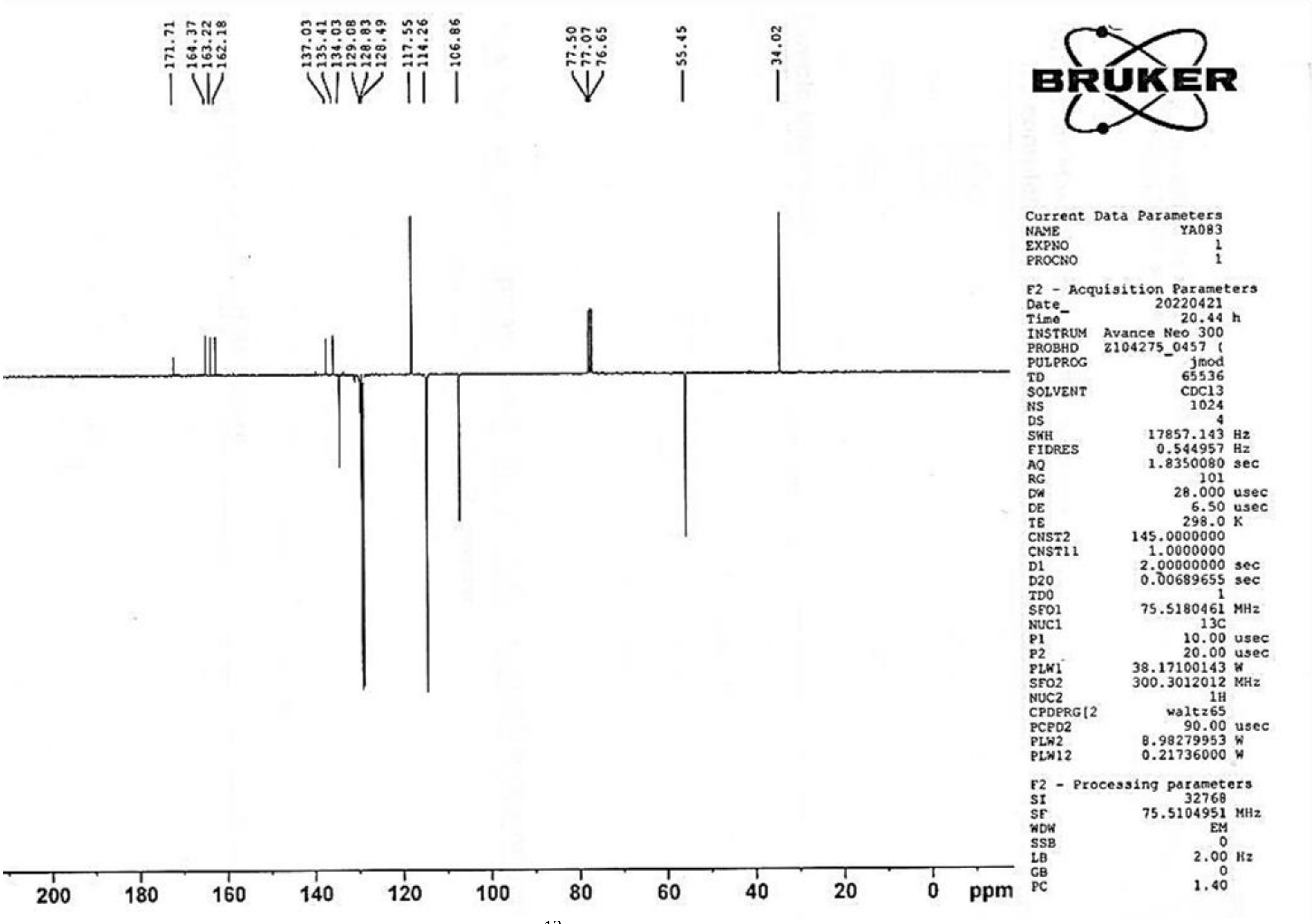

Figure S1.20. ^{13}C NMR of compound **13** ($CDCl_3$)

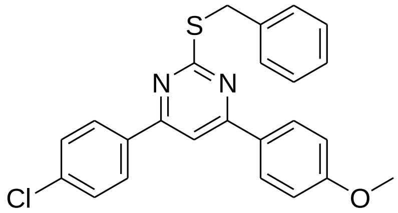

(14)

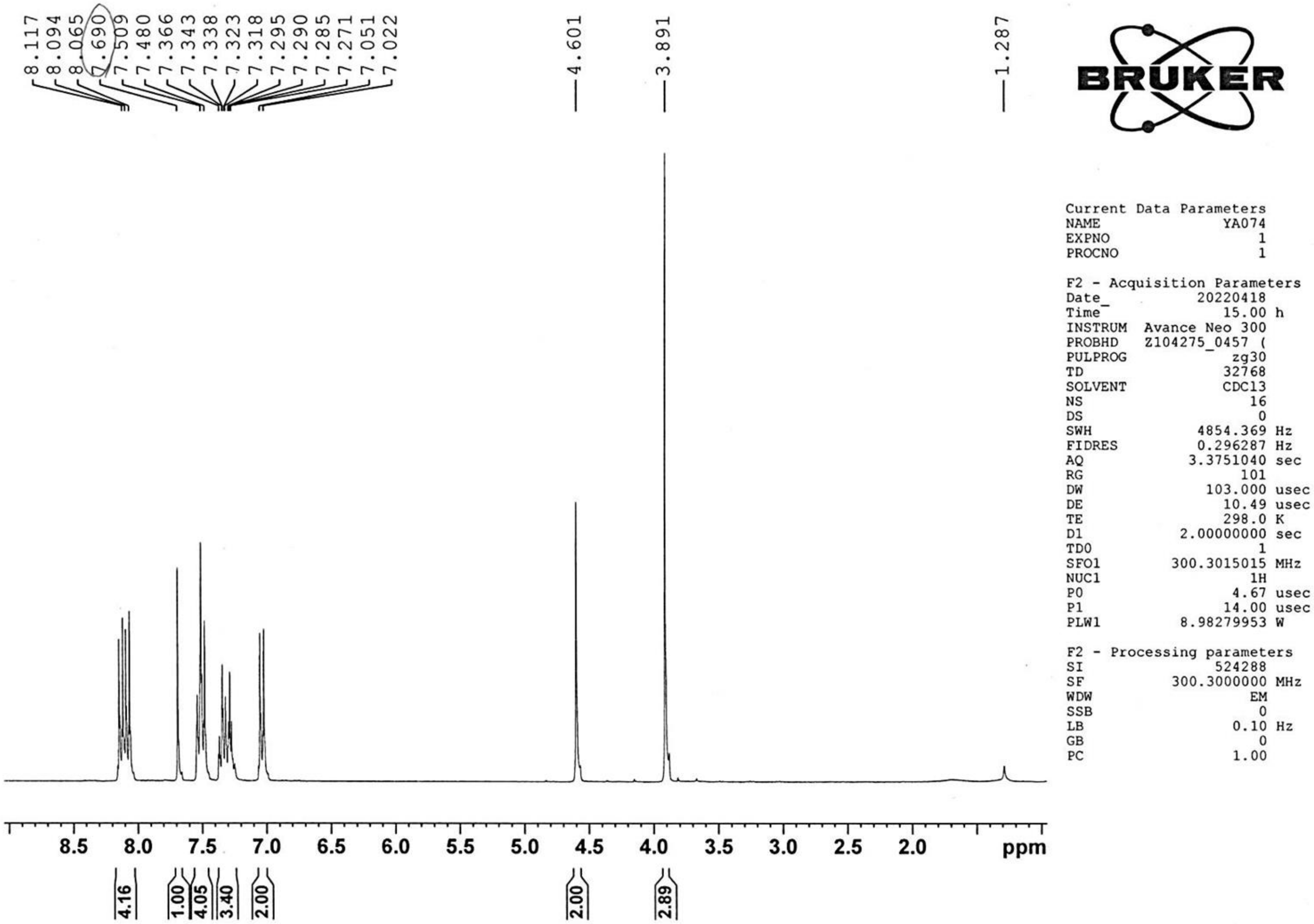

Figure S1.21. ^{1}H NMR of compound **14** ($CDCl_3$)

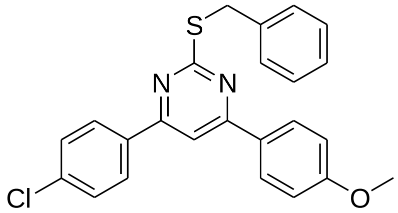

(14)

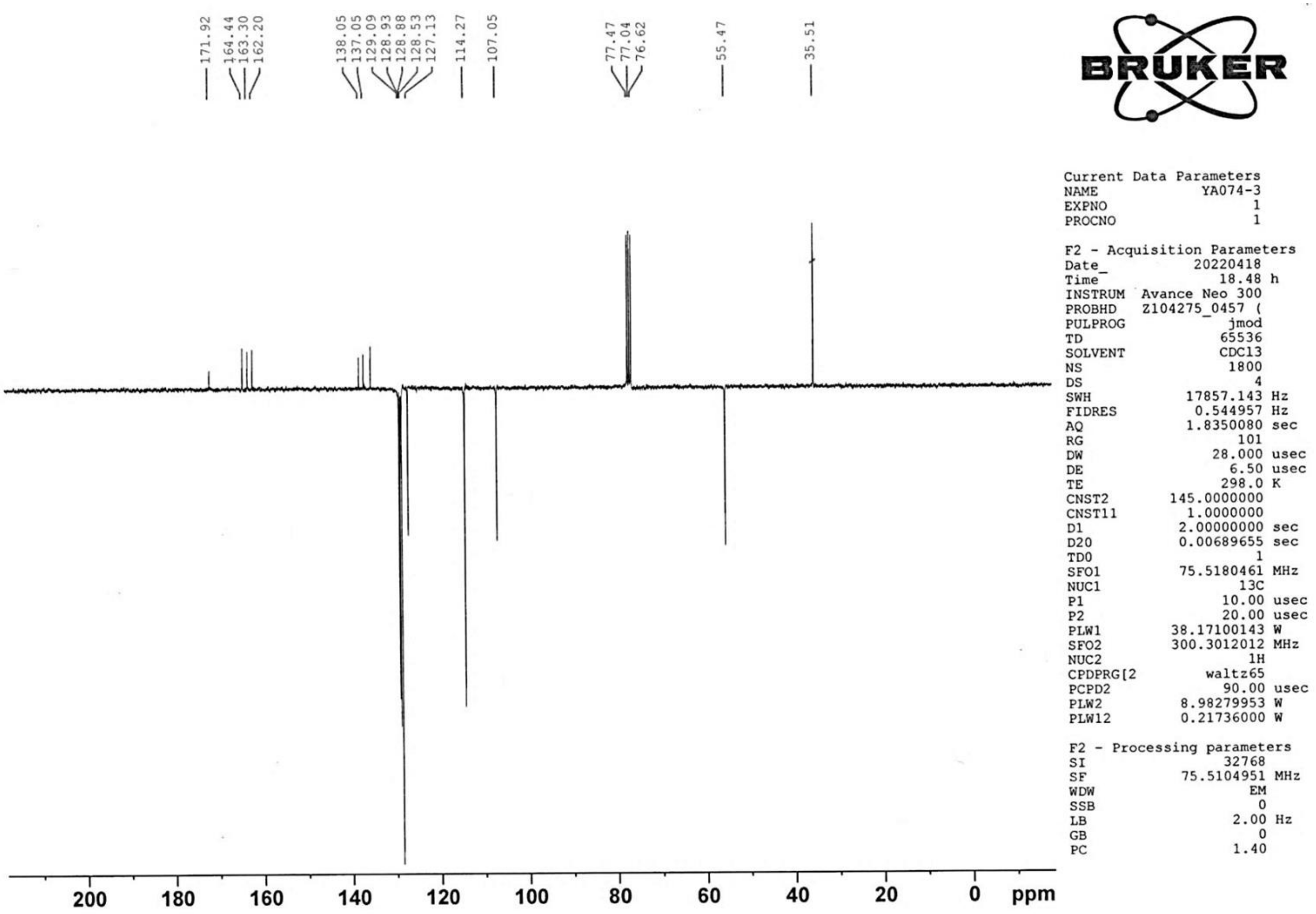

Figure S1.22. ^{13}C NMR of compound **14** ($CDCl_3$)

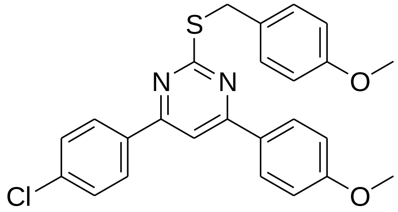

(15)

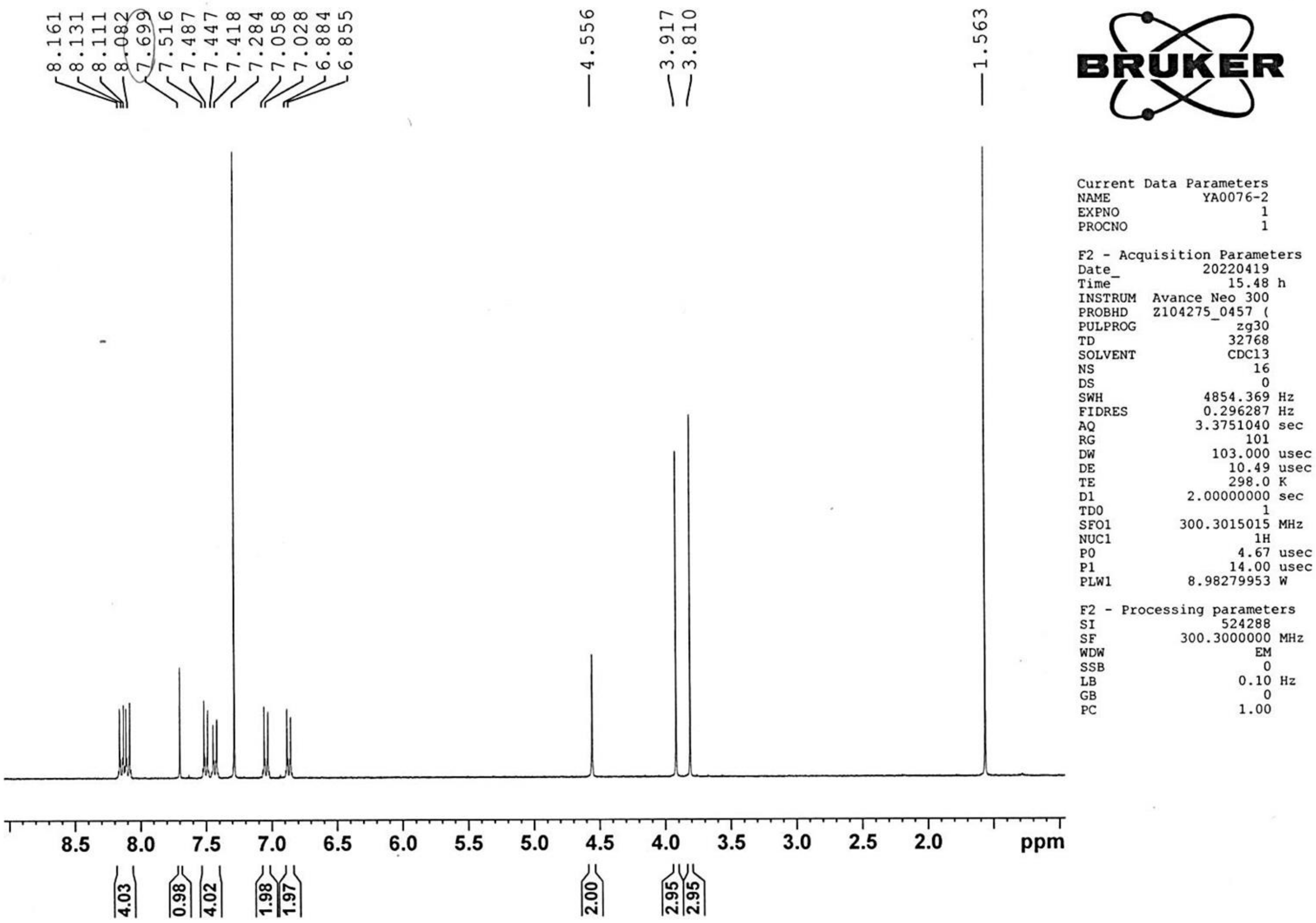

Figure S1.23. ^{1}H NMR of compound **15** ($CDCl_3$)

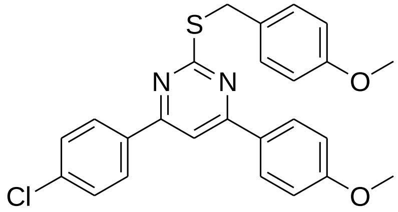

(15)

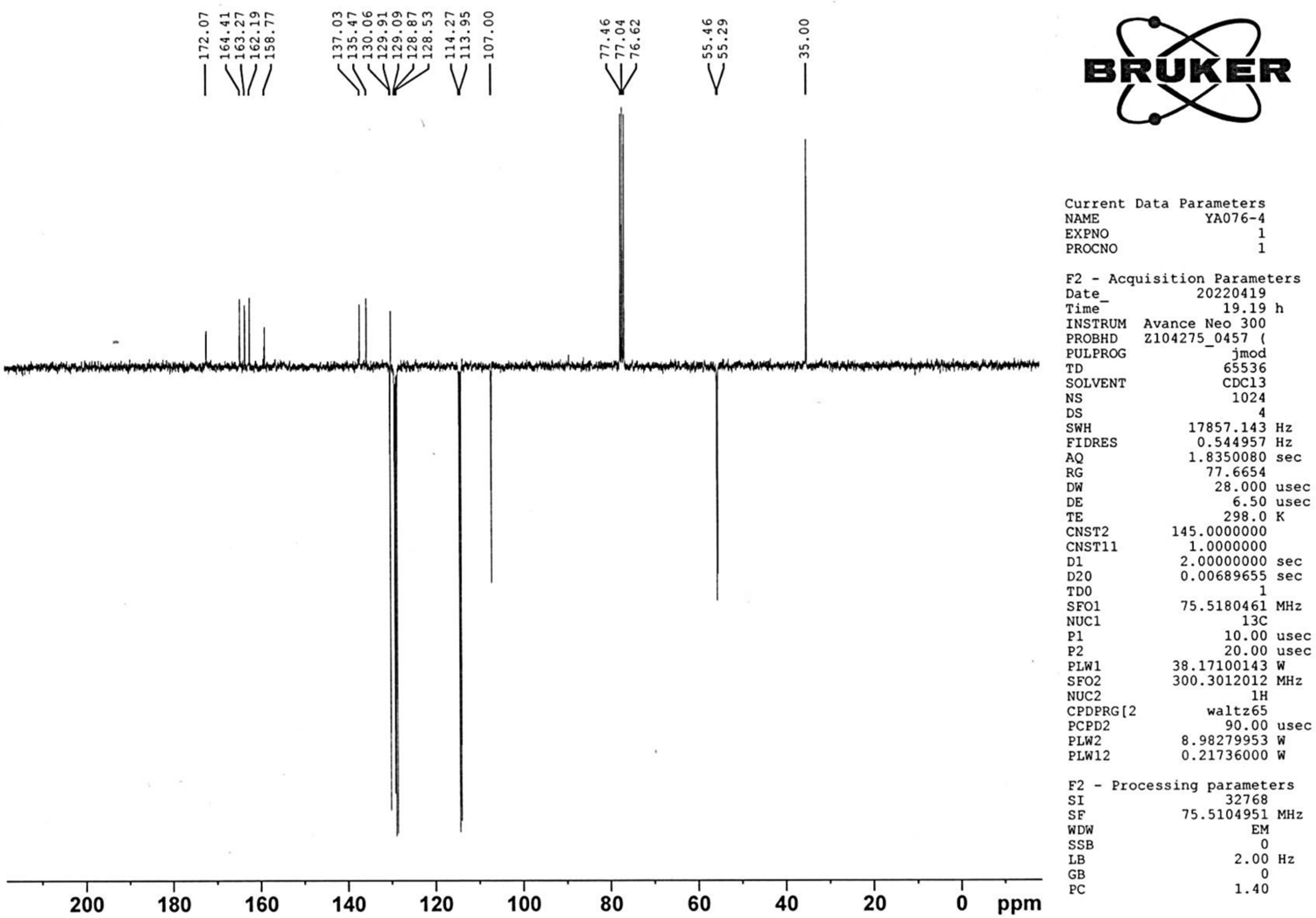

Figure S1.24. ^{13}C NMR of compound **15** ($CDCl_3$)

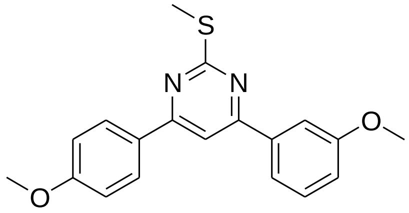

(16)

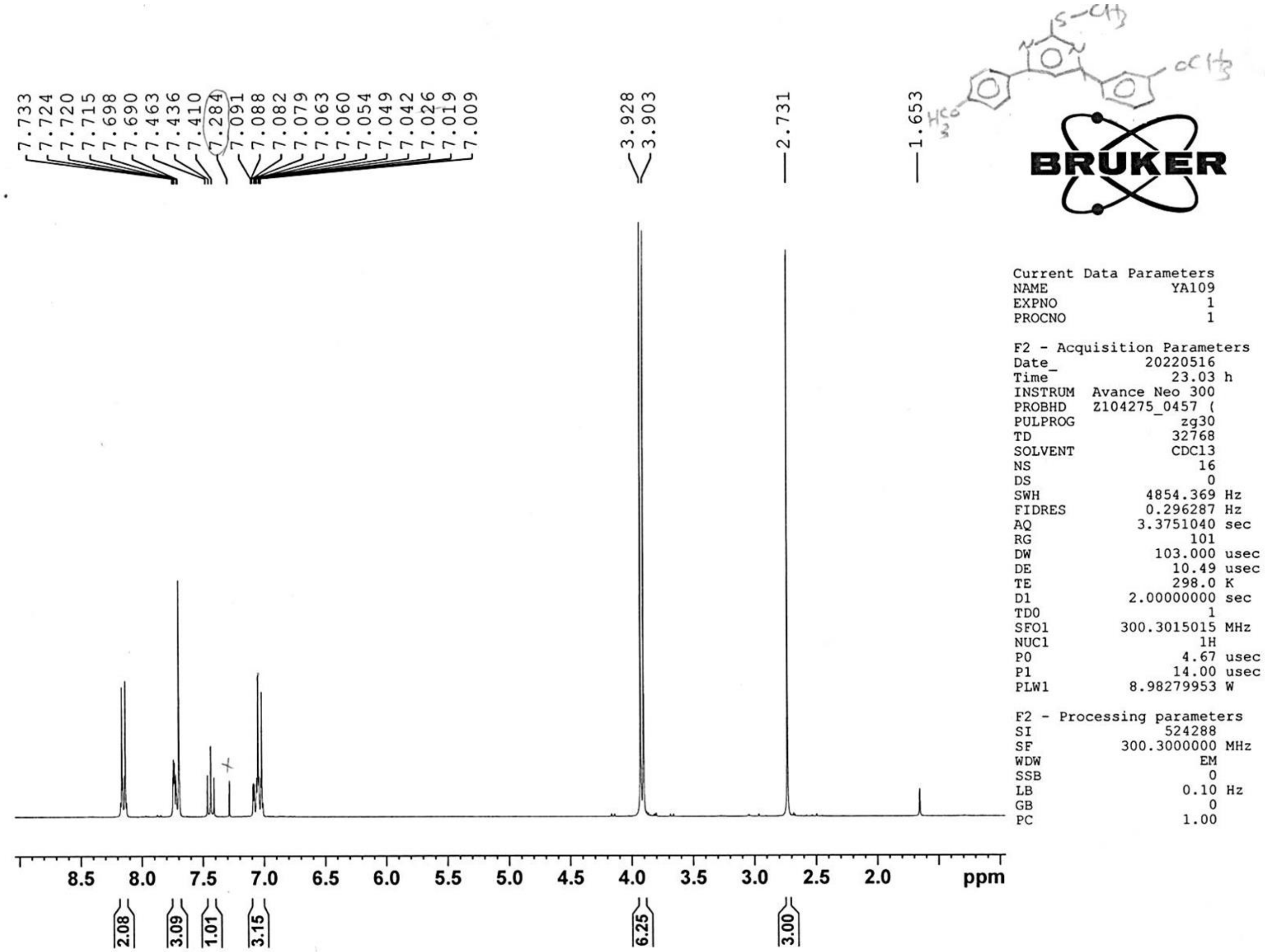

Figure S1.25. ^{1}H NMR of compound **16** ($CDCl_3$)

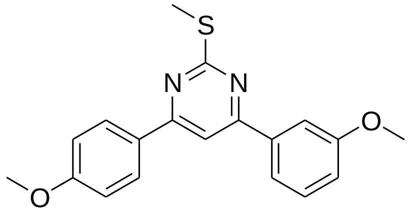

(16)

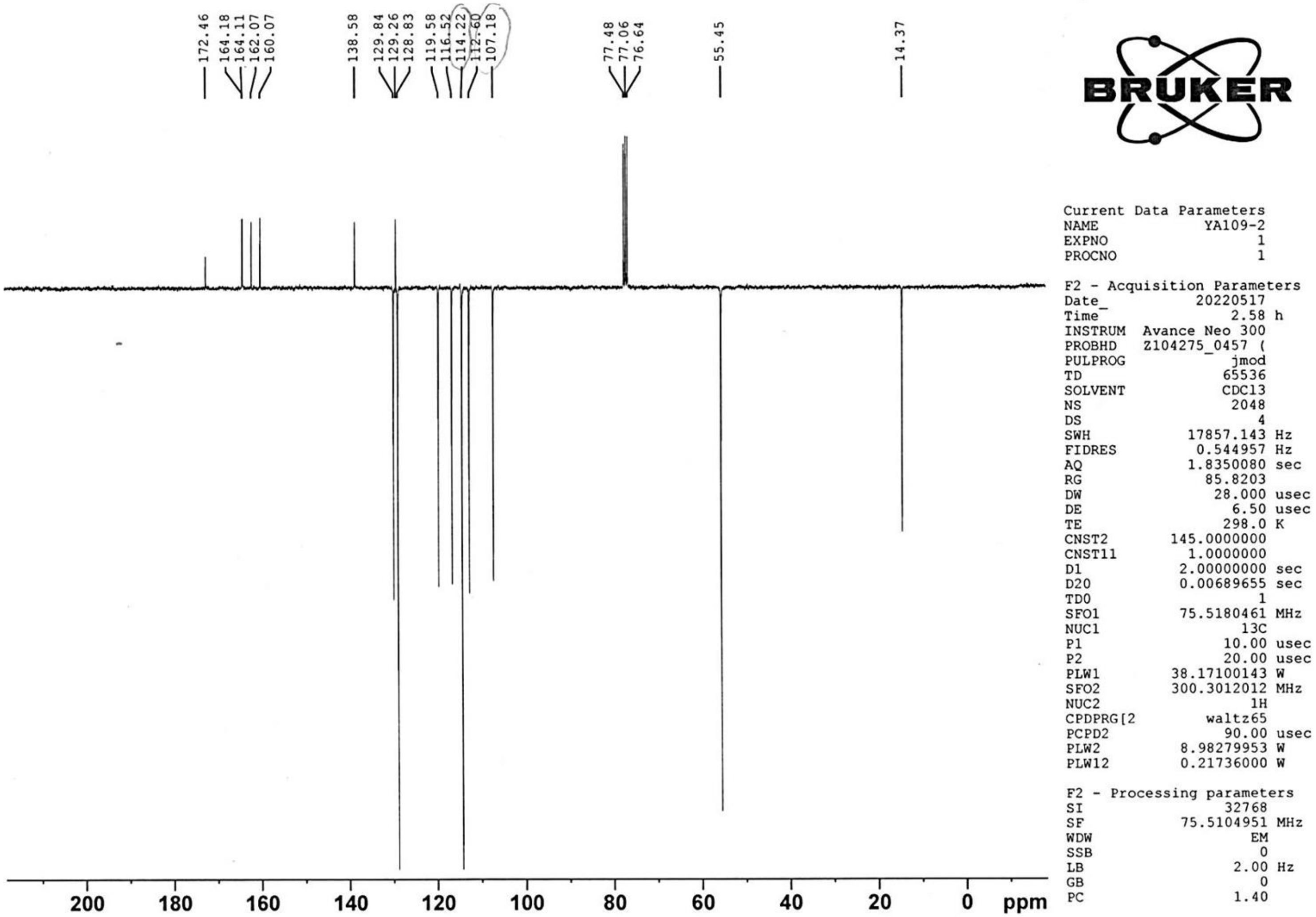

Figure S1.26. ^{13}C NMR of compound **16** ($CDCl_3$)

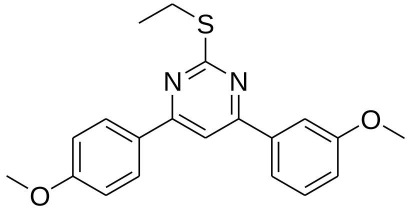

(17)

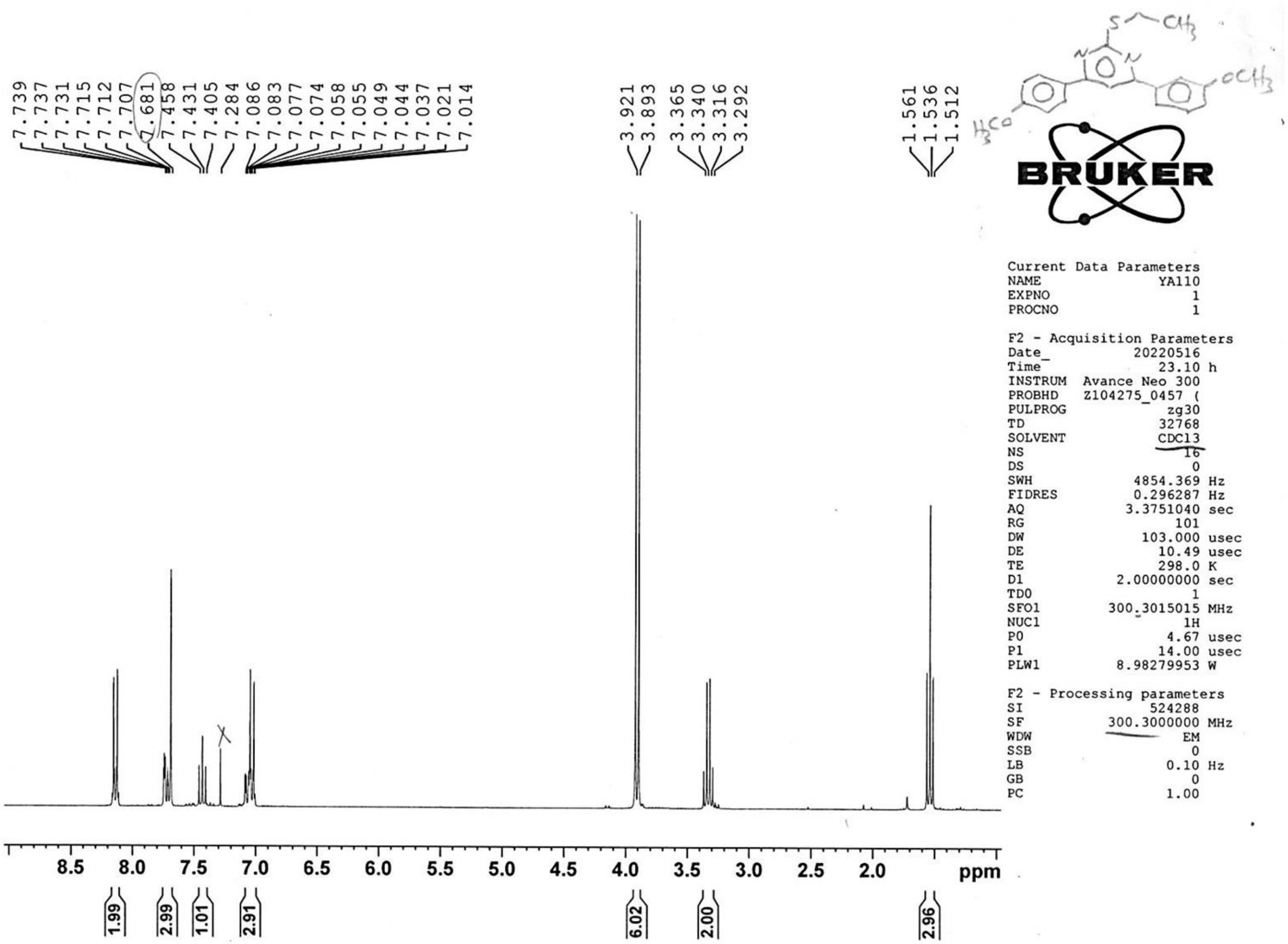

Figure S1.27. ^{1}H NMR of compound **17** ($CDCl_3$)

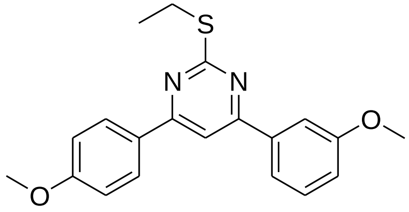

(17)

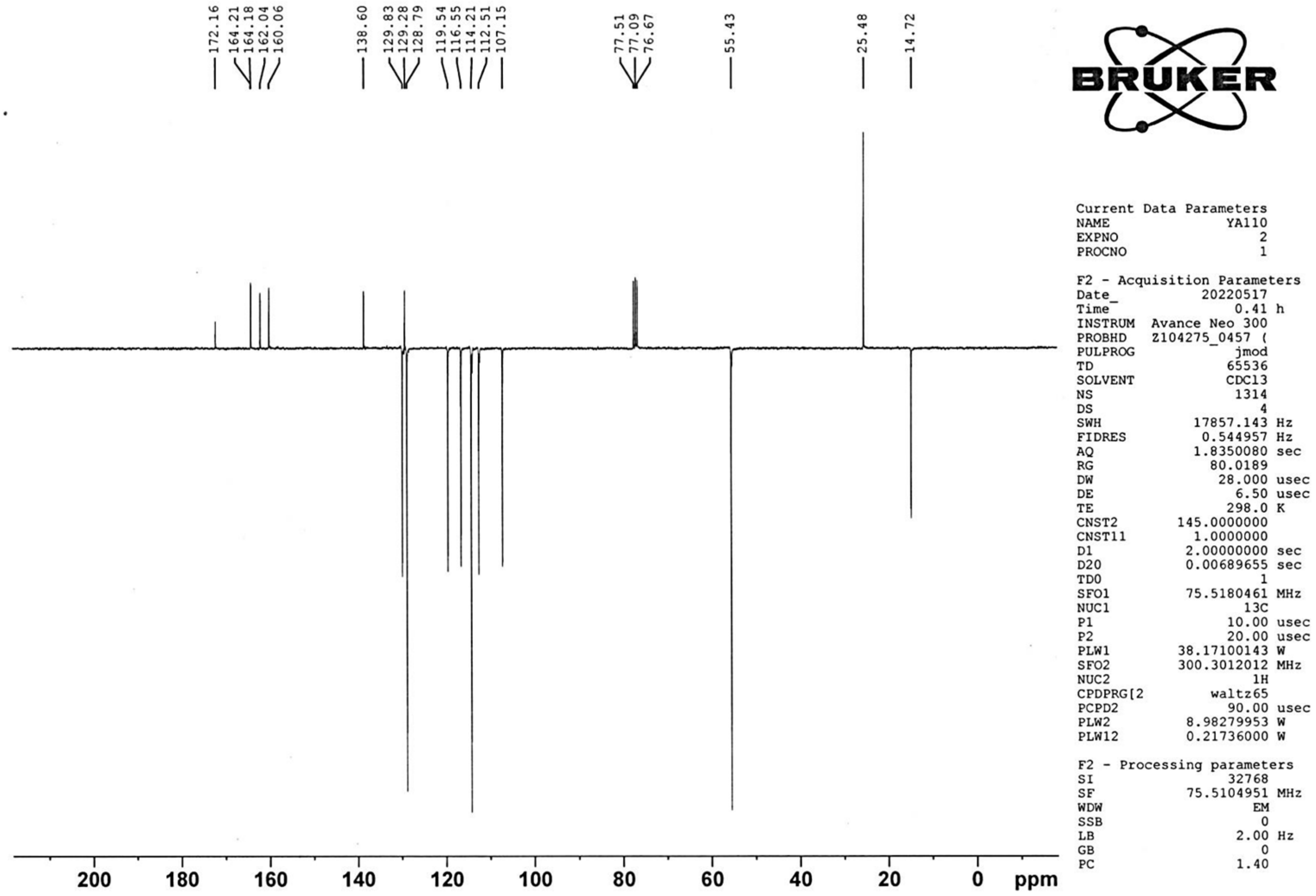

Figure S1.28. ^{13}C NMR of compound **17** ($CDCl_3$)

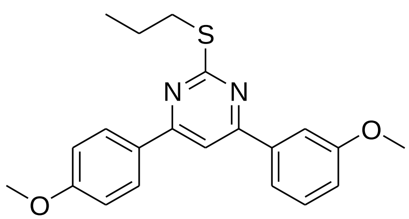

(18)

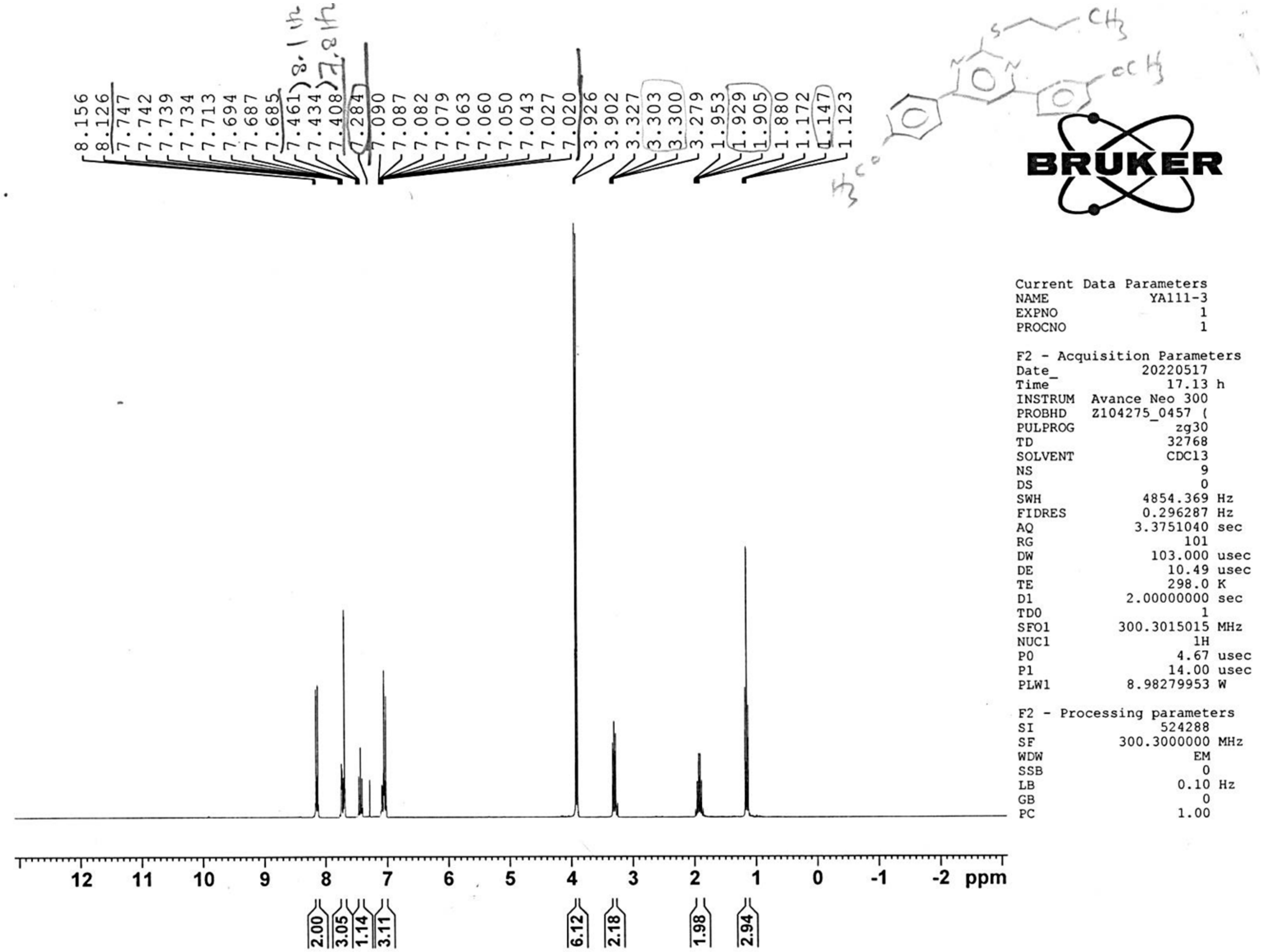

Figure S1.29. ^{1}H NMR of compound **18** ($CDCl_3$)

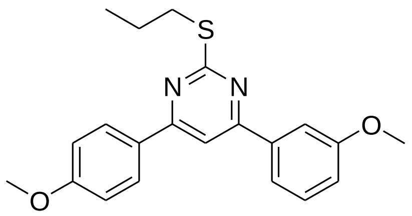

(18)

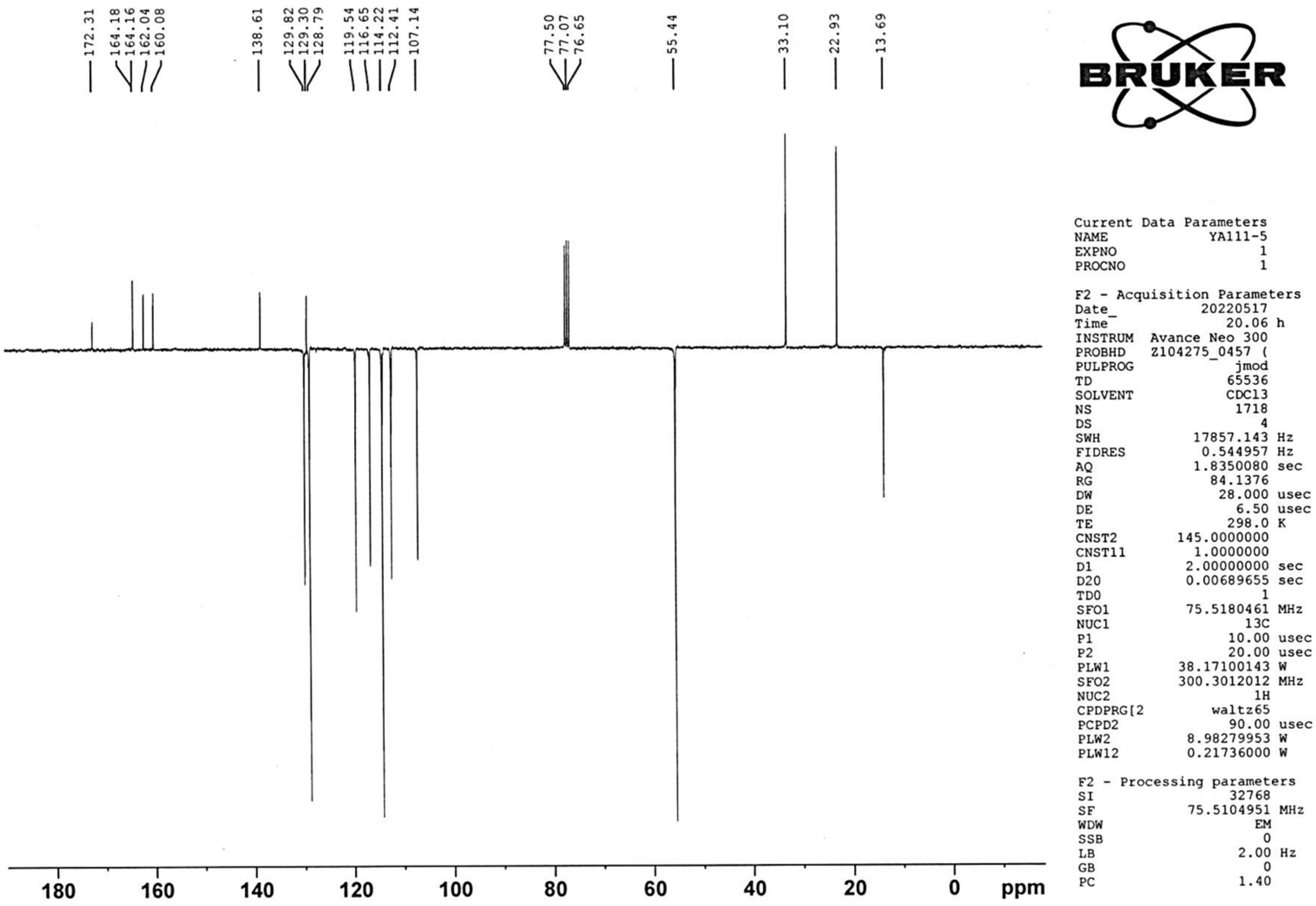

Figure S1.30. ^{13}C NMR of compound **18** ($CDCl_3$)

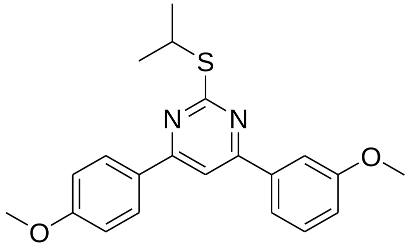

(19)

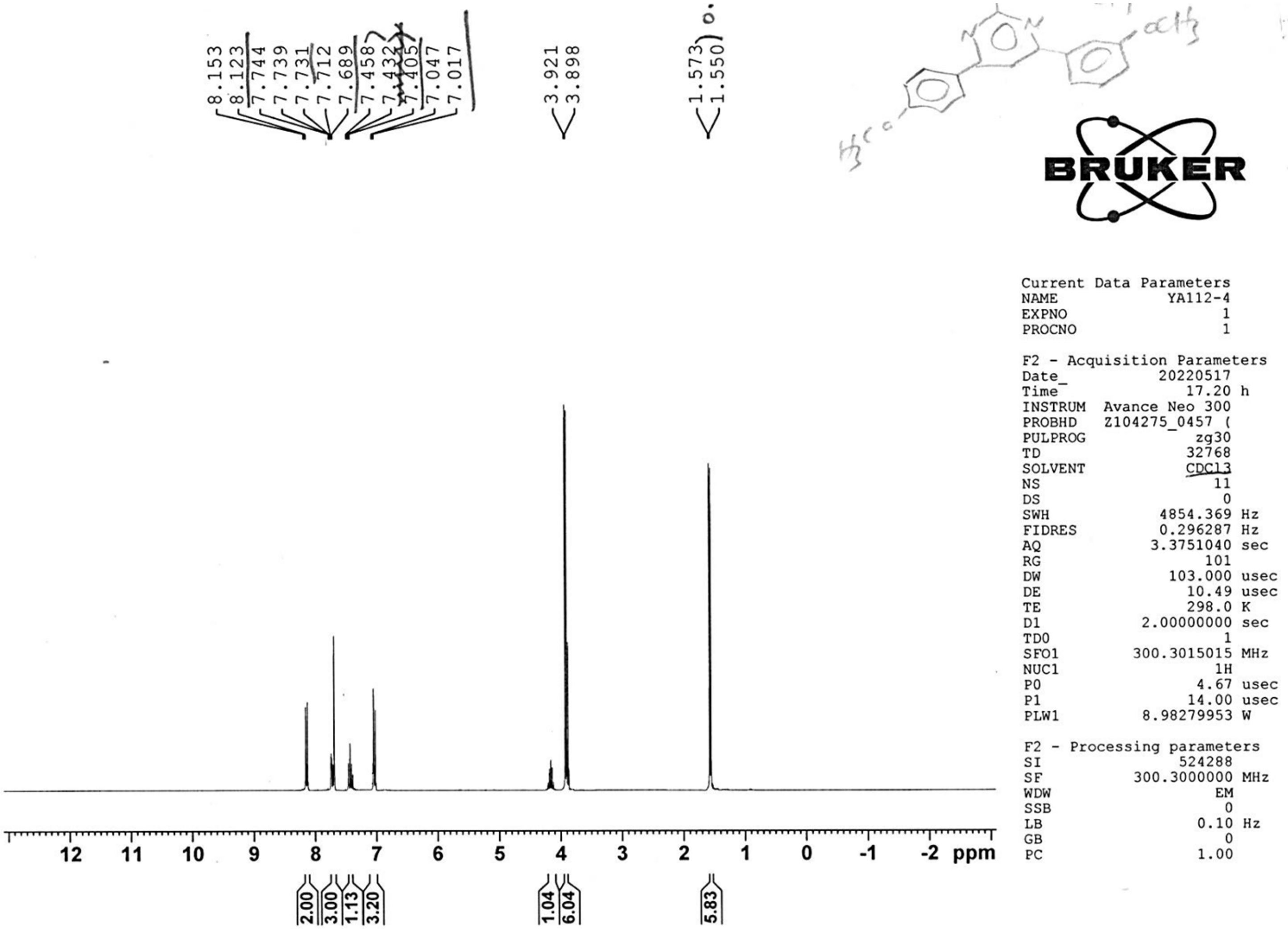

Figure S1.31. ^{1}H NMR of compound **19** ($CDCl_3$)

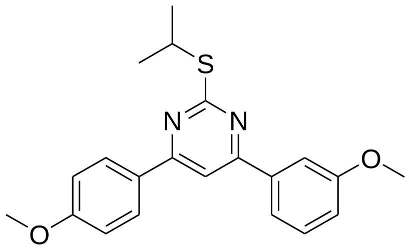

(19)

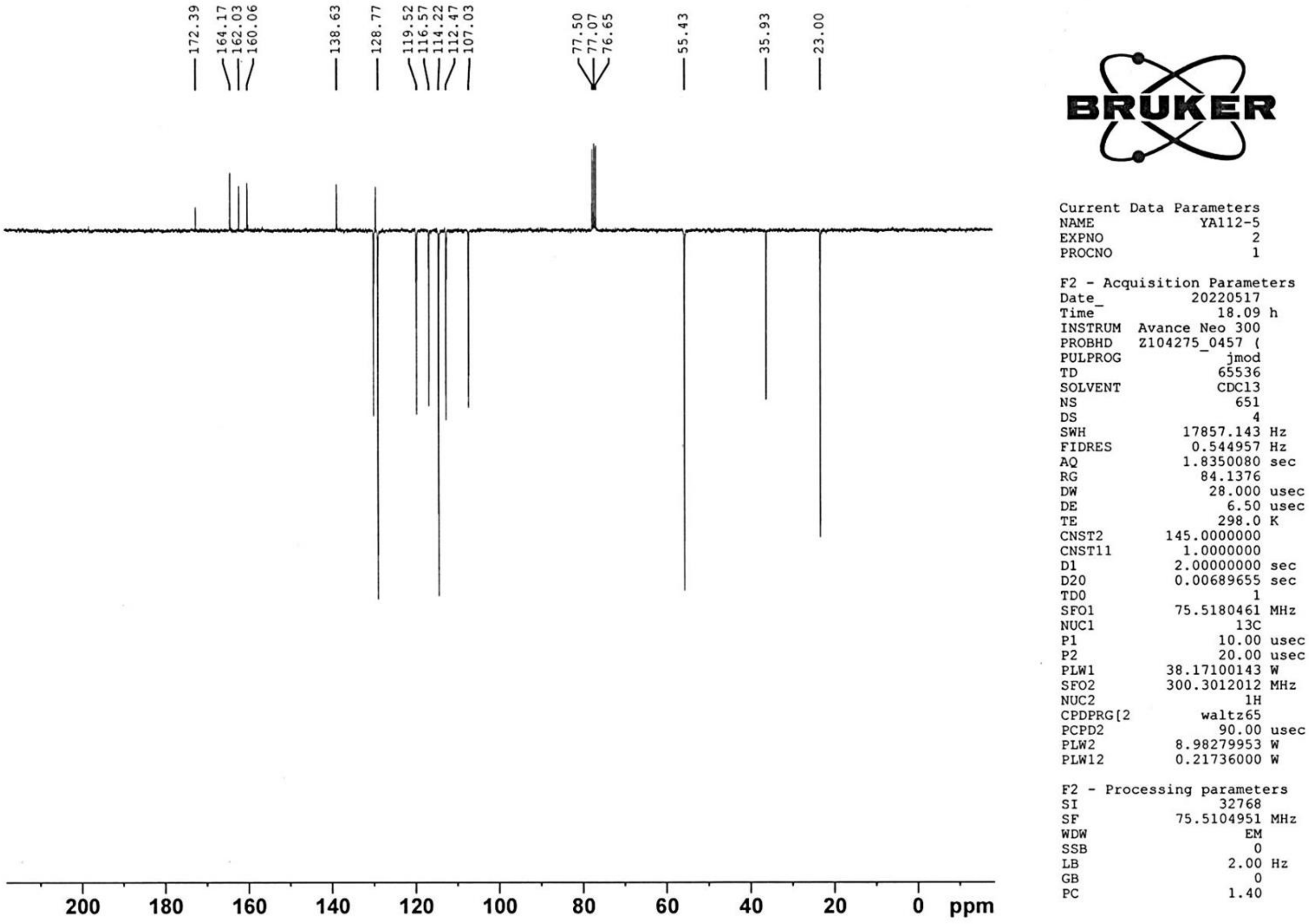

Figure S1.32. ^{13}C NMR of compound **19** ($CDCl_3$)

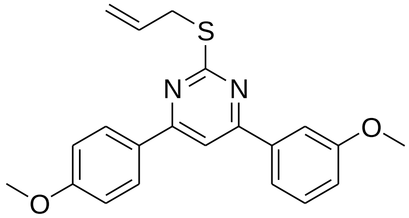

(20)

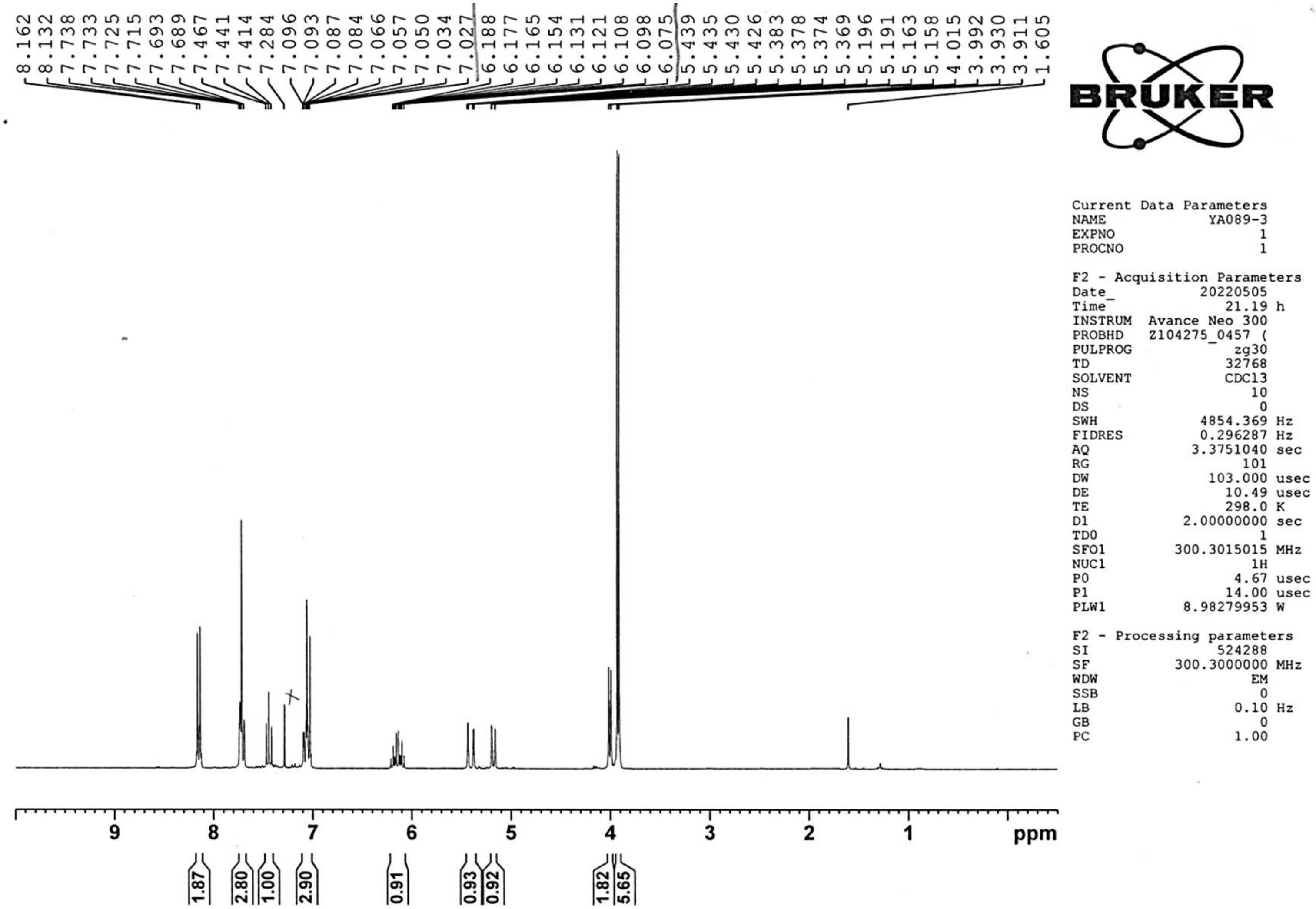

Figure S1.33. ^{1}H NMR of compound **20** ($CDCl_3$)

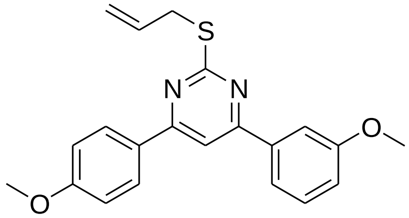

(20)

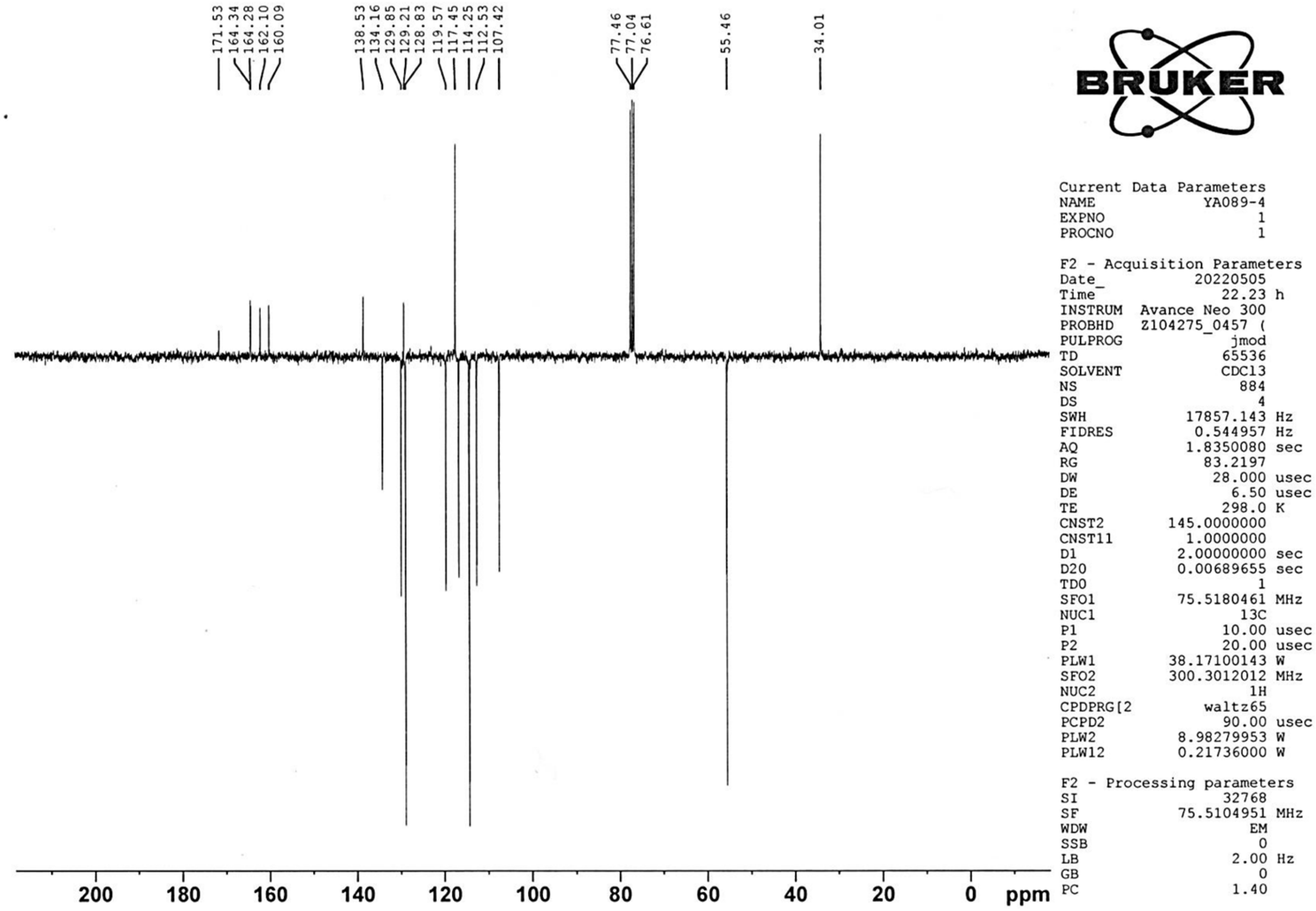

Figure S1.34. ^{13}C NMR of compound **20** ($CDCl_3$)

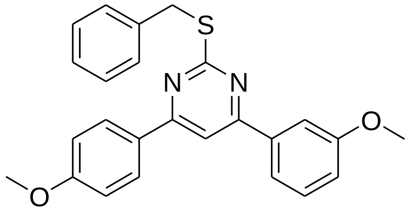

(21)

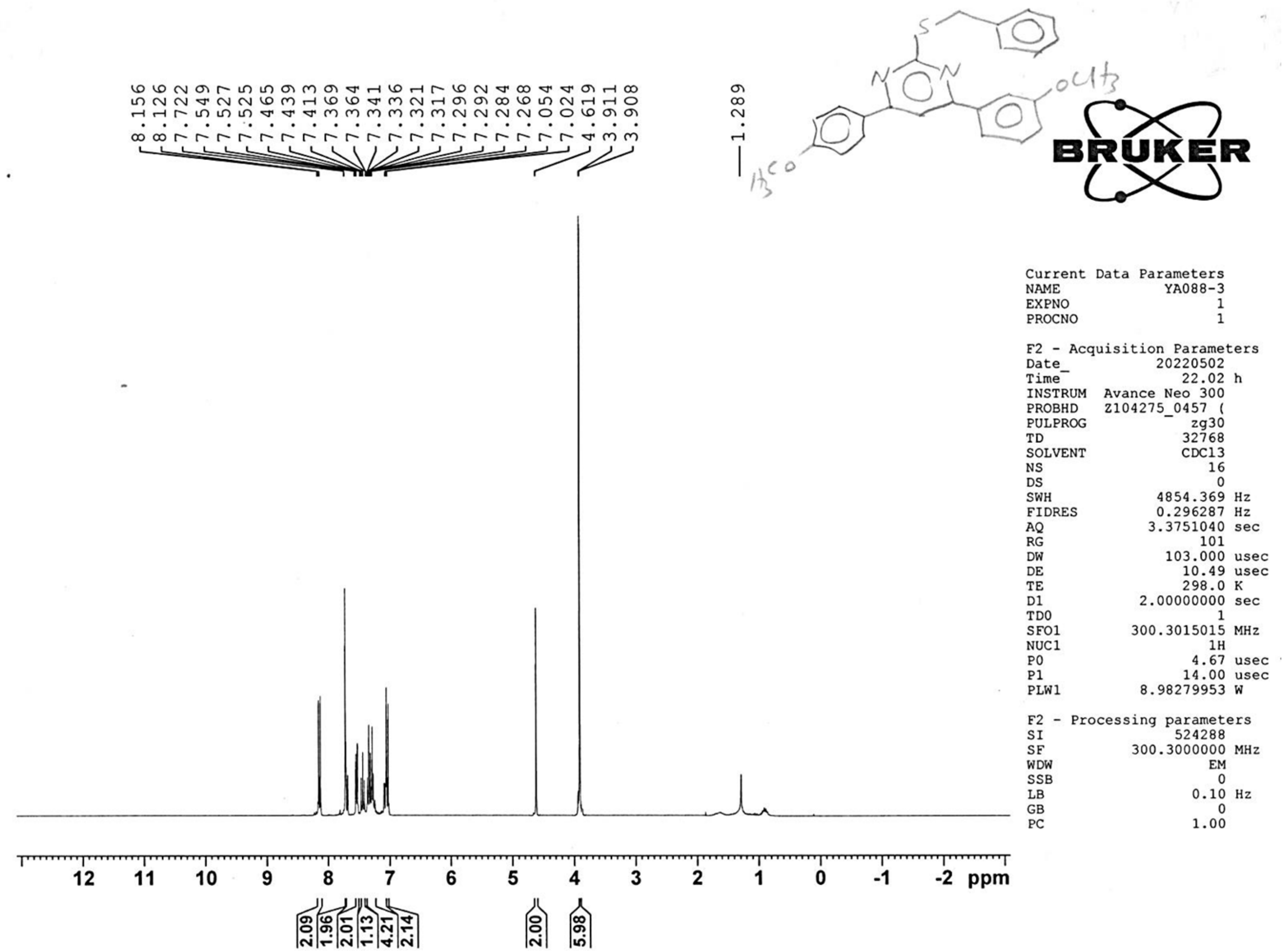

Figure S1.35. ^{1}H NMR of compound **21** ($CDCl_3$)

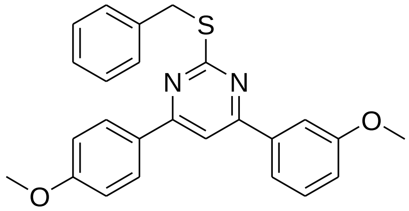

(21)

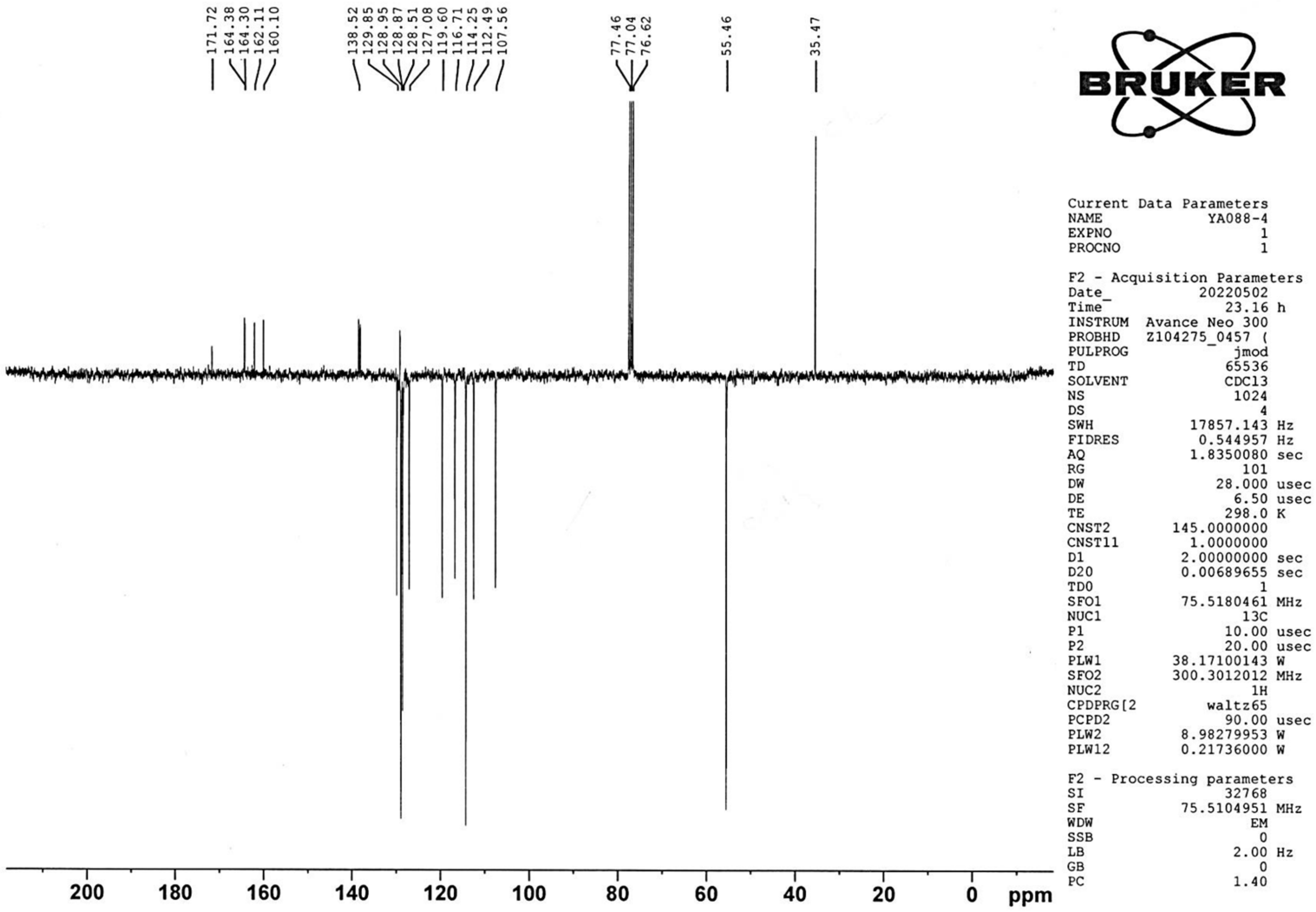

Figure S1.36. ^{13}C NMR of compound **21** ($CDCl_3$)

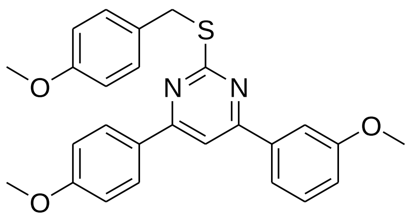

(22)

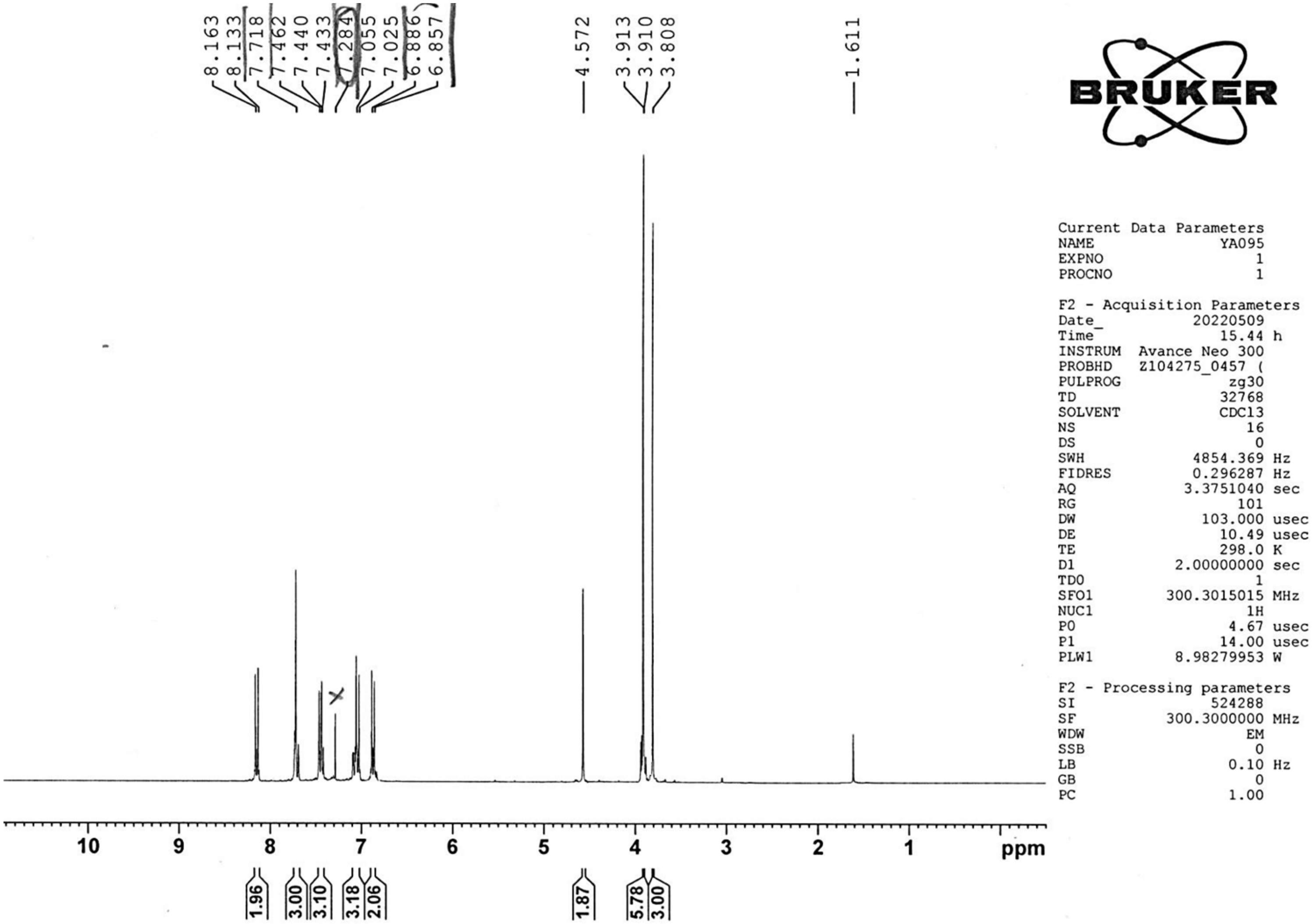

Figure S1.37. ^{1}H NMR of compound **22** ($CDCl_3$)

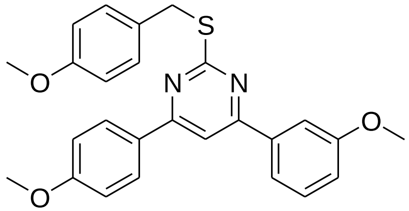

(22)

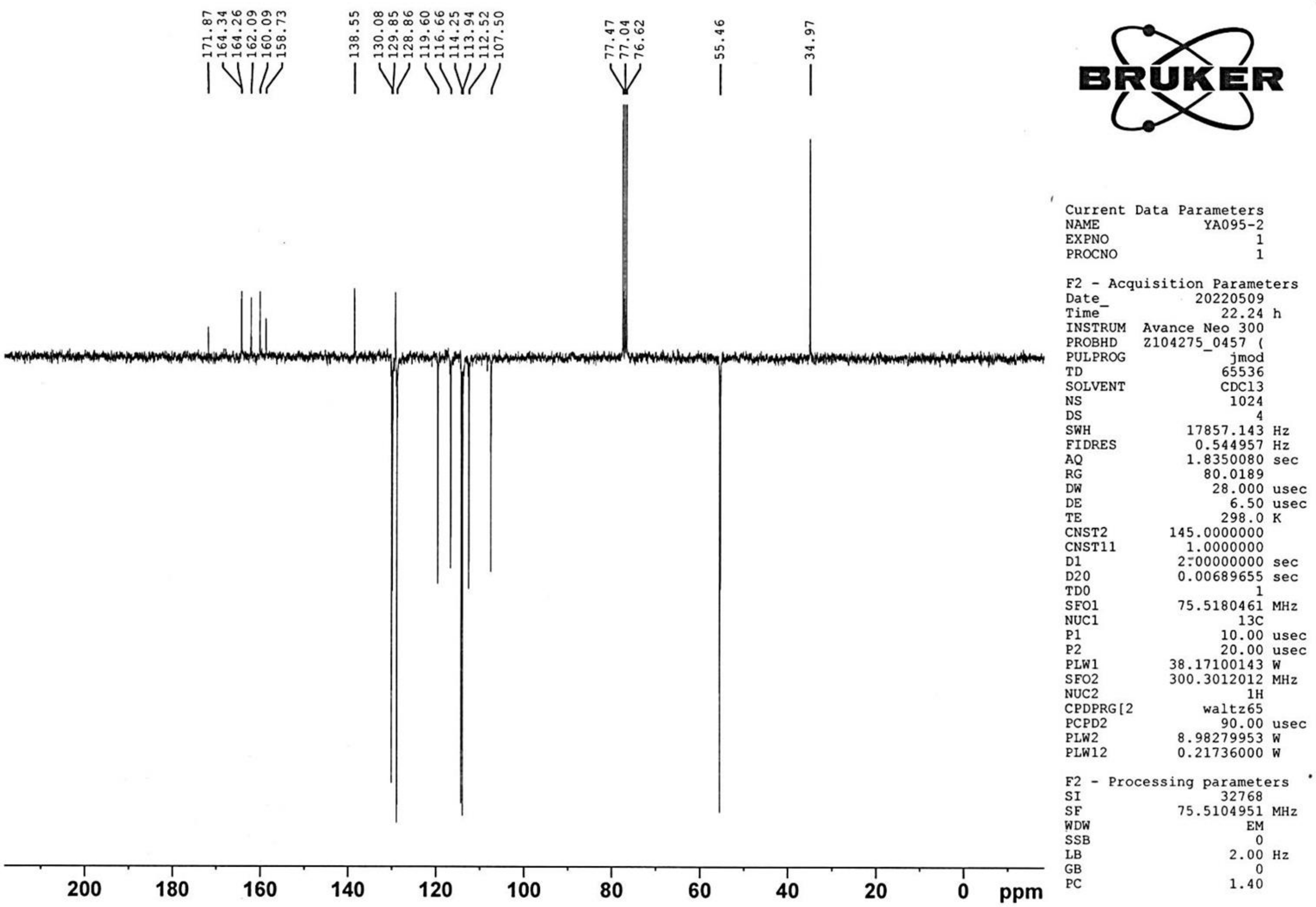

Figure S1.38. ^{13}C NMR of compound **22** ($CDCl_3$)

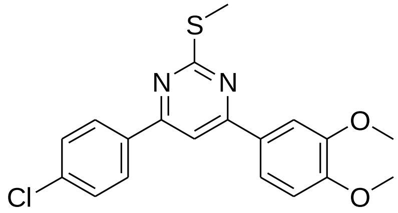

(23)

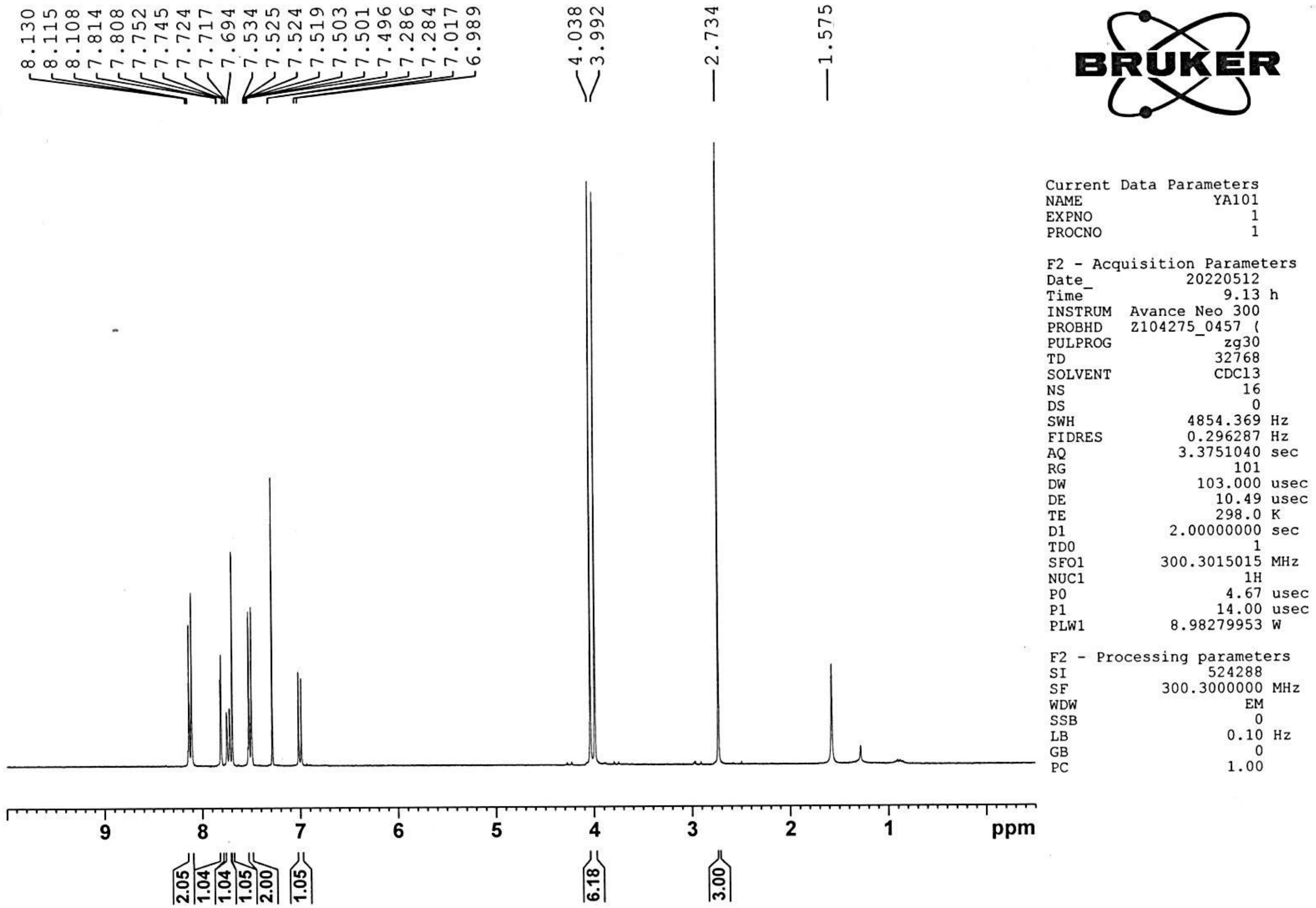

Figure S1.39. ^{1}H NMR of compound **23** ($CDCl_3$)

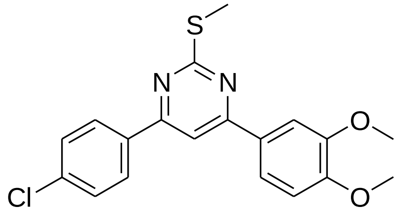

(23)

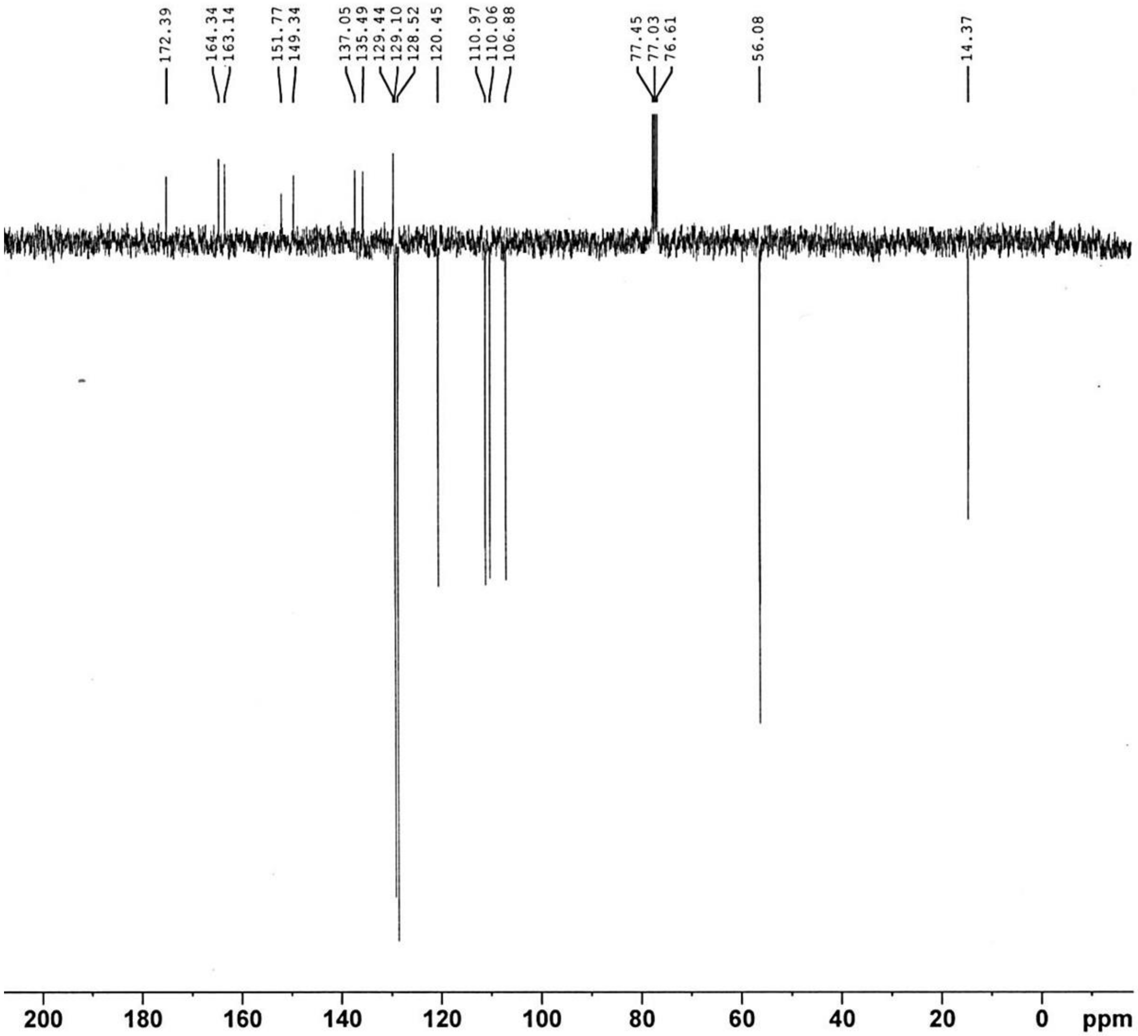

Current Data Parameters		
NAME	YA101-3	
EXPNO	1	
PROCNO	1	
F2 - Acquisition Parameters		
Date_	20220512	
Time	18.00	h
INSTRUM	Avance Neo 300	
PROBHD	Z104275_0457 (	
PULPROG	jmod	
TD	65536	
SOLVENT	CDCl3	
NS	879	
DS	4	
SWH	17857.143	Hz
FIDRES	0.544957	Hz
AQ	1.8350080	sec
RG	85.8203	
DW	28.000	usec
DE	6.50	usec
TE	298.0	K
CNST2	145.0000000	
CNST11	1.0000000	
D1	2.00000000	sec
D20	0.00689655	sec
TD0	1	
SFO1	75.5180461	MHz
NUC1	13C	
P1	10.00	usec
P2	20.00	usec
PLW1	38.17100143	W
SFO2	300.3012012	MHz
NUC2	1H	
CPDPRG[2	waltz65	
PCPD2	90.00	usec
PLW2	8.98279953	W
PLW12	0.21736000	W
F2 - Processing parameters		
SI	32768	
SF	75.5104951	MHz
WDW	EM	
SSB	0	
LB	2.00	Hz
GB	0	
PC	1.40	

Figure S1.40. ^{13}C NMR of compound **23** ($CDCl_3$)

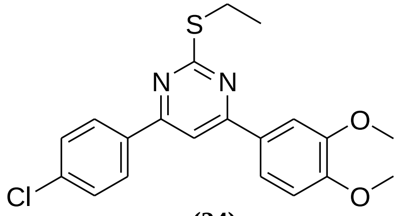

(24)

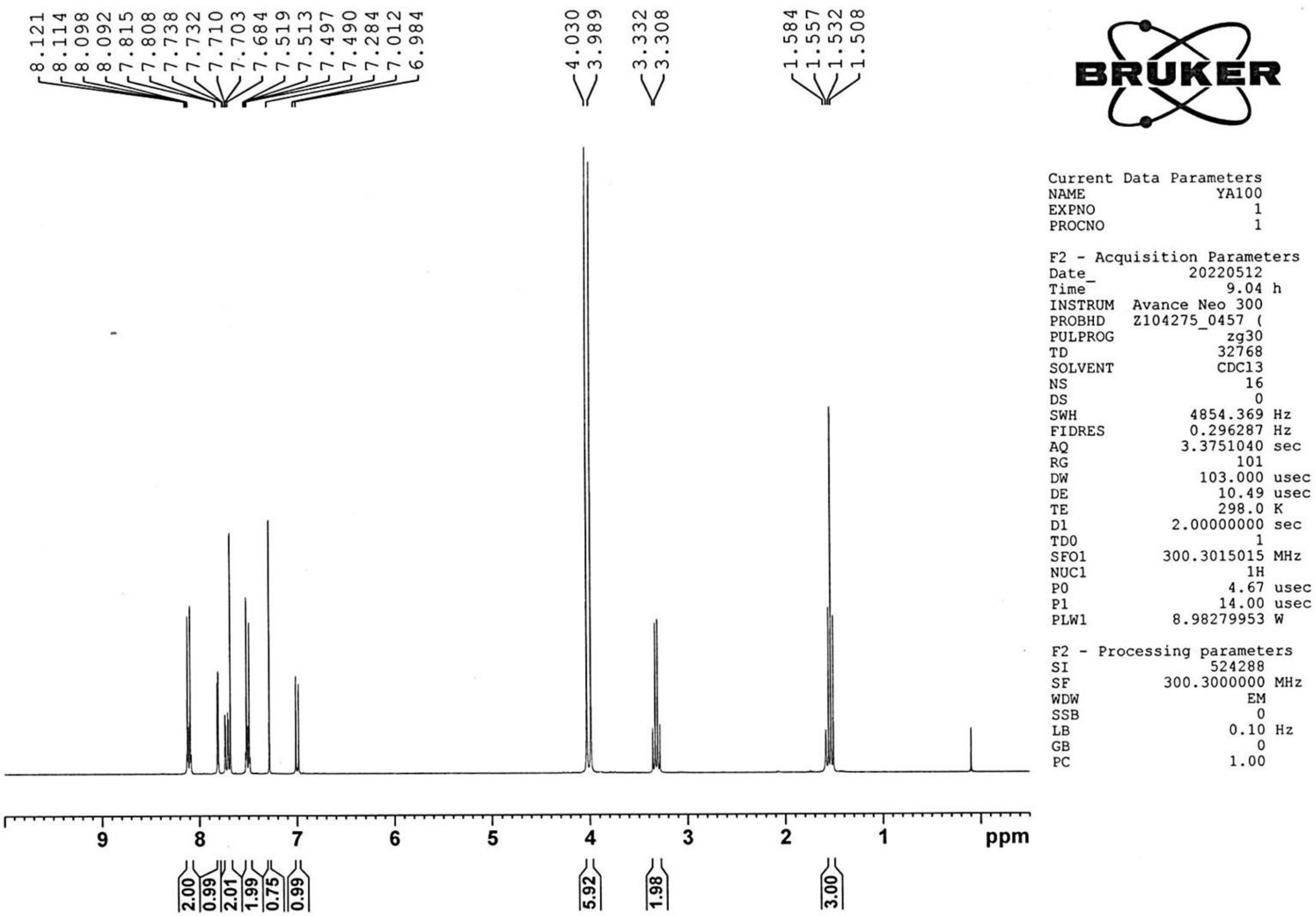

Figure S1.41. ^{1}H NMR of compound **24** ($CDCl_3$)

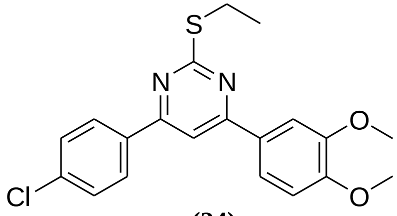

(24)

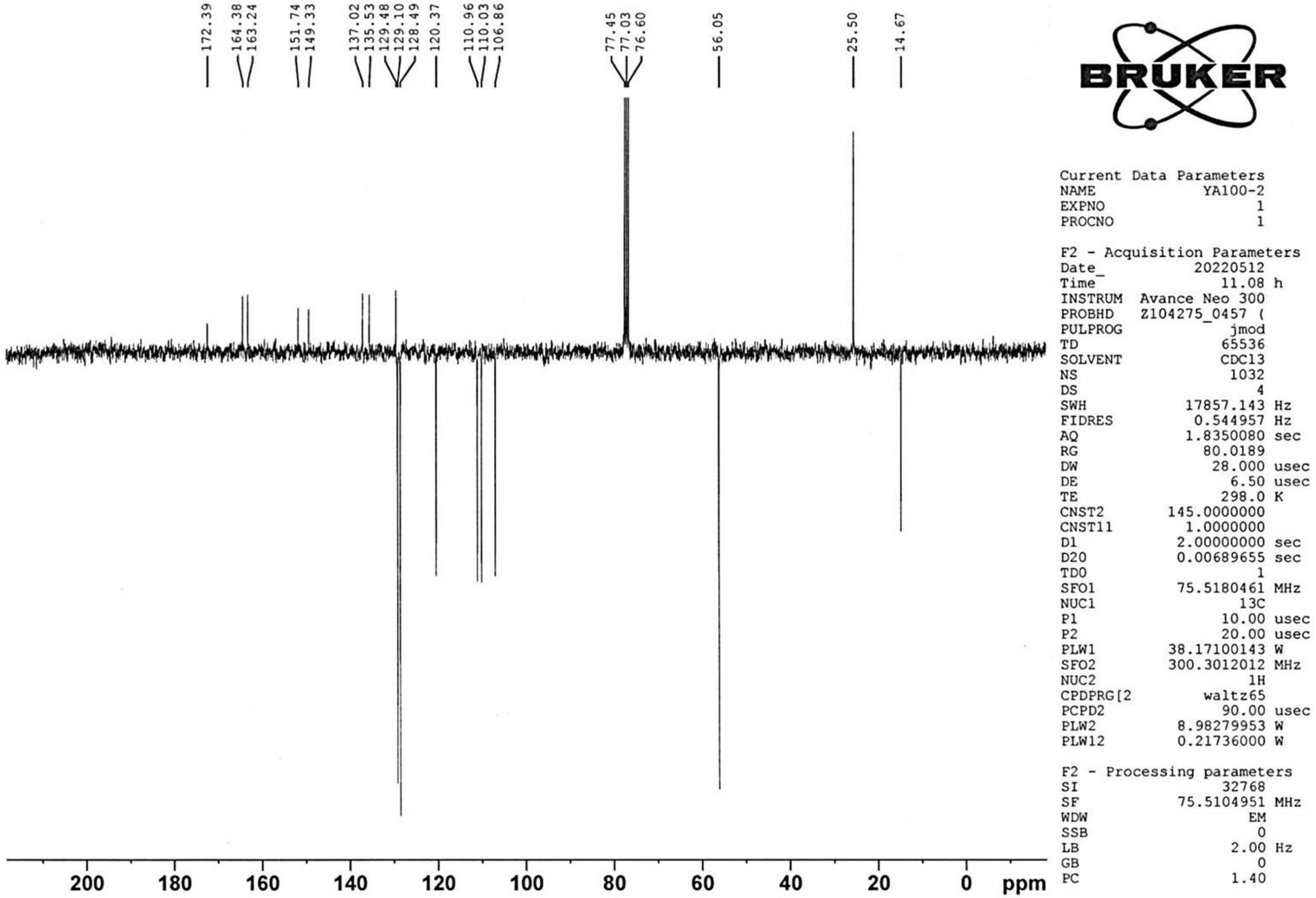

Figure S1.42. ^{13}C NMR of compound **24** ($CDCl_3$)

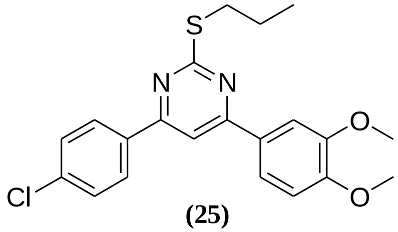

(25)

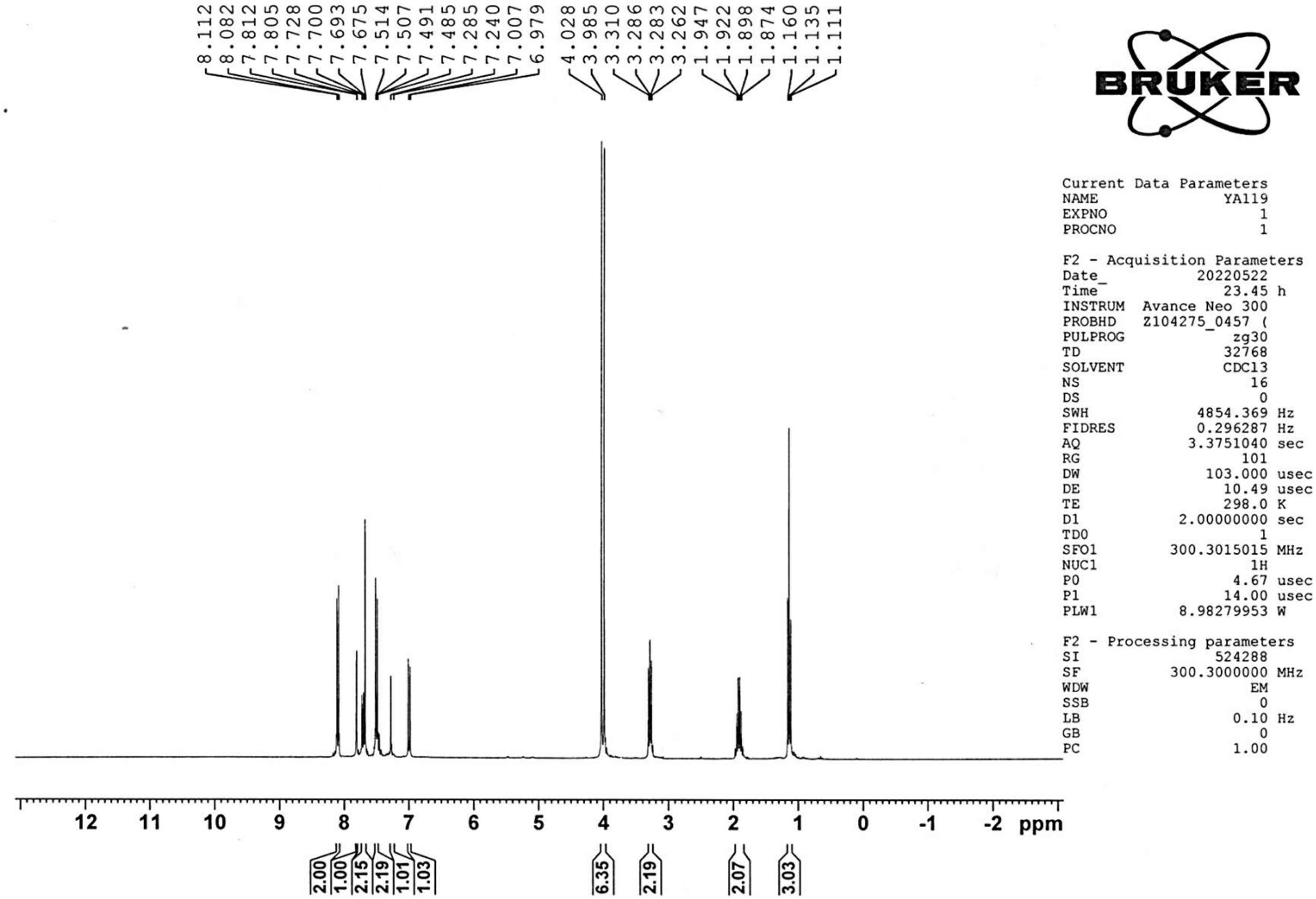

Figure S1.43. ^{1}H NMR of compound **25** ($CDCl_3$)

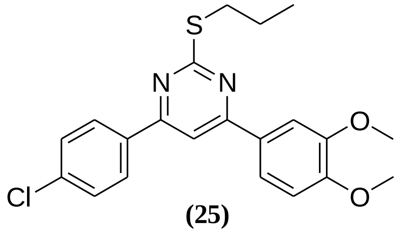

(25)

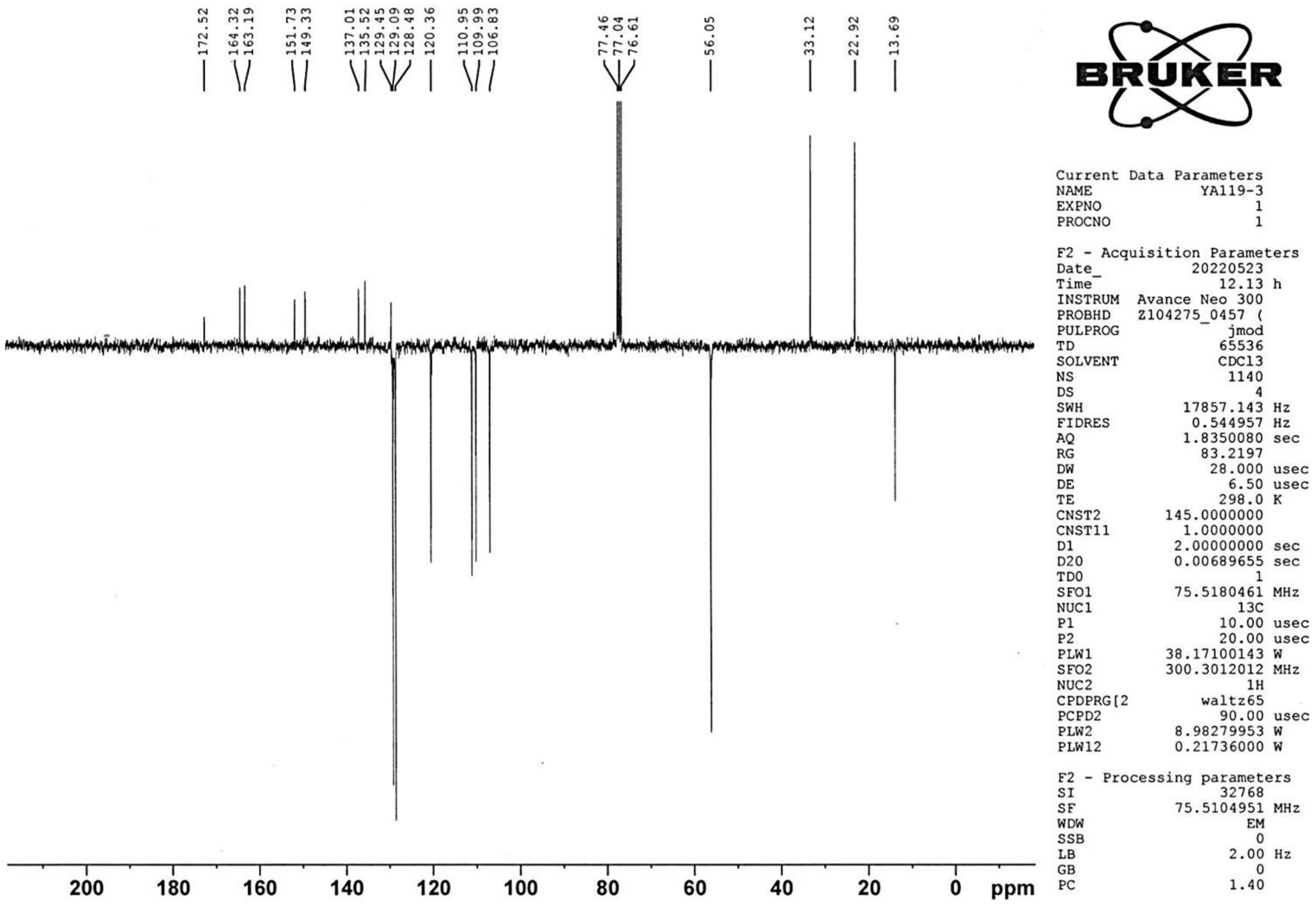

Figure S1.44. ^{13}C NMR of compound **25** ($CDCl_3$)

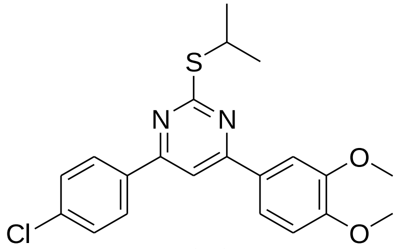

(26)

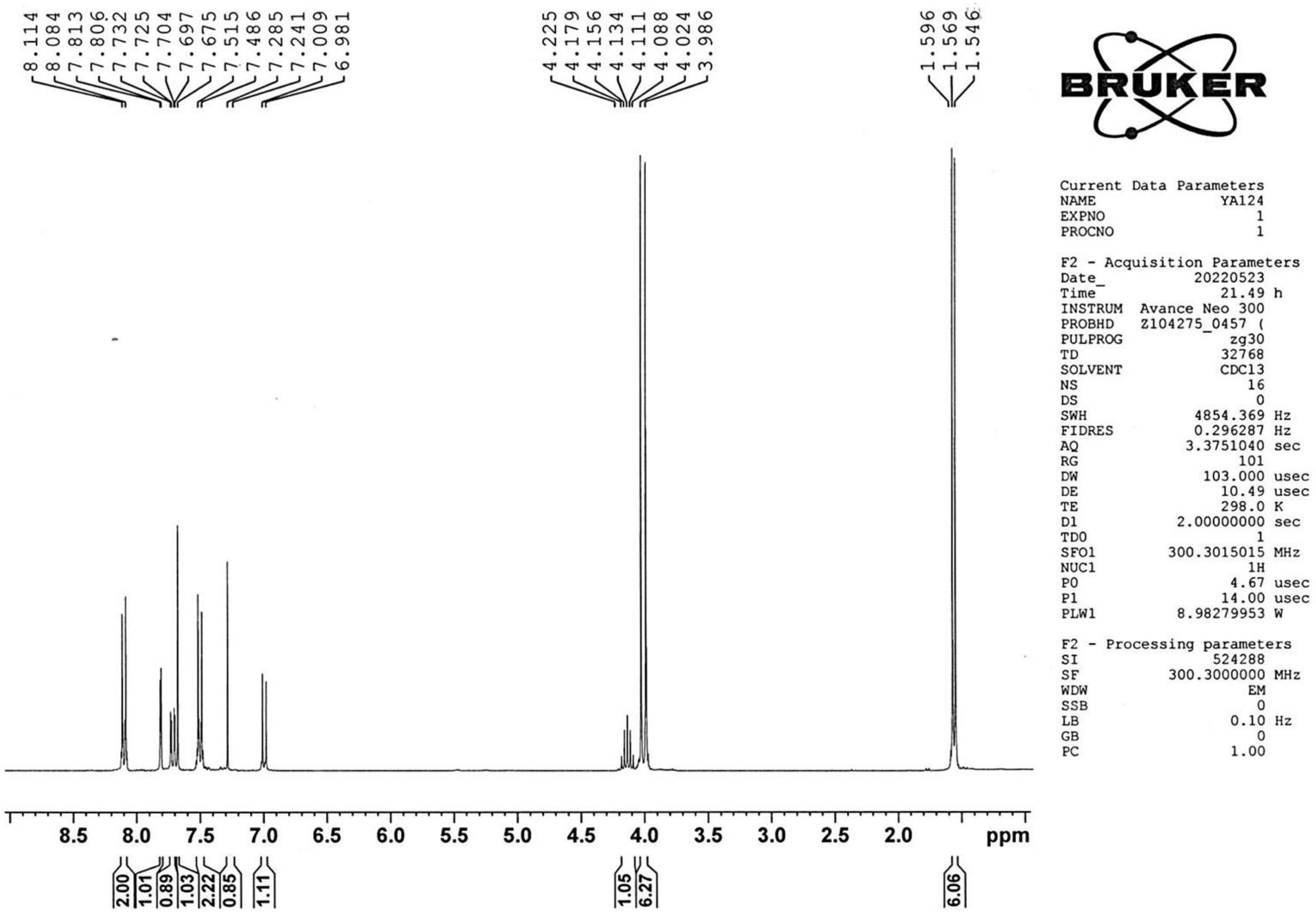

Figure S1.45. ^{1}H NMR of compound **26** ($CDCl_3$)

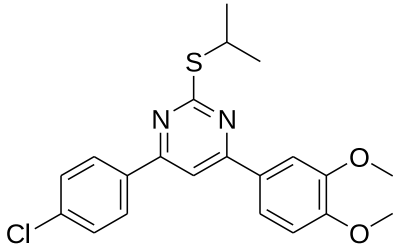

(26)

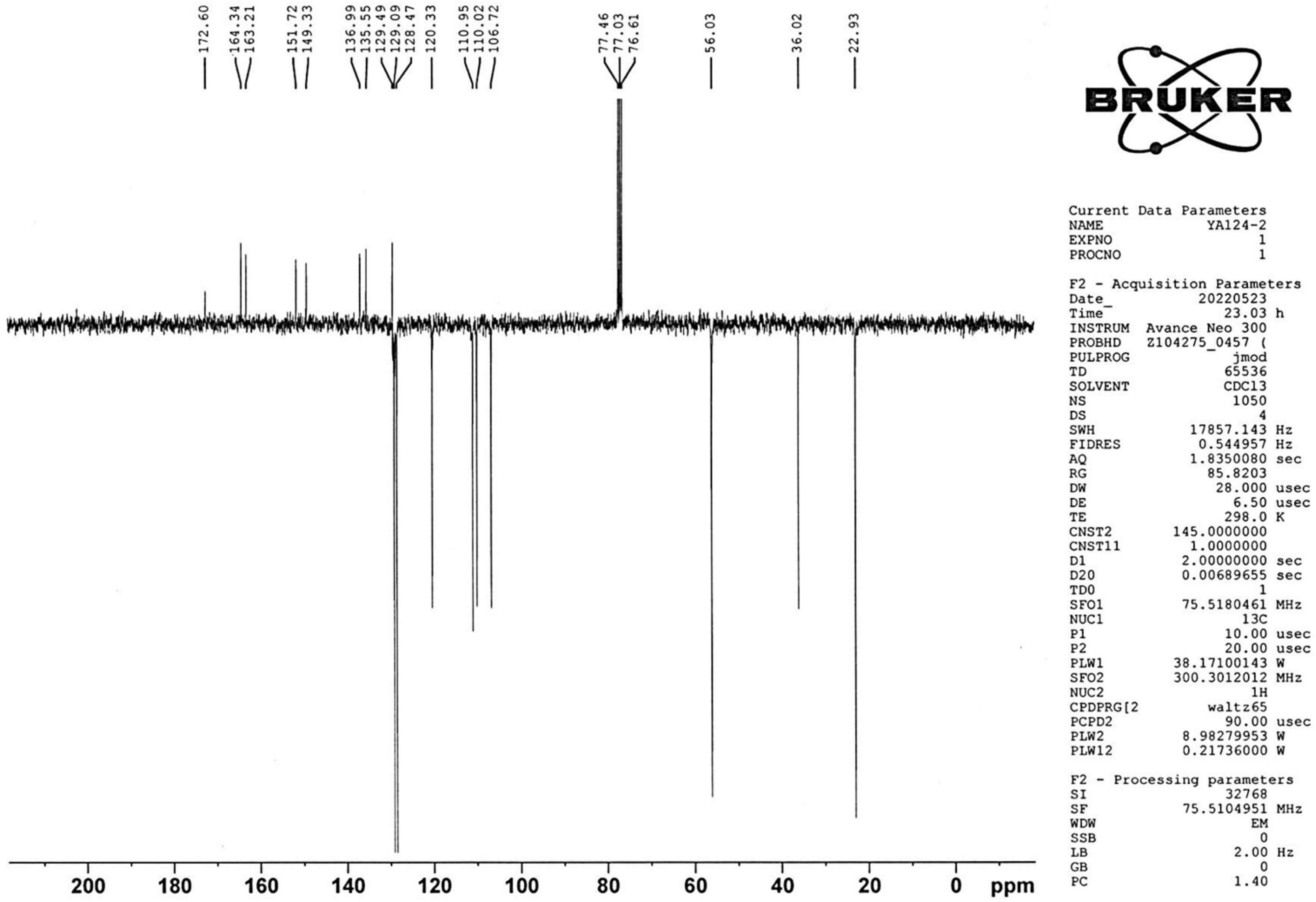

Figure S1.46. ^{13}C NMR of compound **26** ($CDCl_3$)

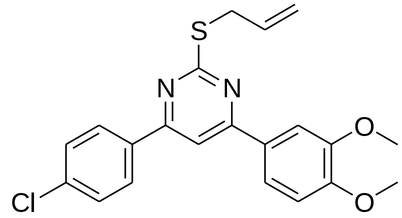

(27)

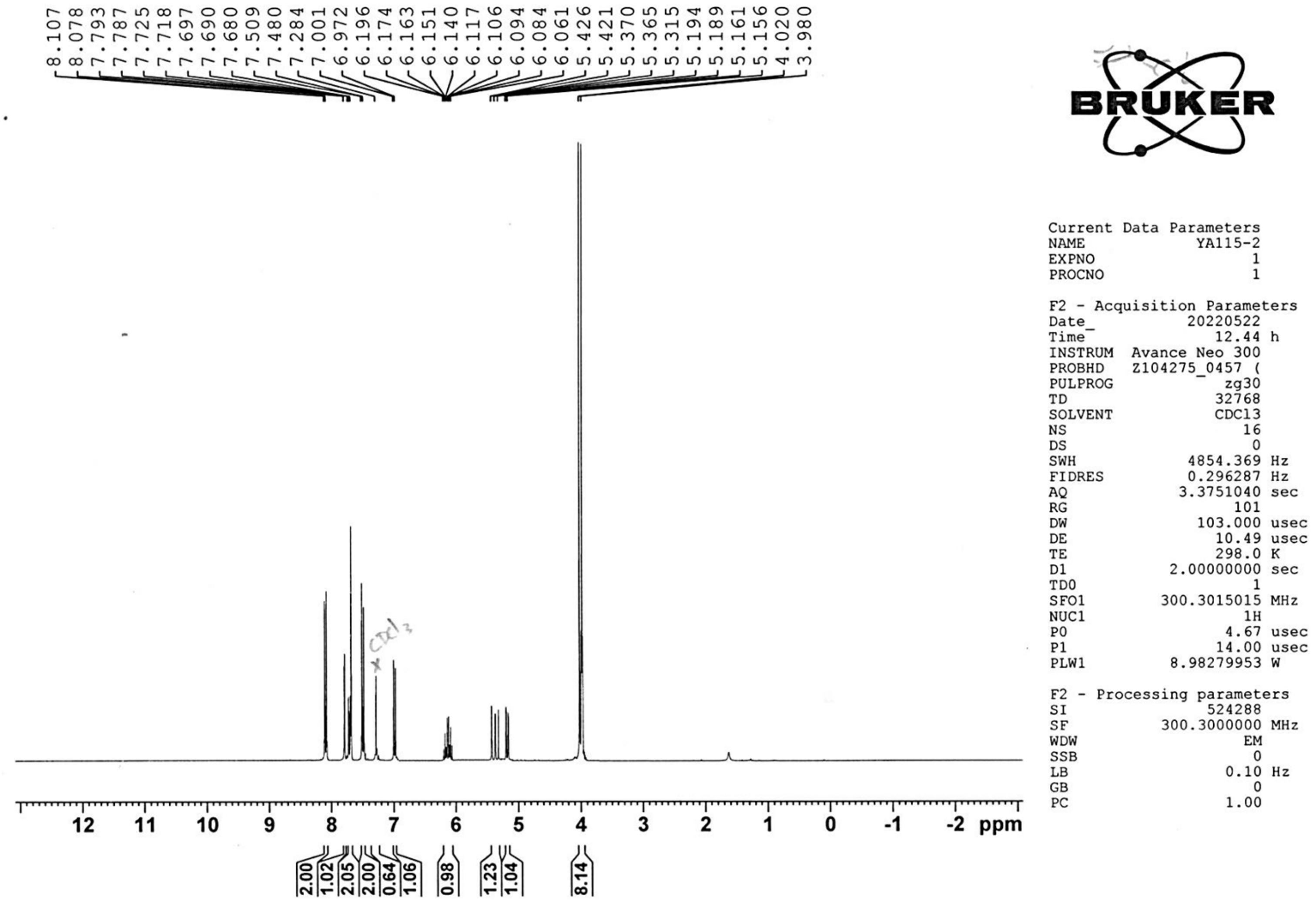

Figure S1.47. ^{1}H NMR of compound **27** ($CDCl_3$)

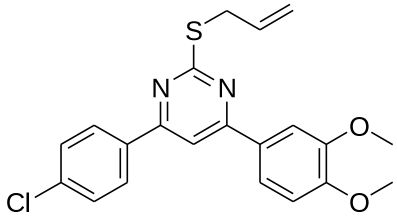

(27)

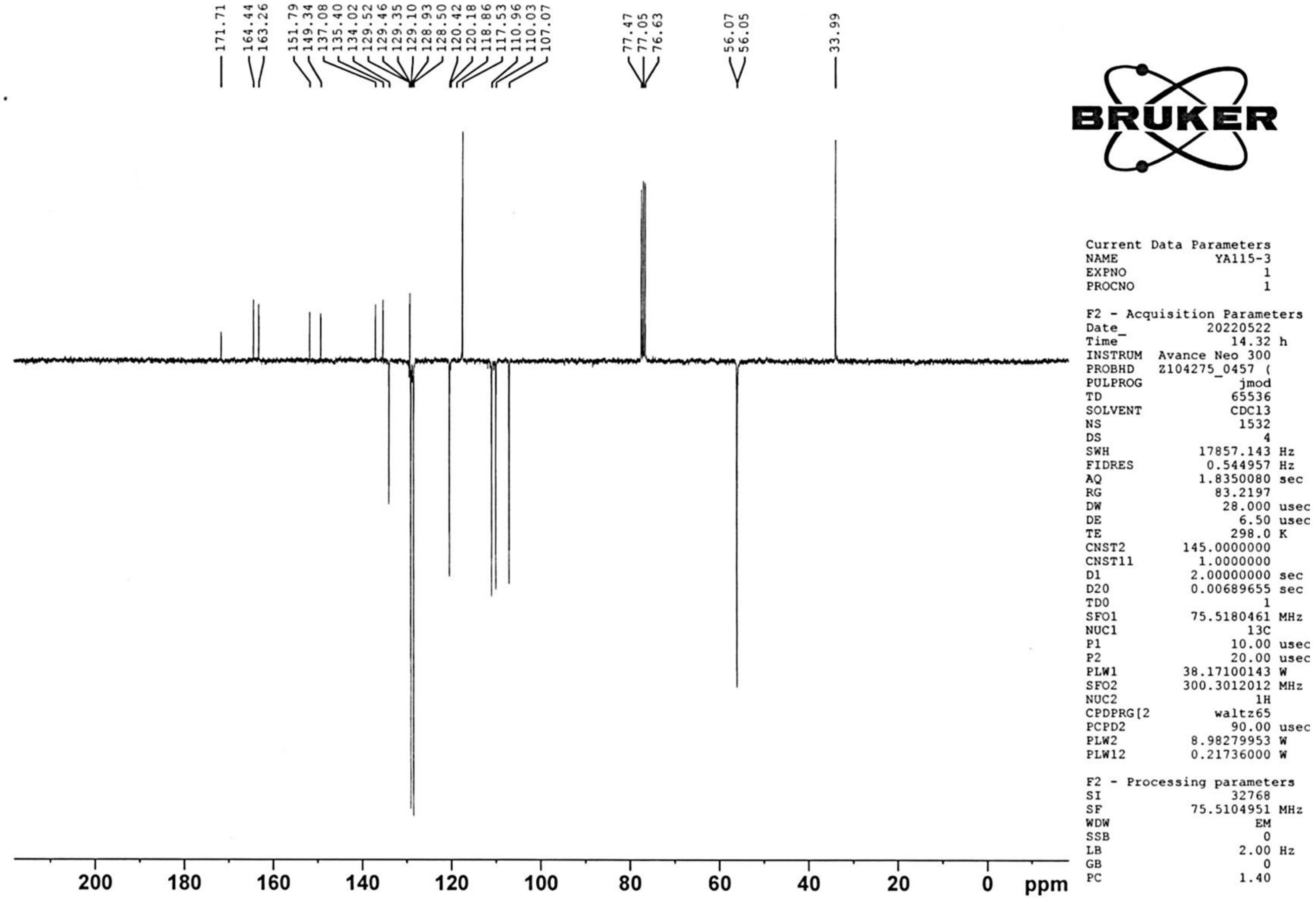

Figure S1.48. ^{13}C NMR of compound **27** ($CDCl_3$)

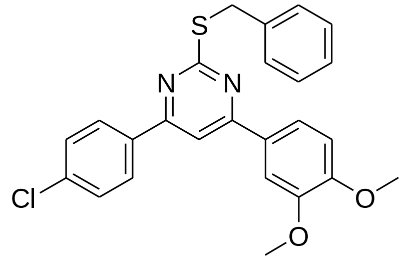

(28)

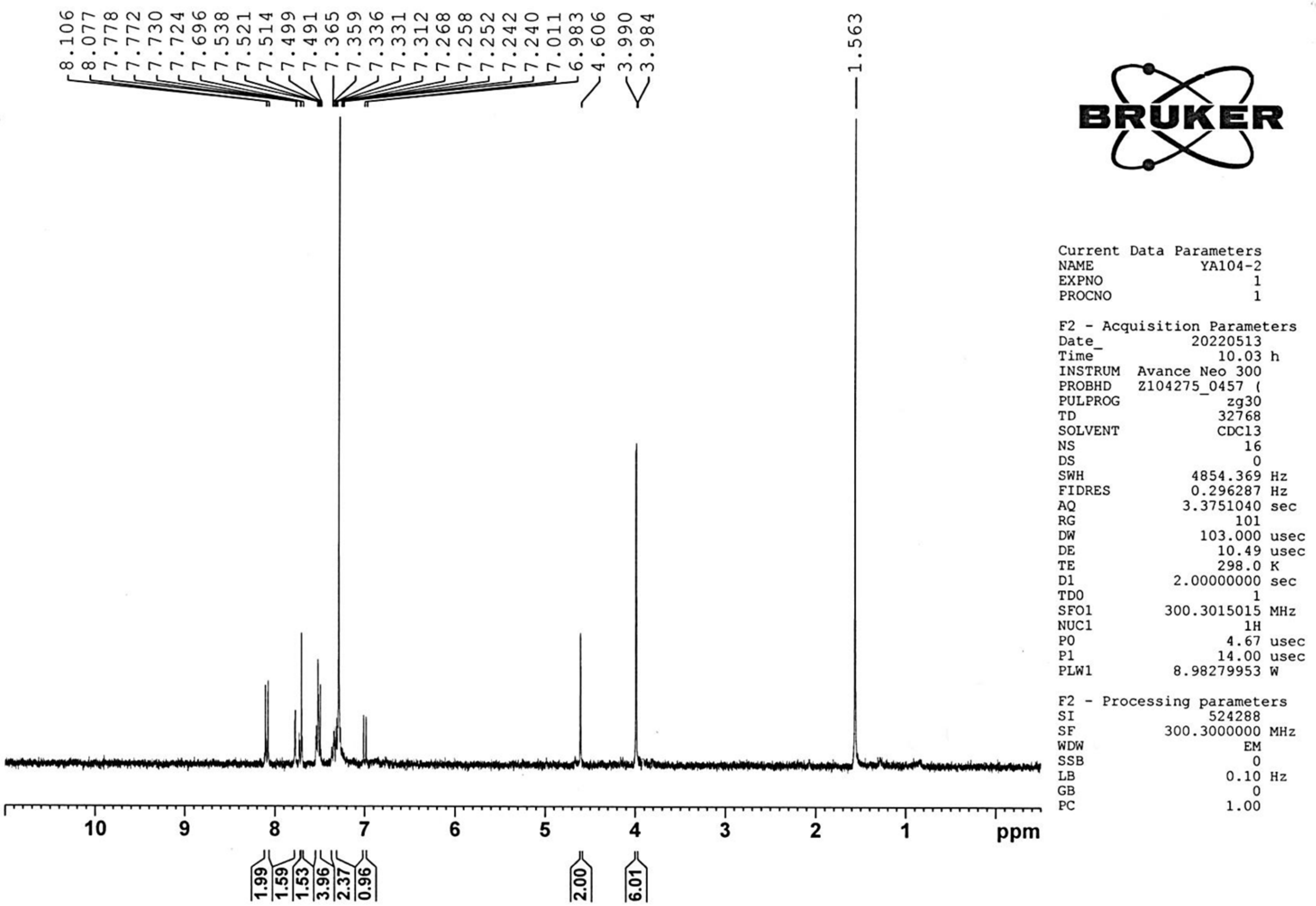

Figure S1.49. ^{1}H NMR of compound **28** ($CDCl_3$)

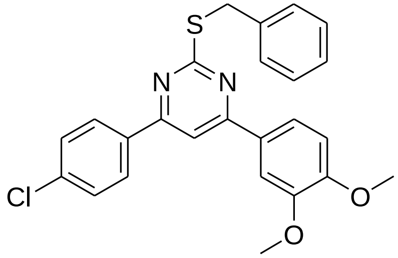

(28)

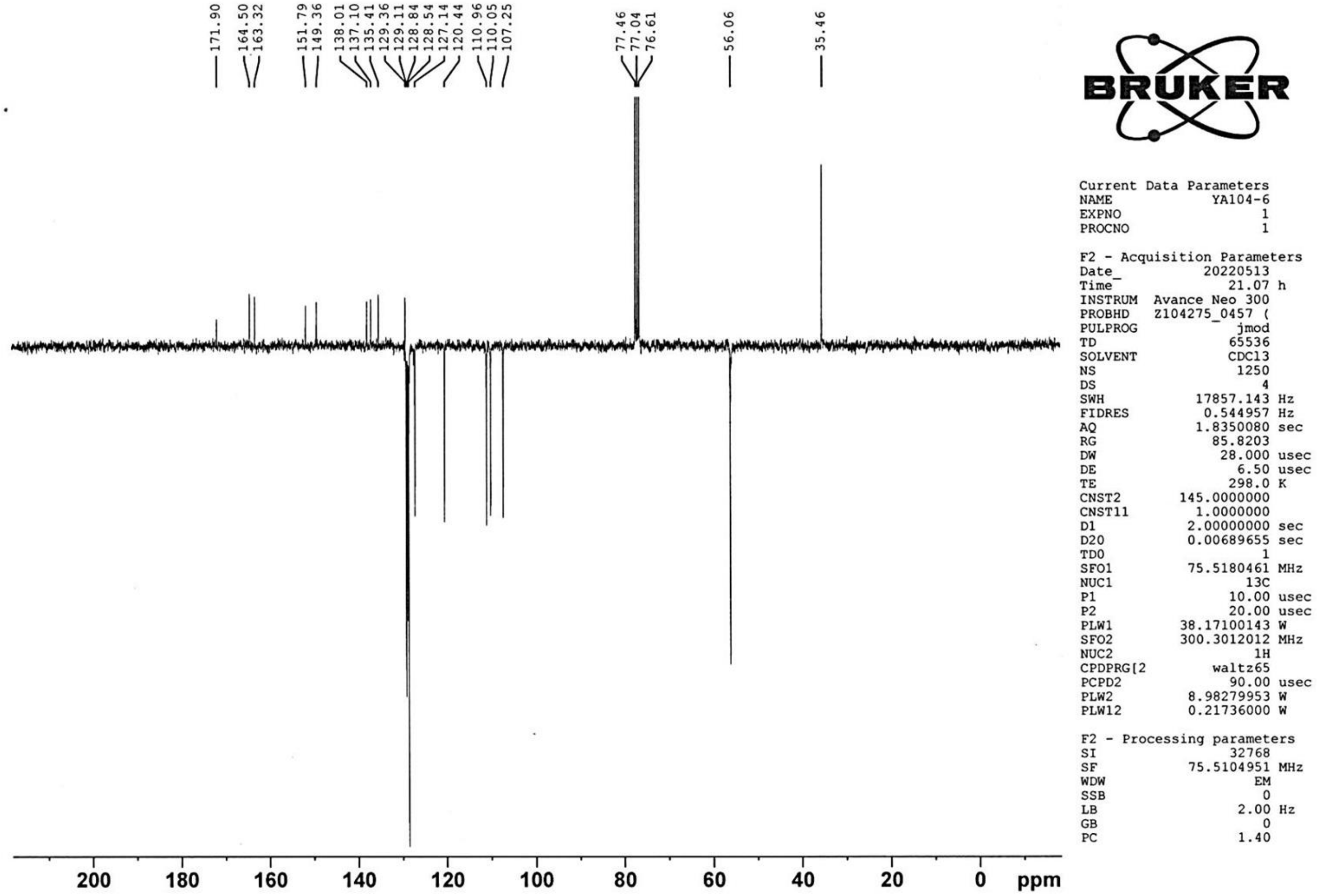

Figure S1.50. ^{13}C NMR of compound **28** ($CDCl_3$)

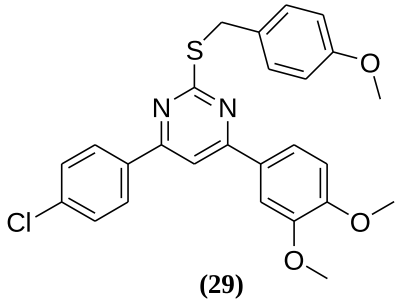

(29)

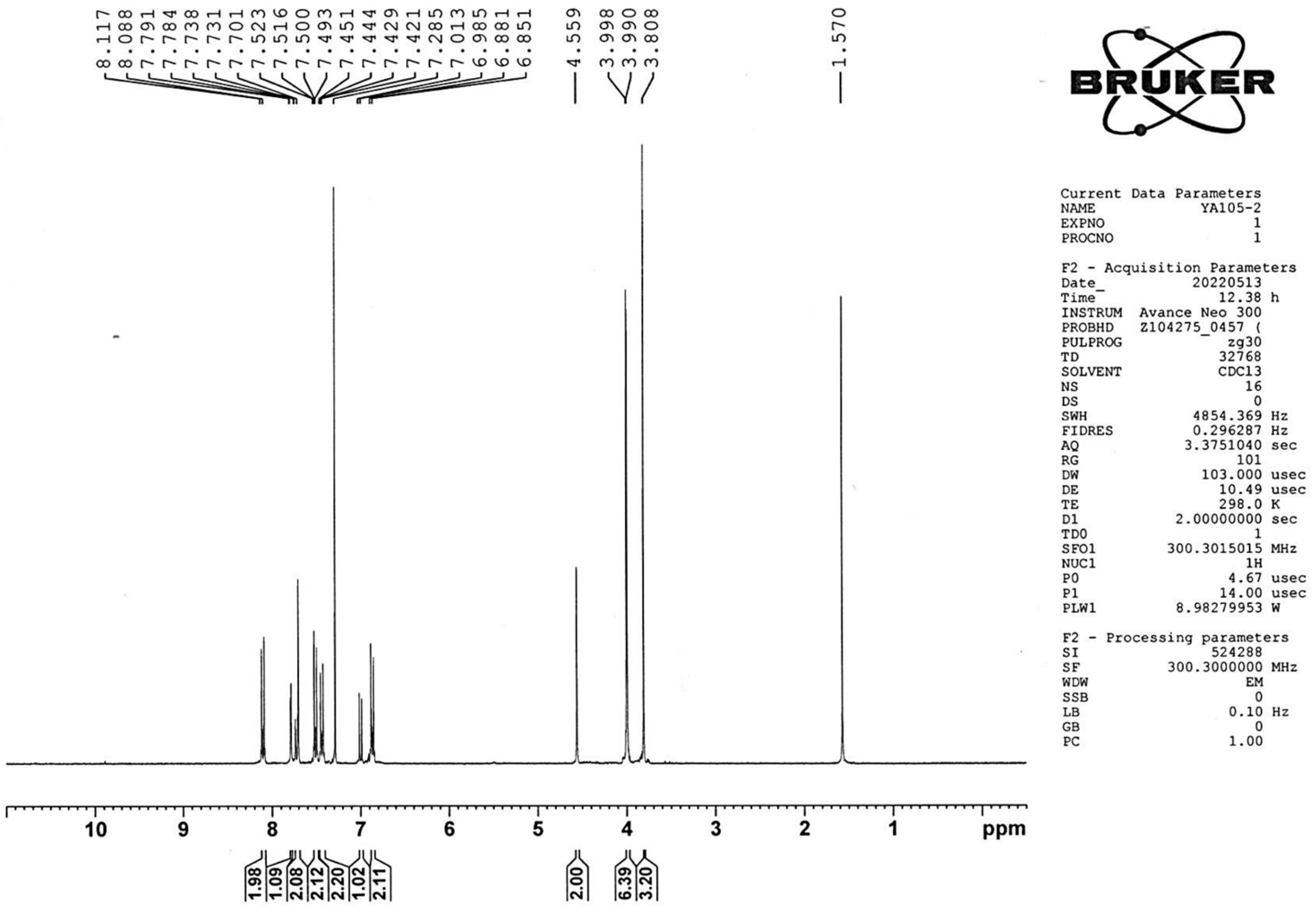

Figure S1.51. ^{1}H NMR of compound **29** ($CDCl_3$)

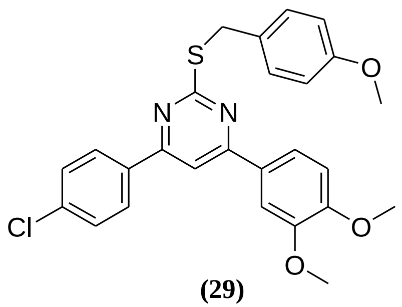

(29)

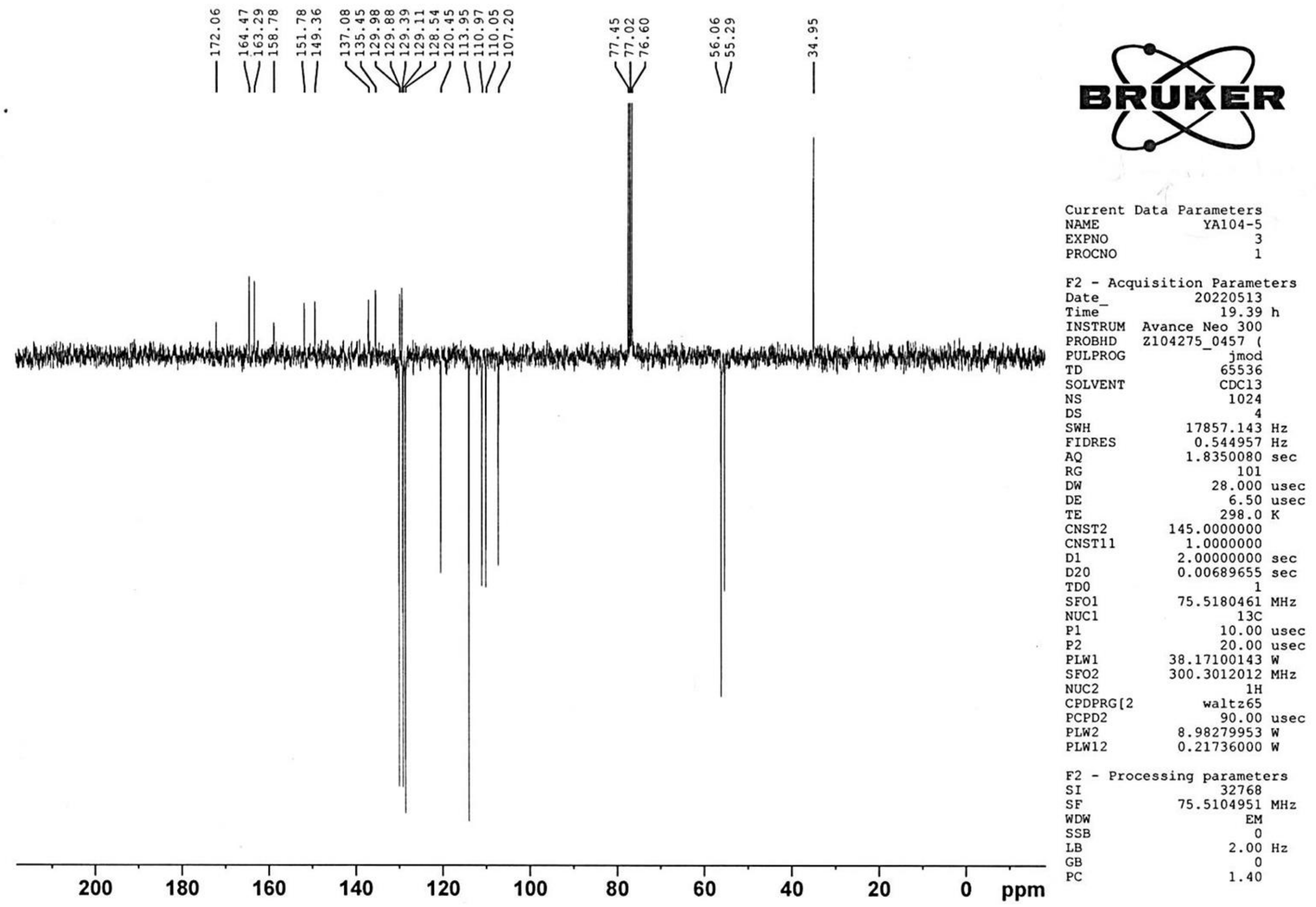

Figure S1.52. ^{13}C NMR of compound **29** ($CDCl_3$)

Section 2. NCI-60 Cancer Cell Panel Assays

a. Tables:

Table S1. NCI-60 % Growth Inhibition[a] of Test Pyrimidines

Cell line	% Growth Inhibition								
	9	16	23	10	17	24	11	18	25
**CCRF-CEM**	0.1	8.85	17.93	-[b]	2.15	19.31	1.71	-	11.22
**HL-60(TB)**	7.42	22.19	18.4	6.15	18.61	22.84	13.49	20.26	15.46
**K-562**	18.58	26.91	39.05	11.53	9.37	41.5	6.55	12.81	27.05
**MOLT-4**	11.65	19.95	26.69	2.95	4.56	36.97	2.06	6.49	19.11
**RPMI-8226**	10.75	3.81	20.05	6.56	3.83	26.37	-	-	10.83
**SR**	12.73	27.72	34.01	19.36	18.4	39.6	14.62	14.89	24.9
**A549/ATCC**	7.56	12.98	19.79	11.18	0.61	23.04	6.74	7.77	17.29
**EKVX**	19.45	21.66	23.15	7.83	1.03	20.45	6.92	-	5.09
**HOP-62**	9.16	1.53	-	-	-	-	-	-	-
**HOP-92**	3.16	1.87	-	-	-	1.94	-	-	-
**NCI-H23**	9.87	2.58	7.41	0.32	-	1.67	-	-	2.74
**NCI-H322M**	14.35	-	-	-	-	13.76	-	-	4.54
**NCI-H460**	4.25	1.75	11.4	-	-	-	-	-	-
**NCI-H522**	15.96	14.06	31.21	8.82	5.39	2.36	9.17	-	8
**COLO 205**	0.19	-	8.14	-	-	35.43	-	0.86	31.68
**HCC-2998**	-	-	-	-	-	4.44	-	-	4.26
**HCT-116**	5.47	17.33	33.72	4.3	5.86	30	-	3.37	-
**HCT-15**	14.98	19.49	27.99	14.44	2.62	30.71	7.25	8.49	20.94
**HT29**	15.57	26.2	26.36	9.13	-	28.29	10.66	4.83	14.66
**KM12**	5.71	6.2	25.4	1.5	2.23	29.81	1.24	1.47	25.53
**SW-620**	6.31	1.89	1.5	-	1.81	20.92	0.11	-	3.63
**SF-268**	4.44	15.65	11.53	-	-	16.87	-	-	6.85
**SF-295**	7.84	4.05	12.38	2.91	-	7.88	6.79	-	5.66
**SF-539**	6.28	-	15.7	-	-	7.9	-	-	1.28
**SNB-19**	4.85	-	3.67	0.21	-	3.01	1.03	-	-
**SNB-75**	-	-	-	3	-	6.37	-	-	2.64
**U251**	3.42	10.71	11.63	4.88	-	14.69	5.99	-	7.53
**LOX IMVI**	12.23	0.51	15.02	-	5.7	11.86	-	-	1.51
**MALME-3M**	11.31	-	-	-	-	-	-	-	7.34
**M14**	3.51	-	0.3	-	-	3.7	-	9.08	-
**MDA-MB-435**	1.45	6.06	17.97	8.38	2.52	19.7	1.63	5.12	0.34
**SK-MEL-2**	-	2.91	8.95	6.03	5.15	11.94	7.18	3.6	14.94
**SK-MEL-28**	-	-	8.87	-	-	5.5	-	-	17.44
**SK-MEL-5**	4.98	6.46	24.48	3.36	6.31	28.95	3.98	3.1	0.38
**UACC-257**	5.09	-	10.03	-	-	13.51	-	-	24.81
**UACC-62**	25.99	23.91	-	-	-	-	18.15	-	3.23
**IGROV1**	3.15	-	-	-	-	-	-	-	-
**OVCAR-3**	-	-	-	-	-	-	-	-	-
**OVCAR-4**	4.46	8.29	27.5	12.87	3.36	26.39	-	3.94	20.2
**OVCAR-5**	-	-	11.9	-	-	10.98	-	-	4.78
**OVCAR-8**	11.02	6.48	3.78	-	-	6.83	2.49	-	6.34
**NCI/ADR-RES**	9.84	11.57	14.91	1.42	-	17.5	-	-	11.24

**SK-OV-3**	-	13.7	2.94	-		-	-	-	2.94
**786-0**	1.88	-	6.05	3.07	-	11.82	0.11	-	-
**A498**	-	-	-	3.38	-	1.95	2.73	-	-
**ACHN 1**	4.08	-	9.14	2.57	-	7.24	-	-	7.82
**CAKI-1**	28.83	20.81	24	13.32	9.72	27.52	7.98	4	13.51
**RXF 393**	7.91	11.61	-	-	-	23.53	-	-	8.5
**SN12C**	11.2	17.87	20.19	0.46	-	22.8	6.25	-	-
**TK-10**	-	-	-	0.94	-	-	-	-	11.59
**UO-31**	18.08	20.02	22.85	10.53	13.71	25.84	9.15	-	8.18
**PC-3**	6.22	16.63	15.68	-	-	26.4	-	-	6.31
**DU-145**	5.68	6.56	9.83	-	-	7.4	0.44	-	17.62
**MCF7**	28.66	11.29	23.85	25.51	10.96	18.54	4.02	17.27	17.5
**MDA-MB231/ATCC**	20.35	10.96	23.22	-	6.41	16.9	-	-	16.21
**HS 578T**	7.95	5.45	16.97	2.58	-	20.62	-	-	-
**BT-549**	-	3.3	4.8	-	-	-	-	-	-
**T-47D**	9.97	22.25	25.54	18.55	10.15	37.62	8.26	13.77	34.51
**MDA-MB-468**	26.22	17.42	6.77	-	-	11.3	7.92	-	-

[a] Growth relative to no-drug control at concentration of 10^{-5} M; [b] No inhibition at concentration of 10^{-5} M

Table S2. NCI-60 % Growth Inhibition[a] of Test Pyrimidines

Cell line	% Growth Inhibition[a]									
	**12**	**19**	**26**	**13**	**20**	**27**	**14**	**28**	**15**	**29**
**CCRF-CEM**	24.12	9.07	1.08	3.79	3.61	13.09	-[b]	17.37	-	14.17
**HL-60(TB)**	32.59	22.58	14.53	26.79	31.34	29.25	18.09	21.35	3.01	38.84
**K-562**	30.86	29.5	6.67	15.58	11.76	41.97	11.68	30.95	12.81	54.54
**MOLT-4**	29.88	23.77	5.51	14.33	12.49	28.46	4.54	32.81	3.03	46.5
**RPMI-8226**	26.7	10.06	0.53	4.91	4.53	14.32	-	24.27	-	36.9
**SR**	47.1	27.55	12.6	19.87	13.04	30.31	10.18	39.44	8.9	45.8
**A549/ATCC**	15.93	15.86	0.02	-	1.93	12.27	2.08	18.86	-	30.07
**EKVX**	16.75	22.72	4.59	2.11	4.8	12.16	2.76	22.24	-	29.67
**HOP-62**	-	-	-	-	-	-	-	-	-	16.4
**HOP-92**	-	-	-	-	-	10.64	-	0.63	-	20.62
**NCI-H23**	10.72	8.76	-	3.36	1.43	7.78	-	2.2	4.6	14.56
**NCI-H322M**	4.22	4.92	1.11	0.59	-	-	-	-	-	17.41
**NCI-H460**	7.21	2.13	-	0.1	-	6.7	-	14.82	-	21.2
**NCI-H522**	21.62	20.97	9.05	10.16	8.49	16.43	-	31.82	-	23.14
**COLO 205**	-	0.02	-	-	-	-	10.32	3.49	2.89	11.35
**HCC-2998**	1.06	-	-	-	2.37	-	-	-	-	-
**HCT-116**	19.58	23.83	2.84	5.47	5.47	21.81	-	25.87	-	30.23
**HCT-15**	38.68	24.52	4.73	7.93	5.58	18.83	4.95	27.64	0.53	42.15
**HT29**	29.24	32.48	13.07	17.19	8.08	22.57	8.96	18.66	4.35	38.02
**KM12**	9.45	5.72	1.72	0.8	-	3.43	-	23.2	-	24.77
**SW-620**	5.57	-	2.64	-	-	1.7	-	2.79	-	12.7
**SF-268**	-	9.81	-	-	-	-	-	10.82	-	32.98
**SF-295**	4.52	4.16	-	1.47	3.19	3	-	7.84	-	16.74
**SF-539**	6.88	0.24	-	-	-	-	-	17.83	-	14.66
**SNB-19**	1.49	7.53	3.77	6.79	5.77	8.39	-	4.06	-	14.21
**SNB-75**	-	-	-	-	-	-	-	-	-	1.93
**U251**	7.59	10.2	-	-	-	-	-	13.1	-	23.29
**LOX IMVI**	13.55	6.05	4.14	4.72	7.36	7.11	3.14	8.32	-	20.89
**MALME-3M**	1.51	-	-	-	-	-	-	-	-	4.95
**M14**	8.46	-	-	4.04	4.9	0.06	-	0.81	-	19.33
**MDA-MB-435**	8.92	3.92	1.25	5.34	1.82	6.35	3.68	15.25	2.38	22.77
**SK-MEL-2**	19.51	7.18	-	4.96	-	0.52	7.79	12.04	-	13.73
**SK-MEL-28**	-	-	-	-	-	-	-	4.01	-	11.28
**SK-MEL-5**	17.88	7.31	-	1	1.41	12.18	2.06	30.09	-	22.58
**UACC-257**	4.1	-	0.69	-	-	-	-	15.29	-	0.85
**UACC-62**	19.06	25.49	17.4	26.17	18.78	25.96	-	-	-	38.69
**IGROV1**	-	-	-	-	-	-	-	-	-	6.14
**OVCAR-3**	-	-	-	-	-	-	6.22	-	5.05	-
**OVCAR-4**	11.81	4.28	-	12.08	5.65	10.26	-	21.48	-	21.92
**OVCAR-5**	-	0.61	-	-	-	-	2.5	6.33	-	7.38
**OVCAR-8**	11.26	10.72	4.2	-	0.92	7.89	-	8.97	1.69	19.42
**NCI/ADR-RES**	11.17	9.86	-	6.28	-	21.79	-	14.28	-	32.11
**SK-OV-3**	-	-	-	-	-	-	-	2.92	-	13.92
**786-0**	7.48	4.09	-	3.01	-	2.53	-	7.96	-	11.62
**A498**	-	-	-	-	-	-	-	0.85	-	-
**ACHN 1**	-	-	-	-	-	6.5	11.04	13.09	15.77	13.24

**CAKI-1**	14.07	24.08	9.22	14.84	7.19	13.14	2.18	25.69	-	23.15
**RXF 393**	26.38	18.92	-	0.35	-	3.07	-	-	-	24.23
**SN12C**	19.78	18.51	8.05	6.62	6.16	14.34	10.39	23.12	11.27	35.57
**TK-10**	-	-	-	-	-	-	-	-	-	-
**UO-31**	15.68	23.65	15.18	18.92	13.18	22.85	-	24.79	-	33.09
**PC-3**	2.88	17.24	-	1.15	-	15.7	20.73	18.97	17.53	36.44
**DU-145**	14.7	4.58	-	1.02	-	2.02	1.74	8.2	0.47	28.55
**MCF7**	28.79	11.62	18.05	-	12.51	17.95	-	12.94	-	27.37
**MDA-MB231/ATCC**	4.87	17.26	4.5	-	-	7.53	-	22.96	-	30.34
**HS 578T**	-	4.95	-	-	-	-	-	18.16	9.27	37.47
**BT-549**	2.74	6.85	-	-	-	3.91	-	3.64	-	21.44
**T-47D**	40.44	24.63	14.16	18.43	21.51	23.15	14.43	32.97	-	34.57
**MDA-MB-468**	35.14	20.16	7.33	16.17	1.99	23.67	-	6.53	-	40.21

[a] Growth relative to no-drug control at concentration of 10^{-5} M; [b] No inhibition at concentration of 10^{-5} M

b. NCI Monographs:

Compound (9)

Developmental Therapeutics Program
One Dose Mean Graph

NSC: D-841405 / 1 | **Conc:** 1.00E-5 Molar | **Test Date:** Jan 09, 2023
Experiment ID: 2301OS86 | **Report Date:** Feb 28, 2023

Panel/Cell Line	Growth Percent
Leukemia	
CCRF-CEM	99.90
HL-60(TB)	92.58
K-562	81.42
MOLT-4	88.35
RPMI-8226	89.25
SR	87.27
Non-Small Cell Lung Cancer	
A549/ATCC	92.44
EKVX	80.55
HOP-62	90.84
HOP-92	96.84
NCI-H23	90.13
NCI-H322M	85.65
NCI-H460	95.75
NCI-H522	84.04
Colon Cancer	
COLO 205	99.81
HCC-2998	102.63
HCT-116	94.53
HCT-15	85.02
HT29	84.43
KM12	94.29
SW-620	93.69
CNS Cancer	
SF-268	95.56
SF-295	92.16
SF-539	93.72
SNB-19	95.15
SNB-75	101.22
U251	96.58
Melanoma	
LOX IMVI	87.76
MALME-3M	88.69
M14	96.49
MDA-MB-435	98.55
SK-MEL-2	107.08
SK-MEL-28	101.96
SK-MEL-5	95.02
UACC-257	94.91
UACC-62	74.01
Ovarian Cancer	
IGROV1	96.85
OVCAR-3	111.77
OVCAR-4	95.54
OVCAR-5	113.60
OVCAR-8	89.98
NCI/ADR-RES	90.16
SK-OV-3	108.57
Renal Cancer	
786-0	98.02
A498	112.72
ACHN	95.92
CAKI-1	71.17
RXF 393	92.09
SN12C	89.80
TK-10	112.80
UO-31	81.92
Prostate Cancer	
PC-3	93.78
DU-145	94.32
Breast Cancer	
MCF7	71.34
MDA-MB-231/ATCC	79.65
HS 578T	92.05
BT-549	100.25
T-47D	90.03
MDA-MB-468	73.78
Mean	92.96
Delta	21.79
Range	42.43

Mean Growth Percent - Growth Percent

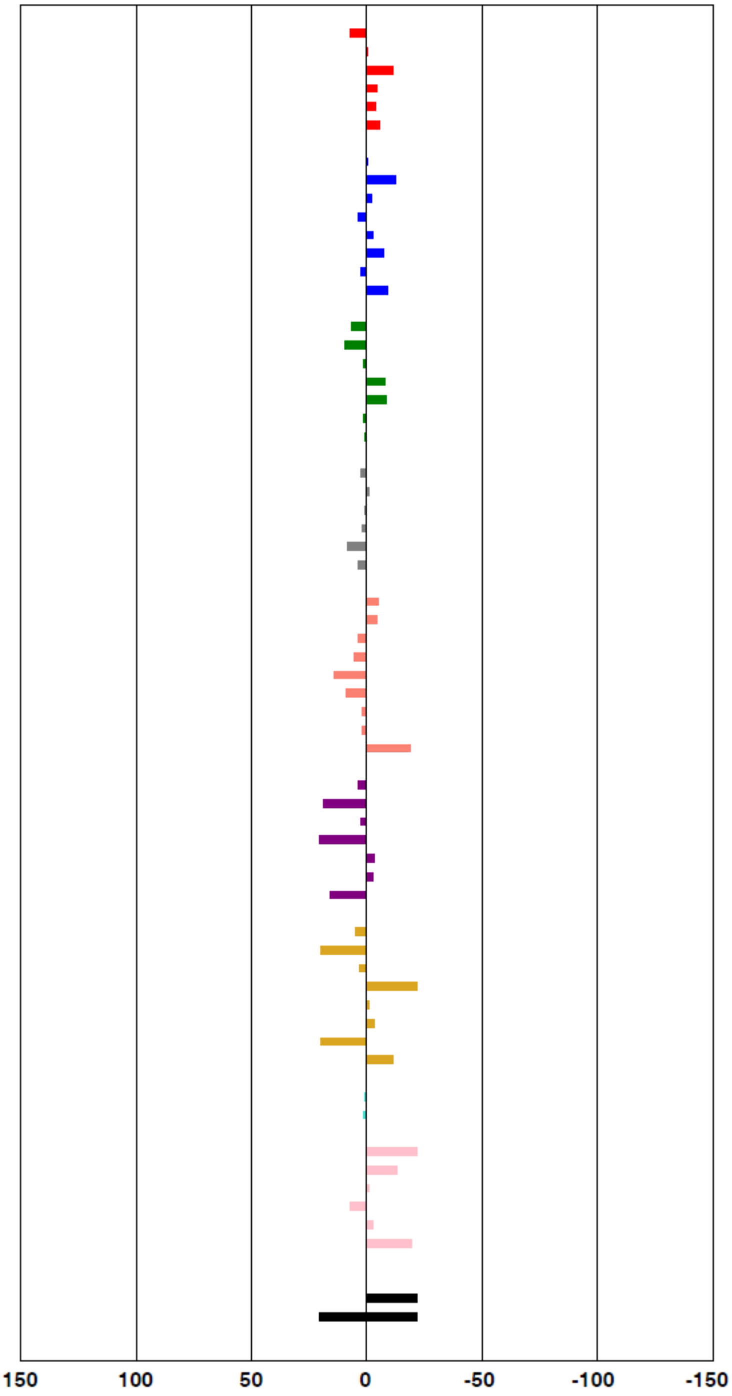

Compound (10)

Developmental Therapeutics Program One Dose Bar Graph	NSC: D-847378 / 1	Conc: 1.00E-5 Molar	Test Date: Aug 07, 2023
	Experiment ID: 2308OS53		Report Date: Oct 17, 2023

Panel/Cell Line	Growth Percent
Leukemia	
CCRF-CEM	101.57
HL-60(TB)	93.86
K-562	88.48
MOLT-4	97.05
RPMI-8226	93.44
SR	80.64
Non-Small Cell Lung Cancer	
A549/ATCC	88.83
EKVX	92.18
HOP-62	109.28
HOP-92	130.05
NCI-H226	99.95
NCI-H23	99.68
NCI-H322M	107.56
NCI-H460	101.89
NCI-H522	91.18
Colon Cancer	
COLO 205	110.52
HCC-2998	113.43
HCT-116	95.70
HCT-15	85.57
HT29	90.88
KM12	98.50
SW-620	100.03
CNS Cancer	
SF-268	102.90
SF-295	97.09
SF-539	103.49
SNB-19	99.79
SNB-75	97.00
U251	95.12
Melanoma	
LOX IMVI	101.27
MALME-3M	117.66
M14	102.71
MDA-MB-435	91.63
SK-MEL-2	93.98
SK-MEL-28	100.96
SK-MEL-5	96.64
UACC-257	104.83
Ovarian Cancer	
IGROV1	112.38
OVCAR-3	104.04
OVCAR-4	87.13
OVCAR-5	109.17
OVCAR-8	102.77
NCI/ADR-RES	98.59
SK-OV-3	113.57
Renal Cancer	
786-0	96.94
A498	96.63
ACHN	97.44
CAKI-1	86.69
SN12C	99.54
TK-10	99.07
UO-31	89.05
Prostate Cancer	
PC-3	118.31
DU-145	100.32
Breast Cancer	
MCF7	74.49
MDA-MB-231/ATCC	100.25
HS 578T	97.43
BT-549	102.61
T-47D	81.45
MDA-MB-468	105.44

Bar Graph

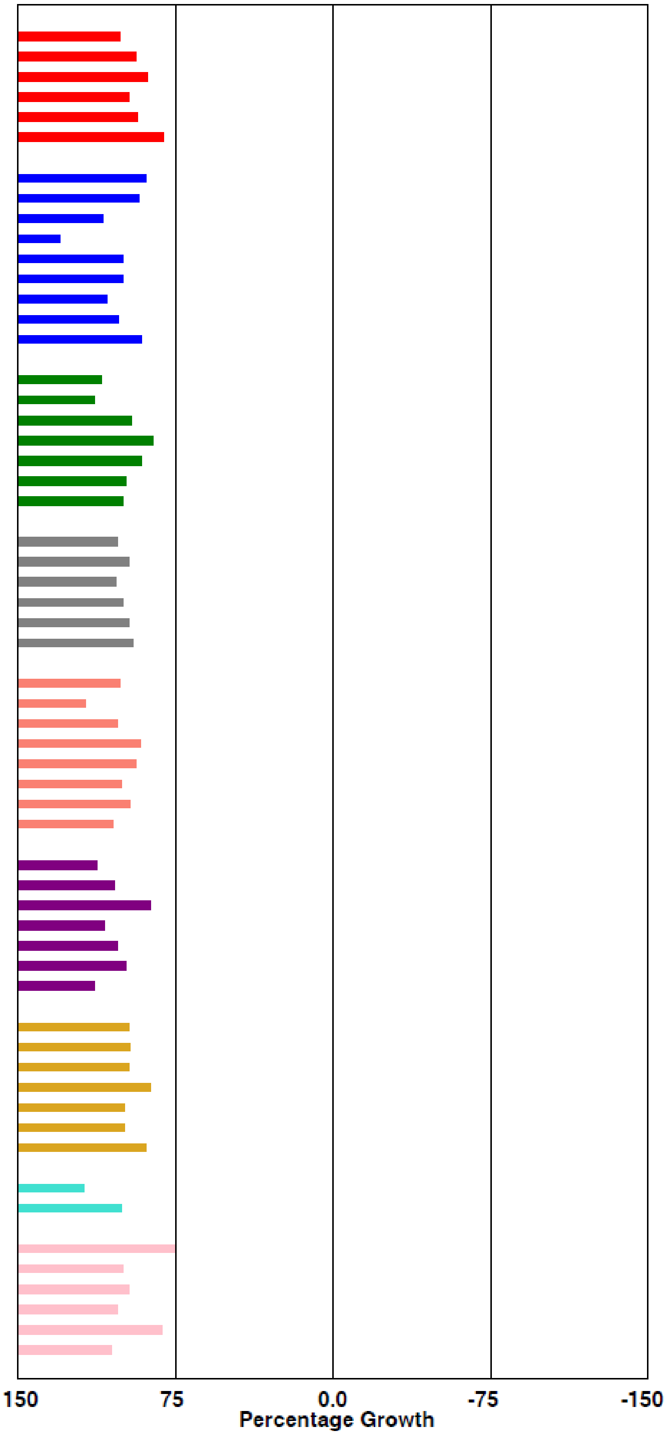

Compound (11)

Developmental Therapeutics Program One Dose Mean Graph	NSC: D-841406 / 1	Conc: 1.00E-5 Molar	Test Date: Jan 09, 2023
	Experiment ID: 2301OS86		Report Date: Feb 28, 2023

Panel/Cell Line	Growth Percent
**Leukemia**	
CCRF-CEM	98.29
HL-60(TB)	86.51
K-562	93.45
MOLT-4	97.94
RPMI-8226	106.08
SR	85.38
**Non-Small Cell Lung Cancer**	
A549/ATCC	93.26
EKVX	93.08
HOP-62	102.08
HOP-92	119.07
NCI-H23	102.14
NCI-H322M	105.71
NCI-H460	101.47
NCI-H522	90.83
**Colon Cancer**	
COLO 205	118.28
HCC-2998	118.29
HCT-116	104.68
HCT-15	92.75
HT29	89.34
KM12	98.76
SW-620	99.89
**CNS Cancer**	
SF-268	101.56
SF-295	93.21
SF-539	108.95
SNB-19	98.97
SNB-75	102.62
U251	94.01
**Melanoma**	
LOX IMVI	101.49
MALME-3M	103.92
M14	106.05
MDA-MB-435	98.37
SK-MEL-2	92.82
SK-MEL-28	114.76
SK-MEL-5	96.02
UACC-257	100.09
UACC-62	81.85
**Ovarian Cancer**	
IGROV1	109.91
OVCAR-3	117.94
OVCAR-4	101.45
OVCAR-5	121.60
OVCAR-8	97.51
NCI/ADR-RES	100.64
SK-OV-3	114.52
**Renal Cancer**	
786-0	99.89
A498	97.27
ACHN	113.35
CAKI-1	92.02
RXF 393	104.17
SN12C	93.75
TK-10	102.61
UO-31	90.85
**Prostate Cancer**	
PC-3	108.01
DU-145	99.56
**Breast Cancer**	
MCF7	95.98
MDA-MB-231/ATCC	106.28
HS 578T	108.58
BT-549	106.33
T-47D	91.74
MDA-MB-468	92.08
Mean	100.98
Delta	19.13
Range	39.75

Mean Growth Percent - Growth Percent

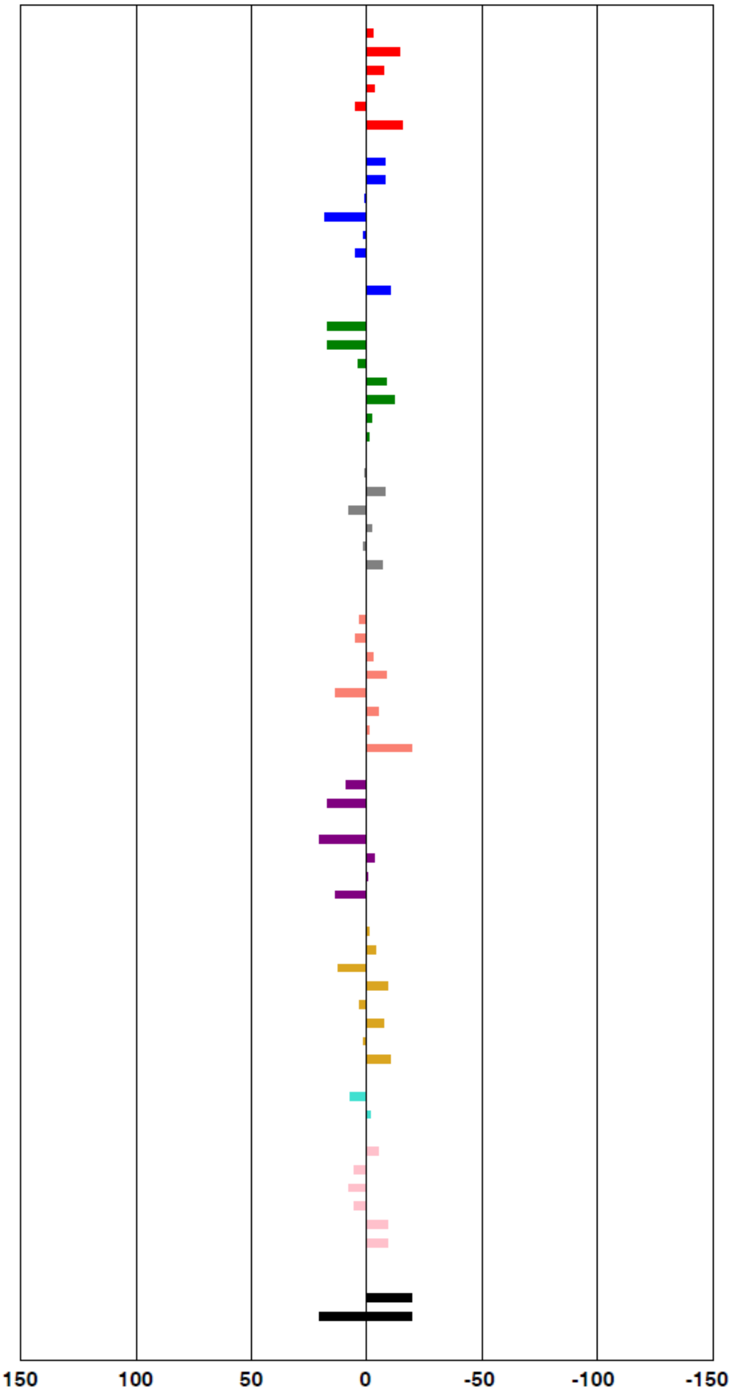

Compound (12)

Developmental Therapeutics Program One Dose Mean Graph	NSC: D-841402 / 1	Conc: 1.00E-5 Molar	Test Date: Jan 09, 2023
	Experiment ID: 2301OS86		Report Date: Feb 28, 2023

Panel/Cell Line	Growth Percent
**Leukemia**	
CCRF-CEM	75.88
HL-60(TB)	67.41
K-562	69.14
MOLT-4	70.12
RPMI-8226	73.30
SR	52.90
**Non-Small Cell Lung Cancer**	
A549/ATCC	84.07
EKVX	83.25
HOP-62	102.84
HOP-92	110.12
NCI-H23	89.28
NCI-H322M	95.78
NCI-H460	92.79
NCI-H522	79.38
**Colon Cancer**	
COLO 205	104.95
HCC-2998	98.94
HCT-116	80.42
HCT-15	61.32
HT29	70.76
KM12	90.55
SW-620	94.43
**CNS Cancer**	
SF-268	103.03
SF-295	95.48
SF-539	93.12
SNB-19	98.51
SNB-75	106.71
U251	92.41
**Melanoma**	
LOX IMVI	86.45
MALME-3M	98.49
M14	91.54
MDA-MB-435	91.08
SK-MEL-2	80.49
SK-MEL-28	106.34
SK-MEL-5	82.22
UACC-257	95.90
UACC-62	80.94
**Ovarian Cancer**	
IGROV1	107.84
OVCAR-3	116.48
OVCAR-4	88.19
OVCAR-5	121.05
OVCAR-8	88.74
NCI/ADR-RES	88.83
SK-OV-3	114.55
**Renal Cancer**	
786-0	92.52
A498	127.05
ACHN	107.09
CAKI-1	85.93
RXF 393	73.62
SN12C	80.22
TK-10	115.20
UO-31	84.32
**Prostate Cancer**	
PC-3	97.12
DU-145	85.30
**Breast Cancer**	
MCF7	71.21
MDA-MB-231/ATCC	95.13
HS 578T	109.70
BT-549	97.26
T-47D	59.56
MDA-MB-468	64.86
Mean	90.21
Delta	37.31
Range	74.15

Mean Growth Percent - Growth Percent

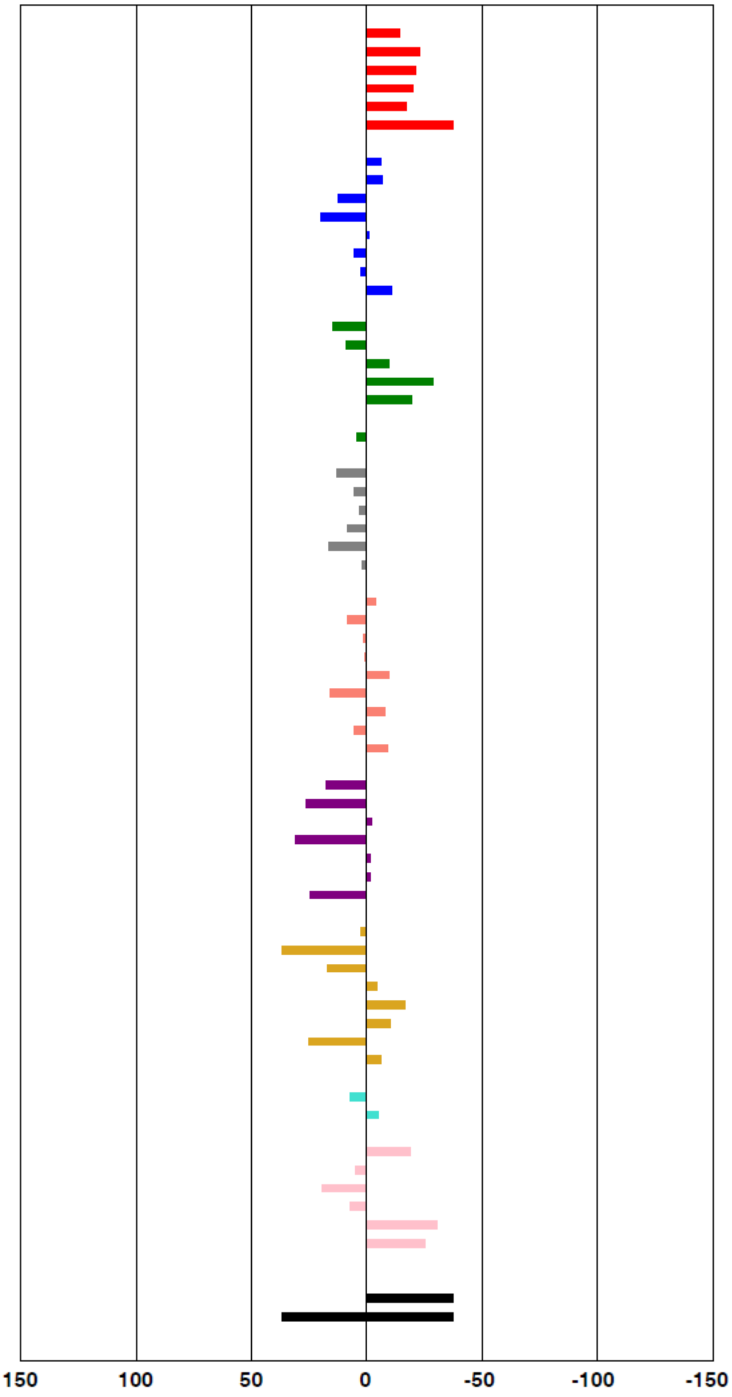

Compound (13)

Developmental Therapeutics Program
One Dose Mean Graph

NSC: D-841409 / 1 | **Conc:** 1.00E-5 Molar | **Test Date:** Jan 09, 2023
Experiment ID: 2301OS86 | **Report Date:** Feb 28, 2023

Panel/Cell Line	Growth Percent
**Leukemia**	
CCRF-CEM	96.21
HL-60(TB)	73.21
K-562	84.42
MOLT-4	85.67
RPMI-8226	95.09
SR	80.13
**Non-Small Cell Lung Cancer**	
A549/ATCC	103.20
EKVX	97.89
HOP-62	103.49
HOP-92	112.83
NCI-H23	96.64
NCI-H322M	99.41
NCI-H460	99.90
NCI-H522	89.84
**Colon Cancer**	
COLO 205	104.88
HCC-2998	106.59
HCT-116	94.53
HCT-15	92.07
HT29	82.81
KM12	99.20
SW-620	100.67
**CNS Cancer**	
SF-268	110.88
SF-295	98.53
SF-539	102.12
SNB-19	93.21
SNB-75	106.57
U251	104.83
**Melanoma**	
LOX IMVI	95.28
MALME-3M	110.65
M14	95.96
MDA-MB-435	94.66
SK-MEL-2	95.04
SK-MEL-28	102.35
SK-MEL-5	99.00
UACC-257	102.80
UACC-62	73.83
**Ovarian Cancer**	
IGROV1	105.91
OVCAR-3	119.41
OVCAR-4	87.92
OVCAR-5	111.54
OVCAR-8	100.38
NCI/ADR-RES	93.72
SK-OV-3	111.40
**Renal Cancer**	
786-0	96.99
A498	102.79
ACHN	105.20
CAKI-1	85.16
RXF 393	99.65
SN12C	93.38
TK-10	107.97
UO-31	81.08
**Prostate Cancer**	
PC-3	98.85
DU-145	98.98
**Breast Cancer**	
MCF7	100.01
MDA-MB-231/ATCC	104.53
HS 578T	108.70
BT-549	106.30
T-47D	81.57
MDA-MB-468	83.83
Mean	97.79
Delta	24.58
Range	46.20

Mean Growth Percent - Growth Percent

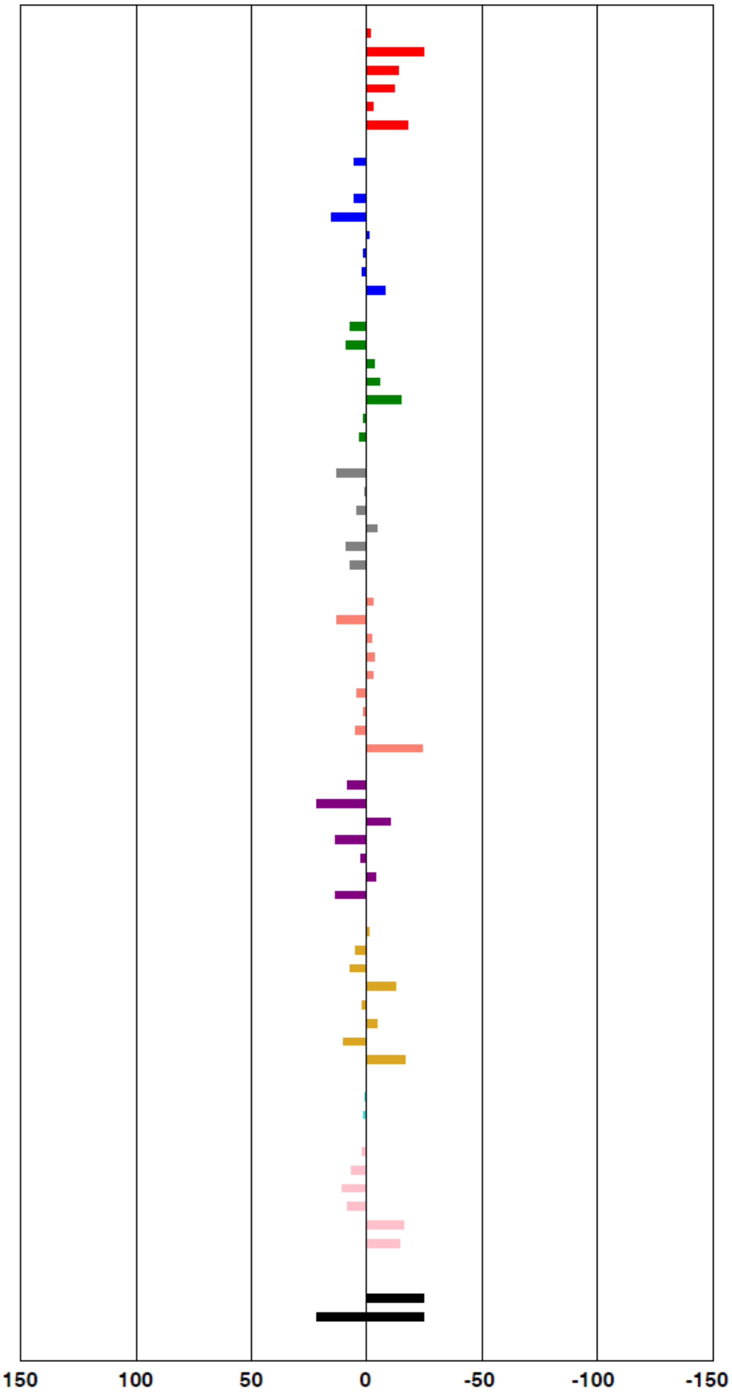

Compound (14)

Developmental Therapeutics Program
One Dose Bar Graph

NSC: D-847377 / 1 | **Conc:** 1.00E-5 Molar | **Test Date:** Aug 07, 2023

Experiment ID: 2308OS53 | **Report Date:** Oct 17, 2023

Panel/Cell Line	Growth Percent
Leukemia	
CCRF-CEM	102.47
HL-60(TB)	81.91
K-562	88.32
MOLT-4	95.46
RPMI-8226	106.58
SR	89.83
Non-Small Cell Lung Cancer	
A549/ATCC	97.93
EKVX	97.25
HOP-62	113.25
HOP-92	111.90
NCI-H226	103.66
NCI-H23	100.10
NCI-H322M	106.43
NCI-H460	100.89
NCI-H522	89.69
Colon Cancer	
COLO 205	112.67
HCC-2998	116.86
HCT-116	95.05
HCT-15	91.04
HT29	96.42
KM12	102.36
SW-620	100.78
CNS Cancer	
SF-268	103.23
SF-295	107.99
SF-539	101.13
SNB-19	100.54
SNB-75	110.54
U251	101.94
Melanoma	
LOX IMVI	96.86
MALME-3M	115.03
M14	108.67
MDA-MB-435	96.33
SK-MEL-2	92.21
SK-MEL-28	104.61
SK-MEL-5	97.94
UACC-257	102.34
Ovarian Cancer	
IGROV1	109.77
OVCAR-3	112.00
OVCAR-4	93.78
OVCAR-5	113.00
OVCAR-8	97.50
NCI/ADR-RES	102.64
SK-OV-3	102.36
Renal Cancer	
786-0	107.22
A498	108.68
ACHN	103.42
CAKI-1	88.96
SN12C	97.82
TK-10	117.39
UO-31	89.62
Prostate Cancer	
PC-3	117.69
DU-145	105.46
Breast Cancer	
MCF7	79.28
MDA-MB-231/ATCC	98.27
HS 578T	107.86
BT-549	118.60
T-47D	85.57
MDA-MB-468	102.51

Bar Graph

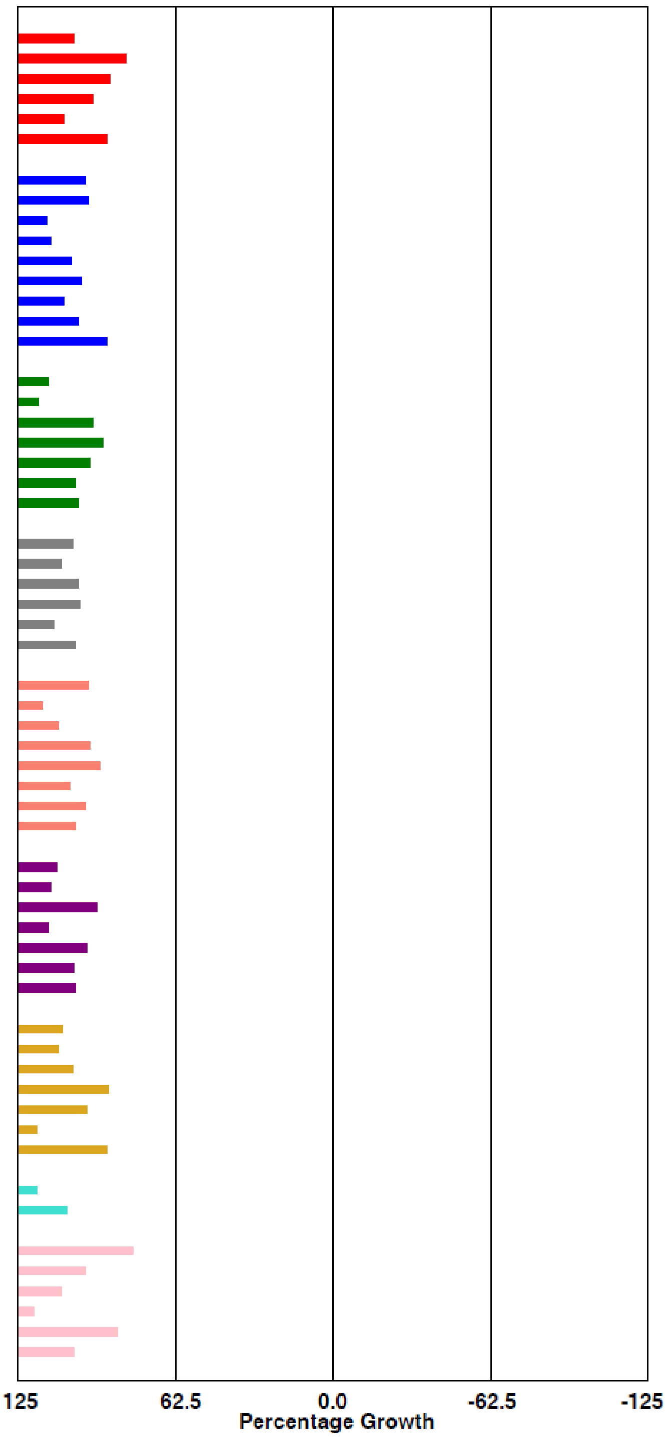

Compound (15)

Developmental Therapeutics Program One Dose Bar Graph	NSC: D-847376 / 1	Conc: 1.00E-5 Molar	Test Date: Aug 07, 2023
	Experiment ID: 2308OS53		Report Date: Oct 17, 2023

Panel/Cell Line	Growth Percent
Leukemia	
CCRF-CEM	103.19
HL-60(TB)	96.99
K-562	87.19
MOLT-4	96.98
RPMI-8226	101.46
SR	91.10
Non-Small Cell Lung Cancer	
A549/ATCC	100.50
EKVX	100.39
HOP-62	115.68
HOP-92	121.19
NCI-H226	95.41
NCI-H23	102.84
NCI-H322M	123.14
NCI-H460	107.95
NCI-H522	97.11
Colon Cancer	
COLO 205	110.21
HCC-2998	111.75
HCT-116	99.47
HCT-15	95.66
HT29	103.47
KM12	103.73
SW-620	100.77
CNS Cancer	
SF-268	107.92
SF-295	105.52
SF-539	106.51
SNB-19	100.02
SNB-75	104.95
U251	107.57
Melanoma	
LOX IMVI	101.90
MALME-3M	120.49
M14	108.20
MDA-MB-435	97.62
SK-MEL-2	103.23
SK-MEL-28	111.24
SK-MEL-5	101.84
UACC-257	102.91
Ovarian Cancer	
IGROV1	118.61
OVCAR-3	115.26
OVCAR-4	94.96
OVCAR-5	107.08
OVCAR-8	100.13
NCI/ADR-RES	98.32
SK-OV-3	106.18
Renal Cancer	
786-0	102.44
A498	115.81
ACHN	101.23
CAKI-1	84.23
SN12C	104.16
TK-10	117.06
UO-31	88.73
Prostate Cancer	
PC-3	113.51
DU-145	105.28
Breast Cancer	
MCF7	82.48
MDA-MB-231/ATCC	99.53
HS 578T	105.87
BT-549	122.00
T-47D	90.73
MDA-MB-468	114.42

Bar Graph

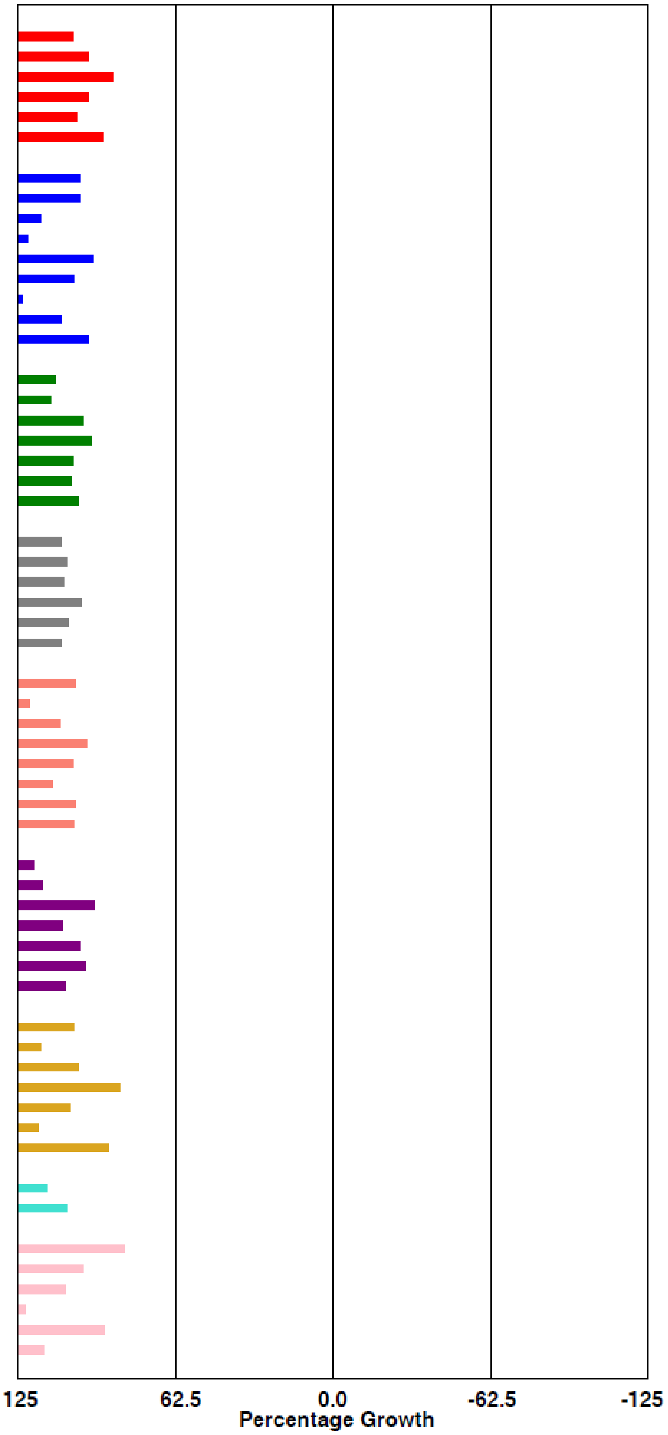

Compound (16)

Developmental Therapeutics Program One Dose Mean Graph	NSC: D-841407 / 1	Conc: 1.00E-5 Molar	Test Date: Jan 09, 2023
	Experiment ID: 2301OS86		Report Date: Feb 28, 2023

Panel/Cell Line	Growth Percent
**Leukemia**	
CCRF-CEM	91.15
HL-60(TB)	77.81
K-562	73.09
MOLT-4	80.05
RPMI-8226	96.19
SR	72.28
**Non-Small Cell Lung Cancer**	
A549/ATCC	87.02
EKVX	78.34
HOP-62	98.47
HOP-92	98.13
NCI-H23	97.42
NCI-H322M	100.09
NCI-H460	98.25
NCI-H522	85.94
**Colon Cancer**	
COLO 205	102.55
HCC-2998	113.28
HCT-116	82.67
HCT-15	80.51
HT29	73.80
KM12	93.80
SW-620	98.11
**CNS Cancer**	
SF-268	84.35
SF-295	95.95
SF-539	101.88
SNB-19	100.05
SNB-75	108.33
U251	89.29
**Melanoma**	
LOX IMVI	99.49
MALME-3M	110.09
M14	103.63
MDA-MB-435	93.94
SK-MEL-2	97.09
SK-MEL-28	110.33
SK-MEL-5	93.54
UACC-257	101.75
UACC-62	76.09
**Ovarian Cancer**	
IGROV1	106.33
OVCAR-3	122.53
OVCAR-4	91.71
OVCAR-5	102.13
OVCAR-8	93.52
NCI/ADR-RES	88.43
SK-OV-3	86.29
**Renal Cancer**	
786-0	100.52
A498	101.75
ACHN	109.42
CAKI-1	79.19
RXF 393	88.39
SN12C	82.13
TK-10	105.01
UO-31	79.98
**Prostate Cancer**	
PC-3	83.37
DU-145	93.44
**Breast Cancer**	
MCF7	88.71
MDA-MB-231/ATCC	89.04
HS 578T	94.55
BT-549	96.70
T-47D	77.75
MDA-MB-468	82.58
Mean	93.02
Delta	20.74
Range	50.25

Mean Growth Percent - Growth Percent

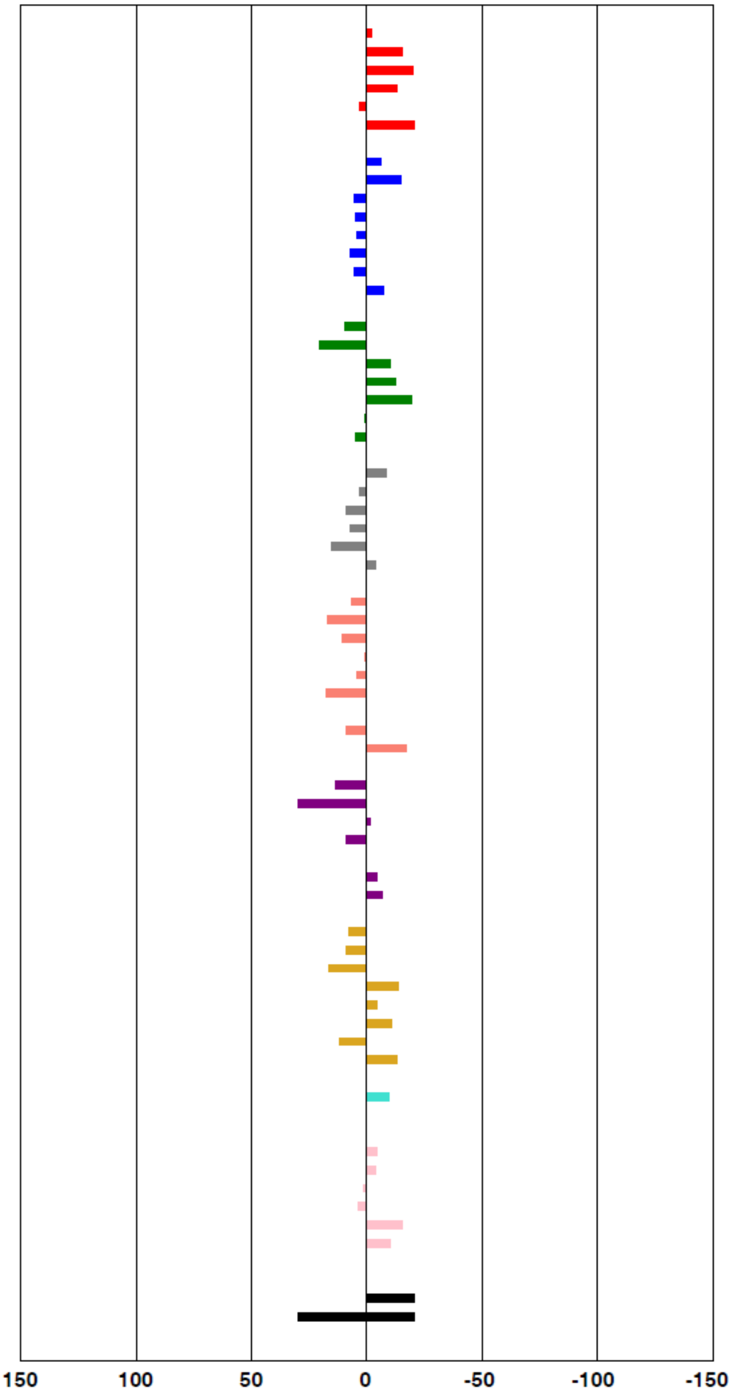

Compound (17)

Developmental Therapeutics Program One Dose Bar Graph	NSC: D-847384 / 1	Conc: 1.00E-5 Molar	Test Date: Aug 07, 2023
	Experiment ID: 2308OS53		Report Date: Oct 17, 2023

Panel/Cell Line	Growth Percent
**Leukemia**	
CCRF-CEM	97.85
HL-60(TB)	81.39
K-562	90.63
MOLT-4	95.45
RPMI-8226	96.18
SR	81.61
**Non-Small Cell Lung Cancer**	
A549/ATCC	99.40
EKVX	98.97
HOP-62	117.57
HOP-92	122.05
NCI-H226	108.87
NCI-H23	102.31
NCI-H322M	115.43
NCI-H460	108.27
NCI-H522	94.62
**Colon Cancer**	
COLO 205	108.94
HCC-2998	121.45
HCT-116	94.15
HCT-15	97.38
HT29	103.28
KM12	97.78
SW-620	98.19
**CNS Cancer**	
SF-268	103.94
SF-295	108.11
SF-539	106.95
SNB-19	101.89
SNB-75	112.51
U251	102.13
**Melanoma**	
LOX IMVI	94.30
MALME-3M	111.93
M14	103.53
MDA-MB-435	97.48
SK-MEL-2	94.85
SK-MEL-28	109.24
SK-MEL-5	93.70
UACC-257	101.61
**Ovarian Cancer**	
IGROV1	103.60
OVCAR-3	116.22
OVCAR-4	96.64
OVCAR-5	111.34
OVCAR-8	104.34
NCI/ADR-RES	102.79
SK-OV-3	108.23
**Renal Cancer**	
786-0	106.48
A498	109.30
ACHN	106.42
CAKI-1	90.29
SN12C	102.78
TK-10	119.01
UO-31	86.29
**Prostate Cancer**	
PC-3	114.28
DU-145	106.13
**Breast Cancer**	
MCF7	89.05
MDA-MB-231/ATCC	93.60
HS 578T	100.12
BT-549	133.43
T-47D	89.86
MDA-MB-468	114.44

Bar Graph

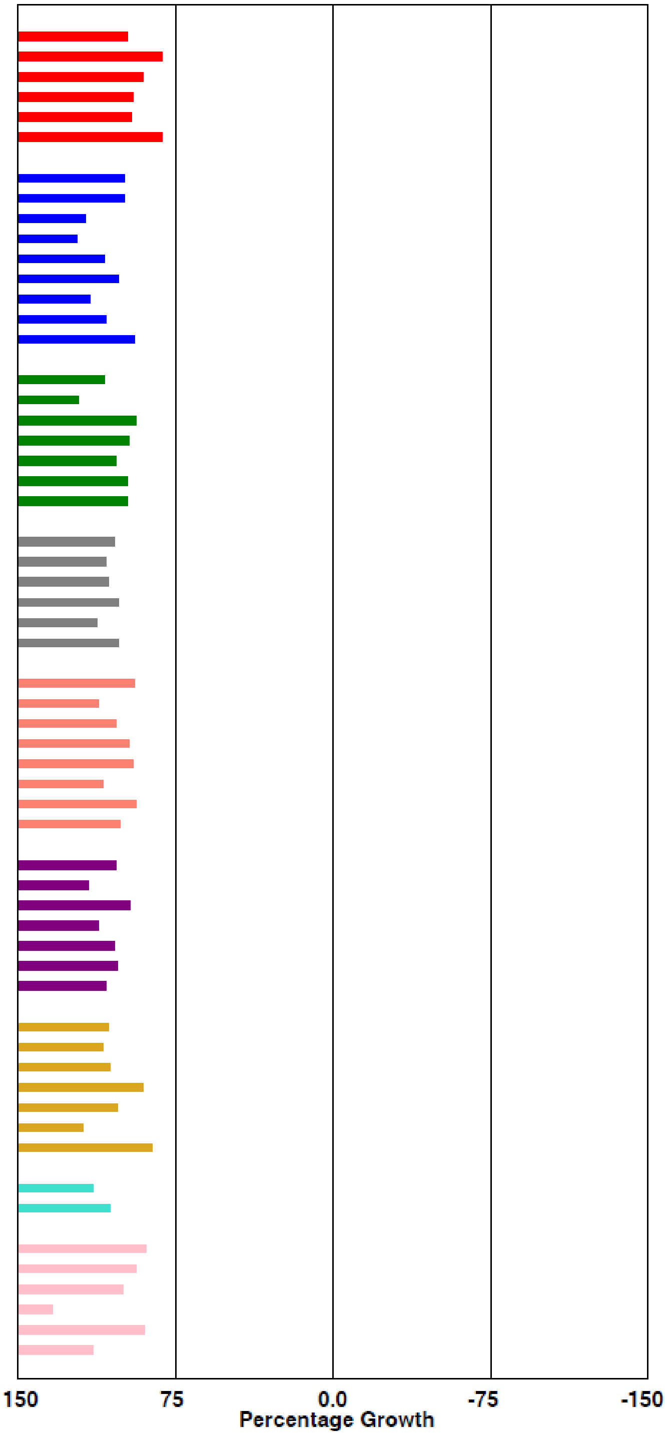

Compound (18)

Developmental Therapeutics Program One Dose Bar Graph	NSC: D-847379 / 1	Conc: 1.00E-5 Molar	Test Date: Aug 07, 2023
	Experiment ID: 2308OS53		Report Date: Oct 17, 2023

Panel/Cell Line	Growth Percent
**Leukemia**	
CCRF-CEM	100.65
HL-60(TB)	79.74
K-562	87.19
MOLT-4	93.52
RPMI-8226	106.48
SR	85.12
**Non-Small Cell Lung Cancer**	
A549/ATCC	92.23
EKVX	101.97
HOP-62	118.21
HOP-92	130.43
NCI-H226	110.30
NCI-H23	106.41
NCI-H322M	114.89
NCI-H460	102.22
NCI-H522	99.14
**Colon Cancer**	
COLO 205	112.39
HCC-2998	128.30
HCT-116	96.64
HCT-15	91.52
HT29	95.18
KM12	98.54
SW-620	101.97
**CNS Cancer**	
SF-268	108.38
SF-295	102.64
SF-539	109.44
SNB-19	110.46
SNB-75	106.95
U251	107.18
**Melanoma**	
LOX IMVI	102.07
MALME-3M	127.78
M14	90.92
MDA-MB-435	94.88
SK-MEL-2	96.40
SK-MEL-28	108.62
SK-MEL-5	96.91
UACC-257	108.07
**Ovarian Cancer**	
IGROV1	116.01
OVCAR-3	118.51
OVCAR-4	96.07
OVCAR-5	109.42
OVCAR-8	107.49
NCI/ADR-RES	101.14
SK-OV-3	108.06
**Renal Cancer**	
786-0	100.98
A498	104.82
ACHN	107.78
CAKI-1	96.01
SN12C	103.50
TK-10	112.91
UO-31	101.24
**Prostate Cancer**	
PC-3	115.82
DU-145	104.35
**Breast Cancer**	
MCF7	82.73
MDA-MB-231/ATCC	100.08
HS 578T	111.57
BT-549	120.23
T-47D	86.24
MDA-MB-468	115.98

Bar Graph

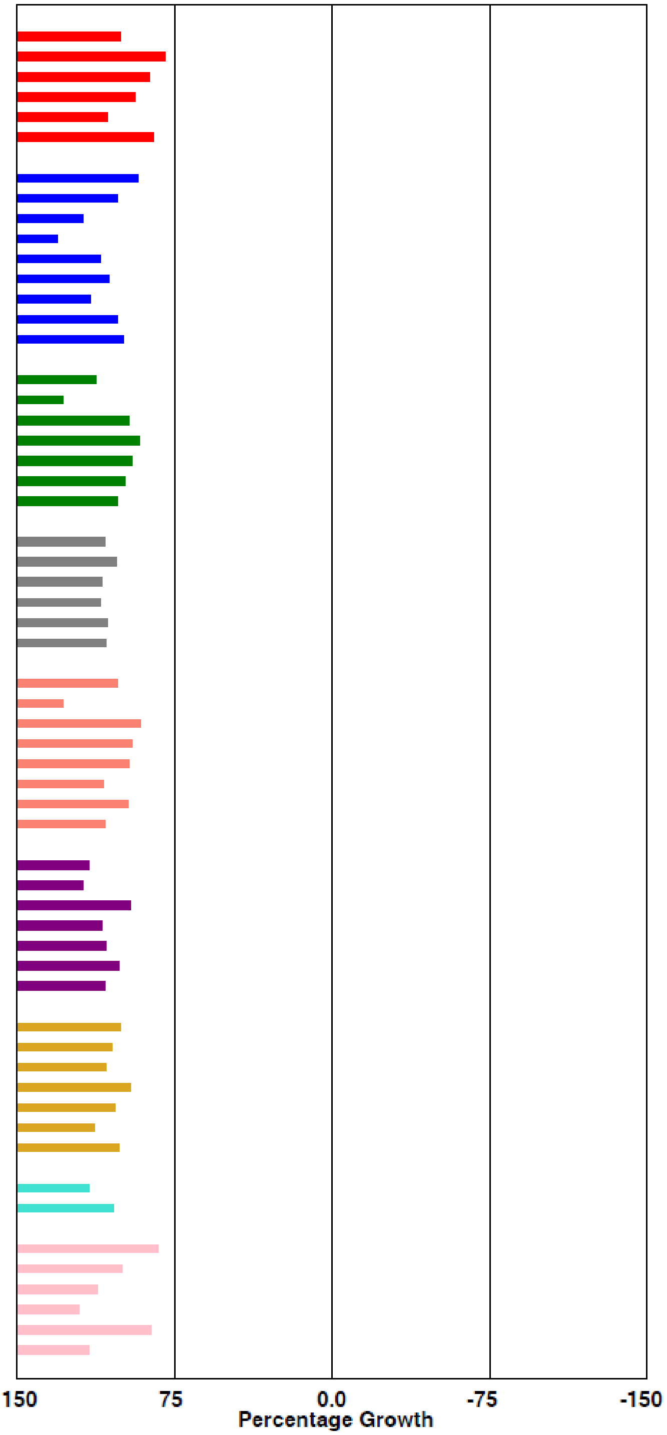

Compound (19)

Developmental Therapeutics Program One Dose Mean Graph	NSC: D-841403 / 1	Conc: 1.00E-5 Molar	Test Date: Jan 09, 2023
	Experiment ID: 2301OS86		Report Date: Feb 28, 2023

Panel/Cell Line	Growth Percent
**Leukemia**	
CCRF-CEM	90.93
HL-60(TB)	77.42
K-562	70.50
MOLT-4	76.23
RPMI-8226	89.94
SR	72.45
**Non-Small Cell Lung Cancer**	
A549/ATCC	84.14
EKVX	77.28
HOP-62	101.38
HOP-92	101.68
NCI-H23	91.24
NCI-H322M	95.08
NCI-H460	97.87
NCI-H522	79.03
**Colon Cancer**	
COLO 205	99.98
HCC-2998	110.63
HCT-116	76.17
HCT-15	75.48
HT29	67.52
KM12	94.28
SW-620	103.22
**CNS Cancer**	
SF-268	90.19
SF-295	95.84
SF-539	99.76
SNB-19	92.47
SNB-75	105.58
U251	89.80
**Melanoma**	
LOX IMVI	93.95
MALME-3M	106.70
M14	101.46
MDA-MB-435	96.08
SK-MEL-2	92.82
SK-MEL-28	107.29
SK-MEL-5	92.69
UACC-257	104.32
UACC-62	74.51
**Ovarian Cancer**	
IGROV1	104.46
OVCAR-3	131.00
OVCAR-4	95.72
OVCAR-5	99.39
OVCAR-8	89.28
NCI/ADR-RES	90.14
SK-OV-3	106.51
**Renal Cancer**	
786-0	95.91
A498	105.29
ACHN	103.34
CAKI-1	75.92
RXF 393	81.08
SN12C	81.49
TK-10	114.37
UO-31	76.35
**Prostate Cancer**	
PC-3	82.76
DU-145	95.42
**Breast Cancer**	
MCF7	88.38
MDA-MB-231/ATCC	82.74
HS 578T	95.05
BT-549	93.15
T-47D	75.37
MDA-MB-468	79.84
Mean	91.85
Delta	24.33
Range	63.48

Mean Growth Percent - Growth Percent

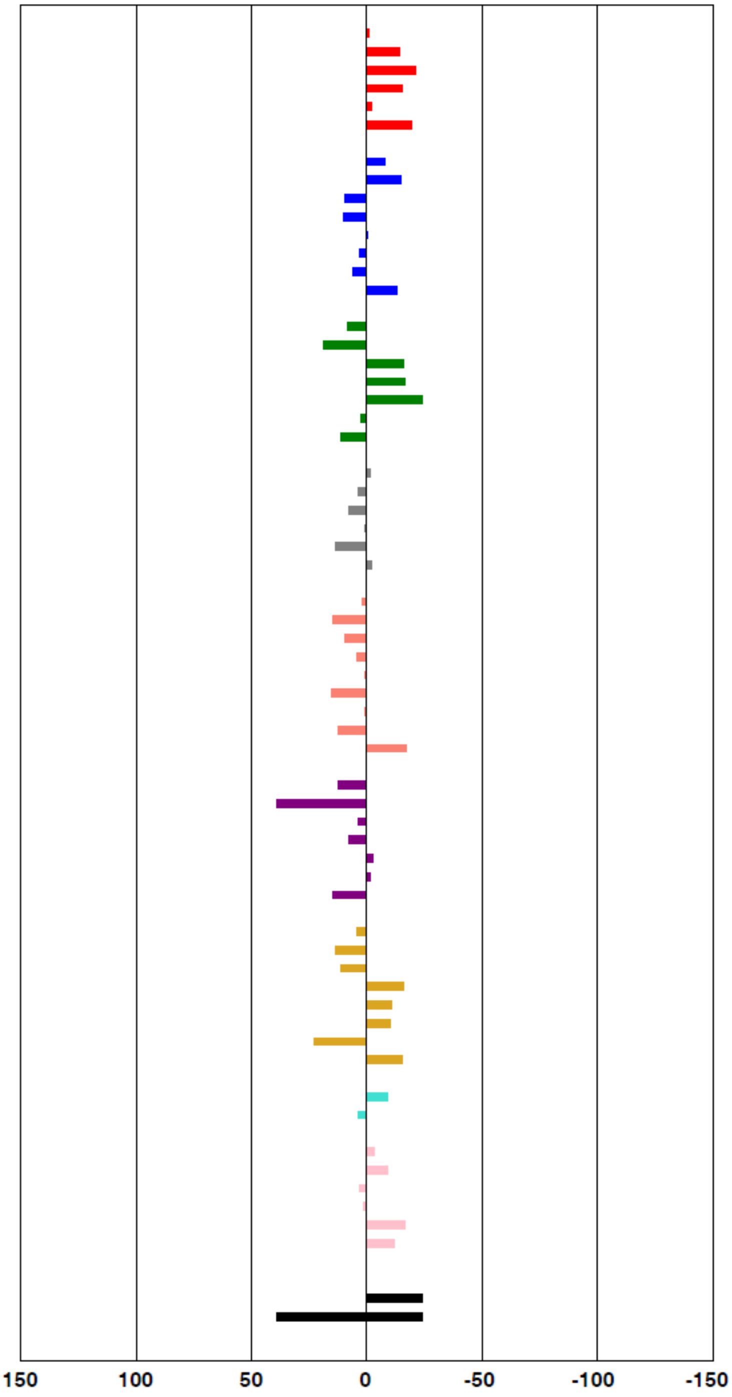

Compound (20)

Developmental Therapeutics Program

One Dose Mean Graph

**NSC:** D-841410 / 1	**Conc:** 1.00E-5 Molar	**Test Date:** Jan 09, 2023	
**Experiment ID:** 2301OS86		**Report Date:** Feb 28, 2023	

Panel/Cell Line	Growth Percent
Leukemia	
CCRF-CEM	96.39
HL-60(TB)	68.66
K-562	88.24
MOLT-4	87.51
RPMI-8226	95.47
SR	86.96
Non-Small Cell Lung Cancer	
A549/ATCC	98.07
EKVX	95.20
HOP-62	109.12
HOP-92	107.88
NCI-H23	98.57
NCI-H322M	102.96
NCI-H460	102.86
NCI-H522	91.51
Colon Cancer	
COLO 205	108.64
HCC-2998	97.63
HCT-116	94.53
HCT-15	94.42
HT29	91.92
KM12	101.92
SW-620	102.61
CNS Cancer	
SF-268	113.82
SF-295	96.81
SF-539	106.73
SNB-19	94.23
SNB-75	107.64
U251	105.17
Melanoma	
LOX IMVI	92.64
MALME-3M	109.66
M14	95.10
MDA-MB-435	98.18
SK-MEL-2	106.43
SK-MEL-28	109.06
SK-MEL-5	98.59
UACC-257	100.29
UACC-62	81.22
Ovarian Cancer	
IGROV1	106.39
OVCAR-3	122.92
OVCAR-4	94.35
OVCAR-5	113.99
OVCAR-8	99.08
NCI/ADR-RES	100.23
SK-OV-3	106.18
Renal Cancer	
786-0	101.72
A498	121.16
ACHN	106.30
CAKI-1	92.81
RXF 393	111.17
SN12C	93.84
TK-10	111.70
UO-31	86.82
Prostate Cancer	
PC-3	106.85
DU-145	106.12
Breast Cancer	
MCF7	87.49
MDA-MB-231/ATCC	101.68
HS 578T	105.82
BT-549	104.23
T-47D	78.49
MDA-MB-468	98.01
Mean	99.90
Delta	31.24
Range	54.26

Mean Growth Percent - Growth Percent

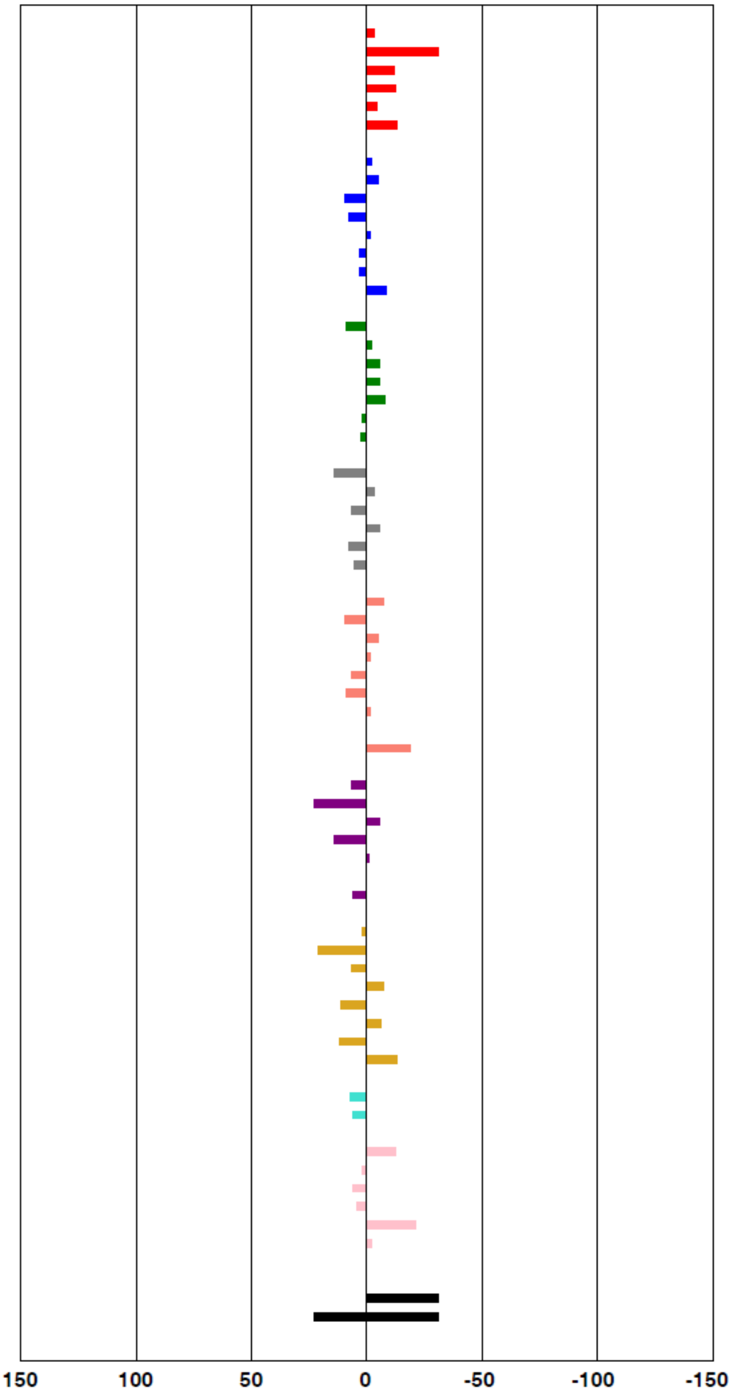

Compound (23)

Developmental Therapeutics Program

One Dose Bar Graph

**NSC:** D-847382 / 1	**Conc:** 1.00E-5 Molar	**Test Date:** Aug 07, 2023
**Experiment ID:** 2308OS53		**Report Date:** Oct 17, 2023

Panel/Cell Line	Growth Percent
Leukemia	
CCRF-CEM	82.07
HL-60(TB)	81.60
K-562	60.95
MOLT-4	73.32
RPMI-8226	79.95
SR	65.99
Non-Small Cell Lung Cancer	
A549/ATCC	80.21
EKVX	76.86
HOP-62	108.49
HOP-92	107.20
NCI-H226	94.40
NCI-H23	92.59
NCI-H322M	107.39
NCI-H460	88.61
NCI-H522	68.79
Colon Cancer	
COLO 205	91.87
HCC-2998	114.42
HCT-116	66.28
HCT-15	72.02
HT29	73.65
KM12	74.60
SW-620	98.50
CNS Cancer	
SF-268	88.48
SF-295	87.62
SF-539	84.30
SNB-19	96.33
SNB-75	105.00
U251	88.38
Melanoma	
LOX IMVI	84.98
MALME-3M	124.04
M14	99.73
MDA-MB-435	82.03
SK-MEL-2	91.05
SK-MEL-28	91.13
SK-MEL-5	75.52
UACC-257	89.98
Ovarian Cancer	
IGROV1	111.86
OVCAR-3	105.93
OVCAR-4	72.51
OVCAR-5	88.10
OVCAR-8	96.23
NCI/ADR-RES	85.10
SK-OV-3	97.06
Renal Cancer	
786-0	93.96
A498	102.29
ACHN	90.86
CAKI-1	76.00
SN12C	79.81
TK-10	113.02
UO-31	77.15
Prostate Cancer	
PC-3	84.33
DU-145	90.18
Breast Cancer	
MCF7	76.16
MDA-MB-231/ATCC	76.78
HS 578T	83.04
BT-549	95.21
T-47D	74.46
MDA-MB-468	93.23

Bar Graph

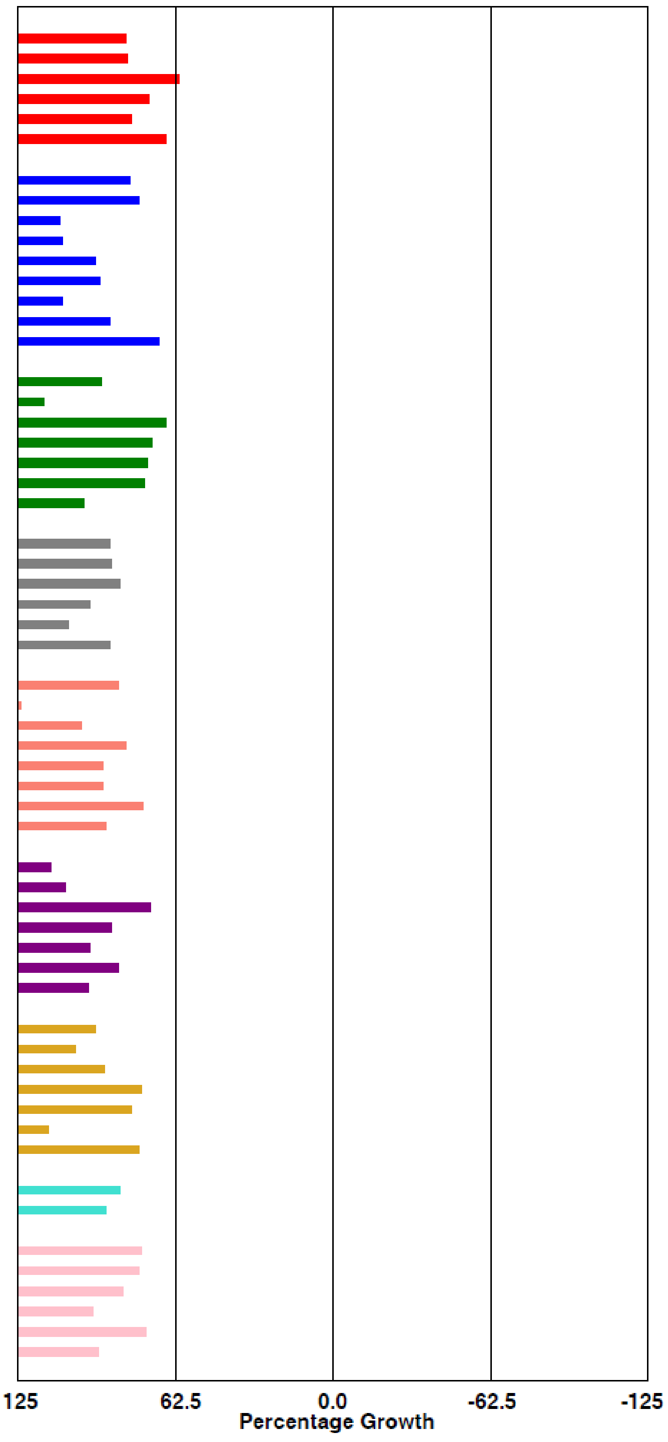

Compound (24)

Developmental Therapeutics Program One Dose Bar Graph	NSC: D-847381 / 1	Conc: 1.00E-5 Molar	Test Date: Aug 07, 2023
	Experiment ID: 2308OS53		Report Date: Oct 17, 2023

Panel/Cell Line	Growth Percent
**Leukemia**	
CCRF-CEM	80.70
HL-60(TB)	77.16
K-562	58.50
MOLT-4	63.04
RPMI-8226	73.63
SR	60.41
**Non-Small Cell Lung Cancer**	
A549/ATCC	76.96
EKVX	79.55
HOP-62	112.01
HOP-92	98.06
NCI-H226	98.33
NCI-H23	86.25
NCI-H322M	102.40
NCI-H460	97.64
NCI-H522	64.57
**Colon Cancer**	
COLO 205	95.56
HCC-2998	110.05
HCT-116	69.30
HCT-15	71.72
HT29	70.20
KM12	79.09
SW-620	96.29
**CNS Cancer**	
SF-268	83.14
SF-295	92.12
SF-539	92.11
SNB-19	96.99
SNB-75	93.64
U251	85.31
**Melanoma**	
LOX IMVI	88.15
MALME-3M	115.88
M14	96.31
MDA-MB-435	80.31
SK-MEL-2	88.07
SK-MEL-28	94.51
SK-MEL-5	71.06
UACC-257	86.49
**Ovarian Cancer**	
IGROV1	103.44
OVCAR-3	113.11
OVCAR-4	73.62
OVCAR-5	89.03
OVCAR-8	93.17
NCI/ADR-RES	82.51
SK-OV-3	100.59
**Renal Cancer**	
786-0	88.18
A498	98.05
ACHN	92.76
CAKI-1	72.48
SN12C	76.47
TK-10	109.86
UO-31	74.16
**Prostate Cancer**	
PC-3	73.61
DU-145	92.96
**Breast Cancer**	
MCF7	81.46
MDA-MB-231/ATCC	83.10
HS 578T	79.39
BT-549	100.52
T-47D	62.39
MDA-MB-468	88.71

Bar Graph

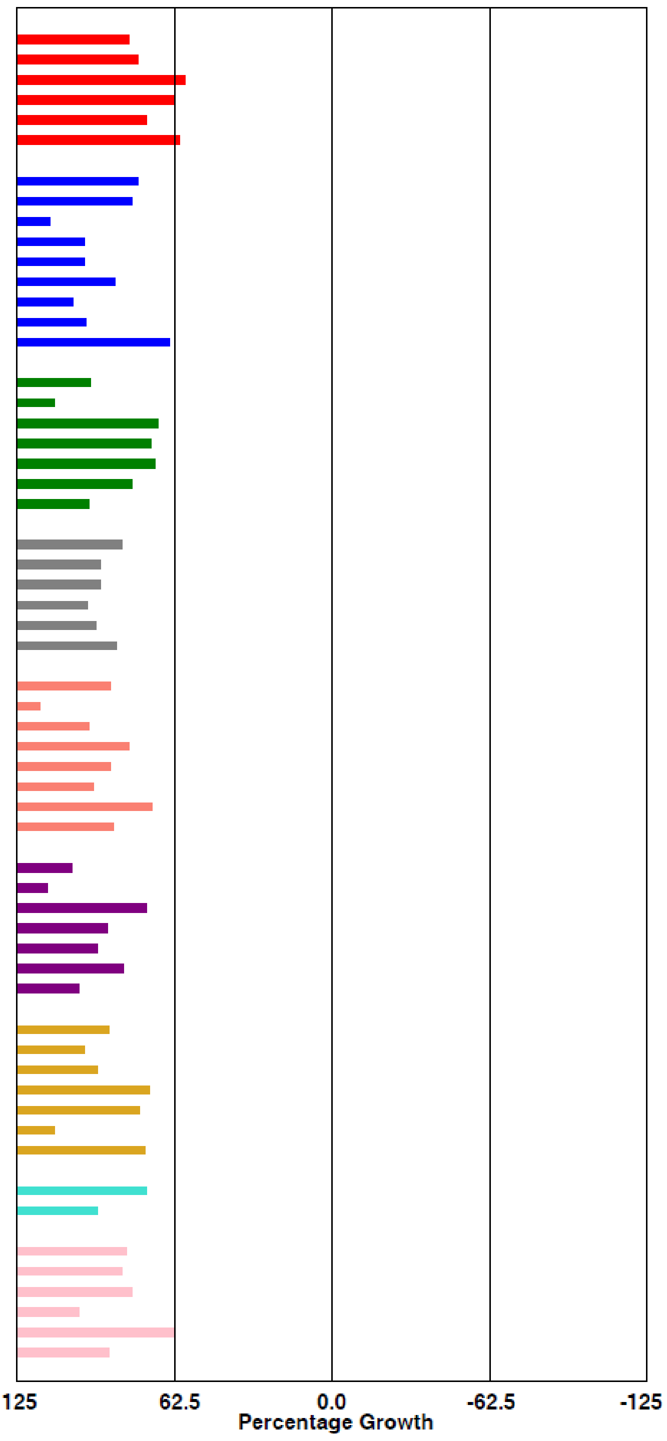

Compound (25)

Developmental Therapeutics Program One Dose Bar Graph	NSC: D-847380 / 1	Conc: 1.00E-5 Molar	Test Date: Aug 07, 2023
	Experiment ID: 2308OS53		Report Date: Oct 17, 2023

Panel/Cell Line	Growth Percent
Leukemia	
CCRF-CEM	88.78
HL-60(TB)	84.55
K-562	72.95
MOLT-4	80.89
RPMI-8226	89.18
SR	75.10
Non-Small Cell Lung Cancer	
A549/ATCC	82.71
EKVX	94.92
HOP-62	112.31
HOP-92	102.30
NCI-H226	97.27
NCI-H23	95.47
NCI-H322M	103.04
NCI-H460	92.00
NCI-H522	68.33
Colon Cancer	
COLO 205	95.75
HCC-2998	124.64
HCT-116	79.07
HCT-15	85.35
HT29	74.48
KM12	96.37
SW-620	93.15
CNS Cancer	
SF-268	94.35
SF-295	98.73
SF-539	107.34
SNB-19	97.36
SNB-75	92.48
U251	98.50
Melanoma	
LOX IMVI	92.67
MALME-3M	112.57
M14	99.67
MDA-MB-435	85.06
SK-MEL-2	82.57
SK-MEL-28	99.62
SK-MEL-5	75.20
UACC-257	96.77
Ovarian Cancer	
IGROV1	105.05
OVCAR-3	112.44
OVCAR-4	79.81
OVCAR-5	95.22
OVCAR-8	93.66
NCI/ADR-RES	88.77
SK-OV-3	97.06
Renal Cancer	
786-0	109.25
A498	109.89
ACHN	92.19
CAKI-1	86.50
SN12C	91.51
TK-10	114.98
UO-31	88.42
Prostate Cancer	
PC-3	91.82
DU-145	93.70
Breast Cancer	
MCF7	82.39
MDA-MB-231/ATCC	83.80
HS 578T	101.25
BT-549	112.55
T-47D	65.50
MDA-MB-468	103.68

Bar Graph

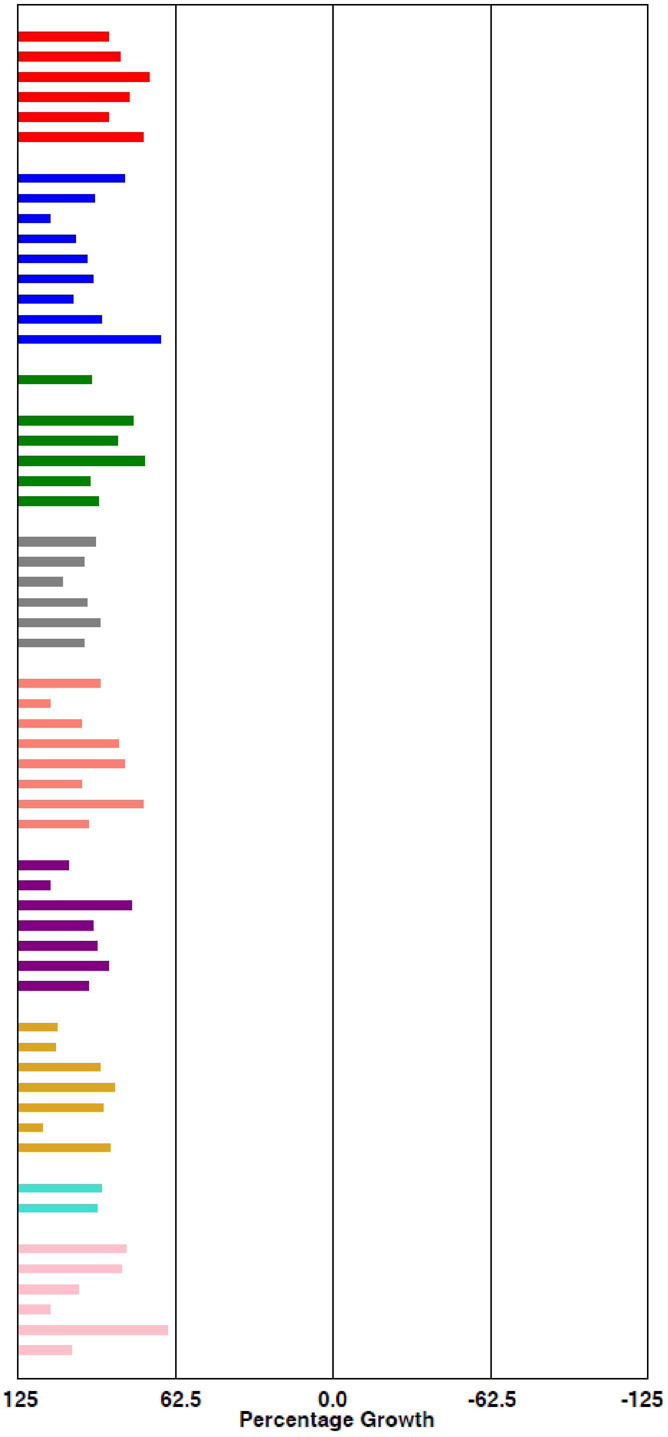

Compound (26)

Developmental Therapeutics Program One Dose Mean Graph	NSC: D-841404 / 1	Conc: 1.00E-5 Molar	Test Date: Jan 09, 2023
	Experiment ID: 2301OS86		Report Date: Feb 28, 2023

Panel/Cell Line	Growth Percent
Leukemia	
CCRF-CEM	98.92
HL-60(TB)	85.47
K-562	93.33
MOLT-4	94.49
RPMI-8226	99.47
SR	87.40
Non-Small Cell Lung Cancer	
A549/ATCC	99.98
EKVX	95.41
HOP-62	108.18
HOP-92	114.65
NCI-H23	100.29
NCI-H322M	98.99
NCI-H460	102.02
NCI-H522	90.95
Colon Cancer	
COLO 205	108.47
HCC-2998	121.80
HCT-116	97.16
HCT-15	95.27
HT29	86.93
KM12	98.28
SW-620	97.36
CNS Cancer	
SF-268	110.01
SF-295	101.99
SF-539	109.78
SNB-19	96.23
SNB-75	109.82
U251	102.10
Melanoma	
LOX IMVI	95.86
MALME-3M	104.39
M14	106.19
MDA-MB-435	98.75
SK-MEL-2	102.68
SK-MEL-28	111.62
SK-MEL-5	102.50
UACC-257	99.31
UACC-62	82.60
Ovarian Cancer	
IGROV1	104.32
OVCAR-3	119.07
OVCAR-4	101.05
OVCAR-5	126.08
OVCAR-8	95.80
NCI/ADR-RES	101.98
SK-OV-3	115.80
Renal Cancer	
786-0	102.74
A498	114.77
ACHN	105.95
CAKI-1	90.78
RXF 393	102.18
SN12C	91.95
TK-10	114.08
UO-31	84.82
Prostate Cancer	
PC-3	104.80
DU-145	103.70
Breast Cancer	
MCF7	81.95
MDA-MB-231/ATCC	95.50
HS 578T	112.48
BT-549	106.44
T-47D	85.84
MDA-MB-468	92.67
Mean	101.07
Delta	19.12
Range	44.13

Mean Growth Percent - Growth Percent

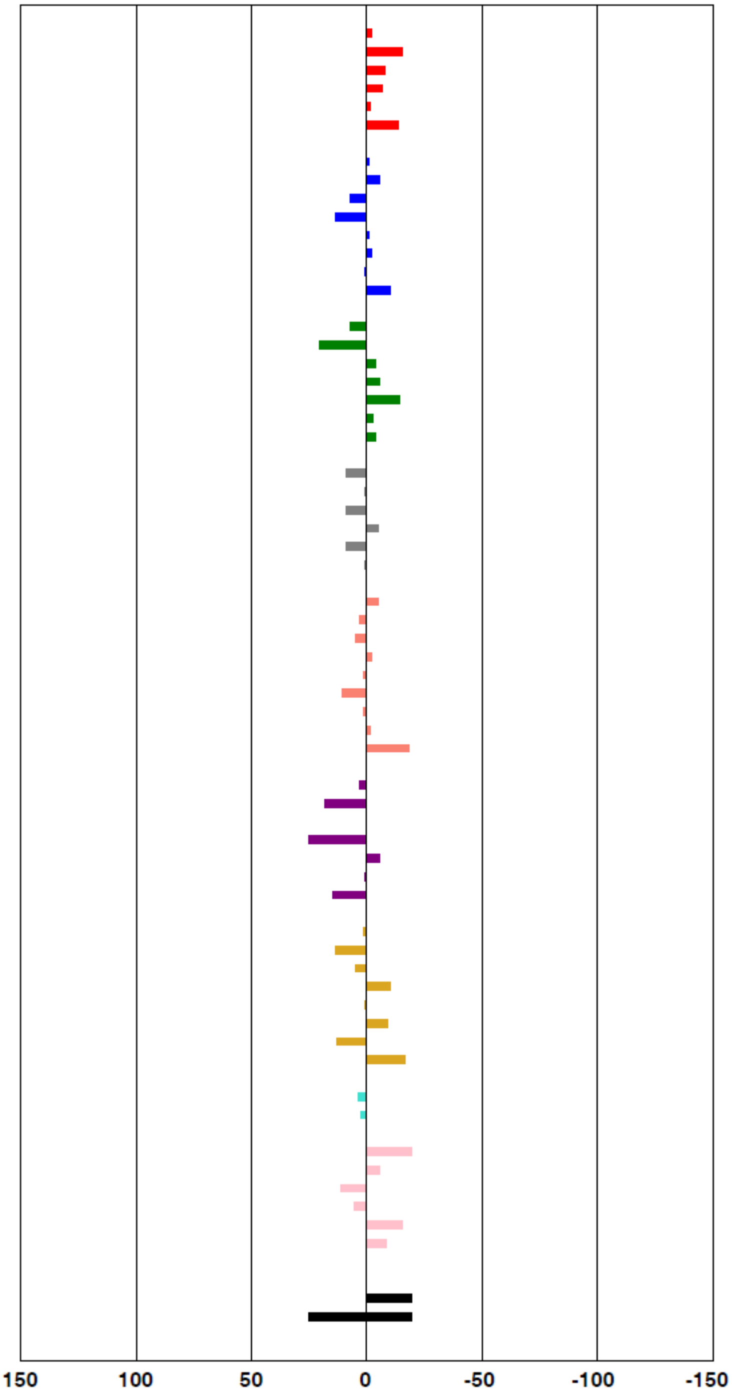

Compound (27)

Developmental Therapeutics Program<br>One Dose Mean Graph	NSC: D-841411 / 1	Conc: 1.00E-5 Molar	Test Date: Jan 09, 2023
	Experiment ID: 2301OS86		Report Date: Feb 28, 2023

Panel/Cell Line	Growth Percent
**Leukemia**	
CCRF-CEM	86.91
HL-60(TB)	70.75
K-562	58.03
MOLT-4	71.54
RPMI-8226	85.68
SR	69.69
**Non-Small Cell Lung Cancer**	
A549/ATCC	87.73
EKVX	87.84
HOP-62	105.83
HOP-92	89.36
NCI-H23	92.22
NCI-H322M	104.29
NCI-H460	93.30
NCI-H522	83.57
**Colon Cancer**	
COLO 205	105.09
HCC-2998	106.92
HCT-116	78.19
HCT-15	81.17
HT29	77.43
KM12	96.57
SW-620	98.30
**CNS Cancer**	
SF-268	102.53
SF-295	97.00
SF-539	100.83
SNB-19	91.61
SNB-75	111.39
U251	100.86
**Melanoma**	
LOX IMVI	92.89
MALME-3M	128.37
M14	99.64
MDA-MB-435	93.65
SK-MEL-2	99.48
SK-MEL-28	107.14
SK-MEL-5	87.82
UACC-257	108.63
UACC-62	74.04
**Ovarian Cancer**	
IGROV1	102.97
OVCAR-3	124.72
OVCAR-4	89.74
OVCAR-5	112.00
OVCAR-8	92.11
NCI/ADR-RES	78.21
SK-OV-3	102.81
**Renal Cancer**	
786-0	97.47
A498	114.45
ACHN	93.50
CAKI-1	86.86
RXF 393	96.93
SN12C	85.66
TK-10	118.95
UO-31	77.15
**Prostate Cancer**	
PC-3	84.30
DU-145	97.98
**Breast Cancer**	
MCF7	82.05
MDA-MB-231/ATCC	92.47
HS 578T	100.47
BT-549	96.09
T-47D	76.85
MDA-MB-468	76.33
Mean	93.33
Delta	35.30
Range	70.34

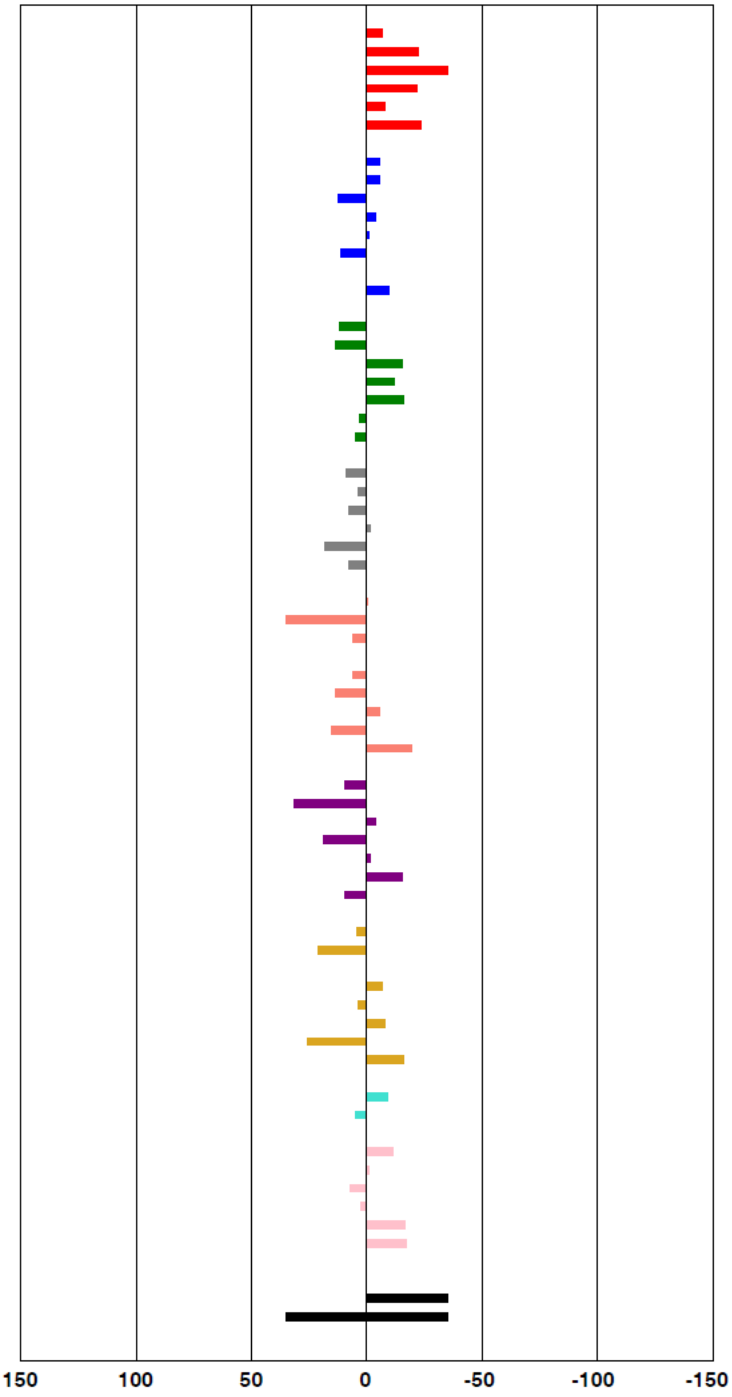

Compound (28)

Developmental Therapeutics Program One Dose Bar Graph	NSC: D-847383 / 1	Conc: 1.00E-5 Molar	Test Date: Aug 07, 2023
	Experiment ID: 2308OS53		Report Date: Oct 17, 2023

Panel/Cell Line	Growth Percent
Leukemia	
CCRF-CEM	82.63
HL-60(TB)	78.65
K-562	69.06
MOLT-4	67.19
RPMI-8226	75.74
SR	60.56
Non-Small Cell Lung Cancer	
A549/ATCC	81.14
EKVX	77.76
HOP-62	109.54
HOP-92	99.37
NCI-H226	97.80
NCI-H23	85.18
NCI-H322M	100.46
NCI-H460	91.59
NCI-H522	68.19
Colon Cancer	
COLO 205	96.51
HCC-2998	117.68
HCT-116	74.13
HCT-15	72.36
HT29	81.35
KM12	76.80
SW-620	97.21
CNS Cancer	
SF-268	89.18
SF-295	92.17
SF-539	82.17
SNB-19	95.94
SNB-75	115.75
U251	86.91
Melanoma	
LOX IMVI	91.68
MALME-3M	114.84
M14	99.20
MDA-MB-435	84.76
SK-MEL-2	87.96
SK-MEL-28	96.00
SK-MEL-5	69.92
UACC-257	84.72
Ovarian Cancer	
IGROV1	108.10
OVCAR-3	117.45
OVCAR-4	78.52
OVCAR-5	93.67
OVCAR-8	91.30
NCI/ADR-RES	85.73
SK-OV-3	97.09
Renal Cancer	
786-0	92.05
A498	99.15
ACHN	86.92
CAKI-1	74.32
SN12C	76.89
TK-10	114.81
UO-31	75.21
Prostate Cancer	
PC-3	81.03
DU-145	91.81
Breast Cancer	
MCF7	87.06
MDA-MB-231/ATCC	77.05
HS 578T	81.85
BT-549	96.36
T-47D	67.04
MDA-MB-468	93.48

Bar Graph

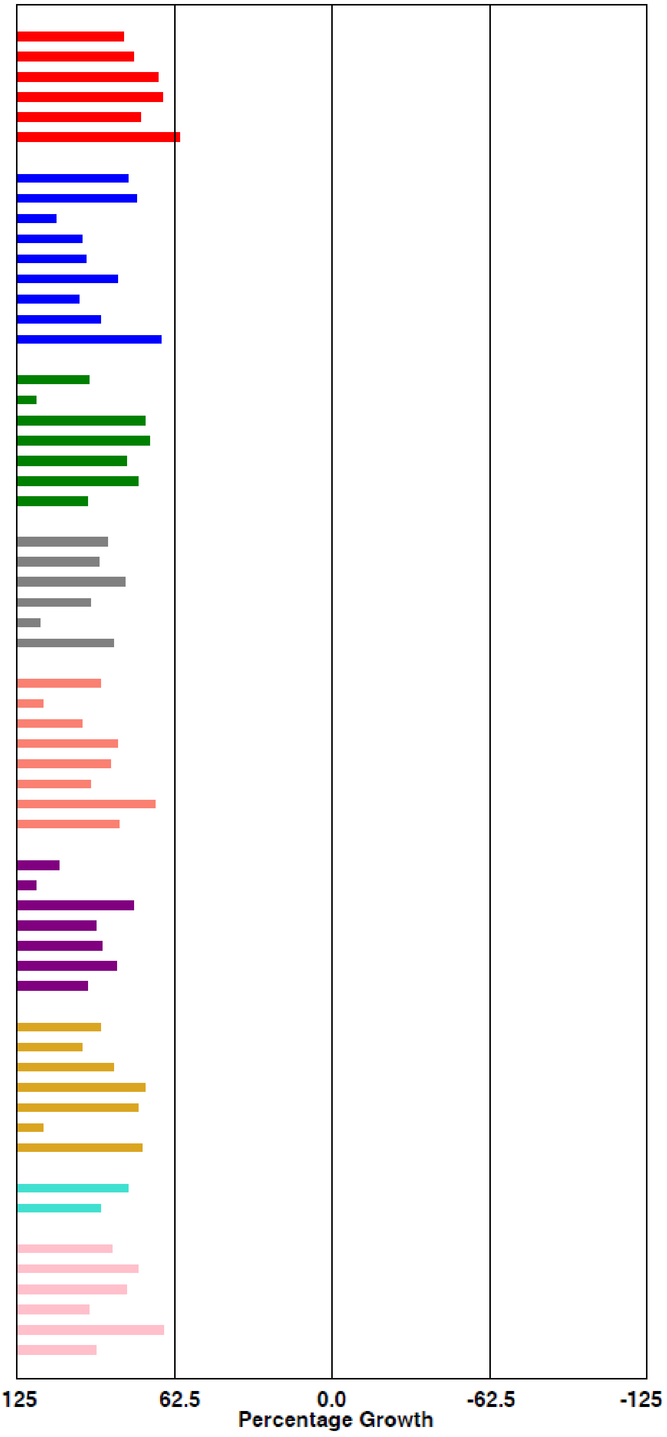

Compound (29)

Developmental Therapeutics Program
One Dose Mean Graph

**NSC:** D-841408 / 1	**Conc:** 1.00E-5 Molar	**Test Date:** Jan 09, 2023	
**Experiment ID:** 2301OS86		**Report Date:** Feb 28, 2023	

Panel/Cell Line	Growth Percent
**Leukemia**	
CCRF-CEM	85.83
HL-60(TB)	61.16
K-562	45.46
MOLT-4	53.50
RPMI-8226	63.10
SR	54.20
**Non-Small Cell Lung Cancer**	
A549/ATCC	69.93
EKVX	70.33
HOP-62	83.60
HOP-92	79.38
NCI-H23	85.44
NCI-H322M	82.59
NCI-H460	78.80
NCI-H522	76.86
**Colon Cancer**	
COLO 205	88.65
HCC-2998	103.02
HCT-116	69.77
HCT-15	57.85
HT29	61.98
KM12	75.23
SW-620	87.30
**CNS Cancer**	
SF-268	67.02
SF-295	83.26
SF-539	85.34
SNB-19	85.79
SNB-75	98.07
U251	76.71
**Melanoma**	
LOX IMVI	79.11
MALME-3M	95.05
M14	80.67
MDA-MB-435	77.23
SK-MEL-2	86.27
SK-MEL-28	88.72
SK-MEL-5	77.42
UACC-257	99.15
UACC-62	61.31
**Ovarian Cancer**	
IGROV1	93.86
OVCAR-3	105.21
OVCAR-4	78.08
OVCAR-5	92.62
OVCAR-8	80.58
NCI/ADR-RES	67.89
SK-OV-3	86.08
**Renal Cancer**	
786-0	88.38
A498	101.78
ACHN	86.76
CAKI-1	76.85
RXF 393	75.77
SN12C	64.43
TK-10	101.18
UO-31	66.91
**Prostate Cancer**	
PC-3	63.56
DU-145	71.45
**Breast Cancer**	
MCF7	72.63
MDA-MB-231/ATCC	69.66
HS 578T	62.53
BT-549	78.56
T-47D	65.43
MDA-MB-468	59.79
Mean	77.71
Delta	32.25
Range	59.75

Mean Growth Percent - Growth Percent

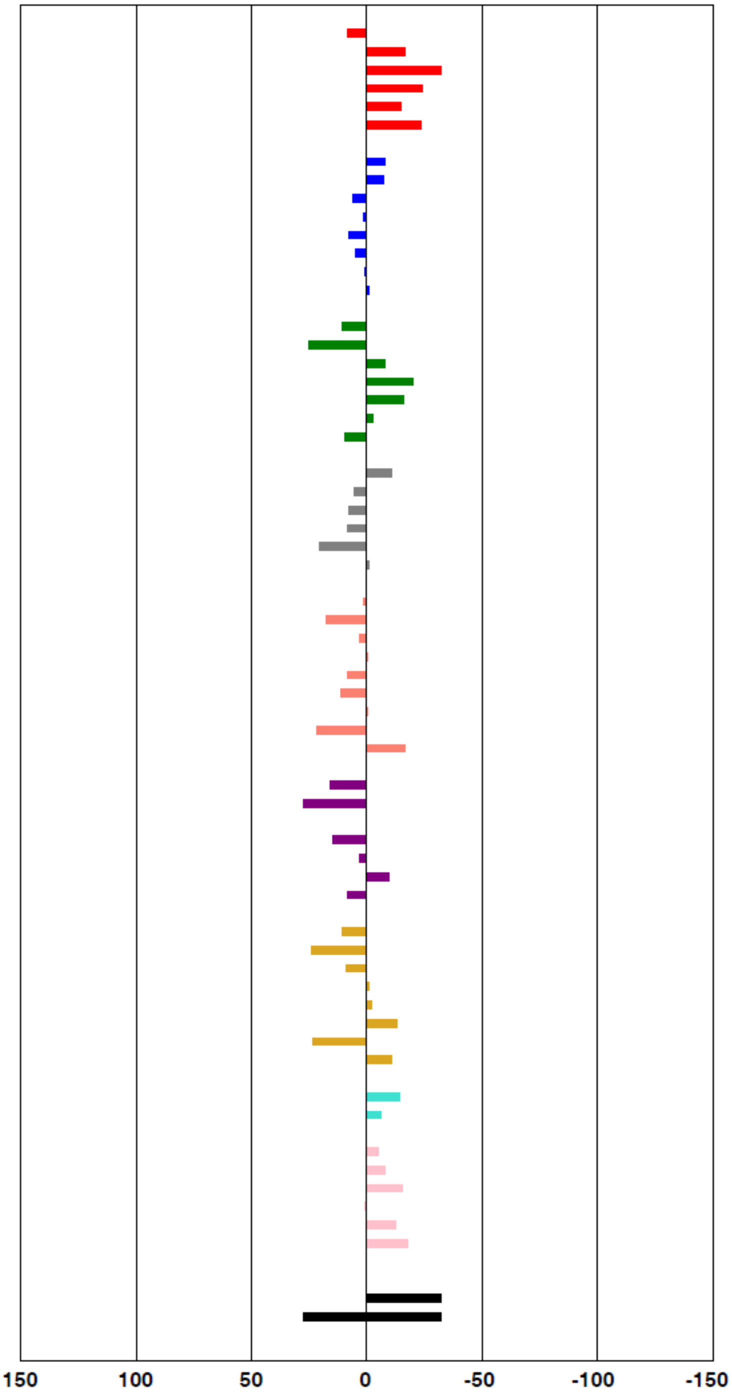

Section 3. Wound Healing Assay

a. Wound Width and Wound Closure % Calculations:

Table S3. Wound Healing Activity of Compounds **26** & **27**

Cpd	Wound Width (mm)±SD				Migration rate	Wound Closure %		
	0 h	24 h	48 h	72 h		24 h	48 h	72 h
**Control**	0.96±0.01	0.62±0.01	0.25±0.03	0	0.014	36.55	74.5	100
**26**	1.01±0.02	0.76±0.04	0.51±0.03	0	0.013	25	49.7	100
**27**	1±0.02	0.88±0.03	0.66±0.03	0.2±0.18	0.011	11.3	34	80

b. Wound Closure Images:

<u>Compound 26</u>

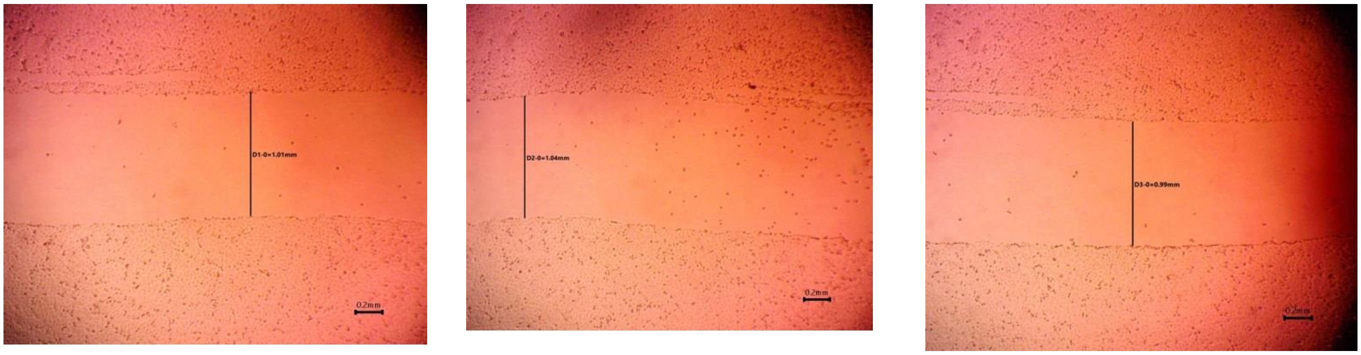

0 hr

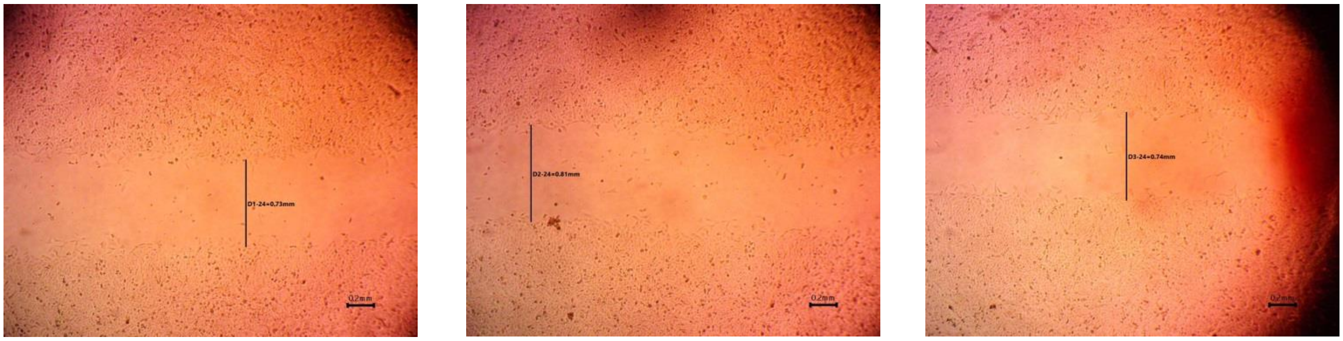

24 hr

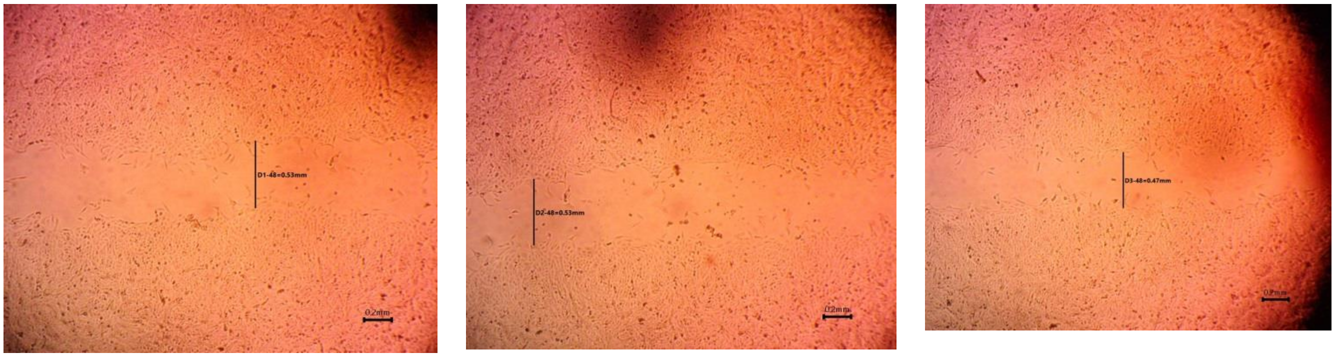

48 hr

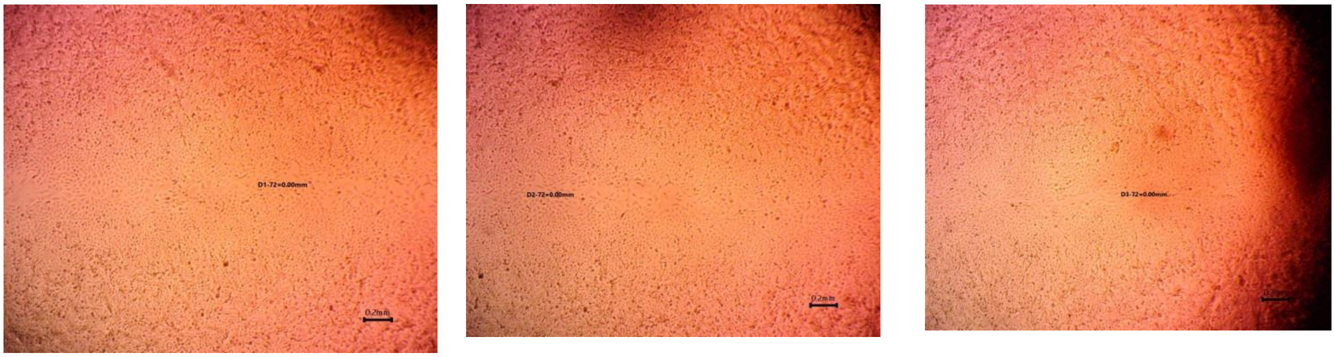

72 hr

<u>**Compound 27**</u>

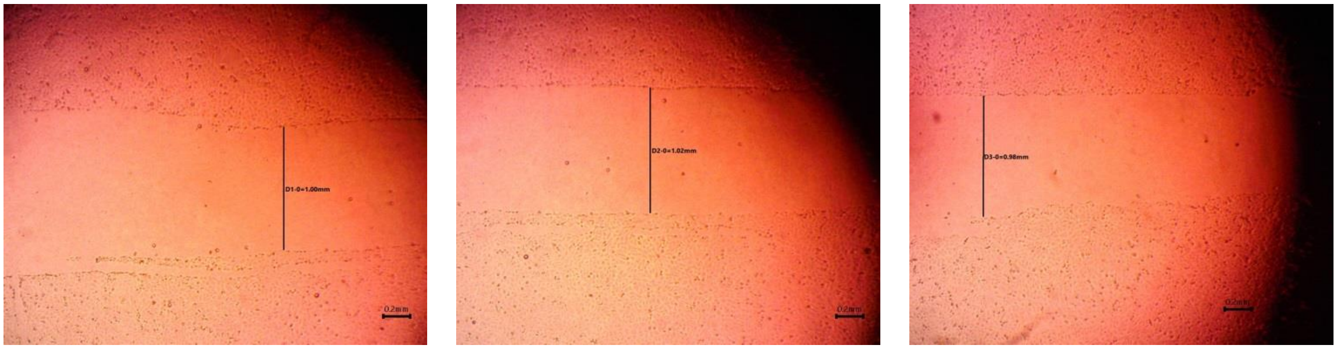

0 hr

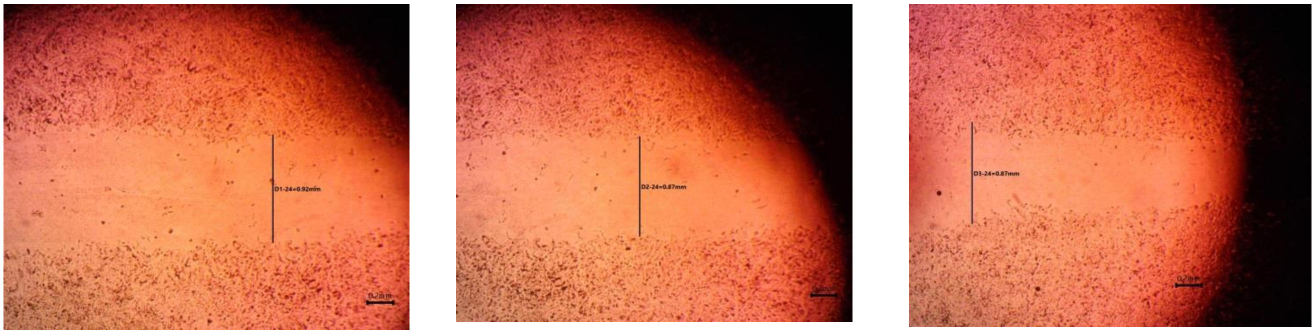

24 hr

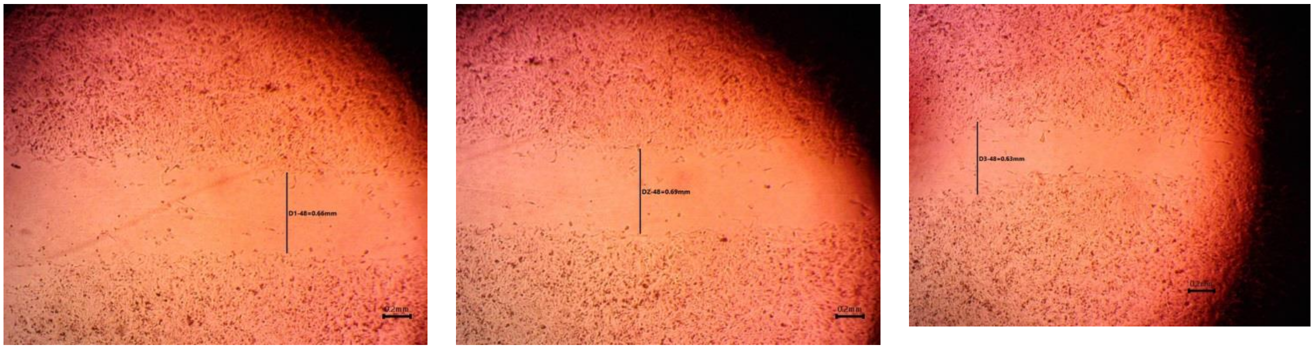

48 hr

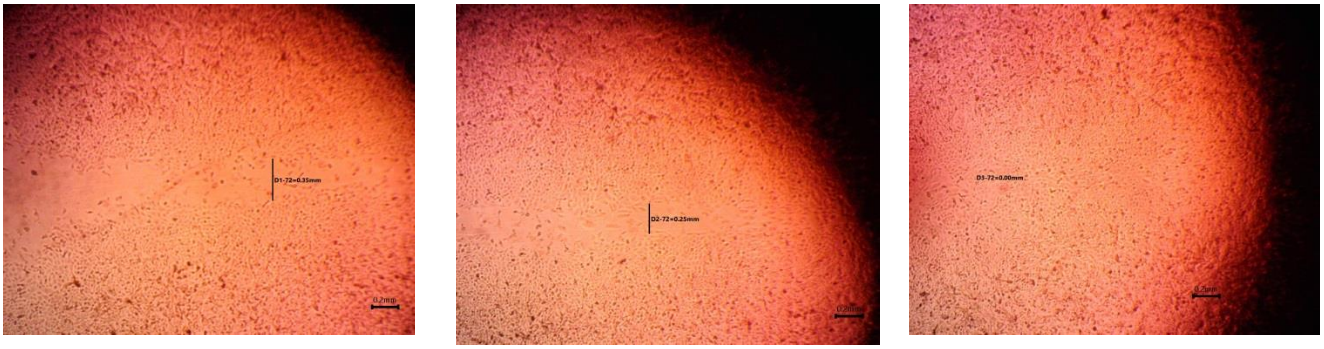

72 hr

<u>Control</u>

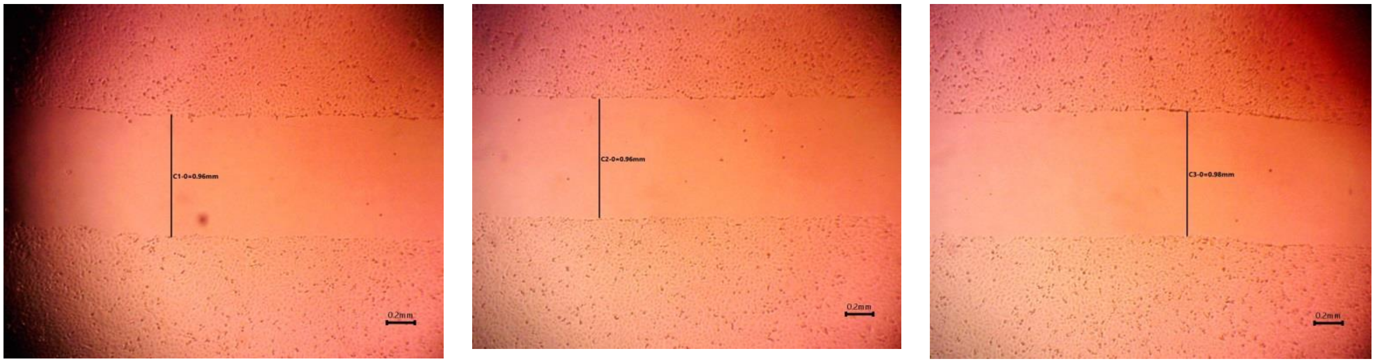

0 hr

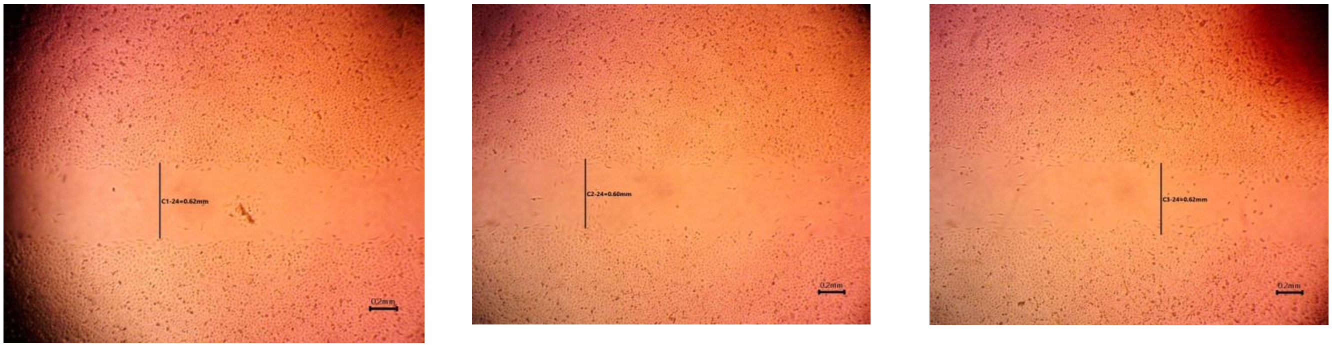

24 hr

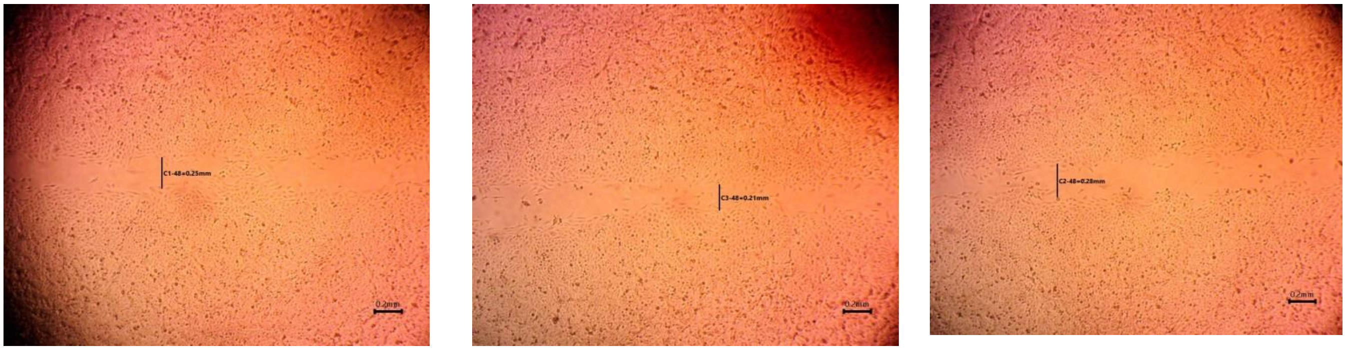

48 hr

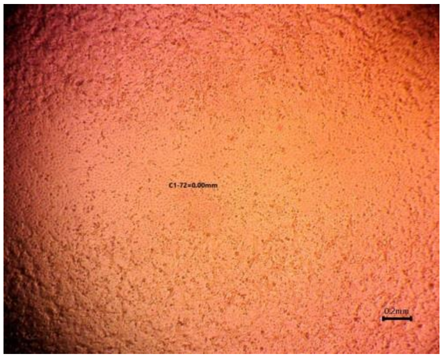

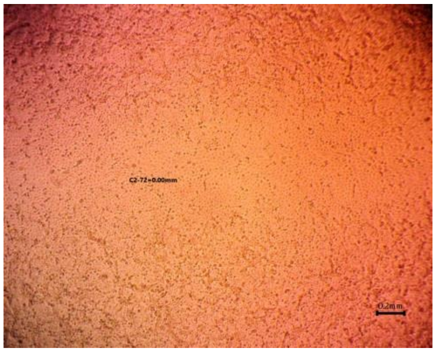

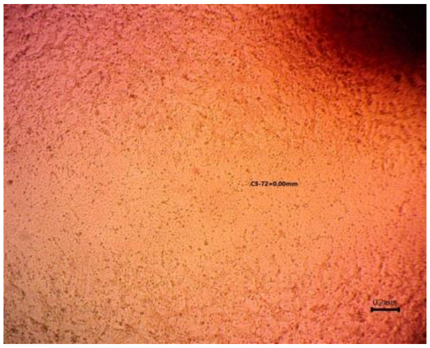

72 hr

Section 4. Determination of Partition Coefficient (lipophilicity measurements) Experiment

Table S4. Experimental Lipophilicity Determination

Cpd	R_{M0} ± SD	b ± SD	r	C_0	C range (%)
**14**	7.024367 ± 0.05913	-0.074 ± 0.00066	- 0.9999	91.3417	70-95
**19**	5.9262 ± 0.0676	-0.0676 ± 0.00075	- 0.9861	87.6579	70-90
**22**	6.448667 ± 0.05873	-0.0713 ± 0.00067	- 0.9999	90.6598	70-95
**26**	5.7961 ± 0.04654	-0.0669 ± 0.00054	- 0.9999	86.518	70-90
**27**	5.322167 ± 0.04242	-0.0637 ± 0.00049	- 0.9999	87.9561	70-90
**28**	7.152333 ± 0.02029	-0.078 ± 0.00025	- 0.9991	91.4199	70-95

The R_{M0} Values, C (the percentage of MeOH), b (Slope) and r (Correlation Coefficient) of the Equation $R_M = R_{M0} + bC$ for the studied compounds

Section 5. ADME Calculations

Table S5. Physicochemical Descriptors of Test Compounds

Cpd	MW (g/mol)[a]	Lipinski Parameters			TPSA[e] (Å^2)	F[f]
		HBA[b]	HBD[c]	nrotb[d]		
**14**	418.94	3	0	6	60.31	0.55
**19**	366.48	4	0	6	69.54	0.55
**22**	444.55	5	0	8	78.77	0.55
**26**	400.92	4	0	6	69.54	0.55
**27**	398.91	4	0	7	69.54	0.55
**28**	448.96	4	0	7	69.54	0.55
**Drug lead-like characteria**		≤10	≤5	≤10	≤140	> 0

[a] **MW**, Molecular weight. [b] **HBA**, H-bond acceptor. [c] **HBD**, H-bond donor. [d] **nrotb**, no. of rotatable bonds. [e]**TPSA**, topological polar surface area. [f]**F**, Abbott bioavailability scores

Section 6. X-Ray Coordinates of Compound 14

Table S6. Crystal data and structure refinement for $C_{24}H_{19}ClN_2OS$

Empirical formula	**$C_{24}H_{19}ClN_2OS$**
Formula weight	418.92
Temperature	RT
Wavelength	0.71073 Å
Crystal system	Orthorhombic
Space group	$P2_12_12_1$
Unit cell dimensions	a = 6.193(2) Å, b = 14.250(5) Å, c = 23.313(8) Å
	α = 90°, β = 90°, γ = 90°
Volume	2057.4(12) $Å^3$
Z	4
Density (calculated)	1.352 g/cm^3
Absorption coefficient	0.305 mm^{-1}
F(000)	872
Crystal size	0.260 x 0.055 x 0.020 mm^3
Theta range for data collection	1.675 to 24.969°.
Index ranges	-7<=h<=3, -16<=k<=16, -26<=l<=27
Reflections collected	15482
Independent reflections	3261 [R(int) = 0.0539]
Completeness to theta = 24.969°	92.7 %
Absorption correction	Semi-empirical from equivalents
Max. and min. transmission	0.7451 and 0.6868
Refinement method	Full-matrix least-squares on F^2
Data / restraints / parameters	3261 / 1 / 263
Goodness-of-fit on F^2	1.127
Final R indices [I>2sigma(I)]	R1 = 0.0557, wR2 = 0.1118
R indices (all data)	R1 = 0.1021, wR2 = 0.1273
Absolute structure parameter	-0.09(13)
Extinction coefficient	0.0028(10)
Largest diff. peak and hole	0.285 and -0.351 e.$Å^{-3}$

Table S7. Atomic coordinates (x 10^4) and equivalent isotropic displacement parameters ($Å^2$x 10^3) for $C_{24}H_{19}ClN_2OS$

	x	y	z	U(eq)
S(1)	3560(3)	4229(1)	3456(1)	79(1)
Cl(1)	14549(4)	8638(2)	3516(1)	165(1)
O(1)	-2447(10)	7011(5)	464(4)	85(2)
C(1)	12315(11)	7948(6)	3351(3)	75(2)
C(2)	11048(11)	8274(5)	2918(3)	77(2)
C(3)	9216(11)	7756(6)	2754(3)	76(2)
C(4)	8688(10)	6924(5)	3014(3)	60(2)
C(5)	10033(11)	6594(5)	3453(3)	72(2)
C(6)	11830(12)	7105(6)	3615(3)	82(2)
C(7)	6741(11)	6384(5)	2845(3)	60(2)
C(8)	5510(12)	6593(5)	2367(3)	64(2)
C(9)	3676(11)	6074(4)	2251(2)	57(2)
N(10)	3144(9)	5339(4)	2583(2)	63(2)
C(11)	4453(13)	5183(5)	3032(3)	65(2)
N(12)	6201(9)	5652(4)	3186(2)	64(1)
C(13)	2202(10)	6293(5)	1776(2)	59(2)
C(14)	2080(11)	7178(5)	1540(3)	80(2)
C(15)	562(13)	7397(6)	1123(3)	92(3)
C(16)	-843(12)	6721(6)	932(3)	73(2)
C(17)	-725(12)	5843(6)	1154(3)	87(2)
C(18)	778(12)	5638(5)	1577(3)	86(2)
C(19)	5346(12)	4301(5)	4074(2)	77(2)
C(20)	4669(12)	5046(5)	4506(3)	60(2)
C(21)	2728(14)	5037(7)	4757(3)	98(3)
C(22)	2140(14)	5691(8)	5162(4)	117(3)
C(23)	3585(17)	6376(6)	5326(4)	98(3)
C(24)	5531(17)	6381(6)	5082(3)	94(3)
C(25)	6109(12)	5740(6)	4662(3)	81(2)
C(26)	-3610(19)	6594(8)	397(5)	138(6)
H(2)	11390	8832	2733	93
H(3)	8339	7985	2462	91

H(5)	9717	6031	3636	87
H(6)	12723	6879	3904	98
H(8)	5915	7080	2124	77
H(14)	3037	7640	1662	96
H(15)	495	8001	973	111
H(17)	-1653	5377	1022	104
H(18)	817	5036	1731	103
H(19A)	5380	3695	4264	92
H(19B)	6797	4438	3942	92
H(21)	1743	4574	4654	117
H(22)	770	5670	5325	140
H(23)	3211	6823	5599	117
H(24)	6534	6829	5198	113
H(25)	7455	5777	4487	97
H(26A)	-4511	6841	98	165
H(26B)	-3057	5993	283	165
H(26C)	-4442	6527	742	165

U(eq) is defined as one third of the trace of the orthogonalized U^{ij} tensor.

Table S8. Anisotropic displacement parameters (Å^{2}x 10^3) for $C_{24}H_{19}ClN_2OS$

	U^{11}	U^{22}	U^{33}	U^{23}	U^{13}	U^{12}
S(1)	119(2)	58(1)	60(1)	4(1)	-14(1)	-2(1)
Cl(1)	107(2)	220(3)	169(3)	-75(3)	-27(2)	30(2)
O(1)	68(4)	81(4)	106(5)	31(4)	-13(3)	0(3)
C(1)	70(5)	82(5)	73(5)	-24(4)	3(4)	19(4)
C(2)	71(5)	87(6)	75(5)	6(4)	0(4)	1(4)
C(3)	75(5)	87(6)	66(5)	4(4)	-8(4)	7(5)
C(4)	66(4)	63(4)	51(4)	-8(3)	1(4)	11(4)
C(5)	80(5)	80(5)	57(4)	-5(4)	-11(4)	25(4)
C(6)	71(5)	104(6)	71(5)	-20(5)	-16(4)	29(5)
C(7)	66(4)	63(4)	51(4)	-8(3)	0(4)	21(4)
C(8)	74(5)	72(5)	48(4)	3(3)	0(4)	4(4)
C(9)	73(4)	56(4)	41(4)	-7(3)	-2(3)	9(4)
N(10)	85(4)	56(3)	48(3)	-4(3)	0(3)	8(3)
C(11)	89(5)	55(4)	50(4)	1(3)	-1(4)	13(4)
N(12)	77(4)	58(3)	56(3)	-4(3)	-2(3)	14(3)
C(13)	75(5)	61(4)	41(3)	5(3)	-1(3)	-4(4)
C(14)	85(5)	90(6)	66(5)	12(4)	-19(4)	-10(4)
C(15)	104(6)	86(6)	88(6)	27(5)	-27(5)	-13(5)
C(16)	70(5)	91(6)	59(4)	9(4)	-4(4)	-5(5)
C(17)	103(6)	78(5)	80(5)	13(5)	-31(5)	-22(5)
C(18)	106(6)	75(5)	78(5)	19(4)	-25(5)	-8(5)
C(19)	111(5)	60(4)	59(4)	10(4)	-20(4)	12(4)
C(20)	76(5)	57(4)	47(4)	5(3)	-8(4)	4(4)
C(21)	88(6)	125(7)	80(6)	-33(6)	3(5)	-20(5)
C(22)	86(6)	158(9)	106(7)	-34(7)	6(5)	-11(7)
C(23)	116(7)	94(6)	82(6)	-26(5)	-13(6)	11(6)
C(24)	124(8)	83(6)	76(6)	-13(5)	-6(6)	-35(6)
C(25)	92(5)	86(5)	65(4)	-4(4)	5(4)	-11(5)
C(26)	182(14)	185(15)	46(5)	-7(8)	-27(8)	115(11)

The anisotropic displacement factor exponent takes the form: $-2\pi^2[h^2 a^{*2}U^{11} + ... + 2 h k a^* b^* U^{12}]$

Table S9. Bond lengths [Å] and angles [°] for $C_{24}H_{19}ClN_2OS$

S(1)-C(11)	1.770(7)
S(1)-C(19)	1.818(6)
Cl(1)-C(1)	1.740(8)
O(1)-C(26)	0.946(12)
O(1)-C(16)	1.532(10)
C(1)-C(2)	1.360(9)
C(1)-C(6)	1.382(10)
C(2)-C(3)	1.406(9)
C(2)-H(2)	0.9300
C(3)-C(4)	1.372(9)
C(3)-H(3)	0.9300
C(4)-C(5)	1.401(8)
C(4)-C(7)	1.484(9)
C(5)-C(6)	1.382(10)
C(5)-H(5)	0.9300
C(6)-H(6)	0.9300
C(7)-N(12)	1.354(8)
C(7)-C(8)	1.383(8)
C(8)-C(9)	1.382(9)
C(8)-H(8)	0.9300
C(9)-N(10)	1.343(7)
C(9)-C(13)	1.470(8)
N(10)-C(11)	1.342(8)
C(11)-N(12)	1.322(8)
C(13)-C(18)	1.364(9)
C(13)-C(14)	1.379(9)
C(14)-C(15)	1.387(9)
C(14)-H(14)	0.9300
C(15)-C(16)	1.372(9)
C(15)-H(15)	0.9300
C(16)-C(17)	1.355(9)
C(17)-C(18)	1.388(9)
C(17)-H(17)	0.9300
C(18)-H(18)	0.9300
C(19)-C(20)	1.522(9)
C(19)-H(19A)	0.9700

C(19)-H(19B)	0.9700
C(20)-C(21)	1.337(10)
C(20)-C(25)	1.381(9)
C(21)-C(22)	1.376(11)
C(21)-H(21)	0.9300
C(22)-C(23)	1.379(12)
C(22)-H(22)	0.9300
C(23)-C(24)	1.332(11)
C(23)-H(23)	0.9300
C(24)-C(25)	1.387(10)
C(24)-H(24)	0.9300
C(25)-H(25)	0.9300
C(26)-H(26A)	0.9600
C(26)-H(26B)	0.9600
C(26)-H(26C)	0.9600

C(11)-S(1)-C(19)	102.0(4)
C(26)-O(1)-C(16)	116.3(12)
C(2)-C(1)-C(6)	120.1(7)
C(2)-C(1)-Cl(1)	115.4(7)
C(6)-C(1)-Cl(1)	124.5(6)
C(1)-C(2)-C(3)	119.2(7)
C(1)-C(2)-H(2)	120.4
C(3)-C(2)-H(2)	120.4
C(4)-C(3)-C(2)	121.7(7)
C(4)-C(3)-H(3)	119.1
C(2)-C(3)-H(3)	119.1
C(3)-C(4)-C(5)	118.1(7)
C(3)-C(4)-C(7)	121.6(6)
C(5)-C(4)-C(7)	120.2(7)
C(6)-C(5)-C(4)	120.1(7)
C(6)-C(5)-H(5)	120.0
C(4)-C(5)-H(5)	120.0
C(1)-C(6)-C(5)	120.8(7)
C(1)-C(6)-H(6)	119.6
C(5)-C(6)-H(6)	119.6
N(12)-C(7)-C(8)	120.2(7)
N(12)-C(7)-C(4)	116.4(6)

C(8)-C(7)-C(4)	123.4(6)
C(9)-C(8)-C(7)	119.6(6)
C(9)-C(8)-H(8)	120.2
C(7)-C(8)-H(8)	120.2
N(10)-C(9)-C(8)	120.5(6)
N(10)-C(9)-C(13)	116.6(6)
C(8)-C(9)-C(13)	122.9(6)
C(11)-N(10)-C(9)	115.5(6)
N(12)-C(11)-N(10)	128.5(6)
N(12)-C(11)-S(1)	119.5(5)
N(10)-C(11)-S(1)	112.0(6)
C(11)-N(12)-C(7)	115.6(6)
C(18)-C(13)-C(14)	117.0(6)
C(18)-C(13)-C(9)	120.8(6)
C(14)-C(13)-C(9)	122.0(6)
C(13)-C(14)-C(15)	121.5(7)
C(13)-C(14)-H(14)	119.3
C(15)-C(14)-H(14)	119.3
C(16)-C(15)-C(14)	119.9(7)
C(16)-C(15)-H(15)	120.0
C(14)-C(15)-H(15)	120.0
C(17)-C(16)-C(15)	119.4(7)
C(17)-C(16)-O(1)	123.7(7)
C(15)-C(16)-O(1)	116.9(7)
C(16)-C(17)-C(18)	120.1(7)
C(16)-C(17)-H(17)	120.0
C(18)-C(17)-H(17)	120.0
C(13)-C(18)-C(17)	122.1(7)
C(13)-C(18)-H(18)	119.0
C(17)-C(18)-H(18)	119.0
C(20)-C(19)-S(1)	113.3(5)
C(20)-C(19)-H(19A)	108.9
S(1)-C(19)-H(19A)	108.9
C(20)-C(19)-H(19B)	108.9
S(1)-C(19)-H(19B)	108.9
H(19A)-C(19)-H(19B)	107.7
C(21)-C(20)-C(25)	118.2(7)
C(21)-C(20)-C(19)	122.0(7)

C(25)-C(20)-C(19)	119.7(7)
C(20)-C(21)-C(22)	122.1(8)
C(20)-C(21)-H(21)	118.9
C(22)-C(21)-H(21)	118.9
C(21)-C(22)-C(23)	119.9(8)
C(21)-C(22)-H(22)	120.0
C(23)-C(22)-H(22)	120.0
C(24)-C(23)-C(22)	118.2(8)
C(24)-C(23)-H(23)	120.9
C(22)-C(23)-H(23)	120.9
C(23)-C(24)-C(25)	122.1(8)
C(23)-C(24)-H(24)	119.0
C(25)-C(24)-H(24)	119.0
C(20)-C(25)-C(24)	119.4(7)
C(20)-C(25)-H(25)	120.3
C(24)-C(25)-H(25)	120.3
O(1)-C(26)-H(26A)	109.5
O(1)-C(26)-H(26B)	109.5
H(26A)-C(26)-H(26B)	109.5
O(1)-C(26)-H(26C)	109.5
H(26A)-C(26)-H(26C)	109.5
H(26B)-C(26)-H(26C)	109.5

Symmetry transformations used to generate equivalent atoms:

Table S10. Intra hydrogen bonds Donor --- H .... Acceptor

	D - H	H ... A	D ... A	D – H ... A
C5 --- H5 ...N12	0.93	2.48	2.797(9)	100
C19 --- H19B ... N12	0.97	2.50	2.876(8)	103
C18 --- H18 ...N10	0.93	2.49	2.798(9)	99.34

Table S11. Analysis of Short Ring-Interactions with Cg-Cg Distances less than 5.0 Å

Cg1 - Cg2	3.703(4)	Cg2 - Cg3	4.473(4)
Cg1 - Cg3	4.842(4)	Cg4 - Cg3	4.609(5)

With Cg(I) = Plane number (I), Cg-Cg is the distance between ring Centroids Å

Cg1 made of C7, C8, C9, N10, C11 and N12

Cg2 made of C1, C2, C3, C4, C5 and C6

Cg3 made of C13, C14, C15, C16, C17 and C18

Cg4 made of C20, C21, C22, C23, C24 and C25

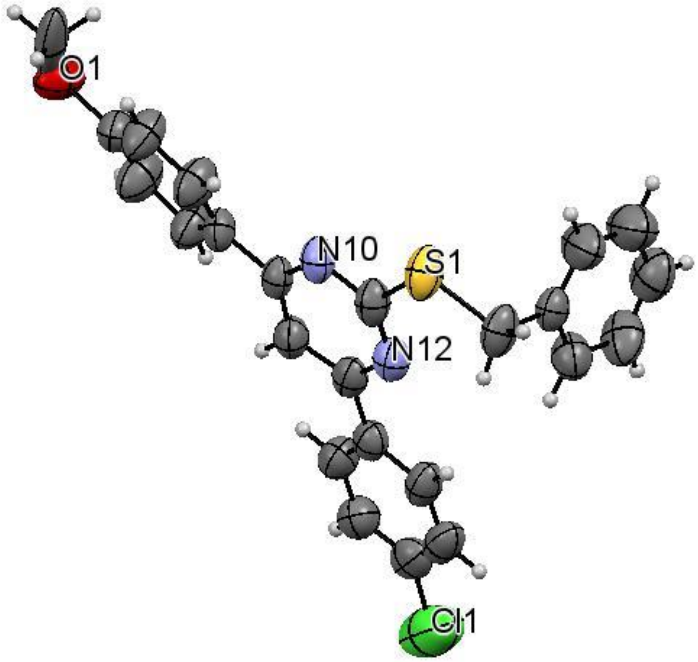
O1
N10
S1
N12
Cl1

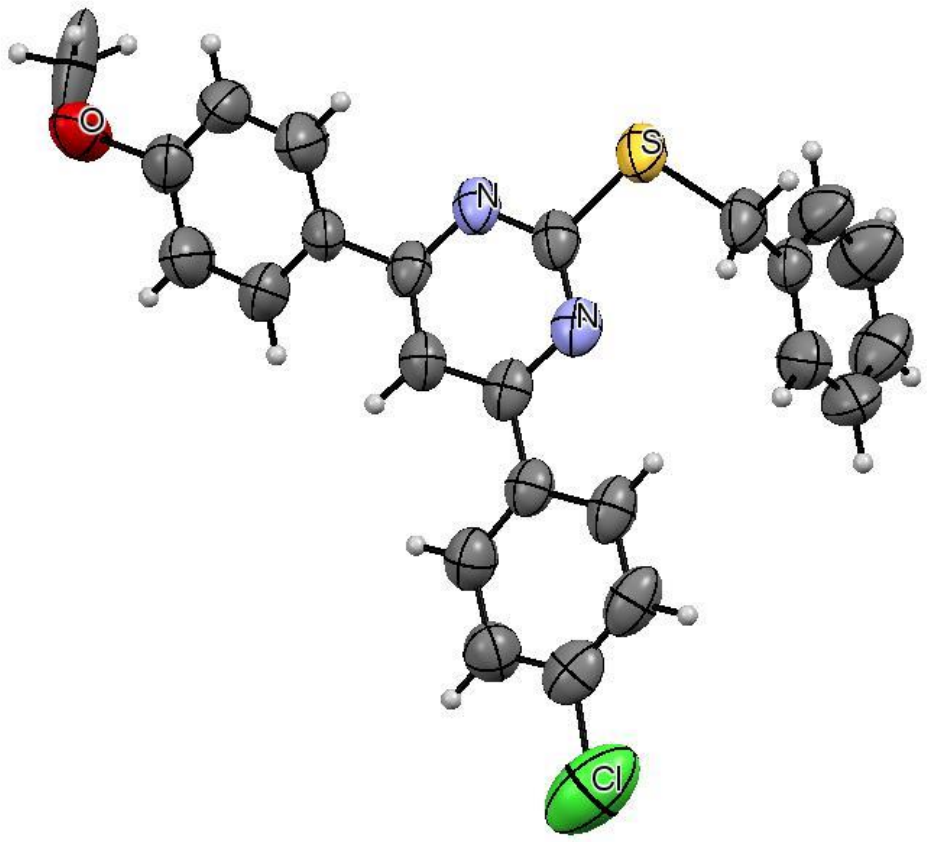
O
N
N
S
Cl

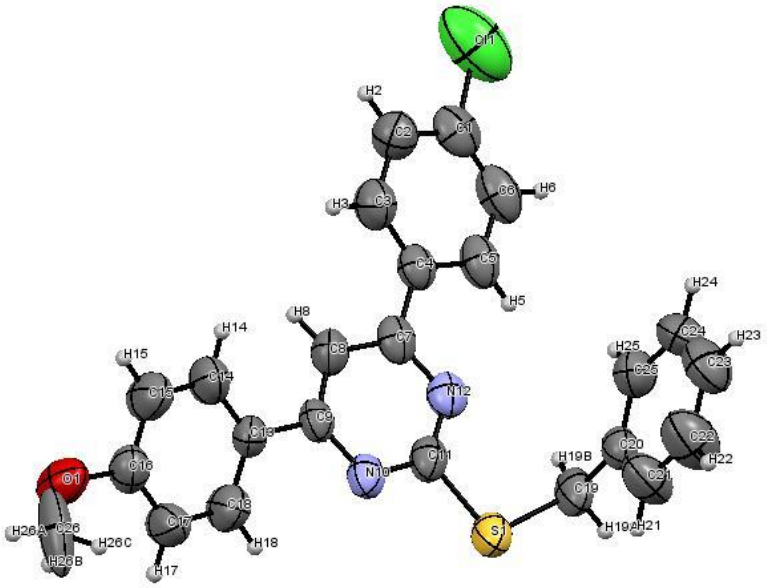
Cl1
H2
C2
C1
H6
C6
H3
C3
C4
C5
H5
H24
H8
H14
C7
C24
H23
H15
C8
H25
C25
C23
C14
C15
N12
C13
C9
C22
H19B
C20
C16
O1
N10
C11
H22
C21
C19
C18
H26A
C26
H26C
S1
H19A
H21
H26B
H18
H17

Appendix A

4. EXPERIMENTAL

4.1. Chemistry

General details:

All commercially available reagents and some of the solvents were used without further purification. Melting points were determined on a Fisher-Johns melting point apparatus and are uncorrected. ^{1}H and ^{13}C NMR spectra were recorded on a Bruker Avance 300 spectrometer. For NMR spectra obtained using $DMSO\text{-}d_6$ and $CDCl_3$ as the solvents, chemical shifts (ppm) for ^{1}H NMR spectra are reported relative to internal Me_4Si (TMS), chemical shifts for ^{13}C spectra are relative to the residual solvent peak (central peak). Low-resolution (LRMS) and high-resolution (HRMS) electrospray ionization (ESI) mass spectra were obtained with a Waters/Micromass QTOF Ultima Global mass spectrometer.

4.2. Biological evaluation

4.2.1. Cytotoxic activity using MTT Assay and evaluation of IC_{50}

4.2.1.1. MTT assay

MTT assay was carried out to study the effect of compounds on mammary epithelial cells (MCF-10A). The medium in which cells were propagated contained Dulbecco's modified Eagle's medium (DMEM)/ Ham's F-12 medium (1:1) supplemented with epidermal growth factor (20 ng/mL), hydrocortisone (500 ng/mL), insulin (10 µg/mL), 2 mM glutamine and 10% foetal calf serum. After every 2-3 days, the cells were passaged using trypsin ethylenediamine tetra acetic acid (EDTA). The cells were seeded at a density of 10^4 cells mL^{-1} in flat-bottomed culture plates containing 96 wells each. After 24 h, medium was removed from the plates and the compounds in

(in 0.1% DMSO) were added (in 200 µL medium to yield a final concentration of 0.1% v/v) to the wells of plates. A single compound was designated with four wells followed by incubation of plates for 96h at 37°C. After incubation, medium was removed completely from the plates followed by addition of MTT (0.4 mg/mL in medium) to each well and subsequent incubation of plates for 3h. MTT (along with the medium) was removed and DMSO (150µL) was added to each well of the culture plates, followed by vertexing and subsequent measurement of absorbance (at 540 nm) using microplate reader. The data are shown as percentage inhibition of proliferation in comparison with controls containing 0.1% DMSO.

4.2.1.2. Assay for antiproliferative effect

To explore the antiproliferative potential of compounds MTT assay was performed according to previously reported procedure using different cell lines to explore the antiproliferative potential of compounds propidium iodide fluorescence assay was performed using different cell lines. To calculate the total nuclear DNA, a fluorescent dye (propidium iodide, PI) is used which can attach to the DNA, thus offering a quick and precise technique. PI cannot pass through the cell membrane and its signal intensity can be considered as directly proportional to quantity of cellular DNA. Cells whose cell membranes are damaged or have changed permeability are counted as dead ones. The assay was performed by seeding the cells of different cell lines at a density of 3000-7500 cells/well (in 200µl medium) in culture plates followed by incubation for 24h at 37 °C in humidified 5% CO_2/95% air atmospheric conditions. The medium was removed; the compounds were added to the plates at 10 µM concentrations (in 0.1% DMSO) in triplicates, followed by incubation for 48 h. DMSO (0.1%) was used as control. After incubation, medium was removed followed by the addition of PI (25 µl, 50µg/mL in water/medium) to each well of the plates. At -80 °C, the plates were allowed to freeze for 24 h, followed by thawing at 25°C. A fluorometer

(Polar-Star BMG Tech) was used to record the readings at excitation and emission wavelengths of 530 and 620 nm for each well. The percentage cytotoxicity of compounds was calculated using the following formula:

$$\% \text{ Cytotoxicity} = \frac{A_C - A_{TC}}{A_C} \times 100$$

Where A_{TC}= Absorbance of treated cells and A_C= Absorbance of control. Erlotinib was used as positive control in the assay.

4.2.1.3. EGFR inhibitory assay

EGFR-TK assay was performed to evaluate the inhibitory potency of the most potent compounds against EGFR. Baculoviral expression vectors including pBlueBacHis2B and pFASTBacHTc were used separately to clone 1.6 kb cDNA coding for EGFR cytoplasmic domain (EGFR-CD, amino acids 645–1186). 5′ upstream to the EGFR sequence comprised a sequence that encoded $(His)_6$. Sf-9 cells were infected for 72h for protein expression. The pellets of Sf-9 cells were solubilized in a buffer containing sodium vanadate (100 µM), aprotinin (10 µg/mL), triton (1%), HEPES buffer (50mM), ammonium molybdate (10 µM), benzamidine HCl (16 µg/mL), NaCl (10 mM), leupeptin (10 µg/mL) and pepstatin (10 µg/mL) at 0°C for 20 min at pH 7.4, followed by centrifugation for 20 min. To eliminate the non-specifically bound material, a Ni-NTA super flow packed column was used to pass through and wash the crude extract supernatant first with 10 mM and then with 100 mM imidazole. Histidine-linked proteins were first eluted with 250 and then with 500 mM imidazole subsequent to dialysis against NaCl (50 mM), HEPES (20 mM), glycerol (10%) and 1 µg/mL each of aprotinin, leupeptin and pepstatin for 120 min. The purification was performed either at 4 °C or on ice. To record autophosphorylation level, EGFR kinase assay was carried out on the basis of DELFIA/Time-Resolved Fluorometry. The compounds were first

dissolved in DMSO absolute, subsequent to dilution to appropriate concentration using HEPES (25 mM) at pH 7.4. Each compound (10 µL) was incubated with recombinant enzyme (10 µL, 5 ng for EGFR, 1:80 dilution in 100 mM HEPES) for 10 min at 25°C, subsequent to the addition of 5X buffer (10 µL, containing 2 mM $MnCl_2$, 100 µM Na_3VO_4, 20 mM HEPES and 1 mM DTT) and ATP-$MgCl_2$ (20 µL, containing 0.1 mM ATP and 50 mM $MgCl_2$) and incubation for 1h. The negative and positive controls were included in each plate by the incubation of enzyme either with or without ATP-$MgCl_2$. The liquid was removed after incubation and the plates were washed thrice using wash buffer. Europium-tagged antiphosphotyrosine antibody (75 µL, 400 ng) was added to each well followed by incubation of 1h and then washing of the plates using buffer. The enhancement solution was added to each well and the signal was recorded at excitation and emission wavelengths of 340 at 615 nm. The autophosphorylation percentage inhibition by compounds was calculated using the following equation:

$$100\% - [(negative\ control)/(positive\ control) - (negative\ control)]$$

Using the curves of percentage inhibition of eight concentrations of each compound, IC_{50} was calculated. Majority of signals detected by antiphosphotyrosine antibody were from EGFR because the enzyme preparation contained low impurities.

4.2.1.4. BRAF kinase assay

V^{600E} mutant BRAF kinase assay was performed to investigate the activity of tested compounds against BRAF. Mouse full-length GST-tagged $BRAF^{V600E}$ (7.5 ng, Invitrogen, PV3849) was pre-incubated with drug (1 µL) and assay dilution buffer (4 µL) for 60 min at 25°C. In assay dilution buffer, a solution (5 µL) containing $MgCl_2$ (30 mM), ATP (200 µM), recombinant human full length (200 ng) and *N*-terminal His-tagged MEK1 (Invitrogen) was added to start the assay,

subsequent to incubation for 25 min at 25°C. The assay was stopped using 5X protein denaturing buffer (LDS) solution (5 µL). To further denature the protein, heat (70° C) was applied for 5 min. 4-12% precast NuPage gel plates (Invitrogen) were used to carry out electrophoresis (at 200 V). 10 µL of each reaction was loaded into the precast plates and electrophoresis was allowed to proceed. After completion of electrophoresis, the front part of the precast gel plate (holding hot ATP) was cut and afterwards cast-off. Dried gel was developed using a phosphor screen. A reaction without active enzyme was used as negative control while that containing no inhibitor served as positive control. To study the effect of compounds on cell-based pERK1/2 activity in cancer cells, commercially available ELISA kits (Invitrogen) were used according to manufacturer's instructions.

4.2.7. Bax activation assay

Bring all reagents, except the human Bax-α Standard, to room temperature for at least 30 minutes prior to opening. The human Bax-α Standard solution should not be left at room temperature for more than 10 minutes. All standards, controls and samples should be run in duplicate. Refer to the Assay Layout Sheet to determine the number of wells to be used and put any remaining wells with the desiccant back into the pouch and seal the ziploc. Store unused wells at 4 °C. Pipet 100 µL of Assay Buffer into the S0 (0 pg/mL standard) wells. Pipet 100 µL of Standards #1 through #6 into the appropriate wells. Pipet 100 µL of the Samples into the appropriate wells. Tap the plate gently to mix the contents. Seal the plate and incubate at room temperature on a plate shaker for 1 hour at ~500 rpm. Empty the contents of the wells and wash by adding 400 µL of wash solution to every well. Repeat the wash 4 more times for a total of **5 washes**. After the final wash, empty or aspirate the wells and firmly tap the plate on a lint free paper towel to remove any remaining wash buffer. Pipet 100 µL of yellow Antibody into each well, except the Blank. Seal the plate and incubate at

room temperature on a plate shaker for 1 hour at ~500 rpm. Empty the contents of the wells and wash by adding 400 μL of wash solution to every well. Repeat the wash 4 more times for a total of **5** washes. After the final wash, empty or aspirate the wells and firmly tap the plate on a lint free paper towel to remove any remaining wash buffer. Add 100 μL of blue Conjugate to each well, except the Blank. Seal the plate and incubate at room temperature on a plate shaker for 30 minutes at ~500 rpm. Empty the contents of the wells and wash by adding 400 μL of wash solution to every well. Repeat the wash 4 more times for a total of **5 washes**. After the final wash, empty or aspirate the wells and firmly tap the plate on a lint free paper towel to remove any remaining wash buffer. Pipet 100 μL of Substrate Solution into each well. Incubate for 30 minutes at room temperature on a plate shaker at ~500 rpm. Pipet 100 μL Stop Solution to each well. Blank the plate reader against the Blank wells, read the optical density at 450 nm. Calculate the average net Optical Density (OD) bound for each standard and sample by subtracting the average Blank OD from the average OD for each standard and sample. Using linear graph paper, plot the Average Net OD for each standard versus Bax concentration in each standard. Approximate a straight line through the points. The concentration of Bax in the unknowns can be determined by interpolation.

4.2.8. Bcl-2 inhibition assay

Mix all reagents thoroughly without foaming before use. Wash the microwells twice with approximately 300 μL Wash Buffer per well with thorough aspiration of microwell contents between washes. Take caution not to scratch the surface of the microwells. After the last wash, empty the wells and tap microwell strips on absorbent pad or paper towel to remove excess Wash Buffer. Use the microwell strips immediately after washing or place upside down on a wet absorbent paper for not longer than 15 minutes. Do not allow wells to dry. Add 100 μL of Sample Diluent in duplicate to all standard wells and to the blank wells. Prepare standard (1:2 dilution) in

duplicate ranging from 32 ng/mL to 0.5 ng/mL. Add 100 µL of Sample Diluent, in duplicate, to the blank wells. Add 80 µL of Sample Diluent, in duplicate, to the sample wells. Add 20 µL of each Sample, in duplicate, to the designated wells. Add 50 µL of diluted biotin-conjugate to all wells, including the blank wells. Cover with a plate cover and incubate at room temperature, on a microplate shaker at 100 rpm if available, for 2 hours. Remove plate cover and empty the wells. Wash microwell strips 3 times as described in step 2. Add 100 µL of diluted Streptavidin-HRP to all wells, including the blank wells. Cover with a plate cover and incubate at room temperature, on a microplate shaker at 100 rpm if available, for 1 hour. Remove plate cover and empty the wells. Wash microwell strips 3 times as described in step 2. Proceed to the next step. Pipette 100 µl of mixed TMB Substrate Solution to all wells, including the blanks. Incubate the microwell strips at room temperature (18° to 25°C) for about 15 minutes, if available on a rotator set at 100 rpm. Avoid direct exposure to intense light. The point, at which the substrate reaction is stopped, is often determined by the ELISA reader. Many ELISA readers record absorbance only up to 2.0 O.D. Therefore, the color development within individual microwells must be watched by the person running the assay and the substrate reaction stopped before positive wells are no longer properly detectable. Stop the enzyme reaction by quickly pipetting 100 µL of Stop Solution into each well, including the blank wells. It is important that the Stop Solution is spread quickly and uniformly throughout the microwells to completely inactivate the enzyme. Results must be read immediately after the Stop Solution is added or within one hour if the microwell strips are stored at 2 - 8°C in the dark. Read absorbance of each microwell on a spectrophotometer using 450 nm as the primary wavelength.

4.3. Wound Healing Assay of MCF-7 Breast Adenocarcinoma

4.3.1. Cell culture

MCF-7 Breast Adenocarcinoma cell line was obtained from Nawah Scientific Inc., (Mokatam, Cairo, Egypt). Cells were maintained in DMEM media supplemented with 100 mg/mL of streptomycin, 100 units/mL of penicillin and 10% of heat-inactivated fetal bovine serum in humidified, 5% (v/v) CO_2 atmosphere at 37 °C.

4.3.2. Wound healing assay

Cells were plated at a density of $2x10^5$/well onto a coated 12-well plate for scratch wound assay and cultured overnight in 5% FBS-DMEM at 37 °C and 5% CO_2. On the next day, horizontal scratches were introduced into the confluent monolayer; the plate was washed thoroughly with PBS, control wells were replenished with fresh medium while drug wells were treated with fresh media containing drug 26 and 27. Images were taken using an inverted microscope at the indicated time intervals. The plate was incubated at 37 °C and 5% CO_2 in-between time points. The acquired images are displayed below and were analyzed by MII ImageView software version 3.7 (Supporting Information)

4.4. Wound Healing Assay Calculations

4.4.1. Wound Width

Wound width can be calculated as the average distance between the edges of the scratches; the wound width decreases as cell migration is induced. The results of compounds 26 and 27 were displayed as mean ± standard deviation (Table S3).

4.4.2. Migration Rate

Migration rate is calculated by dividing the time spent in migration according to the following formula: $R_m=W_i-W_f$ / t.

where: R_m is the rate of cell migration, W_i is the average initial wound width, W_f is the average final wound width, and t is the duration of migration (in hours). Values of test compounds (26 and 27) were listed in Table S3.

4.4.3. Wound Closure Percentage

The migration rate can be expressed as the percentage of area reduction of wound closure, which increases as cells migrate over time. Wound closure percentage is calculated according to the following formula: $(At = 0hr – At = \Delta h / At = 0h) \times 100$

where: At= 0 hr is the average area of the wound measured immediately after scratching (time zero), and At = Δ h is the average area of the wound measured h hours after the scratch is performed. Values of test compounds (26 and 27) were listed in Table ????????.

8. Determination of Lipophilicity using RP-TLC Chromatographic Method

Chromatographic analysis was carried out on SORBTECH TLC silica gel C 18-W w/UV254 aluminium sheets (4 × 8 cm, and 0.15 mm layer thickness; Catalo # 2733187, Lot # 081006P, Norcross GA 30071, USA). The mobile phases were prepared by mixing different proportions of double-distilled water and organic modifier (Methanol; Merck Germany). Standard solution (0.1 mg/mL) of each of the studied compounds (14, 19, 22, and 26-28) was prepared in methanol (MeOH). The content of the organic modifier (MeOH) was varied between 70 and 95% (v/v) with an increment of 5%. The upper and lower content of the organic modifier (MeOH) depends on the linearity between R_M and the organic modifier concentration. The concentration ranges for the tested organic modifier were used, where the upper value of the range indicates that the analyte elutes near the solvent front, and the lower value implies that the analyte is very near to the starting line. Then, the chromatographic chambers were saturated with the mobile phase for 30 min. TLC was performed on RP-TLC plates (4 × 8 cm pieces). The standard solutions (5 μL) were spotted

in triplicate on the plates 5 mm apart, 10 mm from the bottom edges, and 5 mm from the side edges. The plates were air-dried and then developed in the chromatographic chambers. Linear ascending plate development was performed until a migration distance of 60 mm from the origin was reached. After development, the plates were removed and air-dried. Then, spots were visualized under UV light at λ = 254 nm. Finally, R_f values were calculated. R_m values were derived using the R_f of solutes in mobile solvent systems, including water and methanol (organic modifier), followed by construction of a relationship between R_m and C (percentage of MeOH). A linear regression analysis was carried out using Excel 2010 (Microsoft Office) to obtain lipophilicity of the chromatographic descriptors (R_{Mo}, *b*, and C_o) as calculated from the following equations: $R_M = \log(1/R_f - 1)$, $R_M = R_{Mo} + bC$, $C_o = -R_{Mo}/b$, where R_{Mo} is the relative lipophilicity, and is obtained by extrapolation of the R_M value to 0% v/v of the organic modifier (MeOH) in the mobile phase; *b* is the slope of the regression line; and finally C_o represents the hydrophobicity per unit of the specific hydrophobic surface area and is the concentration of an organic modifier in the mobile phase for which the solute is equally distributed between the two phases.

4.3. Statistical analysis

Computerized Prism 5 program was used to statistically analyzed data using one-way ANOVA test followed by Tukey's as post ANOVA for multiple comparison at $P \leq .05$. Data were presented as mean ± SEM.
